# α,β‐Unsaturated Gold(I) Carbenes by Tandem Cyclization and 1,5‐Alkoxy Migration of 1,6‐Enynes: Mechanisms and Applications

**DOI:** 10.1002/chem.201602347

**Published:** 2016-08-16

**Authors:** Pilar Calleja, Óscar Pablo, Beatrice Ranieri, Morgane Gaydou, Anthony Pitaval, María Moreno, Mihai Raducan, Antonio M. Echavarren

**Affiliations:** ^1^ Institute of Chemical Research of Catalonia (ICIQ)Barcelona Institut of Science and Technology (BIST)Av. Països Catalans 1643007TarragonaSpain; ^2^ Departament de Química Analítica i Química OrgánicaUniversitat Rovira i VirgiliC/ Marcel⋅li Domingo s/n43007TarragonaSpain

**Keywords:** cycloisomerization, cyclopropanation, density functional calculations, gold, rearrangement

## Abstract

1,6‐Enynes bearing OR groups at the propargyl position generate α,β‐unsaturated gold(I)‐carbenes/ gold(I) stabilized allyl cations that can be trapped by alkenes to form cyclopropanes or 1,3‐diketones to give products of α‐alkylation. The best migrating group is *p*‐nitrophenyl ether, which leads to the corresponding products without racemization. Thus, an improved formal synthesis of (+)‐schisanwilsonene A has been accomplished. The different competitive reaction pathways have been delineated computationally.

## Introduction

**Scheme 1:**
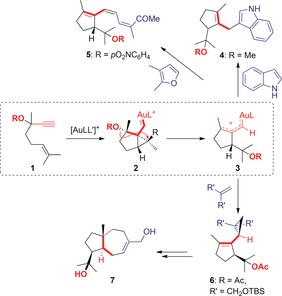
Intermolecular trapping of the intermediates of the gold‐catalyzed cyclization/1,5‐OR migration of enynes **1**.

The study of gold(I)‐catalyzed reactions of 1,*n*‐enynes has led to the discovery of a wealth of cyclization modes, including mechanistically intriguing skeletal rearrangements and nucleophilic addition reactions to cycloaddition processes.^1^ In this context, we recently found that 1,6‐enynes such as **1** bearing propargyl alcohols, ethers, or silyl ethers react with gold(I) catalysts through the usual type of highly delocalized cyclopropyl gold(I) intermediates **2**,^2^ which then undergo a new type of 1,5‐migration of the OR groups to generate species **3** 3 that we postulated as intermediate between α,β‐unsaturated gold(I) carbenes and gold(I)‐stabilized allyl cations.^2^, ^4^ In the presence of carbon nucleophiles such as indole or furans, products **4** 3 or trienes such as **5** 5 were obtained by Friedel–Crafts‐type reactions (Scheme 1). Intermediates **3** can also react with electron‐rich alkenes to form the corresponding cyclopropanes,^3^ a reaction which is also characteristic of gold(I) carbenes **2**.^6^, ^7^, ^8^ We demonstrated the potential of this tandem cyclization/1,5‐OR migration/cyclopropanation to form products **6** that were key intermediates in the first total synthesis of the natural sesquiterpene (+)‐schisanwilsonene A (**7**).^9^ 1

**Scheme 2:**
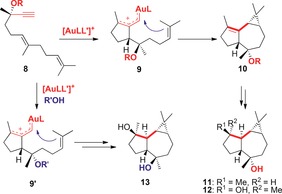
Intramolecular trapping of the intermediates of the gold‐catalyzed cyclization/1,5‐OR migration of dienynes **8**.

This type of gold(I)‐catalyzed 1,5‐migration has been found to compete^10^ with 1,2‐ and 1,3‐migrations of propargylic carboxylate groups.^10^ Related processes have been found in the gold(I)‐catalyzed reactions of dienynes **8**, which undergo cyclization/1,5‐OR migration/intramolecular cyclopropanation through intermediates **9** to form stereoselectively hexahydroazulenes **10**,^3^ which were the key intermediates in our total synthesis of the sesquiterpernes (−)‐epiglobulol (**11**) and (−)‐4β,7α‐aromadendranediol (**12**) (Scheme 2).^11^ Alternatively, when the gold(I)‐catalyzed reaction was performed in the presence of allyl alcohol, this external nucleophile reacted to give **9′**, which underwent intramolecular cyclopropanation to give rise to the sesquiterpene (−)‐4α,7α‐aromadendranediol (**13**).^11^, 2

The proposed mechanism for these inter‐ and intramolecular reactions was based on the isolation of diverse products but not on a rigorous study of this intriguing process and its several possible competitive cycloisomerization pathways. Furthermore, although the chirality transfer in the intramolecular processes was satisfactory, partial racemization was observed in the formation of intermediates **2** when RO=AcO, which led to (+)‐schisanwilsonene A (**7**) with 9:1 e.r. Here we report that 1,3‐dicarbonyl compounds can also be used as the C‐nucleophiles to trap the putative α,β‐unsaturated gold(I) carbenes **3** leading to products of formal alkylation. This and additional studies on the intermolecular trapping of intermediates **3** with alkenes have allowed selecting *p*‐nitrophenyl ether as the protecting group of choice in these reactions. This led us to develop an improved formal synthesis of (+)‐schisanwilsonene A (**7**) in which the key step proceeds with total retention of configuration. We have also found cases in which a skeletal rearrangement takes place preferentially to form six‐membered ring compounds. A detailed computational study has been performed to understand the mechanisms of these complex transformations.

## Results and Discussion

### Selection of the best OR migration group

1,3‐Dicarbonyl compounds react as C‐nucleophiles with 1,6‐enynes in the presence of gold(I) catalysts by formal attack at the alkene via opening of the cyclopropane of intermediates of type **2**.1h We therefore decided to examine whether enynes **1** would react with this type of nucleophiles at the alkene, through intermediates **2**, or at the terminal alkyne carbon through intermediates **3**. In the event, reaction of enynes **1** with 1,3‐diphenyl‐1,3‐propandione in the presence of gold(I) complexes gave products of α‐alkylation of the dicarbonyl compounds **14** by trapping of intermediates **3** at C‐1 (Table 1). Using catalyst [(JohnPhos)Au(NCMe)]SbF_6_ (**A**), the best migrating group proved to be *p*‐nitrophenyl (PNP) ether in substrate **1 a**, which led to adduct **14 a** in 58 % yield after 30 min at 24 °C (Table 1, entry 1). *p*‐Anisyl ether derivative **1 e** gave **14 e** in a moderate 44 % yield (Table 1, entry 5), whereas lower yields (14–18 %) were obtained with the free alcohol **1 b**, methyl ether **1 c**, and acetate **1 d**, and benzyl ethers **1 f**, **g** failed to give any of the expected products **14 f**, **g** (Table 1, entries 2–7). Poor results were obtained with catalysts **B** and **C** bearing *t*BuXphos as the ligand (Table 1, entries 8 and 9). The best results were obtained using the less electrophilic catalysts **D**–**G** with more donating NHC ligands (Table 1, entries 10–13), which have been proposed to enhance the carbene‐like character of the intermediates in gold(I)‐catalyzed reactions.1g,1h, 1

**Table 1:** Gold(I)‐catalyzed reaction of enynes **1 a**–**g** with 1,3‐diphenyl‐1,3‐propandione. 
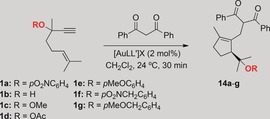

Entry	[AuLL′]X	**1 a**–**g**	**14 a–g** (yield) [%]
1	**A**	**1 a**	**14 a** (58)
2	**A**	**1 b**	**14 b** (14)
3	**A**	**1 c**	**14 c** (18)
4	**A**	**1 d**	**14 d** (14)
5	**A**	**1 e**	**14 e** (44)
6	**A**	**1 f**	**14 f** (−)
7	**A**	**1 g**	**14 g** (−)
8^[a]^	**B**	**1 a**	**14 a** (8)
9^[a]^	**C**	**1 a**	**14 a** (8)
10	**D**	**1 a**	**14 a** (67)
11	**E**	**1 a**	**14 a** (67)
12	**F**	**1 a**	**14 a** (62)
13	**G**	**1 a**	**14 a** (71)

[a]=70–75 min 
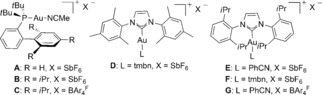

.

1,6‐Enyne **1 a** reacted smoothly with other 1,3‐dicarbonyl compounds and β‐ketoesters as the C‐nucleophiles using the optimal IPr gold(I) complex **G** to form adducts **14 h**–**n** in 59–82 % yield in 30 min at 24 °C (Table 2).2

**Table 2:** Products of the reaction of 1,6‐enyne **1 a** with 1,3‐dicarbonyl compounds and β‐ketoesters in the presence of catalyst **G** (24 °C, 30 min).

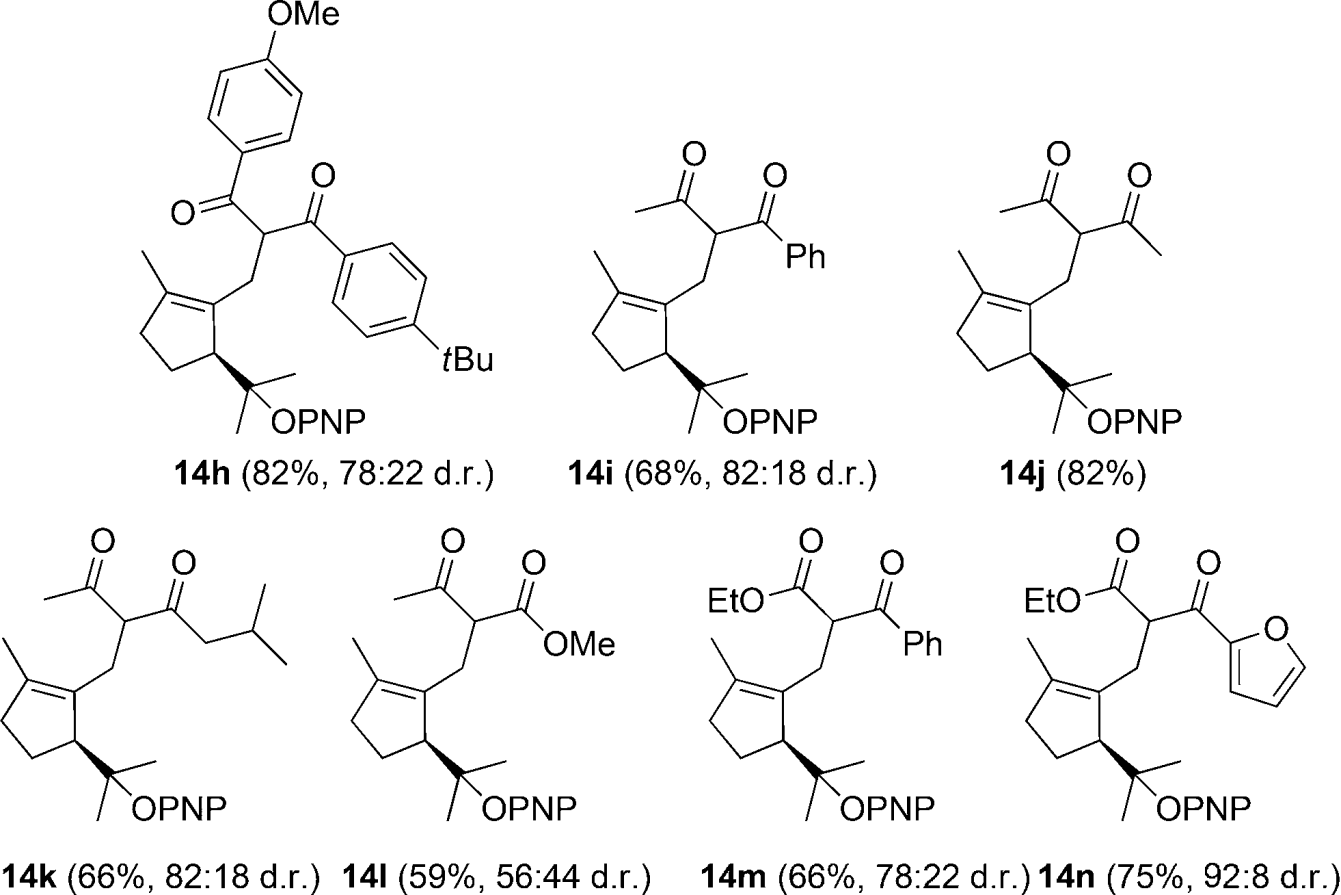

**Scheme 3:**
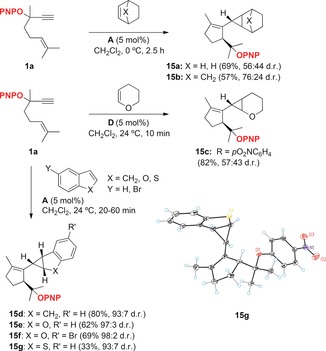
Gold(I)‐catalyzed cyclization/1,5‐OR migration/intermolecular cyclopropanation of **1 a**.

*p*‐Nitrophenyl ether **1 a** was also the substrate of choice for the cyclization/1,5‐OR migration/intermolecular cyclopropanation with cyclohexene and norbornene to form cyclopropanes **15 a**, **b** in satisfactory yields as mixtures of *exo* and *endo* diastereomers by using catalyst **A** under very mild conditions (Scheme 3). Dihydropyrane reacted similarly to give **14 c**, although in this case catalyst **D** gave better results. Indene, benzofuran, 5‐bromobenzofuran, and benzothiophene gave the corresponding adducts **15 d**–**g** more stereoselectively. The configuration of the mayor product **15 g** in the reaction with benzothiophene was assigned by X‐ray diffraction.^12^ This product was formed in lower yield most likely as a consequence of the inhibition of the catalytic reaction by coordination of gold(I) to the thioether **15 g**. Interestingly, in the last three cases, the electron‐rich heterocycles undergo cyclopropanation, which is in contrast to what we observed previously for indole, which gave product **4** after formal alkylation at C‐3 (Scheme 1).^3^, 3

### A second‐generation formal synthesis of (+)‐schisanwilsonene A

**Scheme 4:**
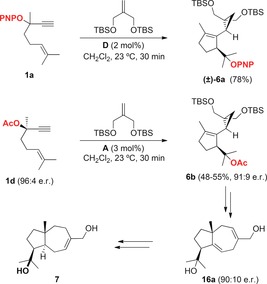
First‐generation synthesis of (+)‐schisanwilsonene A (**7**) via cyclization/1,5‐OR migration/cyclopropanation.^9^

The gold(I)‐catalyzed reaction of racemic **1 a** with the bis‐TBS ether of 2‐methylenepropane‐1,3‐diol gave **6 a** in good yield as a single diasteromer (Scheme 4).^9^ Enantioenriched acetate **1 d** (96:4 e.r.) reacted in slightly lower yield to afford **6 b** in 91:9 e.r. This partial racemization was explained as a result of a 1,2‐acyloxy rearrangement competing as a minor pathway.^9^, 4

**Scheme 5:**
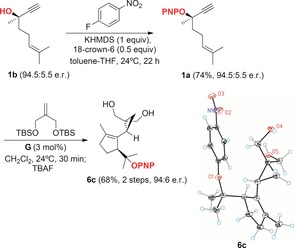
Enantio‐retentive synthesis of key intermediate **6 c** in the synthesis of (+)‐schisanwilsonene A (**7**).

Although the formation of enantioenriched **6 b** allowed us to complete the first total synthesis of (+)‐schisanwilsonene A (**7**) via hexahydroazulene diol **16 a** (90:10 e.r.) (Scheme 4), a better solution has now been found by using enantioenriched **1 a** as the substrate for the cyclization/1,5‐OR migration/cyclopropanation cascade. Thus, alcohol **1 b** was protected as the PNP ether by nucleophilic aromatic substitution of its potassium salt with *p*‐fluoronitrobenzene and 18‐crown‐6 to give **1 a** with full retention of the configuration (94.5:5.5 e.r.) (Scheme 5). The key gold(I)‐catalyzed reaction of **1 a** with the bis‐TBS ether of 2‐methylenepropane‐1,3‐diol, followed by desilylation with TBAF, provided diol **6 c** in 68 % yield without detectable racemization, within experimental error (94:6 e.r.). The configuration of **6 c** was determined by X‐ray diffraction.^12^ This result demonstrates that the enyne cyclization/1,5‐OR migration/cyclopropanation cascade takes place without any racemization, which excludes the involvement of a propargyl carbocation as an intermediate in the process. In the racemic series, **6 c** has been converted into hexahydroazulene diol **16 a** according to the original sequence followed by a two‐setp deprotection of the PNP group (76 % yield).^13^, 5

### Exceptions to the rule: no migration of the propargyl group

**Scheme 6:**
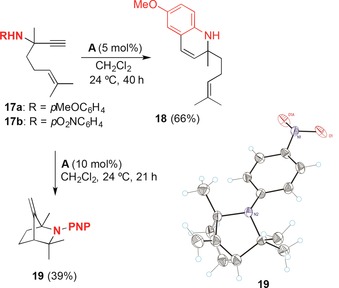
Reaction of propargyl amines **17 a**, **b** with gold(I) catalyst **A**.

We also tried the reaction of 1,6‐enynes **17 a**, **b** with a propargylic amine (Scheme 6). In the case of **17 a**, the electron‐rich *p*‐methoxyphenyl amine undergoes a gold(I)‐catalyzed intramolecular hydroarylation with the terminal alkyne^14^ to give dihydroquinoline **18**, whereas PNP derivative **17 b** afforded 7‐methylene‐2‐azabicyclo[2.2.1]heptane **19** as the major product, whose structure was determined by X‐ray diffraction.^12^, 6

**Scheme 7:**
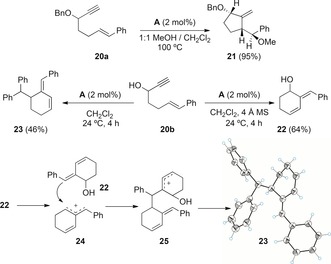
Cyclizations of enynes **20 a**, **b** and **22** with gold(I) catalyst **A** without OR migration and the proposed mechanism for the formation of **23**.

We had reported that 1,6‐enyne **20 a** reacts with methanol in the presence of catalyst **A** to give stereoselectively adduct **21** in which the migration of the benzyloxy group has not taken place^15^ (Scheme 7). Interestingly, alcohol **20 b** also reacted in the presence of catalyst **A** without migration of the OH group to give **22**, the product of an *endo*‐type single cleavage rearrangement.^16^ This reaction was better performed in the presence of 4 Å molecular sieves. Surprisingly, in the absence of molecular sieves, diene **23** was obtained as the major product of the reaction, whose structure was determined by X‐ray diffraction. A speculative mechanism for the formation of this unexpected product could involve a reaction of **22** with allyl cation **24** to form a new allyl cation **25**, followed by aromatization by proton loss and dehydration (Scheme 7).7

**Figure 1:**
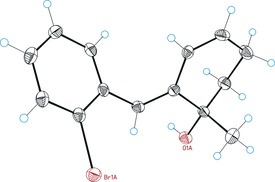
X‐ray diffraction structure of product **27 h** (Table 3, entry 8).

1,6‐Enynes **26 a**–**h** with tertiary propargyl hydroxyl or trimethylsilyloxy groups and different aryl groups at the alkene also react with catalyst **A** to give *endo*‐type single cleavage rearrangement products **27** under mild conditions (Table 3). The reaction proceeds satisfactorily with substrates bearing phenyl or aryl groups with electron‐withdrawing substituents (Table 3, entries 1–5 and 8; Figure 1), whereas enynes **26 f**, **g** with a more electron‐rich anisyl group gave complex reaction mixtures (Table 3, entries 6 and 3, 1). Somewhat surprisingly, the reaction of the corresponding methyl ethers led only to decomposition.

**Table 3:** Gold(I)‐catalyzed reaction of 1,6‐enynes **26 a**–**h** to give *endo*‐type single‐cleavage rearrangement products **27 a**–**h**. 
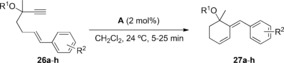

Entry	Enyne	R^1^	R^2^	Product (yield) [%]
1	**26 a**	H	H	**27 a** (52)
2	**26 b**	TMS	H	**27 b** (95)
3	**26 c**	H	*p‐*NO_2_	**27 c** (88)
4	**26 d**	TMS	*p‐*NO_2_	**27 d** (72)
5	**26 e**	H	*o‐*NO_2_	**27 e** (98)
6	**26 f**	H	*p‐*OMe	**27 f** (−)
7	**26 g**	TMS	*p‐*OMe	**27 g** (−)
8	**26 h**	H	*o‐*Br	**27 h** (84)

### Mechanistic discussion

**Scheme 8:**
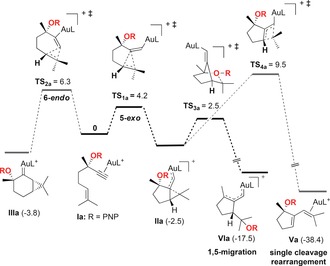
Alternative reaction pathways for the evolution of gold(I) complexes **Ia** into the product of 1,5‐OR migration **VIa** (L=PMe_3_). DFT calculations (B3LYP, 6‐31G(d,p) (C, H, P, O, N) and SDD (Au), CH_2_Cl_2_). Values for free energies in kcal mol^−1^.

**Scheme 9:**
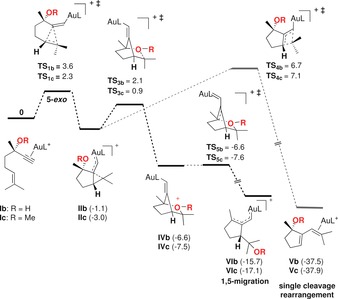
Alternative reaction pathways for the evolution of gold(I) complexes **Ib** and **Ic** into the products of 1,5‐OR migration **VIb** and **VIc**. DFT calculations (B3LYP, 6‐31G(d,p) (C, H, P, O) and SDD (Au), CH_2_Cl_2_). Values for free energies in kcal mol^−1^.

We examined computationally the evolution of enynes **Ia**–**c** coordinated with AuL^+^ (L=PMe_3_). In all cases, in agreement with calculations, **Ia**–**c** reacted by 5‐*exo*‐*dig* pathways to form preferentially **IIa**–**c** (Schemes 8 and 9), which correspond to intermediates of type **2** in Scheme 1.^17^ The alternative 6‐*endo*‐*dig* pathway leading to **IIIa**–**c** was less favorable in all cases. Whereas in the case of PNP‐protected intermediate **IIa**, the migration to form **VIa** proceeds in a direct manner through **TS_3a_** (Scheme 8), the migration of the OR group in complexes **IIb** and **IIc** proceeds via bicyclic intermediates **IVb** and **IVc**, which then lead to products of 1,5‐migration **VIb** and **VIc** through very low barrier transition states **TS_5b_** and **TS_5c_**, respectively (Scheme 9). The alternative evolution of **IIa**–**c** to products of single‐cleavage rearrangement **Va**–**c** was found to be thermodynamically the most favorable pathway,^18^ although kinetically the 1,5‐migration is more favorable as **TS_4a_**_–**c**_ have higher energies than **TS_3a_**_–**c**_ (Schemes 8 and 9).8, 9

**Figure 2:**
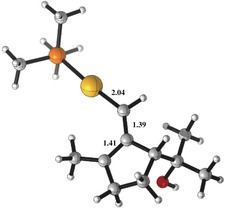
Computed structure for minimum **VIb** (Scheme 9). Bond lengths are in Å.

The calculated structure for minimum **VIb** shows very similar C−C bond lengths of 1.41 and 1.39 Å (Figure 2). The Au−C bond length is 2.04 Å, which might correspond to a single metal–carbon bond, and is similar to that found in well‐characterized heteroatom‐stabilized gold(I) carbenes.^3^, ^4^ Overall, the calculated structure fits better with a gold(I)‐stabilized allylic cation.2

The observed reactivity trends when L=PMe_3_ are reproduced in cases where the ligand on gold(I) is changed to bulkier PPh_3_ phosphine or the model NHC ligand 1,3‐dimethylimidazol‐2‐ylidene (Table 4).4

**Table 4:** Evaluation on the ligand effect for the computed trends of gold(I) complex **Ib**.^[a]^

	PMe_3_	PPh_3_	NHC^[b]^
**Ib**	0.0	0.0	0.0
**TS_1b_**	3.6	5.8	9.5
**IIb**	−1.1	−1.5	1.6
**TS_2b_**	7.4	9.3	11.3
**IIIb**	−3.1	−2.5	0.1
**TS_3b_**	2.1	4.4	7.9
**IVb**	−6.6	−4.3	−2.3
**TS_5b_**	−6.6	−5.1	−0.7
**VIb**	−15.7	−13.9	−13.3
**TS_4b_**	6.7	7.0	11.0
**Vb**	−37.5	−35.4	−33.1

[a] Δ*G* energies are given in kcal mol^−1^. [b] 1,3‐Dimethylimidazol‐2‐ylidene.

**Scheme 10:**
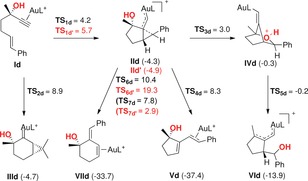
Alternative reaction pathways for the evolution of gold(I) complex **Id** into the product of 1,5‐OR migration **VId**. Numbers in red correspond to the formation and evolution of **IId“**, the diastereomer of **IId**. DFT calculations (B3LYP, 6‐31G(d,p) (C, H, P, O) and SDD (Au), CH_2_Cl_2_). Values for free energies in kcal mol^−1^.

DFT calculations for the reaction of gold(I) complex **Id** led to much less clear‐cut results (Scheme 10). Although the 5‐*exo*‐*dig* pathway leading to **IId** was again more favorable than the 6‐*endo*‐*dig* cyclization to form **IIId**, the 1,5‐migration of **IId** through **IVd** to form **VId** was found to be the kinetically most favorable pathway, which is not what was observed experimentally for **20 b** and **26 a**–**h**.10

## Conclusions

In general, 1,6‐enynes bearing OR groups at the propargyl position react through intermediates that can be formulated in a simplified manner as α,β‐unsaturated gold(I) carbenes, which react in general with alkenes to form cyclopropanes or 1,3‐diketones to form products of α‐alkylation. We have found that among the various migrating OR groups, *p*‐nitrophenyl ether gives the best results. In addition, we have established that the gold(I)‐catalyzed enyne cyclization/1,5‐OR migration/cyclopropanation cascade takes place without racemization, which demonstrates that propargyl carbocations are not formed under the reaction conditions. This has been applied for the preparation of key intermediates for the synthesis of (+)‐schisanwilsonene A with higher enantiomeric purity. DFT calculations suggest that after the initial cyclization, the 1,5‐OR migration proceeds stepwise through a cyclic intermediate although the cleavage occurs through a very low barrier. However, additional mechanistic work is still required to understand why in cases in which propargyl group does not migrate an *endo*‐type single‐cleavage rearrangement is the most favorable reaction pathway.

## Supporting information

As a service to our authors and readers, this journal provides supporting information supplied by the authors. Such materials are peer reviewed and may be re‐organized for online delivery, but are not copy‐edited or typeset. Technical support issues arising from supporting information (other than missing files) should be addressed to the authors.

Supplementary
