# Supplementary material for: α,β‐Unsaturated Gold(I) Carbenes by Tandem Cyclization and 1,5‐Alkoxy Migration of 1,6‐Enynes: Mechanisms and Applications
Source: Chemistry. 2016 Aug 16;22(38):13613–8. doi: 10.1002/chem.201602347 (PMC5132047; doi:10.1002/chem.201602347)
Supplement: Supplementary file 1 — Supplementary [file CHEM-22-13613-s001.pdf]

CHEMISTRY
A European Journal

Supporting Information

α,β-Unsaturated Gold(I) Carbenes by Tandem Cyclization and 1,5-Alkoxy Migration of 1,6-Enynes: Mechanisms and Applications

Pilar Calleja,[a] Óscar Pablo,[a] Beatrice Ranieri,[a] Morgane Gaydou,[a] Anthony Pitaval,[a] María Moreno,[a] Mihai Raducan,[a] and Antonio M. Echavarren*[a, b]

chem_201602347_sm_miscellaneous_information.pdf

Supporting Information

a,b-Unsaturated Gold(I) Carbenes by Tandem Cyclization and 1,5-Alkoxy Migration of 1,6-Enynes: Mechanisms and Applications

Pilar Calleja,[a] Óscar Pablo,[a] Beatrice Ranieri,[a] Morgane Gaydou,[a] Anthony Pitaval,[a] María Moreno,[a] Mihai Raducan,[a] and Antonio M. Echavarren*[a,b]

[a] Institute of Chemical Research of Catalonia (ICIQ), Barcelona Institute of Science and Technology, Av. Països Catalans 16, 43007 Tarragona (Spain).

[b] Departament de Química Orgànica i Analítica, Universitat Rovira y Virgili, C/ Marcel·lí Domingo s/n, 43007 Tarragona (Spain).

Email: aechavarren@iciq.es

TABLE OF CONTENTS

1. General Information 3

2. Gold(I)-carbene Trapping with 1,3-Diketones 4

3. Gold(I)-catalyzed Cyclization / 1,5-OR Migration / Intermolecular Cyclopropanation 10

4. A Second Generation Formal Synthesis of (+)-Schisanwilsonene A 14

6. Endo-type Skeletal Rearrangement instead of OR migration 25

7. X-Ray crystallographic data 34

8. NMR Spectra 41

9. DFT calculations 96

10. References 211

1. General Information

Unless otherwise stated, reactions were carried out under argon atmosphere in solvents dried by passing through an activated alumina column on a PureSolv^{TM} solvent purification system (Innovative Technologies, Inc., MA). Analytical thin layer chromatography was carried out using TLC-aluminum sheets with 0.2 mm of silica gel (Merck GF_{234}) using UV light as the visualizing agent and an acidic solution of vanillin or anisaldehyde in ethanol as the developing agent. Chromatographic purifications were carried out using flash grade silica gel (SDS Chromatogel 60 ACC, 40-63 mm), neutral aluminum oxide (SDS, 63-200 μm) or basic aluminum oxide (SDS, 50-200 μm). Organic solutions were concentrated under reduced pressure on a Büchi rotary evaporator.

NMR spectra were recorded at 298 K (unless otherwise stated) on a Bruker Avance 300, Bruker Avance 400 Ultrashield and Bruker Avance 500 Ultrashield apparatuses. The signals are given as d / ppm (multiplicity, coupling constant (Hertz), number of protons) downfield from tetramethylsilane, with calibration on the residual protio-solvent used (d_H = 7.26 ppm and d_C = 77.16 ppm for $CDCl_3$, d_H = 5.32 ppm and d_C = 53.84 ppm for CD_2Cl_2). Mass spectra were recorded on a Waters Micromass LCT Premier (ESI), Waters Micromass GCT (EI, CI) and Bruker Daltonics Autoflex (MALDI) spectrometers. Melting points were determined using a Büchi melting point apparatus.

Crystal structure determinations were carried out using a Bruker-Nonius diffractomer equipped with an APPEX 2 4K CCD area detector, a FR591 rotating anode with MoK_a radiation, Montel mirrors as monochromator and a Kryoflex low temperature device (T = –173 ºC). Full-sphere data collection was used with w and j scans. *Programs used*: Data collection APEX-2, data reduction Bruker Saint V/.60A and absorption correction SADABS. Structure Solution and Refinement: Crystal structure solutions were achieved using direct methods as implement in SHELXTL and visualized using the program XP. Missing atoms were subsequently located from difference Fourier synthesis and added to the atom list. Least-squares refinement on F2 using all measured intensities was carried out using the program SHELXTL. All non-hydrogen atoms were refined including anisotropic displacement parameters.

HPLC analysis was carried out on an Agilent Tehcnologies instrument HPLC 1100 series with VWD detector or HPLC 1200 series with DAD detector.

All reagents were used as purchased and used with no further purification, unless otherwise stated.

2. Gold(I)-carbene Trapping with 1,3-Diketones

2.1. Synthesis of Functionalized 1,6-Enynes

1-((3,7-Dimethyloct-6-en-1-yn-3-yl)oxy)-4-nitrobenzene (1a)

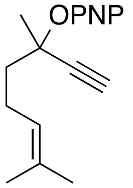

A solution of propargylic alcohol **1b** (1.0 g, 6.57 mmol) in 7.0 mL of dry acetonitrile was added DBU (1.47 mL, 9.85 mmol) and trifluoroacetic anhydride (1.42 mL, 10.18 mmol) at -15ºC. After stirring at this temperature for 2h a solution of DBU (1.47 mL, 9.85 mmol), $CuCl_2 \cdot 2H_2O$ (11.0 mg, 0.066 mmol) and *p*-nitrophenol (1.01g, 7.23 mmol) was added dropwise. After stirring for 30min at room temperature NH_4Cl (aq. sat.) was added, the mixture was extracted with Et_2O, washed with brine, dried over Na_2SO_4 and the solvents were evaporated. Purification by column flash chromatography (cyclohexane / EtOAc, 33 / 1 to 20 / 1) yielded protected alcohol as a yellow oil (0.99 g, 3.61 mmol, 55%). ^{1}H NMR (400 MHz, $CDCl_3$) δ 8.19-8.15 (m, 2H), 7.32-7.28 (m, 2H), 5.14 (ddq, *J* = 8.6, 5.8, 1.5 Hz, 1H), 2.71 (s, 1H), 2.26 (dtq, *J* = 38.1, 13.6, 6.1 Hz, 2H), 2.00 (ddd, *J* = 13.6, 11.3, 5.2 Hz, 1H), 1.90 (ddd, *J* = 13.5, 11.5, 5.2 Hz, 1H), 1.70 (s, 3H), 1.68 (s, 3H), 1.62 (s, 3H). ^{13}C NMR (101 MHz, $CDCl_3$) δ 161.3, 142.0, 132.6, 125.2 (2C), 122.9, 119.0 (2C), 83.5, 76.6, 75.9, 42.4, 26.6, 25.6, 22.9, 17.6. HRMS-ESI: m/z calcd. for $C_{16}H_{20}NO_3$ $[M+H]^+$: 274.1443, found: 274.1432.

1-((3,7-Dimethyloct-6-en-1-yn-3-yl)oxy)-4-methoxybenzene (1e)

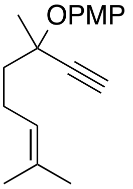

Prepared according to the above-mentioned procedure for the synthesis of compound **1a** and isolated as a yellow oil (48%). ^{1}H NMR (400 MHz, $CDCl_3$) δ 7.11 (d, *J* = 9.0 Hz, 2H), 6.80 (d, *J* = 9.0 Hz, 2H), 5.19-5.10 (m, 1H), 3.78 (s, 3H), 2.54 (s, 1H), 2.36-2.19 (m, 2H), 1.95-1.85 (m, 1H), 1.84-1.74 (m, 1H), 1.70 (d, *J* = 1.7 Hz, 3H), 1.63 (s, 3H), 1.52 (s, 3H). ^{13}C NMR (101 MHz, $CDCl_3$) δ 155.7, 148.9, 132.1, 123.7 (2C), 123.6 (2C), 113.8, 85.4, 76.1, 74.9, 55.5, 42.3, 26.9, 25.7, 23.3, 17.7. HRMS-ESI: *m/z* calcd. for $C_{17}H_{22}NaO_2$ $[M+Na]^+$: 281.1517, found: 281.1512.

2.2. General Procedure for the Gold(I)-carbene Trapping with 1,3-Diketones

A solution of the corresponding enyne (0.10 mmol) in anhydrous CH_2Cl_2 (0.33 mL) was added dropwise over 30 min to a solution of gold catalyst (0.002 mmol) and trapping agent (0.20 mmol) in anhydrous CH_2Cl_2 (0.66 mL) at room temperature. Then, a drop of Et_3N was added, the solvent was evaporated under vacuum, and the crude product was purified by flash column chromatography using different gradients of cyclohexane and ethyl acetate to obtain the pure desired products.

2-((2-Methyl-5-(2-(4-nitrophenoxy)propan-2-yl)cyclopent-1-en-1-yl)methyl)-1,3-diphenylpropane-1,3-dione (14a)

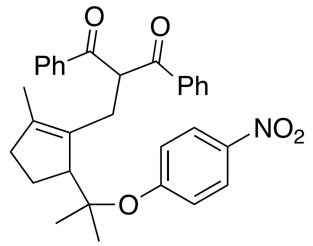

Yellow oil, 71% (optimized conditions, Gold(I) catalyst **G**). ^{1}H NMR (500 MHz, $CDCl_3$) δ 8.01 (d, J = 9.2 Hz, 2H), 7.90 (dd, J = 8.4, 1.2 Hz, 2H), 7.81 (dd, J = 8.4, 1.2 Hz, 2H), 7.56-7.53 (m, 1H), 7.46-7.41 (m, 3H), 7.28-7.23 (m, 2H), 6.95 (d, J = 9.2 Hz, 2H), 5.31 (dd, J = 10.3, 4.1 Hz, 1H), 3.33-3.25 (m, 1H), 3.21 (dd, J = 14.1, 10.3 Hz, 1H), 3.07-2.97 (m, 1H), 2.21-2.09 (m, 1H), 1.90-1.82 (m, 1H), 1.81-1.73 (m, 1H), 1.50-1.44 (m, 1H), 1.42 (s, 3H), 1.41 (s, 3H), 1.25 (s, 3H). ^{13}C NMR (101 MHz, $CDCl_3$) δ 197.0, 196.2, 161.3, 142.1, 140.1, 136.6, 136.2, 133.3, 133.3, 131.2, 128.6 (2C), 128.6 (2C), 128.4 (2C), 128.4 (2C), 125.2 (2C), 121.2 (2C), 87.1, 56.0, 55.7, 36.5, 29.1, 25.7, 25.4, 21.9, 14.3. HRMS-ESI: *m/z* calcd. for $C_{31}H_{30}NO_5$ [M–H]$^+$: 496.2124, found: 496.2116.

2-((5-(2-Hydroxypropan-2-yl)-2-methylcyclopent-1-en-1-yl)methyl)-1,3-diphenylpropane-1,3-dione (14b)

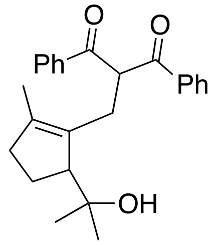

Pale yellow oil, 14%. ^{1}H NMR (500 MHz, $CDCl_3$) δ 8.03-8.00 (m, 2H), 7.96-7.91 (m, 2H), 7.54-7.51 (m, 2H), 7.45-7.39 (m, 4H), 5.58 (dd, *J* = 8.6, 5.4 Hz, 1H), 3.18-3.02 (m, 2H), 2.76 (m, 1H), 2.16-2.08 (m, 1H), 1.87-1.81 (m, 1H), 1.72-1.63 (m, 2H), 1.49 (s, 3H), 1.40-1.36 (m, 1H), 1.23 (s, 3H), 1.06 (s, 3H). ^{13}C NMR (126 MHz, $CDCl_3$) δ 197.1, 197.0, 138.7, 136.6, 136.5, 133.2, 133.2, 128.7, 128.7 (2C), 128.7 (2C), 128.6 (2C), 128.5 (2C), 75.0, 57.3, 55.8, 36.8, 30.7, 29.0, 26.0, 24.4, 14.3. HRMS-ESI: *m/z* calcd. for $C_{25}H_{28}NaO_3$ [M+Na]$^+$: 399.1936, found: 399.1931.

2-((5-(2-Methoxypropan-2-yl)-2-methylcyclopent-1-en-1-yl)methyl)-1,3-diphenylpropane-1,3-dione (14c)

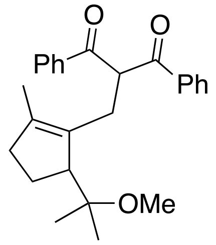

Pale yellow oil, 18%. ^{1}H NMR (500 MHz, $CDCl_3$) δ 8.04-8.01 (m, 2H), 7.95-7.90 (m, 2H), 7.53-7.50 (m, 2H), 7.44-7.39 (m, 4H), 5.62 (dd, *J* = 10.2, 3.7 Hz, 1H), 3.14-3.07 (m, 1H), 3.09 (s, 3H), 2.98-2.89 (m, 1H), 2.88-2.79 (m, 1H), 2.13-2.01 (m, 1H), 1.82-1.74 (m, 1H), 1.70-1.62 (m, 1H), 1.41 (s, 3H), 1.40-1.34 (m, 1H), 1.11 (s, 3H), 0.97 (s, 3H). ^{13}C NMR (126 MHz, $CDCl_3$) δ 197.5, 196.8, 138.5, 136.9, 136.6, 133.1, 133.0, 132.3, 128.7 (2C), 128.6 (2C), 128.47 (2C), 128.45 (2C), 79.1, 56.4, 54.9, 48.6, 36.6, 29.3, 25.3, 23.6, 18.5, 14.2. HRMS-ESI: *m/z* calcd. for $C_{26}H_{30}NaO_3$ [M+Na]$^+$: 413.2092, found: 413.2087.

2-(2-(2-Benzoyl-3-oxo-3-phenylpropyl)-3-methylcyclopent-2-en-1-yl)propan-2-yl acetate (14d)

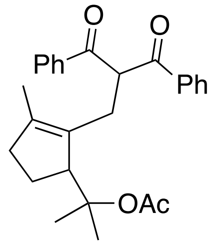

Colorless oil, 14%. ^{1}H NMR (400 MHz, $CDCl_3$) δ 7.98-7.94 (m, 4H), 7.60-7.56 (m, 1H), 7.54-7.46 (m, 3H), 7.43-7.39 (m, 2H), 5.42 (dd, J = 10.6, 3.8 Hz, 1H), 3.46-3.41 (m, 1H), 3.19 (dd, J = 14.2, 10.7 Hz, 1H), 2.92 (d, J = 14.1 Hz, 1H), 2.12-2.04 (m, 1H), 1.77 (s, 3H), 1.69-1.62 (m, 2H), 1.51 (s, 3H), 1.42-1.38 (m, 1H), 1.34 (s, 3H), 1.30 (s, 3H); ^{13}C NMR (126 MHz, $CDCl_3$) δ 197.9, 196.2, 170.7, 139.9, 136.6, 133.3, 130.9, 128.5 (4C), 128.5 (4C), 86.9, 55.0, 54.7, 36.4, 29.0, 25.0, 24.8, 22.7, 21.2, 14.2; HRMS-ESI: m/z: calcd for $C_{27}H_{30}NaO_4$ [$(M+Na)^+$]: 441.2036, found: 441.2041.

2-((5-(2-(4-Methoxyphenoxy)propan-2-yl)-2-methylcyclopent-1-en-1-yl)methyl)-1,3-diphenylpropane-1,3-dione (14e)

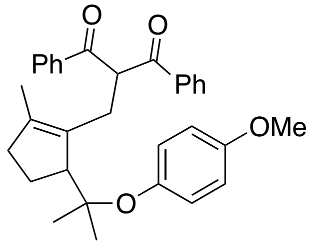

Yellowish oil, 53%. ^{1}H NMR (500 MHz, $CDCl_3$) δ 7.95-7.88 (m, 4H), 7.54-7.51 (m, 1H), 7.42 (t, J = 7.7 Hz, 2H), 7.39-7.35 (m, 1H), 7.12-7.07 (m, 2H), 6.89 (d, J = 9.0 Hz, 2H), 6.78 (d, J = 9.0 Hz, 2H), 5.82 (dd, J = 10.9, 3.8 Hz, 1H), 3.77 (s, 3H), 3.24 (dd, J = 13.7, 11.0 Hz, 1H), 3.11-3.04 (m, 1H), 3.04-2.98 (m, 1H), 2.22-2.13 (m, 1H), 1.95-1.87 (m, 1H), 1.78-1.71 (m, 1H), 1.59 (s, 3H), 1.51-1.44 (m, 1H), 1.20 (s, 3H), 1.04 (s, 3H). ^{13}C NMR (126 MHz, $CDCl_3$) δ 197.2, 196.7, 155.7, 147.9, 139.4, 137.1, 136.0, 133.0, 132.9, 131.8, 128.7 (2C), 128.5 (2C), 128.4 (2C), 128.3 (2C), 125.6 (2C), 113.9 (2C), 84.8, 56.9, 55.6, 54.7, 36.8, 29.8, 26.9, 25.8, 20.5, 14.4. HRMS-ESI: m/z calcd. for $C_{32}H_{34}NaO_4$ $[M+Na]^+$: 505.2354, found: 505.2349.

2-((2-Methyl-5-(2-(4-nitrophenoxy)propan-2-yl)cyclopent-1-en-1-yl)methyl)-1,3-diphenylpropane-1,3-dione (14h)

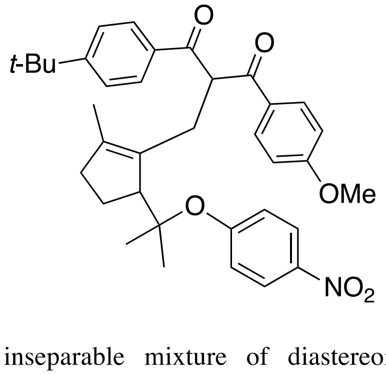

Yellow oil, 82%. Isolated as an inseparable mixture of diastereoisomers (78:22 dr). The following characterization data is reported for the mixture of isomers with the relative integration for each signal. ^{1}H NMR

(500 MHz, $CDCl_3$) δ 8.03-7.99 (m, 2.6H), 7.90 (d, *J* = 8.9 Hz, 2.0H), 7.83 (d, *J* = 8.6 Hz, 0.6H), 7.81 (d, *J* = 8.9 Hz, 0.6H), 7.74 (d, *J* = 8.5 Hz, 2.0H), 7.42 (d, *J* = 8.5 Hz, 0.6H), 7.25 (d, *J* = 8.6 Hz, 2.0H), 6.97-6.93 (m, 2.6H), 6.90 (d, *J* = 8.9 Hz, 2.0H), 6.72 (d, *J* = 8.9 Hz, 0.6H), 5.16 (dd app, *J* = 10.3, 4.0 Hz, 1.3H), 3.86 (s, 3.0H), 3.78 (s, 0.9H), 3.39-3.27 (m, 1.2H), 3.24-3.15 (m, 1.3H), 3.04-2.90 (m, 1.2H), 2.22-2.10 (m, 1.3H), 1.94-1.74 (m, 2.5H), 1.53-1.45 (m, 1.3H), 1.43-1.40 (m, 4.6H), 1.31 (s, 3.0H), 1.28-1.23 (m, 15.2H). ^{13}C NMR (126 MHz, $CDCl_3$) Major diastereoisomer: δ 195.9, 195.4, 163.6, 161.4, 157.1, 142.0, 139.9, 133.5, 131.5, 130.9 (2C), 129.6, 128.4 (2C), 125.5 (2C), 125.2 (2C), 121.0 (2C), 113.8 (2C), 87.1, 55.8, 55.5, 36.6, 35.0, 31.0, 30.9, 29.0, 25.7, 25.4, 22.1, 14.3. HRMS-ESI: m/z calcd. for $C_{36}H_{41}NNaO_6$ $[M+Na]^+$: 606.2832, found: 606.2824.

2-((2-Methyl-5-(2-(4-nitrophenoxy)propan-2-yl)cyclopent-1-en-1-yl)methyl)-1-phenylbutane-1,3-dione (14i)

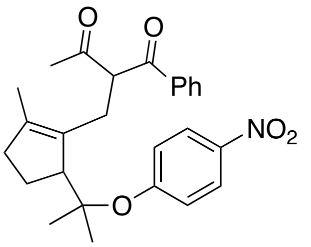

Yellow oil, 68%. Isolated as an inseparable mixture of diastereoisomers (82:18 dr). All signals corresponding to the major and minor diasteroisomer could be identified separately. ^1H NMR (500 MHz, CDCl3) Major diasteroisomer: δ 8.19 (d, J = 9.2 Hz, 2H), 7.88 (dd, *J* = 8.4, 1.2 Hz, 2H), 7.62-7.56 (m, 1H), 7.48 (t, *J* = 7.7 Hz, 2H), 7.08 (d, J = 9.2 Hz, 2H), 4.75 (dd, J = 10.6, 4.0 Hz, 1H), 3.16-3.04 (m, 2H), 2.89-2.80 (m, 1H), 2.20-2.12 (m, 1H), 2.08 (s, 3H), 1.92-1.85 (m, 1H), 1.74-1.67 (m, 1H), 1.52 (s, 3H), 1.47-1.42 (m, 1H), 1.38 (s, 3H), 1.26 (s, 3H). Minor diasteroisomer: δ 7.96 (d, *J* = 9.2 Hz, 2H), 7.79 (dd, *J* = 8.4, 1.2 Hz, 2H), 7.46-7.43 (m, 1H), 7.32-7.28 (m, 2H), 6.87 (d, *J* = 9.2 Hz, 2H), 4.67 (dd, *J* = 10.9, 4.2 Hz, 1H), 3.41-3.33 (m, 1H), 3.21-3.16 (m, 1H), 2.79-2.71 (m, 1H), 2.35-2.28 (m, 1H), 2.25-2.21 (m, 1H), 2.06 (s, 3H), 2.02-1.96 (m, 1H), 1.75 (s, 3H), 1.59-1.54 (m, 1H), 1.42 (s, 3H), 1.23 (s, 3H). ^{13}C NMR (126 MHz, CDCl3) Major diasteroisomer: δ 204.0, 197.5, 161.3, 142.4, 139.9, 137.1, 133.5, 130.9, 128.7 (2C), 128.3 (2C), 125.3 (2C), 121.5 (2C), 87.1, 60.6, 56.7, 36.5, 28.42, 28.36, 25.5, 25.3, 21.3, 14.3. Minor diasteroisomer: δ 204.8, 196.3, 161.2, 142.0, 139.5, 136.2, 133.6, 131.5, 128.6 (2C), 128.4 (2C), 125.2 (2C), 120.9 (2C), 87.3, 61.7, 55.5, 36.9, 28.3, 27.2, 25.6, 25.3, 21.9, 14.5. HRMS-ESI: m/z calcd. for $C_{26}H_{29}NNaO_5$ $[M+Na]^+$: 458.1943, found: 458.1943.

3-((2-Methyl-5-(2-(4-nitrophenoxy)propan-2-yl)cyclopent-1-en-1-yl)methyl)pentane-2,4-dione (14j)

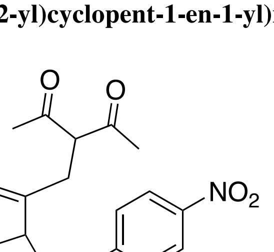

Pale yellow oil, 82%. ^1H NMR (500 MHz, $CDCl_3$) δ 8.17 (d, *J* = 9.3 Hz, 2H), 7.03 (d, *J* = 9.3 Hz, 2H), 4.04 (dd, *J* = 11.0, 4.0 Hz, 1H), 3.23-3.15 (m, 1H), 2.98 (dd, *J* = 14.1, 11.1 Hz, 1H), 2.73-2.64 (m, 1H), 2.36-2.25 (m, 1H), 2.23-2.14 (m, 1H), 2.11 (s, 3H), 2.04 (s, 3H), 2.01-1.93 (m, 1H), 1.72 (s, 3H), 1.61-1.53 (m, 1H), 1.37 (s, 3H), 1.24 (s, 3H). ^{13}C NMR (126 MHz, $CDCl_3$) δ 205.1, 204.2, 161.1, 142.6, 139.5, 131.2, 125.3 (2C), 122.0 (2C),

87.4, 66.1, 56.4, 36.9, 29.13, 29.09, 27.8, 25.5, 25.3, 21.1, 14.5. HRMS-ESI: *m/z* calcd. for $C_{21}H_{27}NNaO_5$ $[M+Na]^+$: 396.1787, found: 396.1791.

6-Methyl-3-((2-methyl-5-(2-(4-nitrophenoxy)propan-2-yl)cyclopent-1-en-1-yl)methyl)heptane-2,4-dione (14k)

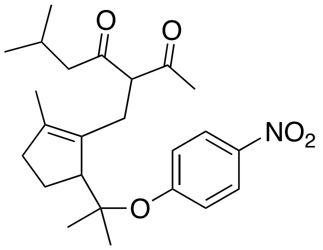

Yellowish oil, 63%. Isolated as an inseparable mixture of diastereoisomers (52:48 dr). The following characterization data is reported for the mixture of isomers with the relative integration for each signal. ^1H NMR (400 MHz, $CDCl_3$) δ 8.17 (d app, J = 9.2 Hz, 2H + 2H), 7.07-7.00 (m, 2H + 2H), 4.03 (dd, J = 11.1, 3.9 Hz, 1H + 1H), 3.24-3.14 (m, 1H + 1H), 3.00 (td app, J = 13.9, 11.1 Hz, 1H + 1H), 2.68-2.57 (m, 1H + 1H), 2.38-2.25 (m, 2H + 2H), 2.22-2.13 (m, 2H + 2H), 2.09 (s, 2.9H), 2.04 (s, 3.0H), 2.03-1.91 (m, 2H + 2H), 1.72 (s, 2.9H), 1.70 (s, 3.0H), 1.60-1.52 (m, 1H + 1H), 1.39 (s, 3H), 1.36 (s, 2.9H), 1.24 (s, 3.0H), 1.22 (s, 2.9H), 0.88-0.84 (m, 3H + 3H), 0.76-0.68 (m, 3H + 3H). ^{13}C NMR (101 MHz, $CDCl_3$) δ 206.3, 206.0, 205.2, 204.1, 161.2, 161.1, 142.8, 142.5, 139.5, 139.3, 131.4, 131.3, 125.3 (2C), 125.2 (2C), 122.4 (2C), 121.7 (2C), 87.5, 87.2, 66.0, 65.7, 56.6, 56.3, 52.1, 51.2, 36.92, 36.85, 28.7, 28.6, 27.9, 27.80, 25.5, 25.4, 25.32, 25.31, 23.9, 23.6, 22.4, 22.31, 22.27, 22.2, 21.14, 21.12, 14.52, 14.50. HRMS-ESI: *m/z* calcd. for $C_{24}H_{33}NNaO_5$ $[M+Na]^+$: 438.2256, found: 438.2255.

Methyl 2-((2-methyl-5-(2-(4-nitrophenoxy)propan-2-yl)cyclopent-1-en-1-yl)methyl)-3-oxobutanoate (14l)

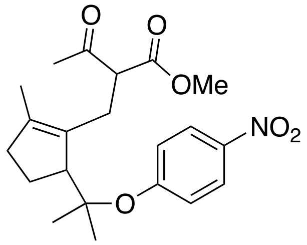

Pale yellow oil, 59%. Isolated as an inseparable mixture of diastereoisomers (56:44 dr). The following characterization data is reported for the mixture of isomers with the relative integration for each signal. ^1H NMR (500 MHz, $CDCl_3$) δ 8.19-8.13 (m, 3.5H), 7.07-7.01 (m, 3.5H), 3.88 (dd, J = 10.9, 4.5 Hz, 0.8H), 3.81 (dd, J = 11.3, 4.1 Hz, 1.0H), 3.67 (s, 3.0H), 3.64 (s, 2.4H), 3.26-3.17 (m, 1.8H), 3.01-2.89 (m, 1.8H), 2.78-2.69 (m, 1.8H), 2.33-2.26 (m, 1.8H), 2.22-2.17 (m, 1.8 H), 2.15 (s, 2.4H), 2.09 (s, 3.0H), 2.02-1.97 (m, 1.8H), 1.70 (s, 2.4H), 1.69 (s, 3.0H), 1.58-1.50 (m, 1.8H), 1.41 (s, 2.4H), 1.37 (s, 3.0H), 1.27 (s, 2.4H), 1.25 (s, 3.0H). ^{13}C NMR (126 MHz, $CDCl_3$) δ 203.7, 202.8, 170.4, 170.3, 161.3, 161.2, 142.6, 142.2, 139.8, 139.7, 131.10, 131.07, 125.3 (2C *dia 2*), 125.2 (2C *dia 1*), 121.8 (2C *dia 2*), 121.2 (2C *dia 1*), 87.3, 87.0, 58.1, 56.9, 56.6, 56.2, 52.3, 52.1, 36.84, 36.84, 29.0, 28.6, 27.8, 27.4, 25.5, 25.41, 25.41, 25.3, 21.10, 21.05, 14.4, 14.3. HRMS-ESI: *m/z* calcd. for $C_{21}H_{27}NNaO_6$ $[M+Na]^+$: 412.1736, found: 412.1734.

Ethyl 2-((2-methyl-5-(2-(4-nitrophenoxy)propan-2-yl)cyclopent-1-en-1-yl)methyl)-3-oxo-3-phenylpropanoate (14m)

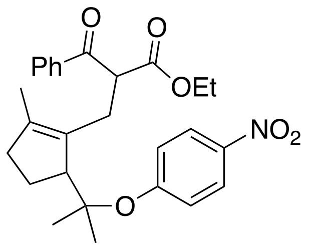

Yellow oil, 66%. Isolated as an inseparable mixture of diastereoisomers (78:22 dr). The following characterization data is reported for the mixture of isomers with the relative integration for each signal. ^{1}H NMR (400 MHz, $CDCl_3$) δ 8.17 (d, J = 9.2 Hz, 2.0H), 7.97 (d, J = 9.2 Hz, 0.5H), 7.90-7.81 (m, 2.5H), 7.60-7.54 (m, 1.0H), 7.50-7.43 (m, 2.2H), 7.35-7.29 (m, 0.5H), 7.09 (d, J = 9.2 Hz, 2.0H), 6.89 (d, J = 9.2 Hz, 0.5H), 4.65 (dd, J = 10.6, 4.4 Hz, 1.0H), 4.49 (dd, J = 11.2, 4.0 Hz, 0.3H), 4.07 (q, J = 7.1 Hz, 2.0H), 4.05-3.98 (m, 0.5H), 3.49-3.42 (m, 0.3H), 3.21-3.11 (m, 1.3H), 3.10-3.04 (m, 1.0H), 2.92-2.82 (m, 1.0H), 2.77-2.69 (m, 0.3H), 2.38-2.26 (m, 0.3H), 2.25-2.12 (m, 1.3H), 2.08-1.98 (m, 0.3H), 1.95-1.85 (m, 1.0H), 1.76-1.67 (m, 1.6H), 1.59 (s, 0.8H), 1.55 (s, 3.0H), 1.48-1.42 (m, 1.0H), 1.44 (s, 0.8H), 1.40 (s, 3.0H), 1.29 (s, 3.0H), 1.26 (s, 0.8H), 1.15-1.07 (m, 3.8H). ^{13}C NMR (101 MHz, $CDCl_3$) δ 196.3, 194.7, 170.1, 169.7, 161.5, 161.3, 142.1, 142.0, 140.1, 139.6, 137.0, 136.0, 133.4, 133.2, 131.4, 130.7, 128.5, 128.5, 128.3, 128.3, 125.3, 125.2, 121.0, 120.8, 87.2, 87.0, 61.3, 61.1, 56.7, 55.5, 53.0, 51.4, 36.9, 36.6, 28.4, 28.0, 25.7, 25.5, 25.4, 25.3, 21.7, 21.3, 14.33, 14.31, 14.0, 13.9. HRMS-ESI: m/z calcd. for $C_{27}H_{31}NNaO_6$ $[M+Na]^+$: 488.2049, found: 488.2050.

Ethyl 3-(furan-2-yl)-2-((2-methyl-5-(2-(4-nitrophenoxy)propan-2-yl)cyclopent-1-en-1-yl)methyl)-3-oxopropanoate (14n)

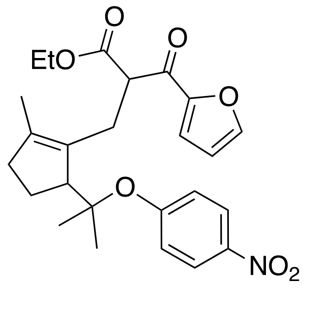

Orange oil, 75%. Isolated as an inseparable mixture of diastereoisomers (92:8 dr). The following characterization data is reported for the major isomer though in the ^{1}H NMR some selected signals of the minor isomer are gathered. ^{1}H NMR (500 MHz, $CDCl_3$) δ 8.17 (d, J = 9.2 Hz, 2H), 7.60 (dd, J = 1.7, 0.7 Hz, 1H), 7.21 (dd, J = 3.6, 0.7 Hz, 1H), 7.09 (d, J = 9.2 Hz, 2H), 6.55 (dd, J = 3.6, 1.7 Hz, 1H), 4.42 (dd, J = 11.2, 4.2 Hz, 1H), 4.08 (qd, J = 7.1, 1.9 Hz, 2H), 3.27-3.21 (m, 1H), 3.16 (dd, J = 13.9, 11.2 Hz, 1H), 2.91-2.84 (m, 1H), 2.24-2.16 (m, 1H), 2.04-1.95 (m, 1H), 1.89-1.80 (m, 1H), 1.53 (s, 3H), 1.52-1.46 (m, 1H), 1.42 (s, 3H), 1.30 (s, 3H), 1.11 (t, J = 7.1 Hz, 3H). [Minor diasteroisomer, selected signals: δ 8.03 (d, J = 9.2 Hz, 2H), 7.45 (dd, J = 1.7, 0.8 Hz, 1H), 6.93 (d, J = 9.2 Hz, 2H), 6.43 (dd, J = 3.6, 1.7 Hz, 1H), 4.24 (dd, J = 11.0, 4.3 Hz, 1H)]. ^{13}C NMR (126 MHz, $CDCl_3$) δ 184.6, 169.7, 161.5, 152.3, 146.6, 142.1, 140.1, 130.7, 125.3 (2C), 121.0 (2C), 117.8, 112.5, 87.0, 61.3, 56.5, 52.1, 36.6, 27.9, 25.5, 25.3, 21.2, 14.2, 14.0. HRMS-ESI: m/z calcd. for $C_{25}H_{29}NNaO_7$ $[M+Na]^+$: 478.1842, found: 478.1843.

3. Gold(I)-catalyzed Cyclization / 1,5-OR Migration / Intermolecular Cyclopropanation

3.1. General Procedure for the Gold(I)-Catalyzed Cyclization

In a 10 mL flask was added the gold catalyst (4.2 mg, 5.49 μmol) and put under vacuum. After 10/15 min the flask was put under argon and CH_2Cl_2 (1 ml) was added, followed by the trapping agent (0.220 mmol) and 1-((3,7-dimethyloct-6-en-1-yn-3-yl)oxy)-4-nitrobenzene (30 mg, 0.110 mmol). The reaction was stirred at the indicated temperature and monitored by TLC (9:1 cyclohexane/diethyl ether). The mixture was diluted with CH_2Cl_2 (2 ml), quenched with QuadraPure MPA resin and filtered through a cotton pipette. The crude was purified firstly by flash column chromatography (95:5 cyclohexane/diethyl ether) then by preparative TLC (98:2 cyclohexane/diethyl ether).

7-(2-Methyl-5-(2-(4-nitrophenoxy)propan-2-yl)cyclopent-1-en-1-yl)bicyclo[4.1.0]heptane (15a)

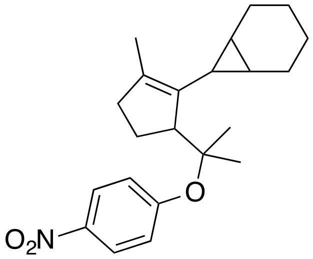

7-(2-Methyl-5-(2-(4-nitrophenoxy)propan-2-yl)cyclopent-1-en-1-yl)bicyclo[4.1.0]heptane (15a): Cyclization performed at 0 ºC for 2h 30 min following the general procedure for *intermolecular cyclopropanation*. Yellow resin, 69% (56:44 dr). ^{1}H NMR (500 MHz, $CDCl_3$) δ 8.23 – 8.13 (m, 3.8H), 7.12 – 7.01 (m, 4H), 3.20 – 3.10 (m, 1H), 3.06 – 2.96 (m, 1H), 2.46 – 2.30 (m, 2H), 2.27 – 2.11 (m, 2H), 2.07 (ddt, *J* = 13.7, 8.3, 1.6 Hz, 1H), 2.00 – 1.78 (m, 9H), 1.77 – 1.74 (m, 6H), 1.53 (s, 3H), 1.51 – 1.45 (m, 4H), 1.39 (s, 3H), 1.35 (s, 3H), 1.32 – 1.09 (m, 10H), 0.95 – 0.85 (m, 3H), 0.84 – 0.75 (m, 2H). The carbon signals arising from the two isomers could be differentiated (labeled as *isomer a* and *isomer b*); however, these could not be assigned definitively to one of them. ^{13}C NMR (126 MHz, $CDCl_3$) δ 162.4 (*isomer a*), 162.3 (*isomer b*) , 142.2 (*isomer a*) , 142.0 (*isomer b*), 141.6 (*isomer a*), 140.0 (*isomer b*), 136.1 (*isomer a*) , 131.6 (*isomer b*), 125.2 (2C *isomer a*), 125.1 (2C *isomer b*), 121.7 (2C *isomer a*) , 121.3 (2C *isomer b*), 87.4 (*isomer a*), 87.1 (*isomer b*), 57.6 (*isomer a*), 57.3 (*isomer b*), 37.4 (*isomer a*), 37.0 (*isomer b*), 25.9 (*isomer a*), 25.4 (*isomer a*), 25.2 (*isomer b*), 25.1 (*isomer b*), 23.8 (*isomer a*), 23.7 (*isomer a* + *isomer b*), 23.4 (*isomer b*), 23.1 (*isomer a*), 22.7 (*isomer a*), 22.2 (*isomer b*), 21.6 (*isomer a*), 21.5 (*isomer b*), 21.48 (*isomer a*), 20.7 (*isomer b*), 19.6 (*isomer b*), 18.5 (i*somer a*) , 16.9 (*isomer b*), 15.5 (*isomer a*), 15.1 (*isomer b*) , 13.5 (*isomer a*), 12.9 (*isomer b*). HRMS-ESI calculated for $C_{22}H_{29}NNaO_3$ $[M+Na]^+$: 378.2040; found: 378.2037

3-(2-Methyl-5-(2-(4-nitrophenoxy)propan-2-yl)cyclopent-1-en-1-yl)tricyclo[3.2.1.0^{2,4}]octane (15b)

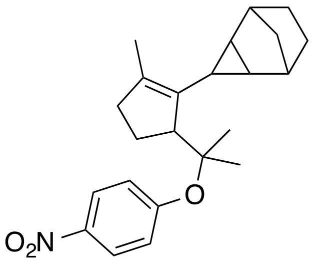

Cyclization performed at 25 ºC for 50 min following the general procedure for *intermolecular cyclopropanation*. Colorless resin, 58% (75:25 dr). ^{1}H NMR (400 MHz, $CDCl_3$) δ 8.24 – 8.12 (m, 2.8H), 7.13 – 7.04 (m, 2.8H), 3.14 – 8.08 (m, 1H), 3.05 (broad d, J = 9.3 Hz, 0.3H), 2.40 – 2.26 (m, 3H), 2.20 – 2.09 (m, 1.5H), 2.02 (broad s, 1H), 1.92 – 1.83 (m, 2.3H), 1.82 – 1.80 (broad s, 1H), 1.78 – 1.73 (broad s, 3.5H), 1.53 (s, 1H), 1.46 (s, 3H), 1.44 – 1.40 (m, 2.6H), 1.39 (s, 3H), 1.26 – 1.13 (m, 4H), 0.98 – 0.92 (m, 1.8H), 0.81 – 0.77 (m, 1H), 0.74 – 0.68 (m, 0.4H), 0.66 – 0.54 (m, 2.5H). ^{13}C NMR (101 MHz, $CDCl_3$) δ 162.5 (*minor*), 162.4 (*major*), 141.8 (minor), 141.7 (*major*), 139.7 (*major*), 138.9 (*minor*), 133.8 (*major*), 133.0 (*minor*), 125.2 (2C *major*), 125.22 (2C *minor*), 120.9 (2C *minor*), 120.8 (2C *major*), 86.8 (*major*), 86.7 (*minor*), 58.2 (*minor*), 57.4 (*major*), 37.5 (m*ajor*), 37.1 (2C *minor*), 36.4 (*minor*), 36.1 (major), 35.6 (*major*), 30.2 (*minor*), 30.0 (*minor*), 29.8 (*minor*), 29.6 (*major*) , 29.4 (*major*), 28.2 (*major*), 25.8 (*minor*), 25.9 (*major*), 25.4 (*minor*), 25.2 (*major*), 25.1 (*major*), 24.2 (*minor*), 24.1 (*minor*), 24.0 (*major*), 22.7 (*major*), 21.2 (*minor*), 17.1 (*minor*), 15.1 (*major*), 14.6 (*minor*), 13.3 (*major*). HRMS-ESI calculated for $C_{25}H_{29}NNaO_3$ $[M+Na]^+$: 390.2040; found: 390.2045

7-(2-Methyl-5-(2-(4-nitrophenoxy)propan-2-yl)cyclopent-1-en-1-yl)-2-oxabicyclo[4.1.0]heptane (15c)

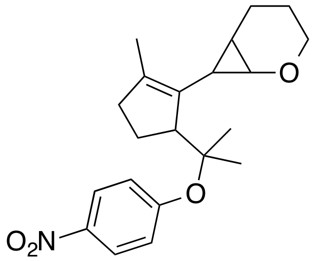

Purified by flash column chromatography (100:9:1 hexane/ethyl acetate/Et_3N). Light yellow oil, 82%. *Major isomer*: ^{1}H NMR (400 MHz, $CDCl_3$, selected signals) δ 8.15-8.11 (m, 2H), 7.12-7.08 (m, 2H), 3.60-3.56 (m, 1H), 3.40 (dd, J = 7.2, 2.6 Hz, 1H), 3.31 (td, J = 11.0, 2.3 Hz, 1H), 3.05 (br d, J = 9.0 Hz, 1H), 2.33-2.27 (m, 1H), 2.15-2.07 (m, 2H), 2.02-1.94 (m, 1H), 1.92-1.81 (m, 2H), 1.69 (s, 3H), 1.53 (s, 3H), 1.40 (s, 3H), 1.03-0.98 (m, 1H); ^{13}C NMR (100 MHz, $CDCl_3$) δ 162.4, 142.0, 141.1, 133.6, 125.3 (2C), 121.2 (2C), 86.7, 65.6, 57.8, 56.9, 37.4, 25.3, 25.1, 24.4, 24.2, 22.8, 21.4, 20.0, 15.0; *minor isomer*: ^{1}H NMR (400 MHz, $CDCl_3$, selected signals) δ 7.08-7.04 (m, 2H), 2.95-2.93 (m, 1H), 1.83 (m, 3H), 1.41 (s, 3H), 1.33 (s, 3H), 0.89-0.84 (m, 1H); ^{13}C NMR (100 MHz, $CDCl_3$) δ 162.3, 142.5, 141.3, 132.7, 125.3 (2C), 122.1 (2C), 87.0, 64.4, 59.7, 57.2, 37.6, 25.3, 25.3, 24.1, 24.1, 22.5, 19.3, 17.6, 15.2; HRMS calcd. for $C_{21}H_{27}NO_4Na$ (M+Na): 380.1838; found: 380.1827.

1-(2-Methyl-5-(2-(4-nitrophenoxy)propan-2-yl)cyclopent-1-en-1-yl)-1,1a,6,6a-tetrahydrocyclopropa[*a*]indene (15d)

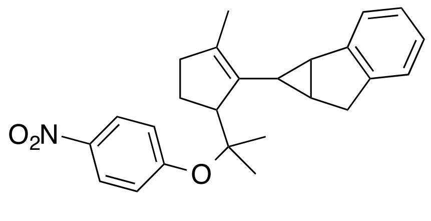

Cyclization performed at 0 ºC for 2h 40 min following the general procedure for *intermolecular cyclopropanation*. Yellow resin, 80% (93:7 dr). ^{1}H NMR (500 MHz, $CDCl_3$) *for the major isomer* δ 8.18 – 8.11 (m, 2H), 7.22 – 7.17 (m, 1H), 7.10 – 7.05 (m, 2H), 7.05 – 6.99 (m, 1H), 6.96 – 6.86 (m, 2H), 3.05 (dd, *J* = 17.0, 7.0 Hz, 1H), 2.66 (d, *J* = 17.0 Hz, 1H), 2.60 (ddd, *J* = 7.8, 6.1, 1.5 Hz, 1H), 2.34 – 2.20 (m, 1H), 2.04 – 1.97 (m, 1H), 1.96 – 1.93 (m, 1H), 1.84 (s, 3H), 1.79 – 1.68 (m, 2H), 1.51 (s, 3H), 1.28 (s, 3H), 1.13 (dq, *J* = 13.4, 9.5 Hz, 1H). ^{13}C NMR (126 MHz, $CDCl_3$) δ 162.4 (*minor*), 162.2 (*major)*, 143.5 (*major*), 143.4 (*minor*), 143.1 (*major*), 143.0 (*minor*), 143.0 (*major*), 142.3 (*minor*), 142.0 (*major*), 129.6 (*major*), 125.7 (*major*), 125.6 (*major*), 125.3 (2C *minor*), 125.2 (*minor*), 125.1 (2C *major*), 125.0 (C *minor*), 124.5 (*major*), 124.4 (*major*), 123.5 (*minor*), 121.4 (2C *major*), 120.9 (2C *minor*), 119.1 (*minor*), 87.1 (*major*), 87.07 (*minor*), 56.7 (*minor*), 54.5 (*major*), 37.1 (*major*), 36.6 (*minor*), 32.9 (*minor*) , 32.2 (*major*), 31.2 (*minor*), 29.6 (*major*), 25.5 (*major*), 25.2 (*minor*), 25.0 (*major*), 24.6(*minor*), 24.2 (*major*), 24.1 (*minor*), 23.0 (*major*), 22.7 (*minor*), 22.4 (*major*), 20.7 (*minor*), 15.9 (*minor*), 15.0 (*major*). HRMS-ESI calculated for $C_{25}H_{27}NNaO_3$ $[M+Na]^+$: 412.1883; found: 412.1888.

1-(2-Methyl-5-(2-(4-nitrophenoxy)propan-2-yl)cyclopent-1-en-1-yl)-1a,6b-dihydro-1*H*-cyclopropa[*b*]benzofuran (15e)

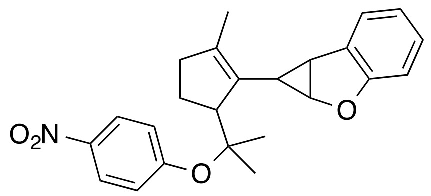

Cyclization performed at 24 ºC for 20 min following the general procedure for *intermolecular cyclopropanation*. Yellow oil, 62% (97:3 dr). ^{1}H NMR (500 MHz, $CDCl_3$) δ 8.17 – 8.04 (m, 2H), 7.09 (dd, *J* = 7.3, 1.4 Hz, 1H), 6.97 (td, *J* = 7.8, 1.4 Hz, 1H), 6.94 – 6.90 (m, 2H), 6.75 (td, *J* = 7.5, 1.0 Hz, 1H), 6.59 (dd, *J* = 8.0, 0.8 Hz, 1H), 4.86 (t, *J* = 5.4 Hz, 1H), 2.52 (dd, *J* = 9.0, 5.3 Hz, 1H), 2.40 – 2.34 (m, 1H), 2.17 – 2.06 (m, 1H), 1.83 – 1.76 (m, 1H), 1.74 (s, 3H), 1.54 – 1.42 (m, 2H), 1.33 (s, 3H), 1.27 (s, 3H), 1.05 (dtd, *J* = 13.5, 9.8, 8.3 Hz, 1H). ^{13}C NMR (125 MHz, $CDCl_3$) δ 162.3, 159.7, 145.3, 141.8, 127.1, 127.0, 125.5, 125.2 (2C), 124.7, 120.8 (2C), 120.2, 109.0, 86.9, 65.3, 55.0, 37.0, 25.3, 24.8, 24.7, 24.5, 15.9, 15.4. HRMS-ESI calculated for $C_{24}H_{25}NNaO_4$ $[M+Na]^+$: 414.1676; found: 414.1669.

5-Bromo-1-(2-methyl-5-(2-(4-nitrophenoxy)propan-2-yl)cyclopent-1-en-1-yl)-1a,6b-dihydro-1H-cyclopropa[b]benzofuran (15f)

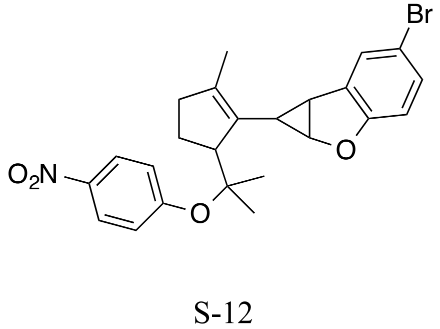

Cyclization performed at 24 ºC for 1 h following the general procedure for *intermolecular cyclopropanation*. Yellow oil, 69% (98:2 dr). ^{1}H NMR (500 MHz, $CDCl_3$) δ 8.27 – 8.23 (m, 2H), 7.28 (d, *J* = 2.1 Hz, 1H), 7.17 (dd, *J* = 8.5, 2.1 Hz, 1H), 7.05 – 6.99 (m, 2H), 6.58 (d, *J* = 8.5 Hz, 1H), 4.99 (t, *J* = 5.3 Hz, 1H), 2.63 (dd, *J* = 9.1, 5.3 Hz, 1H), 2.55 – 2.45 (m, 1H), 2.30 – 2.17 (m, 1H), 1.98 – 1.86 (m, 1H), 1.82 (s, 3H), 1.65 – 1.56 (m, 2H), 1.43 (s, 3H), 1.37 (s, 3H), 1.27 – 1.21 (m, 1H). ^{13}C NMR (125 MHz, $CDCl_3$) δ 162.1, 158.8, 145.8, 142.0, 129.9, 129.6, 127.6, 125.4 (2C), 125.0, 120.85 (2C), 111.9, 110.5, 86.7, 66.2, 55.0, 37.0, 25.3, 24.8 , 24.7, 24.7, 16.0, 15.4. HRMS-ESI calculated for $C_{24}H_{24}BrNNaO_4$ $[M+Na]^+$: 492.0781; found: 492.0785.

1-(2-Methyl-5-(2-(4-nitrophenoxy)propan-2-yl)cyclopent-1-en-1-yl)-1a,6b-dihydro-1H-benzo[b]cyclopropa[d]thiophene (15g)

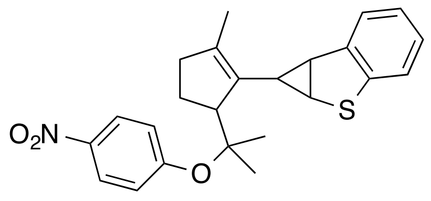

Cyclization performed at 26 ºC for 40 min following the general procedure for *intermolecular cyclopropanation*. White crystal, 33% (93:7 dr). mp: 155.5-155.6 °C. ^{1}H NMR (500 MHz, $CDCl_3$) δ 8.22 – 8.17 (m, 2H), 7.35 – 7.31 (m, 1H), 7.13 – 7.08 (m, 2H), 7.07 – 7.00 (m, 2H), 6.99 – 6.91 (m, 1H), 4.17 – 4.08 (m, 1H), 3.35 (dd, *J* = 8.7, 7.3 Hz, 1H), 2.95 (t, *J* = 7.4 Hz, 1H), 2.20 – 2.07 (m, 1H), 1.91 – 1.81 (m, 1H), 1.77 – 1.63 (m, 2H), 1.49 (s, 3H), 1.46 (s, 3H), 1.45 (s, 3H), 1.33 – 1.29 (m, 1H). ^{13}C NMR (125 MHz, $CDCl_3$) δ 162.5 (Cq), 143.8 (Cq), 143.7, 141.6, 137.5, 128.1, 126.59, 126.56, 125.3 (2C), 123.2, 120.5, 120.3 (2C), 87.2, 53.6, 36.9, 35.5, 28.9, 25.3, 24.7, 24.6, 17.0, 16.0. HRMS-ESI calculated for $C_{24}H_{25}NNaO_3S$ $[M+Na]^+$: 430.1447, found: 430.1454.

4. A Second Generation Formal Synthesis of (+)-Schisanwilsonene A

((((*S*)-2-((*R*)-2-Methyl-5-(2-(4-nitrophenoxy)propan-2-yl)cyclopent-1-en-1-yl)cyclopropane-1,1-diyl)bis(methylene))bis(oxy))bis(*tert*-butyldimethylsilane) ((±)-6a)

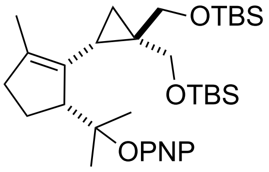

A solution of the protected alcohol **(±)-1a** (500 mg, 1.83 mmol) in anhydrous CH_2Cl_2 (6.1 mL) was added dropwise over 30 min to a solution of gold catalyst IMesAutmbn (34.0 mg, 0.0365 mmol) and 2,2,3,3,9,9,10,10-octamethyl-6-methylene-4,8-dioxa-3,9-disilaundecane (1.16 g, 3.66 mmol) in anhydrous CH_2Cl_2 (12.2 mL) at room temperature. A few drops of NEt_3 were added and the solution was filtered through a short pad of SiO_2 (washed with 4:1 hexanes/ethyl acetate) then concentrated. Purification over silica gel (hexanes to 50:1 hexanes/ethyl acetate) afforded a 1:1.25 mixture (ratio determined by ^{1}H-NMR) of the cyclopropane **(±)-6a** and bis-TBS protected alkene as a colourless oil (total 1.37 g, 79% calculated yield, 62 wt% purity). ^{1}H NMR (400 MHz, $CDCl_3$) δ 8.15-8.11 (m, 2H), 7.06-7.02 (m, 2H), 3.66 (dd, *J* = 16.2, 10.1 Hz, 2H), 3.26 (dd, *J* = 26.1, 10.1 Hz, 2H), 3.24 (br. s, 1H), 2.40-2.28 (m, 1H), 2.20-2.11 (m, 1H), 1.91-1.81 (m, 2H), 1.75 (s, 3H), 1.47-1.43 (m, 1H), 1.43 (s, 3H), 1.35 (s, 3H), 0.91 (s, 9H), 0.91-0.84 (m, 1H), 0.84 (s, 9H), 0.41 (dd, *J* = 5.9, 4.8 Hz, 1H), 0.05 (s, 3H), 0.04 (s, 3H), -0.03 (s, 3H), -0.04 (s, 3H); ^{13}C NMR (100 MHz, $CDCl_3$) δ 162.5, 142.0, 140.9, 133.1, 125.3 (2C), 121.4 (2C), 87.2, 65.6, 63.3, 58.9, 38.0, 30.3, 28.2, 27.1, 26.1(3C), 26.1 (3C), 25.8, 25.6, 25.4, 24.3, 21.8, 18.6, 18.5, 15.4, 15.0, -5.3 (4C); HRMS-ESI: *m/z*: calcd for $C_{32}H_{55}O_5NSi_2Na$ [(M+Na)$^+$]: 612.3516, found: 612.3517.

((*S*)-2-((*R*)-2-Methyl-5-(2-(4-nitrophenoxy)propan-2-yl)cyclopent-1-en-1-yl)cyclopropane-1,1-diyl)dimethanol ((±)-6c)

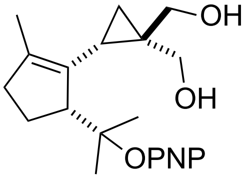

TBAF (8.48 mL, 1.0M, 8.48 mmol) was added dropwise to a solution of cyclopropane **(±)-6a** (1 g, 1.695 mmol) in anhydrous THF (17 mL, 0.10 M) at room temperature. The resulting mixture was stirred for 1 h, then quenched with H_2O and extracted with EtOAc. The combined organic layers were washed with brine, dried over anhydrous $MgSO_4$, filtered and concentrated. Purification over silica gel (1:1 to 1:2 hexanes/EtOAc) afforded the diol **(±)-6c** (545 mg, 89%) as a white solid. ^{1}H NMR (400 MHz, $CDCl_3$) δ 8.17 (d, *J* = 9.1 Hz, 2H), 7.09 (d, *J* = 9.1 Hz, 2H), 3.70 (d, *J* = 11.1 Hz, 1H), 3.55 (d, *J* = 11.2 Hz, 2H), 3.38 (d, *J* = 11.1 Hz, 1H), 3.13 (d, *J* = 9.4 Hz, 1H), 2.37-2.27 (m, 1H), 2.24-2.18 (m, 1H), 2.00-1.93 (m, 1H), 1.79 (s, 3H), 1.69-1.63 (m, 1H), 1.59-1.55 (m, 1H), 1.32 (s, 3H), 1.28 (s, 3H), 0.99 (dd, *J* = 8.4, 4.9 Hz, 1H), 0.67 (dd, *J* = 5.8, 5.4 Hz, 1H); ^{13}C NMR (126 MHz, $CDCl_3$) δ 13C NMR (126 MHz, CDCl3) δ 160.7, 141.9, 132.3, 125.3 (2C), 123.6 (2C), 88.7, 69.9, 65.3, 59.3, 37.7, 28.5, 25.8, 25.6, 23.2, 22.2, 16.4, 15.3; HRMS-ESI: *m/z*: calcd for $C_{20}H_{27}O_5NNa$ [(M+Na)$^+$]: 384.1773, found: 384.1787.

((1*R*,3a*S*)-3a-Methyl-1-(2-(4-nitrophenoxy)propan-2-yl)-1,2,3,3a,4,7-hexahydroazulen-6-yl)methyl acetate ((±)-16b)

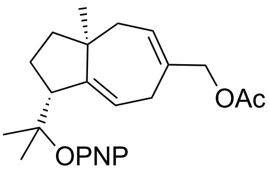

This compound was obtained from diol **(±)-6c** in the following three step sequence (51% yield, over 3 steps).

Step1

((1*R*,2*S*)-1-(Hydroxymethyl)-2-((*R*)-2-methyl-5-(2-(4-nitrophenoxy)propan-2-yl)cyclopent-1-en-1-yl)cyclopropyl)methyl acetate

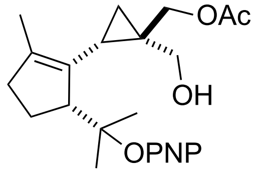

Ac_2O (0.07 mL, 1.0 M solution in CH_2Cl_2, 0.74 mmol) was added dropwise over 1 h to a solution of DMAP (4.3 mg, 0.035 mmol), anhydrous pyridine (0.12 mL, 1.4 mmol) and the diol **(±)-6c** (253 mg, 0.7 mmol) at 0 ºC. The resulting mixture was subsequently stirred for 1 h at 0 ºC, then quenched with aqueous HCl (0.5 M). The aqueous were extracted with EtOAc and the combined organic layers were washed with aqueous HCl (0.5M), water and brine, dried over anhydrous $MgSO_4$, filtered and concentrated. Purification over silica (10:1 to 3:1 cyclohexanes/EtOAc) afforded monoacetate (203 g, 72%) as a colourless oil. The undesired monoacetate and bis-acetate were combined in order to be submitted to a recycling procedure. ^{1}H NMR (400 MHz, $CDCl_3$) δ 8.18-8.14 (m, 2H), 7.11-7.07 (m, 2H), 4.23 (d, *J* = 11.6 Hz, 1H), 3.60 (br. d, *J* = 11.2 Hz, 1H), 3.57 (d, *J* = 11.6 Hz, 1H), 3.28 (d, *J* = 11.2 Hz, 2H), 2.66 (bs, 1H), 2.38-2.26 (m, 1H), 2.26-2.16 (m, 1H), 2.02 (s, 3H), 1.93 (dtd, *J* = 13.7, 9.6, 6.3 Hz, 1H), 1.77 (s, 3H), 1.77-1.69 (m, 1H), 1.62-1.55 (m, 1H), 1.36 (s, 3H), 1.33 (s, 3H), 1.05 (dd, *J* = 8.4, 5.0 Hz, 1H), 0.86 (tdd, *J* = 6.4, 5.3, 2.4 Hz, 1H); ^{13}C NMR (126 MHz, $CDCl_3$) δ 171.8, 161.4, 143.0, 131.7, 125.4 (2C), 122.4 (2C), 87.6, 69.1, 63.1, 58.2, 37.7, 26.3, 25.6, 25.1, 23.6, 23.3, 21.1, 16.6, 15.5; HRMS-ESI: *m*/*z*: calcd for $C_{22}H_{29}O_6NNa$ [(M+Na)$^+$]: 426.1888, found: 426.1893.

Step2

((1*S*,2*S*)-1-Formyl-2-((*R*)-2-methyl-5-(2-(4-nitrophenoxy)propan-2-yl)cyclopent-1-en-1-yl)cyclopropyl)methyl acetate

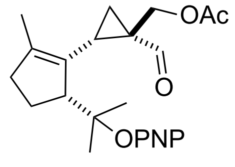

$NaHCO_3$ (429 mg, 5.11 mmol) and Dess-Martin periodinane (650 mg, 1.53 mmol) were added to a solution of monoacetate (412 mg, 1.02 mmol) in CH_2Cl_2 (20 mL, 0.05 M) at room temperature. The resulting mixture was stirred for 1 h, then quenched with a 1:1 mixture of saturated aqueous $NaHCO_3$ and saturated aqueous Na_2SO_3. The aqueous were extracted with EtOAc twice and the combined organic layers were washed with brine, dried

over anhydrous $MgSO_4$, filtered and concentrated. The crude product was used in the following step.

Step 3

((1*R*,3a*S*)-3a-Methyl-1-(2-(4-nitrophenoxy)propan-2-yl)-1,2,3,3a,4,7-hexahydroazulen-6-yl)methyl acetate ((±)-16b)

*n*BuLi (0.15 mL, 1.5 M, 1.72 mmol) was added dropwise to a suspension of PPh_3CH_3Br (658 mg, 1.84 mmol) in anhydrous THF (18.0 mL) at -20 ºC. The resulting yellowish solution was stirred for 10 min at -20 ºC, whereupon a solution of the aldehyde xx (492.8 mg, 1.23 mmol) was added dropwise. After 10 min at -20 ºC, the reaction mixture was allowed to warm to room temperature and stirred overnight. The reaction was quenched with saturated aqueous NH_4Cl, extracted with Et_2O, and the combined organic layers were washed with brine, dried over anhydrous $MgSO_4$, filtered and concentrated. Purification over silica gel (20:1 to 10:1 hexanes/EtOAc) afforded the diene **(±)-16b** (343 mg, 70%) as a colourless oil. The compound was stored in a glove box to avoid oxidation in the presence of air (storage in a vial under inert atmosphere proved to be insufficient). ^{1}H NMR (400 MHz, $CDCl_3$) δ 8.16 (d, *J* = 9.2 Hz, 2H), 7.07 (d, *J* = 9.2 Hz, 2H), 5.83 (ddd, *J* = 6.4, 4.0, 2.3 Hz, 1H), 5.80-5.78 (m, 1H), 4.47 (d, *J* = 11.9 Hz, 1H), 4.42 (d, *J* = 12.0 Hz, 1H), 3.18-3.13 (m, 1H), 3.00-2.86 (m, 2H), 2.35 (d, *J* = 14.4 Hz, 1H), 2.13 (dd, *J* = 15.6, 8.3 Hz, 1H), 2.07 (s, 3H), 1.82-1.73 (m, 1H), 1.63-1.45 (m, 3H), 1.40 (s, 3H), 1.37 (s, 3H), 1.11 (s, 3H); ^{13}C NMR (126 MHz, $CDCl_3$) δ 171.1, 161.8, 150.0, 142.6, 132.9, 127.8, 125.4 (2C), 122.1 (2C), 119.9, 85.8, 70.9, 54.7, 46.2, 41.4, 40.8, 31.3, 26.9, 26.3, 24.0, 23.1, 21.2; HRMS-ESI: *m*/*z*: calcd for $C_{23}H_{29}O_5NNa$ [$(M+Na)^+$]: 422.1923, found: 422.1943.

2-(6-(Hydroxymethyl)-3a-methyl-1,2,3,3a,4,7-hexahydroazulen-1-yl)propan-2-ol ((±)-16a)

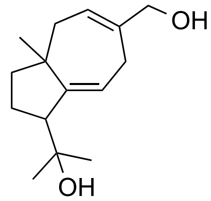

This compound was prepared from **(±)-16b** in a two step sequence (76%, over 2 steps).

Step1

(1-(2-(4-Aminophenoxy)propan-2-yl)-3a-methyl-1,2,3,3a,4,7-hexahydroazulen-6-yl)methanol (S-(±)-16a)

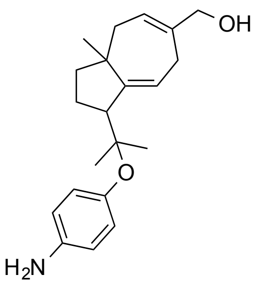

To a suspension of copper(II) acetylacetonate (24.3 mg, 0,093 mmol) and the nitro-compound **(±)-16b** (185.4 mg, 0,46 mmol) in ethanol (4.6 mL) was added sodium borohydride (52.7 mg, 1.39 mmol) at 0ºC and the mixture was stirred overnight at room temperature. Water was then added and the mixture was filtered through a short pad of Celite. The reaction was extracted twice with EtOAc, and dried over $MgSO_4$ before removing the solvent under reduced pressure. Purification over silica gel (cyclohexane / EtOAc, 2 / 1 to 1 / 1) afforded the

product as a pale yellow oil (129.5 mg, 0.40 mmol, 85%). ^{1}H NMR (400MHz, $CDCl_3$) δ 6.83-6.77 (m, 2H), 6.61-6.55 (m, 2H), 6.10-6.07 (m, 1H), 5.72 (ddd, J = 8.1, 3.1, 1.7 Hz, 1H), 3.98 (s, 2H), 3.09-2.89 (m, 3H), 2.37-2.31 (m, 1H), 2.11 (ddd, J = 15.6, 8.0, 1.5 Hz, 1H), 1.70 (tdd, J = 13.1, 5.3, 3.3 Hz, 1H), 1.56-1.47 (m, 2H), 1.46-1.40 (m, 1H), 1.20 (s, 3H), 1.15 (s, 3H), 1.09 (s, 3H); ^{13}C NMR (100MHz, $CDCl_3$) δ 150.4, 147.0, 142.3, 137.7, 125.7 (2C), 124.2, 119.9, 115.6 (2C), 82.8, 69.7, 54.4, 46.1, 41.6, 40.8, 31.0, 27.1, 26.4, 24.0, 22.3; HRMS-ESI calcd for $C_{21}H_{30}NO_2$ [$(M+H)^+$]: 328.2277; found: 328.2285.

Note: the isolation of this intermediate is not necessary. Crude can be used directly to the following deprotection step.

Step2

2-(6-(Hydroxymethyl)-3a-methyl-1,2,3,3a,4,7-hexahydroazulen-1-yl)propan-2-ol ((±)-16a)

A solution of $(NH_4)_2Ce(NO_3)_6$ (CAN, 415.0 mg, 0.76 mmol) in water (3.8 mL) was added dropwise to a solution of **(S-(±)-16a)** (82.9 mg, 0.25 mmol) in CH_3CN (11.4 mL) at 0ºC. The mixture was stirred for 15 mins when water was added and the mixture was extracted with EtOAc and washed with 10 % aqueous $NaHCO_3$. The aqueous layer was re-extracted with EtOAc and the organic layers were combined, washed successively with 10 % $NaHSO_3$, 10 % $NaHCO_3$ and brine. The extracts were then dried over Na_2SO_4, filtered and evaporated to dryness. The crude product was then purified by column chromatography (cyclohexane / EtOAc, 1 / 1) to afford the diol **((±)-16a** (54.0 mg, 0.23 mmol, 90%) as an orange solid. mp 94-97 ºC ^{1}H NMR (400MHz, $CDCl_3$) δ 5.74 (dtd, J = 12.3, 4.1, 3.3, 1.7 Hz, 2H), 3.98 (s, 2H), 2.94 (dd, J = 5.3, 2.5 Hz, 2H), 2.67 (ddq, J = 10.7, 7.9, 2.4 Hz, 1H), 2.32 (dd, J = 16.0, 3.5 Hz, 1H), 2.07 (dd, J = 15.3, 8.1 Hz, 1H), 1.78 - 1.71 (m, 1H), 1.59 (s, 1H), 1.56 - 1.50 (m, 2H), 1.46 - 1.40 (m, 2H), 1.24 (s, 3H), 1.17 (s, 3H), 1.09 (s, 3H); ^{13}C NMR (100MHz, $CDCl_3$) δ 151.0, 138.0, 124.6, 120.2, 73.4, 69.3, 57.0, 45.8, 43.6, 41.9, 40.4, 30.6, 27.0, 26.3, 23.4. HRMS-ESI: *m/z*: calcd for $C_{15}H_{24}O_2Na$ [$(M+Na)^+$]: 259.1674, found: 259.1674.

(*R*)-3,7-Dimethyloct-6-en-1-yn-3-ol (1b)

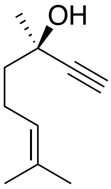

Step i:

A round-bottomed single-necked flask was charged with Molecular Sieves (same grams than SM) before equipping with a Septum and Ar atmosphere. Then, dry CH_2Cl_2 (43.2 ml) and (2*S*,3*S*)-diethyl 2,3-dihydroxysuccinate (2.93 ml, 17.12 mmol) were added. After cooling down to -10 ºC, tetraisopropoxytitanium (4.72 ml, 15.56 mmol) was added. This mixture was stirred during 5 min prior addition of TBHP (7.07 ml, 38.9 mmol). The mixture was stirred at the same temperature for 30 min. Then, the system was cooled down to -25 ºC and a mixture of (*E*)-3,7-dimethylocta-2,6-dien-1-ol (4.57 ml, 25.9 mmol) in dry CH_2Cl_2 (8.64 ml) was added with the aid of an auto-pump (30 min). The reaction temperature was checked not to rise above -20 ºC (actually it was always between -30 and -25 ºC). The reaction was monitored by TLC and typically requires 5-6 h.

Once the reaction is complete, the reaction mixture was poured into an aqueous solution of NaOH and NaCl (around 10 g each) in water (100 mL) while stirring vigorously. The solution was stirred at room temperature for 15 min and passed through a Celite cake (5 cm diameter, 4 cm high) and always rinsed with CH_2Cl_2. A 2 layer

system was obtained it was extracted with CH_2Cl_2 (3 × 30 mL). Removal of volatiles under vacuum afforded the reaction crude which is pure enough for the next step.

Note: Following this work-up provide crudes pure enough for the next step skipping column chromatography.

Step ii:

A round-bottomed single-necked flask was charged with ((2*R*,3*R*)-3-methyl-3-(4-methylpent-3-en-1-yl)oxiran-2-yl)methanol (10,8 g, 50,7 mmol), triphenylphosphane (15,97 g, 60,9 mmol) and $NaHCO_3$ (0,853 g, 10,15 mmol) and Ar atmosphere was generated. Carbon tetrachloride (103 ml, 1066 mmol) was added and the reaction was refluxed typically for 6 h. After completion, solvent was removed under vacuum and the residue was purified by flash column chromatography (7:1 cyclohexane/ethyl acetate, R_f = 0.65).

Step iii:

A round-bottomed single-necked flask was charged with (2*R*,3*S*)-3-(chloromethyl)-2-methyl-2-(4-methylpent-3-en-1-yl)oxirane (7,00 g, 37,1 mmol) and Ar atmosphere was generated. Dry THF (106 ml) was added and the system was cooled down to -40 ºC. Butyllithium (48,4 ml, 111 mmol) was added dropwise over a period of 20 min (with the aid of an automatic pump) and the reaction was stirred for an extra hour at the same temperature. After completion (checked by TLC), the reaction was quenched by adding NH_4Cl (sat, aq.) and extracted with Et_2O (3 × 40 mL). Organics were removed under vacuum and the residue was purified by flash column chromatography (7:1 cyclohexane/ethyl acetate) to afford **1b** as a colorless oil (60%, 89%ee, over 3 steps).

(*R*)-1-((3,7-Dimethyloct-6-en-1-yn-3-yl)oxy)-4-nitrobenzene (1a)

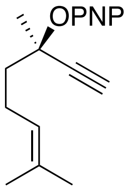

A single-necked round-bottomed flask was charged with 1-fluoro-4-nitrobenzene (100 mg, 0.709 mmol), 3,7-dimethyloct-6-en-1-yn-3-ol (119 mg, 0.780 mmol), and 18-crown-6 (94 mg, 0.354 mmol). The flask was equipped with a septum, Ar atmosphere was generated and dry THF (4.7 mL) was added. The system was cooled down to 0 ºC (ice-bath). KHMDS 0.5 M in toluene (1559 μl, 0.780 mmol) solution was added dropwise (with the aid of an auto-pump, 25 min) to the reaction mixture. After that, the cooling bath was removed and the mixture was stirred at 26 ºC over a period of 22 h. Then, the reaction mixture was quenched by the addition of sat'd aq. NH_4Cl and diluted with Et_2O. The organic layer was kept, and the aqueous layer was extracted again with Et_2O (3 × 10 mL). The combined organic layers were dried over Na_2SO_4. Removal of solvents under vacuum led to the reaction crude, which was purified by flash column chromatography (cyclohexane/ethyl acetate, 33 / 1 to 20 / 1) to afford the protected alcohol as a yellow oil (74%). *Characterization data matches with the data described before.*

Notes: *Scaling up the reaction does not seem to lead to a detrimental in the yield. KHMDS 1 M in THF can also be used, though amount of solvent should be adjusted to maintain final concentration. The color immediately after the addition was deep green/black. After 1h 30' at 26 C it turned to deep blue. After 20 h the solution was deep orange-yellow.*

((((*S*)-2-((*S*)-2-Methyl-5-(2-(4-nitrophenoxy)propan-2-yl)cyclopent-1-en-1-yl)cyclopropane-1,1-diyl)bis(methylene))bis(oxy))bis(*tert*-butyldimethylsilane) (6c)

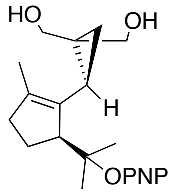

Prepared according to the previously described procedure for **((±)-6a)** using enantioenriched **(*R*)-1-((3,7-Dimethyloct-6-en-1-yn-3-yl)oxy)-4-nitrobenzene (1a)**. Purification over silica gel (1:1 to 1:2 hexanes/EtOAc) afforded the diol **6c** (68%, 2 steps) as a white solid. *Characterization data matches with the data described before*.

HPLC analysis

1-((3,7-Dimethyloct-6-en-1-yn-3-yl)oxy)-4-nitrobenzene ((±)-1a)

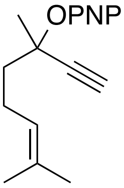

Data File C:\HPCHEM\1\DATA\OSCAR\OS-PNP_RAC-IA.D
Sample Name: OS-PNP_RAC

===

Acq. Operator : OSCAR
Acq. Instrument : HPLC1100 Location : Vial 91
Injection Date : 9/16/2014 4:15:12 PM
Inj Volume : 1 µl
Acq. Method : C:\HPCHEM\1\DATA\KATYA\IMMA.M
Last changed : 9/16/2014 3:47:23 PM by OSCAR
(modified after loading)
Analysis Method : C:\HPCHEM\1\DATA\KATYA\IMMA.M
Last changed : 9/16/2014 3:45:43 PM by OSCAR
(modified after loading)
Method Info : STANDARD FLAVANONE IB

Sample Info : IA
97:3 HEX:IPA
1ml/min

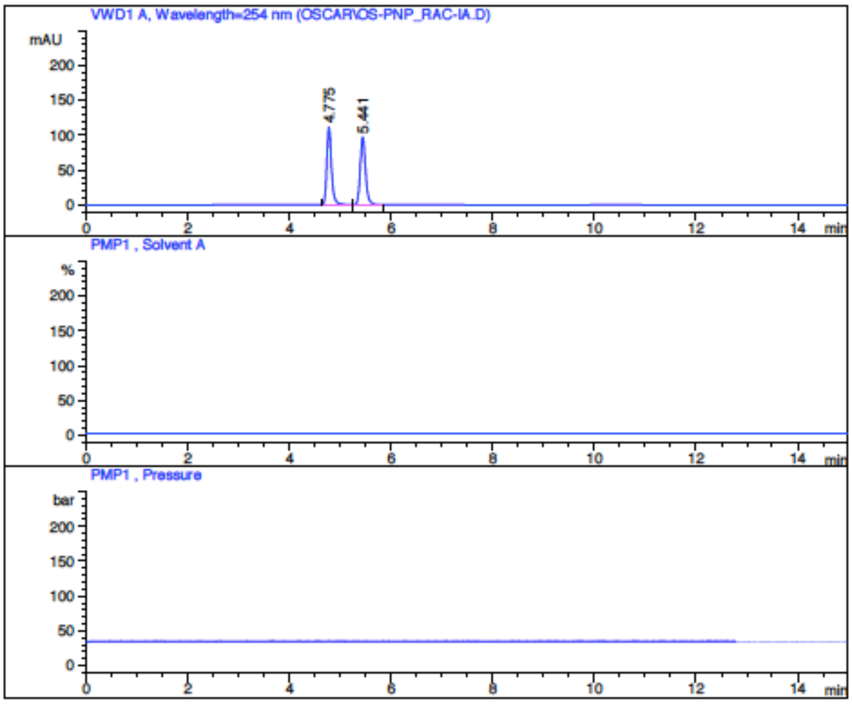

(*R*)-1-((3,7-Dimethyloct-6-en-1-yn-3-yl)oxy)-4-nitrobenzene (1a)

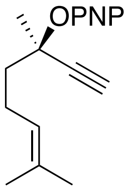


```
Data File C:\HPCHEM\1\DATA\OSCAR\OSCAR 2014-10-03 15-27-45\OS-321.D
Sample Name: OS-321

    =====================================================================
    Acq. Operator   : OSCAR                              Seq. Line :   2
    Acq. Instrument : HPLC1100                            Location : Vial 92
    Injection Date  : 10/3/2014 3:44:43 PM                     Inj :   1
                                                        Inj Volume : 1 µl
    Acq. Method     : C:\HPCHEM\1\DATA\OSCAR\OSCAR 2014-10-03 15-27-45\OS-PNP.M
    Last changed    : 10/3/2014 1:13:44 PM by laura
    Analysis Method : C:\HPCHEM\1\DATA\OSCAR\OSCAR 2014-10-03 15-27-45\OS-321.D\DA.M (OS-PNP.M)
    Last changed    : 10/3/2014 4:26:42 PM by OSCAR
                      (modified after loading)
    Sample Info     : ChiralPack IA
                      97:3 HEX:IPA
                      1.0 ml/miN
```

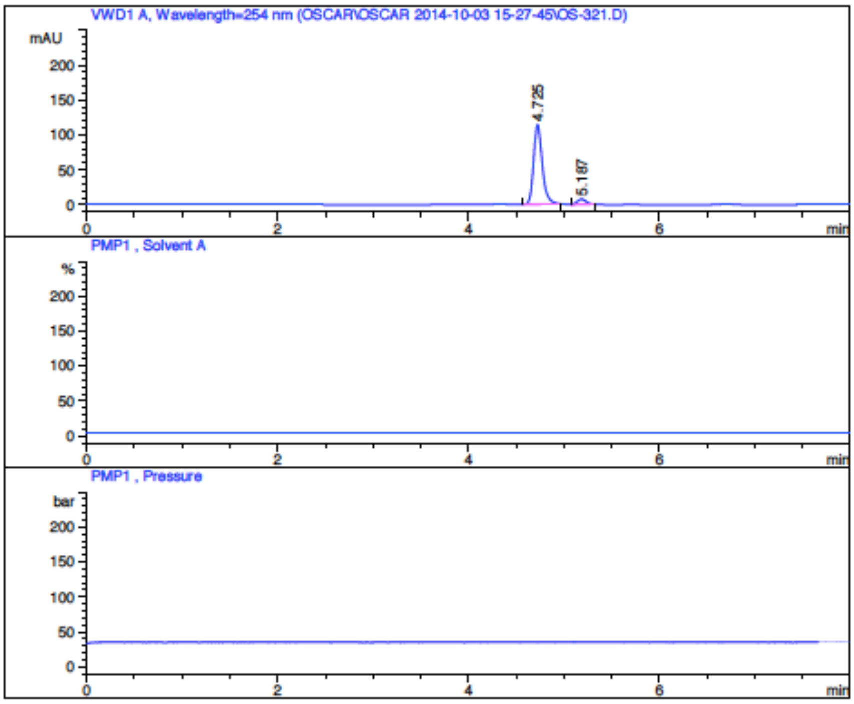

((*S*)-2-((*R*)-2-Methyl-5-(2-(4-nitrophenoxy)propan-2-yl)cyclopent-1-en-1-yl)cyclopropane-1,1-diyl)dimethanol ((±)-6c)

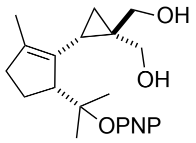


```
Data File C:\HPCHEM\2\DATA\OSCAR\OS-426 RAC.D
Sample Name: OS-426 rac
    =====================================================================
    Acq. Operator   : Oscar
    Acq. Instrument : AG1200HPLC                    Location : Vial 91
    Injection Date  : 1/23/2015 10:42:04 AM
                                                  Inj Volume : 5 µl
    Acq. Method     : C:\HPCHEM\2\METHODS\LAURA.M
    Last changed    : 1/23/2015 11:02:43 AM by Oscar
                      (modified after loading)
    Analysis Method : C:\HPCHEM\2\METHODS\LAURA.M
    Last changed    : 1/23/2015 11:08:09 AM by Oscar
                      (modified after loading)
    Sample Info     : CHIRALPACK IC
                      80:20 Hex:IPA
                      1 ml/min
                      Sample in Hexane

    =====================================================================
```

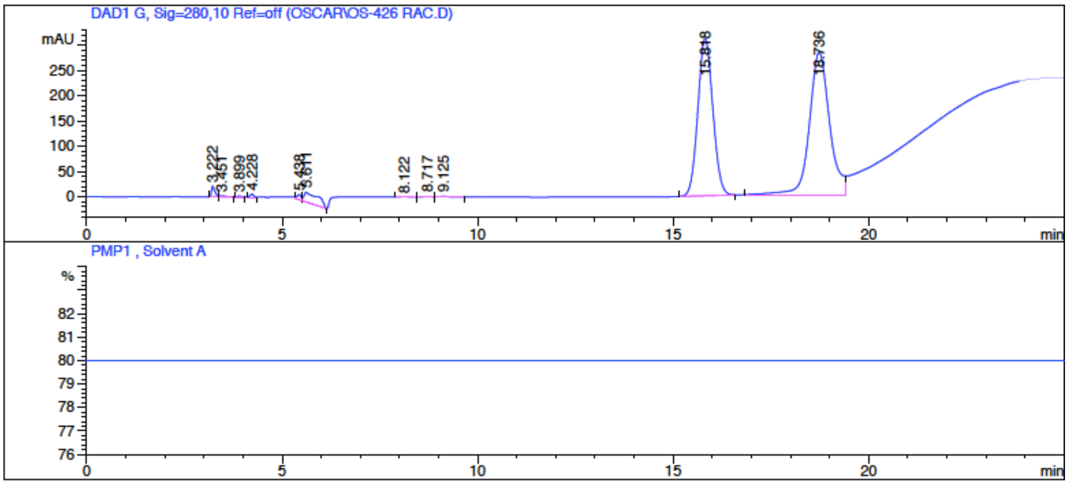

((((*S*)-2-((*S*)-2-Methyl-5-(2-(4-nitrophenoxy)propan-2-yl)cyclopent-1-en-1-yl)cyclopropane-1,1-diyl)bis(methylene))bis(oxy))bis(*tert*-butyldimethylsilane) (6c)

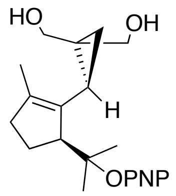


```
Data File C:\HPCHEM\2\DATA\OSCAR\OS-430.D
Sample Name: OS-430
    =====================================================================
    Acq. Operator    : Oscar
    Acq. Instrument  : AG1200HPLC                    Location : Vial 81
    Injection Date   : 1/23/2015 12:02:26 PM
                                                   Inj Volume : 5 µl
    Acq. Method      : C:\HPCHEM\2\METHODS\LAURA.M
    Last changed     : 1/23/2015 11:47:30 AM by Oscar
                       (modified after loading)
    Analysis Method  : C:\HPCHEM\2\METHODS\LAURA.M
    Last changed     : 1/23/2015 12:28:27 PM by Oscar
                       (modified after loading)
    Sample Info      : CHIRALPACK IC
                       80:20 Hex:IPA
                       1 ml/min
                       Sample in Hexane

    =====================================================================
```

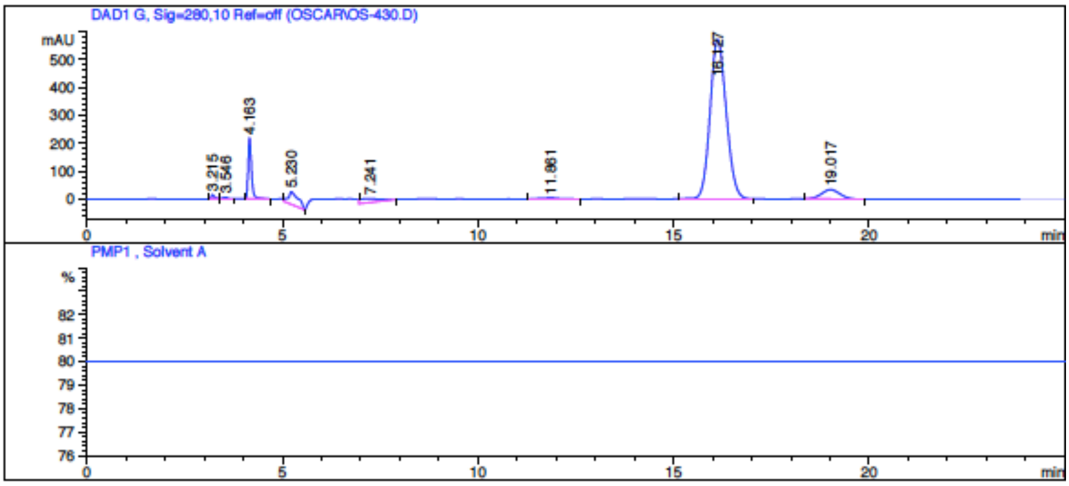

5.1. General procedure for the synthesis of propargylamines

A solution of **1d** (195 mg, 1.00 mmol), the corresponding N-nucleophile and CuCl (5-10 mol%) in anhydrous THF (2.0 mL) was heated at the indicated temperature in a sealed tube for the indicated time. After cooling the mixture was treated with Et_3N (same number of equivalents as the N-nucleophile), concentrated over Florisil and purified by flash chromatography[1].

N-(3,7-Dimethyloct-6-en-1-yn-3-yl)-4-methoxyaniline (17a)

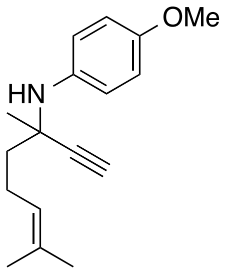

Pale yellow oil (hexane/ethyl acetate/NEt_3 = 100 : 9 : 1), 68%. ^{1}H NMR (400 MHz, C_6D_6) δ 7.33-7.30 (m, 2H), 6.84-6.81 (m, 2H), 5.19 (triple septuplet, *J* = 7.2, 1.4 Hz, 1H), 3.92 (AB system, *J* = 12.0 Hz, 1H), 3.87 (AB system, *J* = 12.0 Hz, 1H), 3.33 (s, 3H), 2.31-2.26 (m, 2H), 2.11 (s, 1H), 1.71-1.66 (m, 2H), 1.65-1.64 (m, 3H), 1.57 (s, 3H), 1.31 (s, 3H), 1.09 (br s, 1H); ^{13}C NMR (100 MHz, C_6D_6) δ 159.3, 133.6, 131.5, 129.9, 124.8, 114.2, 88.6, 71.2, 54.8, 53.7, 48.3, 42.3, 27.2, 25.8, 23.6, 17.7; HRMS calcd. for $C_{18}H_{26}NO$ $[M+H]^+$: 272.2014, found: 272.2017.

***N*-(3,7-Dimethyloct-6-en-1-yn-3-yl)-4-nitroaniline (17b)**

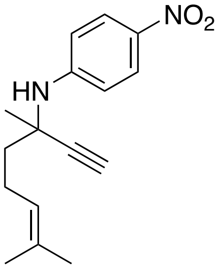

Orange oil after vacuum drying overnight at 50 ºC (hexane/ethyl acetate/Et_3N = 100 : 12 : 1 to 100 : 20 : 1), 83%. ^{1}H NMR (400 MHz, $CDCl_3$) δ 8.11-8.07 (m, 2H), 6.87-6.83 (m, 2H), 5.17-5.12 (m, 1H), 4.61 (br s, 1H), 2.50 (s, 1H), 2.33-2.14 (m, 2H), 1.95-1.82 (m, 2H), 1.71 (m, 3H), 1.63 (s, 3H), 1.61 (s, 3H); ^{13}C NMR (100 MHz, $CDCl_3$) δ 151.2, 138.7, 133.4, 126.0, 123.1, 113.7, 84.9, 73.2, 51.8, 42.0, 27.8, 25.8, 23.3, 17.9; HRMS calcd. for $C_{16}H_{20}N_2O_2Na$ $[M+Na]^+$: 295.1422, found: 295.1415.

5.2. General Procedure for the Gold(I)-catalyzed Cyclization of Propargylamines

The solid catalyst was added over a solution of the substrate (0.1M) in dry CH_2Cl_2 and the mixture was stirred at the indicated temperature for the indicated time. After quenching with Et_3N (>1 equiv.), the mixture was concentrated over Florisil® and purified by flash colum chromatography.

6-Methoxy-2-methyl-2-(4-methylpent-3-en-1-yl)-1,2-dihydroquinoline (18)

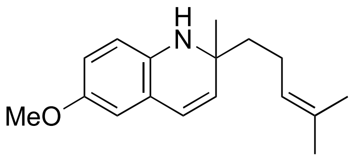

Brownish yellow oil (hexane/ethyl acetate/Et_3N = 100 : 5 : 1), 66%. ^1H NMR (400 MHz, C_6D_6) δ 6.67 (dd, *J* = 8.5, 2.8 Hz, 1H), 6.55 (d, *J* = 2.8 Hz, 1H), 6.18 (d, *J* = 9.8 Hz, 1H), 6.15 (d, *J* = 8.5 Hz, 1H), 5.23 (d, *J* = 9.7 Hz, 1H), 5.19-5.15 (m, 1H), 3.40 (s, 3H), 2.91 (br s, 1H), 2.24-2.14 (m, 1H), 2.11-2.02 (m, 1H), 1.67 (s, 3H), 1.53 (s, 3H), 1.40 (ddd, *J* = 13.6, 11.2, 5.5 Hz, 1H), 1.30 (ddd, *J* = 13.6, 11.3, 5.3 Hz, 1H), 1.06 (s, 3H); ^{13}C NMR (100 MHz, C_6D_6) δ 152.5, 138.3, 131.1, 130.6, 125.3, 125.1, 121.0, 115.1, 113. 6, 112.6, 55.5, 55.1, 44.0, 29.7, 25.9, 23.9, 17.7; HRMS calcd. for $C_{17}H_{24}NO$ (M+H): 258.1858, found: 258.1850.

1,3,3-Trimethyl-7-methylene-2-(4-nitrophenyl)-2-azabicyclo[2.2.1]heptane (19)

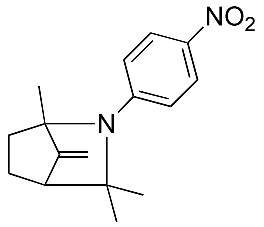

Orange solid (hexane/ethyl acetate/Et_3N = 100 : 5 : 1). ^1H NMR (400 MHz, C_6D_6) δ 8.03-7.99 (m, 2H), 6.38-6.34 (m, 2H), 4.63 (s, 1H), 4.56 (s, 1H), 1.85 (ddd, *J* = 12.3, 9.4, 4.5 Hz, 1H), 1.79 (d, *J* = 4.4 Hz, 1H), 1.51 (ddd, *J* = 12.8, 9.4, 4.4 Hz, 1H), 1.33-1.25 (m, 1H), 1.17 (s, 3H), 1.12 (s, 3H), 1.07 (td, J = 12.1, 4.4 Hz, 1H), 0.89 (s, 3H); ^{13}C NMR (125 MHz, C_6D_6) δ 156.5, 150.9, 138.6, 124.9, 117.0, 97.3, 65.6, 65.3, 53.3, 30.5, 29.4, 23.3, 22.4, 15.8; HRMS calcd for $C_{16}H_{20}N_2NaO_2$ $[M+Na]^+$: 295.1422, found: 295.1426. X-Ray quality crystals were obtained by slow evaporation of a pentane solution of the product.

6. Endo-type Skeletal Rearrangement instead of OR migration

(*E*)-3-Methyl-7-phenylhept-6-en-1-yn-3-ol (26a)

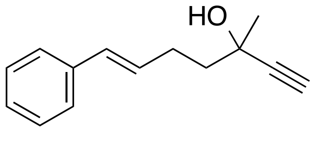

Ethynylmagnesium bromide (0.5 M in THF, 3.3 mL, 1.5 equiv) was added dropwise to a solution of (*E*)-6-phenylhex-5-en-2-one[2] (191 mg, 1 equiv) in THF (0.1 M) at -10 ºC. The mixture was allowed to warm up to 23 ºC and stirred overnight. The reaction was quenched with saturated NH_4Cl and ethyl acetate was added. Phases were separated and the aqueous layer was extracted with ethyl acetate (3 times). The combined organic extracts were dried over Na_2SO_4, filtered and concentrated under reduced pressure. The residue was purified by flash column chromatography (9:1 cyclohexane/ethyl acetate) to afford **26a** as a pale yellow oil (150 mg, 68% yield). ^1H NMR (500 MHz, $CDCl_3$) δ 7.36 – 7.27 (m, 4H), 7.22 – 7.18 (m, 1H), 6.46 (d, *J* = 15.8 Hz, 1H), 6.26 (dt, *J* = 15.8, 6.9 Hz, 1H), 2.55 – 2.41 (m, 3H), 1.89 – 1.83 (m, 2H), 1.55 (s, 3H). ^{13}C NMR (125 MHz, $CDCl_3$) δ 137.6, 130.4, 129.9, 128.5 (2C), 127.0, 126.0 (2C), 87.4, 71.8, 68.0, 42.8, 30.0, 28.4. HRMS-ESI: *m/z* calculated for $C_{14}H_{16}ONa$ $[M+Na]^+$: 223.1099, found: 223.1094.

(*E*)-Trimethyl((3-methyl-7-phenylhept-6-en-1-yn-3-yl)oxy)silane (26b)

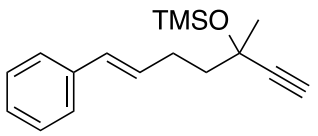

TMSOTf (54.0 μL, 1.50 equiv) was added dropwise to a solution of **26a** (34.0 mg, 1 equiv) and Et_3N (50.0 μL, 1.80 equiv) in CH_2Cl_2 (0.1 M) at 0 ºC. The resulting mixture was allowed to warm up to 23 ºC and stirred overnight. The crude material was purified by flash column chromatography (pure hexane, 1% Et_3N) to afford **26b** as a pale yellow oil (30 mg, 65%). ^{1}H NMR (400 MHz, $CDCl_3$) δ 7.26 (dd, *J* = 6.6, 5.0 Hz, 2H), 6.86 – 6.81 (m, 2H), 6.36 (d, *J* = 15.8 Hz, 1H), 6.11 (dt, *J* = 15.8, 6.8 Hz, 1H), 3.80 (s, 3H), 2.47 (s, 1H), 2.38 (dt, *J* = 10.9, 5.8 Hz, 2H), 1.83 – 1.73 (m, 2H), 1.50 (s, 3H), 0.20 (d, *J* = 3.0 Hz, 9H). ^{13}C NMR (100 MHz, $CDCl_3$) δ 137.9, 130.6, 129.8, 128.5 (2C), 126.8, 125.9 (2C), 87.6, 72.6, 69.1, 44.5, 31.2, 28.3, 1.9 (3C). HRMS-ESI: *m/z* calculated for $C_{17}H_{25}OSi$ $[M+H]^+$: 273.1675, found: 273.1682.

Ethyl (*E*)-2-acetyl-5-(4-nitrophenyl)pent-4-enoate

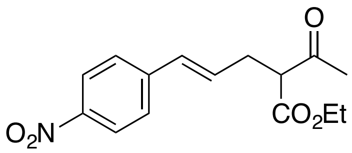

Ethyl 3-oxobutanoate (0.33 mL, 1 equiv) was added dropwise to a suspension of NaH (60 wt% in mineral oil, 109 mg, 1.05 equiv) in THF (0.05 M) at 0 ºC. The resulting mixture was allowed to warm up to 23 ºC and stirred for 30 min. Then a solution of (*E*)-1-(3-bromoprop-1-en-1-yl)-4-nitrobenzene[3] (625 mg, 1.00 equiv) in THF (0.1 M) was added dropwise and the reaction mixture was stirred at 23 ºC overnight. The reaction was quenched with saturated NH_4Cl. Ethyl acetate was added and phases were separated. The aqueous layer was extracted with ethyl acetate (3 times). The combined organic extracts were washed with brine, dried over Na_2SO_4, filtered and concentrated under reduced pressure. The crude material was purified by flash chromatography over silica gel (9:1 hexane/ethyl acetate) to afford the desired product as a yellow oil (675 mg, 90% yield). ^{1}H NMR (400 MHz, $CDCl_3$) δ 8.20 – 8.10 (m, 2H), 7.49 – 7.40 (m, 2H), 6.53 (d, *J* = 15.9 Hz, 1H), 6.41 – 6.26 (m, 1H), 4.22 (q, *J* = 7.1 Hz, 2H), 3.61 (t, *J* = 7.2 Hz, 1H), 2.79 (td, *J* = 7.2, 1.3 Hz, 2H), 2.27 (s, 3H), 1.27 (t, *J* = 7.1 Hz, 3H). ^{13}C NMR (100 MHz, $CDCl_3$) δ 201.8, 168.9, 146.8, 143.3, 131.1, 130.9, 126.7 (2C), 124.0 (2C), 61.7, 59.1, 31.4, 29.24, 14.1. HRMS-ESI: *m/z* calculated for

(*E*)-6-(4-Nitrophenyl)hex-5-en-2-one

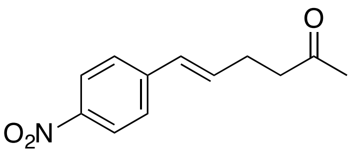

To a solution of ethyl (*E*)-2-acetyl-5-(4-nitrophenyl)pent-4-enoate (670 mg, 1 equiv) in THF (0.2 M) was added KOH and water. The reaction mixture was stirred at 60 ºC for 4-5 h (TLC monitoring) and then cooled down to 23 ºC. Ethyl acetate was added and phases were separated. The aqueous layer was extracted with ethyl acetate (3 times) and the combined organic extracts were washed with brine, dried over Na_2SO_4, filtered and concentrated under reduced pressure. The crude material was purified by flash column chromatography (9:1 hexane/ethyl acetate) to afford the desired product as a yellow oil (407 mg, 81% yield). ^{1}H NMR (400 MHz, $CDCl_3$) δ 8.17 – 8.11 (m, 2H), 7.47 – 7.39 (m, 2H), 6.51 – 6.35 (m, 2H), 2.66 (dd, *J* = 10.8, 3.7 Hz, 2H), 2.53 (dt, *J* = 8.2, 3.9 Hz,

2H), 2.18 (s, 3H). ^{13}C NMR (100 MHz, $CDCl_3$) δ 207.4, 146.6, 143.9, 134.2, 129.1, 126.5 (2C), 124.0 (2C), 42.6, 30.0, 27.1. HRMS-ESI: *m/z* calculated for $C_{12}H_{13}NO_3Na$ $[M+Na]^+$: 242.0793, found: 242.0797.

(*E*)-3-Methyl-7-(4-nitrophenyl)hept-6-en-1-yn-3-ol (26c)

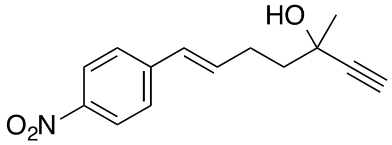

Ethynylmagnesium bromide (0.5 M in THF, 2.5 mL, 1.5 equiv) was added dropwise to a solution of (*E*)-6-(4-nitrophenyl)hex-5-en-2-one (185 mg, 1 equiv) in THF (0.1 M) at -10 ºC. The mixture was allowed to warm up to 23 ºC and stirred overnight. The reaction was quenched with saturated NH_4Cl and ethyl acetate was added. Phases were separated and the aqueous layer was extracted with ethyl acetate (3 times). The combined organic extracts were dried over Na_2SO_4, filtered and concentrated under reduced pressure. The residue was purified by flash column chromatography (8:2 cyclohexane/ethyl acetate) to afford **26c** as a pale yellow oil (182 mg, 88% yield). ^{1}H NMR (400 MHz, $CDCl_3$) δ 8.16 (dd, *J* = 9.1, 2.0 Hz, 2H), 7.48 – 7.41 (m, 2H), 6.55 – 6.42 (m, 2H), 2.61 – 2.45 (m, 3H), 1.90 – 1.78 (m, 2H), 1.56 (s, 3H). ^{13}C NMR (100 MHz, $CDCl_3$) δ 146.6, 144.1, 135.5, 128.6, 126.4, 124.0, 87.1, 72.0, 67.8, 42.3, 30.1, 28.5. HRMS-ESI: *m/z* calculated for $C_{14}H_{16}NO_3$ $[M+H]^+$: 246.1130, found: 246.1140.

(*E*)-Trimethyl((3-methyl-7-(4-nitrophenyl)hept-6-en-1-yn-3-yl)oxy)silane (26d)

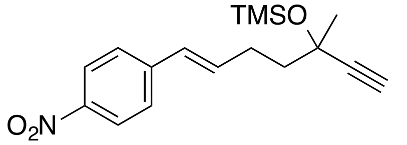

TMSOTf (59.0 μL, 1.50 equiv) was added dropwise to a solution of **26c** (53 mg, 1 equiv) and Et_3N (54.0 μL, 1.80 equiv) in CH_2Cl_2 (0.1 M) at 0 ºC. The resulting mixture was allowed to warm up to 23 ºC and stirred overnight. The crude material was purified by flash column chromatography (pure hexane, 1% Et_3N) to afford **26d** as a pale yellow oil (60 mg, 88%). ^{1}H NMR (500 MHz, $CDCl_3$) δ 8.15 (d, *J* = 7.2 Hz, 2H), 7.45 (m, *J* = 7.2 Hz, 2H), 6.50 – 6.46 (m, 2H), 2.51 – 2.44 (m, 2H), 2.48 (s, 1H), 1.85 – 1.76 (m, 3H), 1.51 (s, 3H), 0.20 (m, 9H). ^{13}C NMR (125 MHz, $CDCl_3$) δ 146.5, 144.4, 136.2, 128.1, 126.3, 124.0, 100.0 (2C), 87.6, 72.8, 68.9, 44.1, 28.6, 1.9 (3C). HRMS-ESI: *m/z* calculated for $C_{17}H_{24}NO_3Si$ $[M+H]^+$: 318.1525, found: 318.1530.

(*E*)-6-(2-nitrophenyl)hex-5-en-2-one

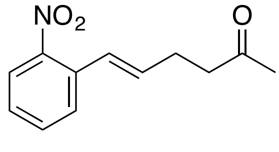

To a suspension of NaH (60 wt% in mineral oil, 1.44 g, 35.9 mmol, 1.1 eq) in THF (70 mL) was added methyl 3-oxobutanoate (4.55 g, 4.2 mL, 39.2 mmol, 1.2 equiv). The resulting mixture was and stirred for 30 min. Then, a solution of (E)-1-(3-bromoprop-1-en-1-yl)-2-nitrobenzene[4] (7.91 g, 32.7 mmol) in THF (30 mL) was added added dropwise. The resulting mixture was stirred at 23 ºC overnight. Then, water (100 mL) and KOH (9.2 g, 163 mmol, 5 equiv) were added and the resulting mixture stirred at 60 ºC until full conversion was achieved. The reaction mixture was cooled to 23 ºC and diluted with EtOAc. Phases were separated and the aqueous layer was extracted with EtOAc (3 times). The organic combined extracts were washed with dried over Na_2SO_4, filtered and concentrated under reduced pressure. The residue (6.5 g) was purified by flash column chromatography (9:1 cyclohexane/ethyl acetate) to afford the desired product as a yellowish oil (3.65 g, 51%, 2 steps).

^{1}H NMR (500 MHz, $CDCl_3$) δ 7.91 – 7.85 (m, 1H), 7.57 – 7.50 (m, 2H), 7.35 (ddd, *J* = 8.5, 7.0, 1.8 Hz, 1H), 6.86 (dt, *J* = 15.7, 1.6 Hz, 1H), 6.22 (dt, *J* = 15.7, 6.7 Hz, 1H), 2.66 (t, *J* = 7.2 Hz, 2H), 2.56 – 2.50 (m, 2H), 2.19 (s, 3H). ^{13}C NMR (125 MHz, $CDCl_3$) δ 207.8, 147.8, 134.7, 133.2, 133.1, 128.7, 127.8, 126.0, 124.6, 42.8, 30.1, 27.2.

(*E*)-3-Methyl-7-(2-nitrophenyl)hept-6-en-1-yn-3-ol (26e)

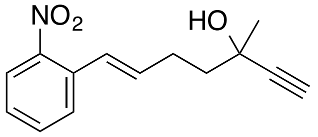

Ethynylmagnesium bromide (0.5 M in THF, 30 mL, 1.7 equiv) was added dropwise to a solution of (*E*)-6-(2-nitrophenyl)hex-5-en-2-one (1.94 g, 1 equiv) in THF (0.1 M) at -10 ºC. The mixture was allowed to warm up to 23 ºC and stirred overnight. The reaction was quenched with saturated NH_4Cl and ethyl acetate was added. Phases were separated and the aqueous layer was extracted with ethyl acetate (3 times). The combined organic extracts were dried over Na_2SO_4, filtered and concentrated under reduced pressure. The residue was purified by flash column chromatography (9:1 cyclohexane/ethyl acetate) to afford **26e** as a pale yellow oil (1.79 g, 82% yield). ^{1}H NMR (400 MHz, $CDCl_3$) δ 7.88 (dd, *J* = 1.2, 8.3 Hz, 1H), 7.58 – 7.50 (m, 2H), 7.36 – 7.32 (m, 1H), 6.90 (d, *J* = 16.1 Hz, 1H), 6.27 (dt, *J* = 6.8, 15.5 Hz, 1H), 2.60 – 2.50 (m, 2H), 2.50 (s, 1H), 2.07 (s, 1H), 1.93 – 1.83 (m, 2H), 1.55 (s, 3H). ^{13}C NMR (100 MHz, $CDCl_3$) δ 147.8, 135.9, 133.3, 133.0, 128.6, 127.7, 125.6, 124.5, 87.3, 72.1, 68.0, 42.5, 30.2, 28.7.

Ethyl (*E*)-2-acetyl-5-(4-methoxyphenyl)pent-4-enoate

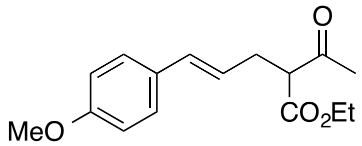

Ethyl 3-oxobutanoate (0.39 mL, xx mmol) was added dropwise to a suspension of NaH (60 wt% in mineral oil, 78 mg, 1.05 equiv) in THF (0.05 M) at 0 ºC. The resulting mixture was allowed to warm up to 23 ºC and stirred for 30 min. Then a solution of (*E*)-1-(3-bromoprop-1-en-1-yl)-4-methoxybenzene[5] (311 mg, 1.00 equiv) in THF (0.1 M) was added dropwise and the reaction mixture was stirred at 23 ºC overnight. The reaction was quenched with saturated NH_4Cl. Ethyl acetate was added and phases were separated. The aqueous layer was extracted with ethyl acetate (3 times). The combined organic extracts were washed with brine, dried over Na_2SO_4, filtered and concentrated under reduced pressure. The crude material was purified by flash chromatography over silica gel (9:1 hexane/ethyl acetate) to afford the desired product as a yellow oil (349 mg, 82% yield). ^{1}H NMR (400 MHz, $CDCl_3$) δ 7.24 (dt, *J* = 9.6, 2.5 Hz, 2H), 6.85 – 6.79 (m, 2H), 6.39 (d, *J* = 15.8 Hz, 1H), 5.96 (dt, *J* = 15.7, 7.2 Hz, 1H), 4.23 – 4.16 (m, 2H), 3.79 (s, 3H), 3.57 (t, *J* = 7.4 Hz, 1H), 2.72 (t, *J* = 7.1 Hz, 2H), 2.25 (s, 3H), 1.26 (dd, *J* = 8.6, 5.6 Hz, 3H). ^{13}C NMR (100 MHz, $CDCl_3$) δ 202.6, 169.3, 159.1, 132.1, 129.8, 127.3 (2C), 123.4, 113.9 (2C), 61.5, 59.8, 55.3, 31.6, 29.2, 14.1. HRMS-ESI: *m/z* calculated for $C_{16}H_{20}O_4Na$ $[M+Na]^+$: 299.1259, found: 299.1252.

(*E*)-6-(4-Methoxyphenyl)hex-5-en-2-one

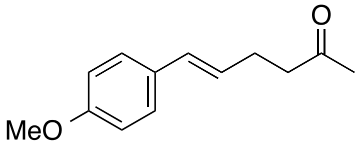

To a solution of ethyl *(E)*-2-acetyl-5-(4-methoxyphenyl)pent-4-enoate (1.05 g, 1 equiv) in THF (0.2 M) was added KOH and water. The reaction mixture was stirred at 60 ºC for 4-5 h (TLC monitoring) and then cooled down to 23 ºC. Ethyl acetate was added and phases were separated. The aqueous layer was extracted with ethyl acetate (3 times) and the combined organic extracts were washed with brine, dried over Na_2SO_4, filtered and concentrated under reduced pressure. The crude material was purified by flash column chromatography (10:1 hexane/ethyl acetate to afford the desired ketone as a yellow oil (731 mg, 94% yield). ^{1}H NMR (400 MHz, $CDCl_3$) δ 7.28 – 7.23 (m, 2H), 6.86 – 6.80 (m, 2H), 6.35 (d, *J* = 15.8 Hz, 1H), 6.04 (dt, *J* = 15.8, 6.8 Hz, 1H), 3.79 (d, *J* = 5.9 Hz, 3H), 2.60 (t, *J* = 7.3 Hz, 2H), 2.48 – 2.42 (m, 2H), 2.16 (s, 3H). ^{13}C NMR (100 MHz, $CDCl_3$) δ 208.2, 158.8, 130.2, 130.1, 127.1 (2C), 126.6, 113.9 (2C), 55.3, 43.4, 30.0, 27.1. HRMS-ESI: *m/z* calculated for $C_{13}H_{16}O_2Na$ [M+Na]$^+$: 227.1048, found: 227.1050.

(*E*)-7-(4-Methoxyphenyl)-3-methylhept-6-en-1-yn-3-ol (26f)

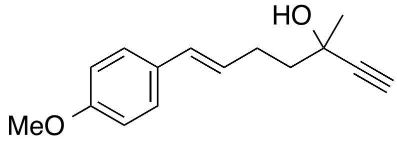

Ethynylmagnesium bromide (0.5 M in THF, 5.10 mL, 1.5 equiv) was added dropwise to a solution of (*E*)-6-(4-methoxyphenyl)hex-5-en-2-one (372 mg, 1 equiv) in THF (0.1 M) at -10 ºC. The mixture was allowed to warm up to 23 ºC and stirred overnight. The reaction was quenched with saturated NH_4Cl and ethyl acetate was added. Phases were separated and the aqueous layer was extracted with ethyl acetate (3 times). The combined organic extracts were dried over Na_2SO_4, filtered and concentrated under reduced pressure. The residue was purified by flash column chromatography (8:2 cyclohexane/ethyl acetate) to afford **26f** as a pale yellow oil (358 mg, 86% yield). ^{1}H NMR (400 MHz, $CDCl_3$) δ 7.32 – 7.24 (m, 2H), 6.85 – 6.81 (m, 2H), 6.40 (d, *J* = 15.9 Hz, 1H), 6.11 (dt, *J* = 15.8, 6.9 Hz, 1H), 3.80 (s, 3H), 2.49 (s, 1H), 2.47 – 2.36 (m, 2H), 1.88 – 1.80 (m, 2H), 1.54 (s, 3H). ^{13}C NMR (125 MHz, $CDCl_3$) δ 158.8, 130.4, 129.8, 127.8, 127.1 (2C), 113.9 (2C), 87.4, 71.7, 68.0, 55.3, 43.0, 29.9, 28.4. HRMS-ESI: *m/z* calculated for $C_{15}H_{19}O_2$ [M+H]$^+$: 231.1385, found: 231.1380.

(*E*)-((7-(4-Methoxyphenyl)-3-methylhept-6-en-1-yn-3-yl)oxy)trimethylsilane (26g)

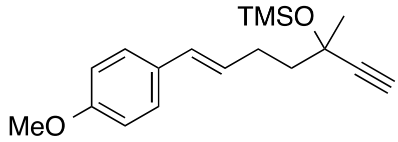

TMSOTf (120.0 µL, 1.50 equiv) was added dropwise to a solution of **26f** (100 mg, 1 equiv) and Et_3N (110.0 µL, 1.80 equiv) in CH_2Cl_2 (0.1 M) at 0 ºC. The resulting mixture was allowed to warm up to 23 ºC and stirred overnight. The crude material was purified by flash column chromatography (95:5:1 hexane/ethyl acetate/Et_3N) to afford **26g** as a pale yellow oil (85 mg, 75%). ^{1}H NMR (400 MHz, $CDCl_3$) δ 7.26 (dd, *J* = 6.6, 5.0 Hz, 2H), 6.86 – 6.81 (m, 2H), 6.36 (d, *J* = 15.8 Hz, 1H), 6.11 (dt, *J* = 15.8, 6.8 Hz, 1H), 3.80 (s, 3H), 2.47 (s, 1H), 2.38 (dt, *J* = 10.9, 5.8 Hz, 2H), 1.83 – 1.73 (m, 2H), 1.50 (s, 3H), 0.20 (d, *J* = 3.0 Hz, 9H). ^{13}C NMR (100 MHz, $CDCl_3$) δ 158.6, 130.7, 129.1, 128.4, 127.0 (2C), 113.9 (2C), 87.9, 72.5, 69.1, 55.3, 44.7, 31.2, 28.3, 1.9 (3C). HRMS-ESI: *m/z* calculated for $C_{18}H_{27}O_2Si$ [M+H]$^+$: 303.1780, found: 303.1782.

(*E*)-6-(2-Bromophenyl)hex-5-en-2-one

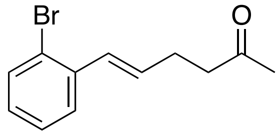

Methyl 3-oxobutanoate (1.7 mL, 1 equiv) was added dropwise to a suspension of NaH (60 wt% in mineral oil, 582 mg, 1.05 equiv) in THF (0.05 M) at 0 ºC. The resulting mixture was allowed to warm up to 23 ºC and stirred for 30 min. Then a solution of (*E*)-1-bromo-2-(3-bromoprop-1-en-1-yl)benzene[6] (5.84 g, 1.00 equiv) in THF (0.1 M) was added dropwise and the reaction mixture was stirred at 23 ºC overnight. The reaction was quenched with saturated NH_4Cl. Ethyl acetate was added and phases were separated. The aqueous layer was extracted with ethyl acetate (3 times). The combined organic extracts were washed with brine, dried over Na_2SO_4, filtered and concentrated under reduced pressure. Decarboxylation was performed subsequently in one-pot with KOH (3.70 g, 5.00 equiv) and water (50 mL). The reaction mixture was stirred at 60 ºC for 4-5 h (TLC monitoring) and then cooled down to 23 ºC. Ethyl acetate was added and phases were separated. The aqueous layer was extracted with ethyl acetate (3 times) and the combined organic extracts were washed with brine, dried over Na_2SO_4, filtered and concentrated under reduced pressure. Crude was directly engaged in the subsequent step without further purification (4.67 g crude).

(*E*)-7-(2-Bromophenyl)-3-methylhept-6-en-1-yn-3-ol (26h)

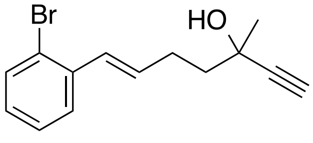

Ethynylmagnesium bromide (0.5 M in THF, 11.0 mL, 1.2 equiv) was added dropwise to a solution of (*E*)-6-(2-bromophenyl)hex-5-en-2-one (1.19 g crude) in THF (0.1 M) at -10 ºC. The mixture was allowed to warm up to 23 ºC and stirred overnight. The reaction was quenched with saturated NH_4Cl and ethyl acetate was added. Phases were separated and the aqueous layer was extracted with ethyl acetate (3 times). The combined organic extracts were dried over Na_2SO_4, filtered and concentrated under reduced pressure. The residue was purified by flash column chromatography (15:1 pentane/diethyl ether) to afford **26h** as a pale yellow oil (730 mg, 56% yield). ^{1}H NMR (500 MHz, $CDCl_3$) δ 7.53 (dd, *J* = 8.0, 1.3 Hz, 1H), 7.48 (dd, *J* = 7.8, 1.7 Hz, 1H), 7.26 – 7.22 (m, 1H), 7.06 (ddd, *J* = 7.9, 7.3, 1.7 Hz, 1H), 6.78 (dt, *J* = 15.7, 1.7 Hz, 1H), 6.21 (dt, *J* = 15.7, 6.9 Hz, 1H), 2.59 – 2.43 (m, 3H), 2.04 – 2.00 (m, 1H), 1.95 – 1.82 (m, 2H), 1.56 (s, 3H). ^{13}C NMR (125 MHz, $CDCl_3$) δ 137.6, 133.3, 133.0, 129.4, 128.5, 127.6, 127.0, 123.3, 87.4, 72.0, 68.1, 42.8, 30.2, 28.6. HRMS-ESI: *m/z* calculated for $C_{14}H_{16}BrO$ $[M+H]^+$: 279.0379, found: 279.0368; *m/z* calculated for $C_{14}H_{14}Br$ $[M-OH]^+$: 261.0273, found: 261.0273.

6.2. General Procedure for the Gold(I)-catalyzed Cyclization via Endo-type Skeletal Rearrangement

Gold catalyst **A** (2 mol%) was added to a solution of the corresponding enyne in CH_2Cl_2 (0.1 M) with activated powdered 4 Å MS. The reaction mixture was stirred at 23 ºC for 4 h and quenched with Et_3N. The solution was filtered through a short pad of silica (elution EtOAc) and concentrated under reduced pressure. The residue was purified by flash chromatography over silica gel.

(*E*)-2-Benzylidenecyclohex-3-enol (22)

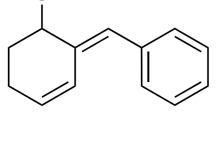

Cyclization performed following the general procedure for *Endo-type Skeletal Rearrangement* with **20b** (100 mg, 1.00 equiv). Purified by flash column chromatography (10:1 cyclohexane/ethyl acetate) to afford **22** as a pale yellow oil (64.0 mg, 64% yield). ^{1}H NMR (400 MHz, $CDCl_3$) δ 7.36 – 7.29 (m, 4H), 7.26 – 7.20 (m, 1H), 6.59 – 6.54 (m, 2H), 5.94 (dtd, *J* = 9.8, 4.0, 1.4 Hz, 1H), 4.42 (dd, *J* = 6.9, 3.8 Hz, 1H), 2.48 – 2.36 (m, 1H), 2.32 – 2.22 (m, 1H), 2.00 – 1.92 (m, 2H), 1.62 (s, 1H). ^{13}C NMR (100 MHz, $CDCl_3$) δ 138.1, 137.1, 131.6, 129.4 (2C), 128.3 (2C), 126.9, 125.2, 123.4, 71.0, 30.9, 23.1. HRMS-ESI: *m/z* calculated for $C_{13}H_{13}$ [M-OH]$^+$: 169.1012, found: 169.1011.

(*E*)-((2-Benzylidenecyclohex-3-en-1-yl)methylene)dibenzene (23)

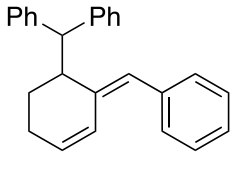

Cyclization performed following the general procedure for *Endo-type Skeletal Rearrangement* with **20b** (100 mg, 1.00 equiv) without using activated powdered 4 Å MS. Purified by preparative TLC (: cyclohexane/ethyl acetate) to afford **23** as a white solid (42 mg, 46% yield).

mp: 153-157 ºC ^{1}H NMR (500 MHz, $CDCl_3$) δ 7.46 (app. dd, *J* = 8.1, 1.1 Hz, 2H), 7.35 (t, *J* = 7.7 Hz, 2H), 7.26 – 7.18 (m, 7H), 7.17 – 7.11 (m, 2H), 6.85 (d, *J* = 6.4 Hz, 2H), 6.44 (app. dq, *J* = 10.2, 1.0 Hz, 1H), 5.96 – 5.91 (m, 1H), 5.53 (s, 1H), 4.01 (d, *J* = 11.7 Hz, 1H), 3.18 (dt, *J* = 11.8, 3.4 Hz, 1H), 2.24 (m, 1H), 2.05 (dq, *J* = 17.8, 3.4 Hz, 1H), 1.89 (dt, *J* = 8.8, 3.3 Hz, 2H). ^{13}C NMR (100 MHz, $CDCl_3$) δ 144.7, 143.4, 137.7, 136.3, 130.6, 129.2, 128.8 (2C), 128.7 (4C), 128.2 (2C), 128.2 (2C), 128.0 (2C), 126.5, 126.3, 126.1, 124.5, 52.9, 46.0, 25.3, 22.3. MS-ESI: *m/z* calculated for $C_{26}H_{24}Na$ [M+Na]$^+$: 359.17, found: 359.2.

(*E*)-2-Benzylidene-1-methylcyclohex-3-en-1-ol (27a)

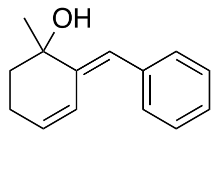

Cyclization performed following the general procedure for *Endo-type Skeletal Rearrangement* with **26a** (70.0 mg, 1.00 equiv). Purified by flash column chromatography (9:1 cyclohexane/ethyl acetate) to afford **27a** as a pale yellow oil (36.0 mg, 52% yield). ^{1}H NMR (400 MHz, $CDCl_3$) δ 7.30 (dd, *J* = 12.9, 7.6 Hz, 4H), 7.21 (d, *J* = 6.7 Hz, 1H), 6.80 (s, 1H), 6.53 (d, *J* = 9.9 Hz, 1H), 5.88 – 5.81 (m, 1H), 2.33 – 2.26 (m, 2H), 1.89 – 1.80 (m, 2H), 1.40 (s, 3H). ^{13}C NMR (100 MHz, $CDCl_3$) δ 142.8, 137.4, 129.7, 129.3 (2C), 128.1 (2C), 126.5, 124.1, 122.0, 71.2, 37.8, 26.7, 24.9. HRMS-ESI: *m/z* calculated for $C_{14}H_{15}$ [M-OH]$^-$: 183.1174, found: 183.1174. **(*E*)-((2-Benzylidene-1-methylcyclohex-3-en-1-yl)oxy)trimethylsilane (27b)**

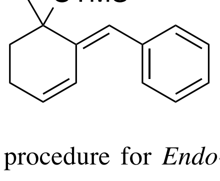

Cyclization performed following the general procedure for *Endo-type Skeletal Rearrangement* with **26b** (54.0 mg, 1.00 equiv). Purified by flash column chromatography (9:1 cyclohexane/ethyl acetate) to afford **27b** as a pale yellow oil (51.0 mg, 95% yield). ^{1}H NMR (400 MHz, $CDCl_3$) δ 7.35 – 7.30 (m, 4H), 7.23 (dd, *J* = 9.7, 4.3

Hz, 1H), 6.74 (s, 1H), 6.51 (d, *J* = 10.0 Hz, 1H), 5.84 – 5.78 (m, 1H), 2.39 – 2.22 (m, 2H), 1.98 – 1.83 (m, 2H), 1.42 (s, 3H), 0.18 (s, 9H). ^{13}C NMR (100 MHz, $CDCl_3$) δ 142.5, 137.9, 129.3 (2C), 128.0 (2C), 126.3, 124.2, 122.8, 74.1, 37.6, 28.4, 24.9, 2.6 (3C). HRMS-ESI: *m/z* calculated for $C_{17}H_{25}OSi$ $[M+H]^+$: 273.1596, found: 273.1598.

(*E*)-1-Methyl-2-(4-nitrobenzylidene)cyclohex-3-en-1-ol (27c)

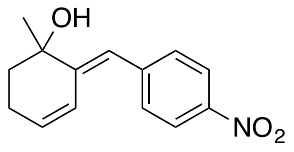

Cyclization performed following the general procedure for *Endo-type Skeletal Rearrangement* with **26c** (70.0 mg, 1.00 equiv). Purified by flash column chromatography (9:1 cyclohexane/ethyl acetate) to afford **27c** as a pale yellow oil (50.0 mg, 88% yield). ^{1}H NMR (400 MHz, $CDCl_3$) δ 8.23 – 8.13 (m, 2H), 7.50 – 7.35 (m, 2H), 6.83 (s, 1H), 6.47 – 6.43 (m, 1H), 6.00 – 5.94 (m, 1H), 2.47 – 2.26 (m, 2H), 1.97 – 1.79 (m, 2H), 1.59 (s, 1H), 1.40 (d, *J* = 0.6 Hz, 3H). ^{13}C NMR (125 MHz, $CDCl_3$) δ 146.1, 144.7, 132.3, 129.9 (2C), 123.4 (2C), 123.2, 119.9, 77.3, 76.8, 71.3, 37.6, 26.7, 25.1. HRMS-ESI: *m/z* calculated for $C_{14}H_{16}NO_3$ $[M+H]^+$: 245.1052, found: 245.1060.

(*E*)-Trimethyl((1-methyl-2-(4-nitrobenzylidene)cyclohex-3-en-1-yl)oxy)silane (27d)

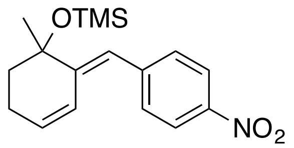

Cyclization performed following the general procedure for *Endo-type Skeletal Rearrangement* with **26d** (48.0 mg, 1.00 equiv). Purified by flash column chromatography (9:1 cyclohexane/ethyl acetate) to afford **27d** as a pale yellow oil (38.0 mg, 79% yield).^{1}H NMR (400 MHz, $CDCl_3$) δ 8.18 (d, *J* = 8.5 Hz, 2H), 7.42 (d, *J* = 8.6 Hz, 2H), 6.74 (s, 1H), 6.42 (d, *J* = 10.0 Hz, 1H), 5.95 – 5.88 (m, 1H), 2.32 – 2.20 (m, 2H), 1.97 – 1.85 (m, 2H), 1.40 (s, 3H), 0.18 (s, 9H). ^{13}C NMR (100 MHz, $CDCl_3$) δ 146.5, 146.0, 145.2, 131.8, 129.9 (2C), 123.4 (2C), 123.3, 120.5, 74.2, 37.3, 28.1, 25.2, 2.6 (3C). HRMS-ESI: *m/z* calculated for $C_{17}H_{24}NO_3Si$ $[M+H]^+$: 318.1525, found: 318.1522.

(*E*)-1-Methyl-2-(2-nitrobenzylidene)cyclohex-3-en-1-ol (27e)

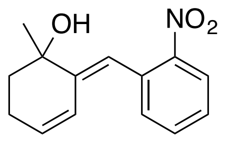

Cyclization performed following the general procedure for *Endo-type Skeletal Rearrangement* with **26e** (51.0 mg, 1.00 equiv). Purified by flash column chromatography (9:1 cyclohexane/ethyl acetate) to afford **27e** as a pale yellow oil (74.3.0 mg, 98% yield). ^{1}H NMR (400 MHz, $CDCl_3$) δ 8.04 (dd, *J* = 8.2, 1.3 Hz, 1H), 7.56 (td, *J* = 7.6, 1.3 Hz, 1H), 7.45 – 7.34 (m, 2H), 7.03 (s, 1H), 6.14 – 6.10 (m, 1H), 5.87 – 5.81 (m, 1H), 2.42 – 2.22 (m, 2H), 1.93 – 1.81 (m, 2H), 1.71 (s, 1H), 1.43 (s, 3H). ^{13}C NMR (100 MHz, $CDCl_3$) δ 148.5, 144.5, 132.9, 132.7, 131.3, 127.7, 124.8, 123.4, 118.8, 71.3, 37.4, 26.9, 25.0. HRMS-ESI: *m/z* calculated for $C_{14}H_{15}NNaO_3$ $[M+Na]^+$: 268.0944, found: 268.0943.

(*E*)-2-(2-Bromobenzylidene)-1-methylcyclohex-3-en-1-ol (27h)

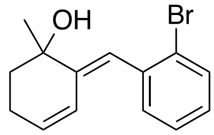

Cyclization performed following the general procedure for *Endo-type Skeletal Rearrangement* with **26h** (60.0 mg, 1.00 equiv). Purified by flash column chromatography (10:1 cyclohexane/ethyl acetate) to afford **27h** as white solid (74.0 mg, 74% yield) that crystalized upon drying under high vacuum (see crystallographic data). mp: 77 – 80 ºC. ^{1}H NMR (400 MHz, $CDCl_3$) δ 7.63 – 7.56 (m, 1H), 7.32 – 7.27 (m, 2H), 7.16 – 7.08 (m, 1H), 6.82 – 6.75 (m, 1H), 6.35 – 6.25 (m, 1H), 5.90 – 5.80 (m, 1H), 2.43 – 2.22 (m, 2H), 1.94 – 1.85 (m, 2H), 1.46 (s, 3H). ^{13}C NMR (100 MHz, $CDCl_3$) δ 143.9, 137.6, 132.7, 131.6, 130.5, 128.3, 126.8, 124.6, 123.9, 122.2, 71.4, 37.6, 27.1, 25.0. HRMS-ESI: *m/z* calculated for $C_{14}H_{14}Br$ $[M-OH]^+$: 261.0273, found: 261.0286.

7. X-Ray crystallographic data

1-(2-Methyl-5-(2-(4-nitrophenoxy)propan-2-yl)cyclopent-1-en-1-yl)-1a,6b-dihydro-1H-benzo[b]cyclopropa[d]thiophene (15g)

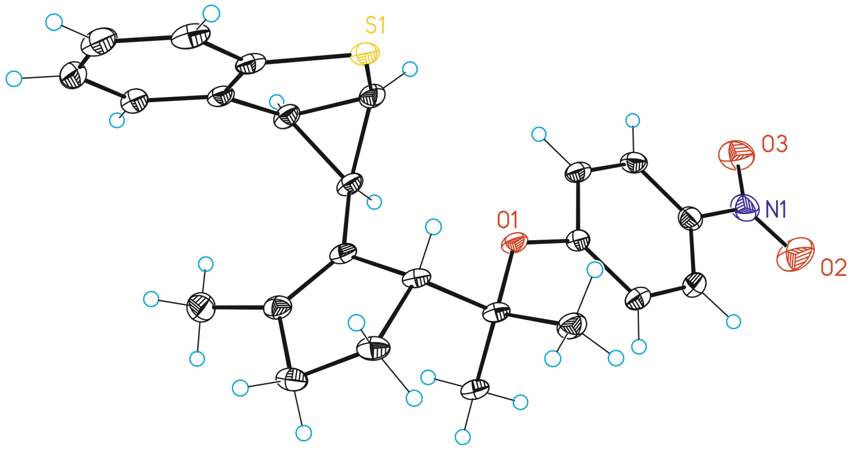

Table S1. Crystal data and structure refinement for **15g** (major isomer).

Empirical formula	C24 H25 N O3 S
Formula weight	407.51
Temperature	100(2) K
Wavelength	0.71073 Å
Crystal system	Monoclinic
Space group	P2(1)/n
Unit cell dimensions	a = 9.074(3)Å a= 90°.
	b = 20.906(5)Å b = 110.872(7)°.
	c = 11.289(3)Å g = 90°.
Volume	2001.1(9) Å3
Z	4
Density (calculated)	1.353 Mg/m3
Absorption coefficient	0.188 mm-1
F(000)	864
Crystal size	0.20 x 0.20 x 0.20 mm3
Theta range for data collection	2.163 to 31.511°.
Index ranges	-8<=h<=13,-30<=k<=28,-16<=l<=13
Reflections collected	14518

Independent reflections 6425[R(int) = 0.0388]

Completeness to theta =31.511° 96.200005%

Absorption correction Empirical

Max. and min. transmission 0.963 and 0.741

Refinement method Full-matrix least-squares on F2

Data / restraints / parameters 6425/ 0/ 265

Goodness-of-fit on F2 0.986

Final R indices [I>2sigma(I)] R1 = 0.0488, wR2 = 0.1338

R indices (all data) R1 = 0.0660, wR2 = 0.1419

Largest diff. peak and hole 0.862 and -0.492 e.Å-3

1,3,3-Trimethyl-7-methylene-2-(4-nitrophenyl)-2-azabicyclo[2.2.1]heptane (19)

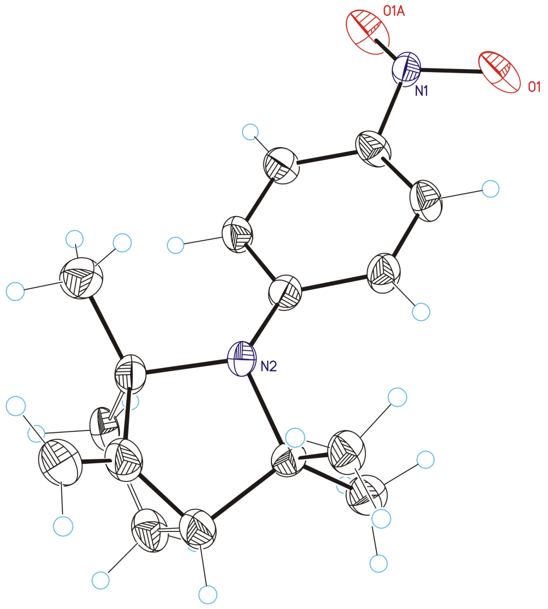

Table S2. Crystal data and structure refinement for **19**.

Identification code MR466_0m

Empirical formula C8 H10 N O

Formula weight 136.17

Temperature 293(2) K

Wavelength 0.71073 Å

Crystal system Tetragonal

Space group P4(3)2(1)2

Unit cell dimensions a = 8.391 Å α= 90.00 °.

b = 8.391 Å β = 90.00 °.

c = 20.584 Å   γ = 90.00 °.

Volume 1449.3 Å3

Z 8

Density (calculated) 1.248 Mg/m3

Absorption coefficient 0.083 mm-1

F(000) 584

Crystal size 0.15 x 0.1 x 0.1 mm3

Theta range for data collection 2.62 to 28.31 °.

Index ranges -11 <=h<=11 ,-10 <=k<=11 ,-27 <=l<=27

Reflections collected 1808

Independent reflections 1610 [R(int) = 0.0662]

Completeness to theta =28.31 ° 0.998 %

Absorption correction Empirical

Max. and min. transmission ? and ?

Refinement method Full-matrix least-squares on F2

Data / restraints / parameters 1808 / 50 / 137

Goodness-of-fit on F2 1.119

Final R indices [I>2sigma(I)] R1 = 0.0441 , wR2 = 0.1120

R indices (all data) R1 = 0.0518 , wR2 = 0.1173

Largest diff. peak and hole 0.218 and -0.235 e.Å-3

((((*S*)-2-((*S*)-2-Methyl-5-(2-(4-nitrophenoxy)propan-2-yl)cyclopent-1-en-1-yl)cyclopropane-1,1-diyl)bis(methylene))bis(oxy))bis(*tert*-butyldimethylsilane) (6c)

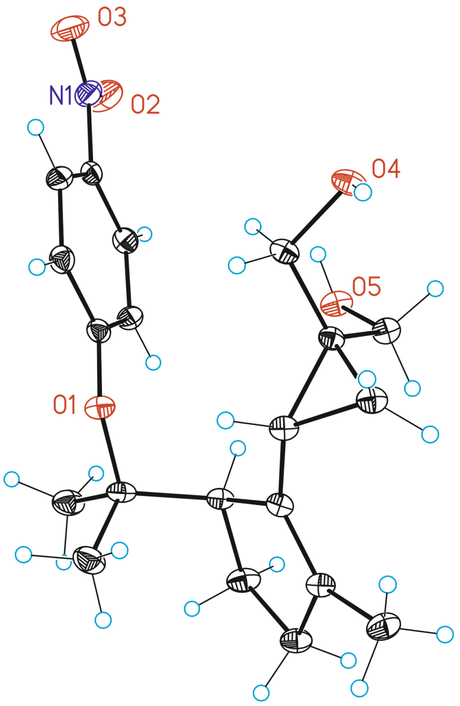

Table S3. Crystal data and structure refinement for **6c**.

Identification code	OS42	
Empirical formula	C20 H27 N O5	
Formula weight	361.42	
Temperature	100(2) K	
Wavelength	0.71073 Å	
Crystal system	Monoclinic	
Space group	P2(1)/n	
Unit cell dimensions	a = 16.5374(14)Å	α= 90°.
	b = 6.1295(6)Å	β = 103.160(10)°.
	c = 18.2483(18)Å	γ = 90°.
Volume	1801.2(3) Å3	
Z	4	
Density (calculated)	1.333 Mg/m3	
Absorption coefficient	0.095 mm-1	
F(000)	776	
Crystal size	0.1 x 0.1 x 0.04 mm3	
Theta range for data collection	2.530 to 29.842°.	
Index ranges	-22<=h<=22,-8<=k<=7,-25<=l<=25	

Reflections collected 15593

Independent reflections 4377[R(int) = 0.0835]

Completeness to theta =29.842° 84.3%

Absorption correction Multi-scan

Max. and min. transmission 0.996 and 0.766

Refinement method Full-matrix least-squares on F2

Data / restraints / parameters 4377/ 0/ 240

Goodness-of-fit on F2 1.001

Final R indices [I>2sigma(I)] R1 = 0.0499, wR2 = 0.1048

R indices (all data) R1 = 0.1013, wR2 = 0.1213

Largest diff. peak and hole 0.307 and -0.281 e.Å-3

(*E*)-((2-Benzylidenecyclohex-3-en-1-yl)methylene)dibenzene (23)

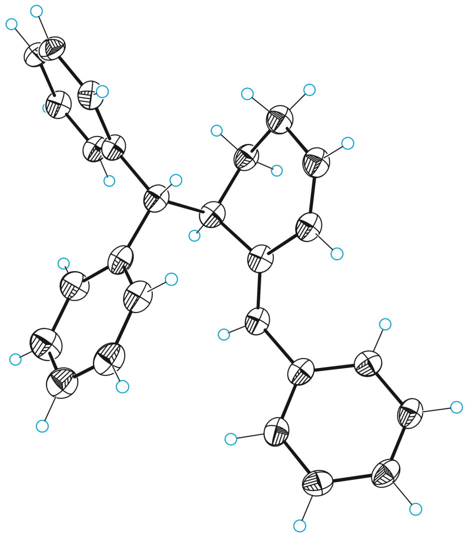

Table S4. Crystal data and structure refinement for **23**.

Identification code mo_PCR_N073B_05

Empirical formula C26 H24

Formula weight 336.45

Temperature 100(2) K

Wavelength 0.71073 Å

Crystal system Monoclinic

Space group P2(1)/n

Unit cell dimensions a = 9.529(3)Å a= 90°.

b = 9.688(3)Å b = 92.057(10)°.

c = 20.056(6)Å   g = 90°.

Volume 1850.3(9) Å3

Z 4

Density (calculated) 1.208 Mg/m3

Absorption coefficient 0.068 mm-1

F(000) 720

Crystal size 0.25 x 0.08 x 0.04 mm3

Theta range for data collection 2.032 to 24.884°.

Index ranges -11<=h<=11,0<=k<=11,0<=l<=23

Reflections collected 6557

Independent reflections 6557[R(int) =?]

Completeness to theta =24.884° 99.2%

Absorption correction Empirical

Max. and min. transmission 0.997 and 0.552

Refinement method Full-matrix least-squares on F2

Data / restraints / parameters 6557/ 0/ 236

Goodness-of-fit on F2 1.037

Final R indices [I>2sigma(I)] R1 = 0.0757, wR2 = 0.1671

R indices (all data) R1 = 0.1343, wR2 = 0.2020

Largest diff. peak and hole 0.304 and -0.296 e.Å-3

(E)-2-(2-Bromobenzylidene)-1-methylcyclohex-3-en-1-ol (27h)

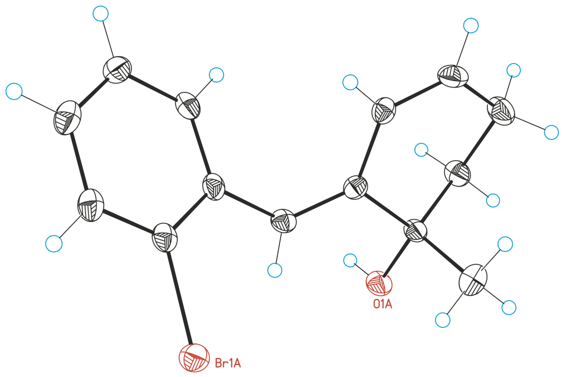

Table S5. Crystal data and structure refinement for **27h**.

Identification code mo_API10075_0m

Empirical formula C14 H15 Br O

Formula weight 279.17

Temperature 100(2) K

Wavelength 0.71073 Å

Crystal system Triclinic

Space group P-1

Unit cell dimensions a = 7.6576(3) Å a= 95.5550(10) °.

b = 12.5297(4) Å b = 94.2200(10) °.

c = 13.4288(5) Åg = 102.6250(10) °.

Volume 1245.43(8) Å3

Z 4

Density (calculated) 1.489 Mg/m3

Absorption coefficient 3.277 mm-1

F(000) 568

Crystal size 0.10 x 0.10 x 0.06 mm3

Theta range for data collection 2.14 to 28.15 °.

Index ranges -10 <=h<=6 ,-16 <=k<=14 ,-17 <=l<=17

Reflections collected 9522

Independent reflections 5910 [R(int) = 0.0275]

Completeness to theta =28.15 ° 97.0%

Absorption correction Empirical

Max. and min. transmission 0.8276 and 0.7353

Refinement method Full-matrix least-squares on F2

Data / restraints / parameters 5910 / 0 / 293

Goodness-of-fit on F2 1.030

Final R indices [I>2sigma(I)] R1 = 0.0469 , wR2 = 0.0987

R indices (all data) R1 = 0.0790 , wR2 = 0.1106

Largest diff. peak and hole 1.512 and -1.243 e.Å-3

8. NMR Spectra

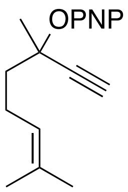
OPNP

1a

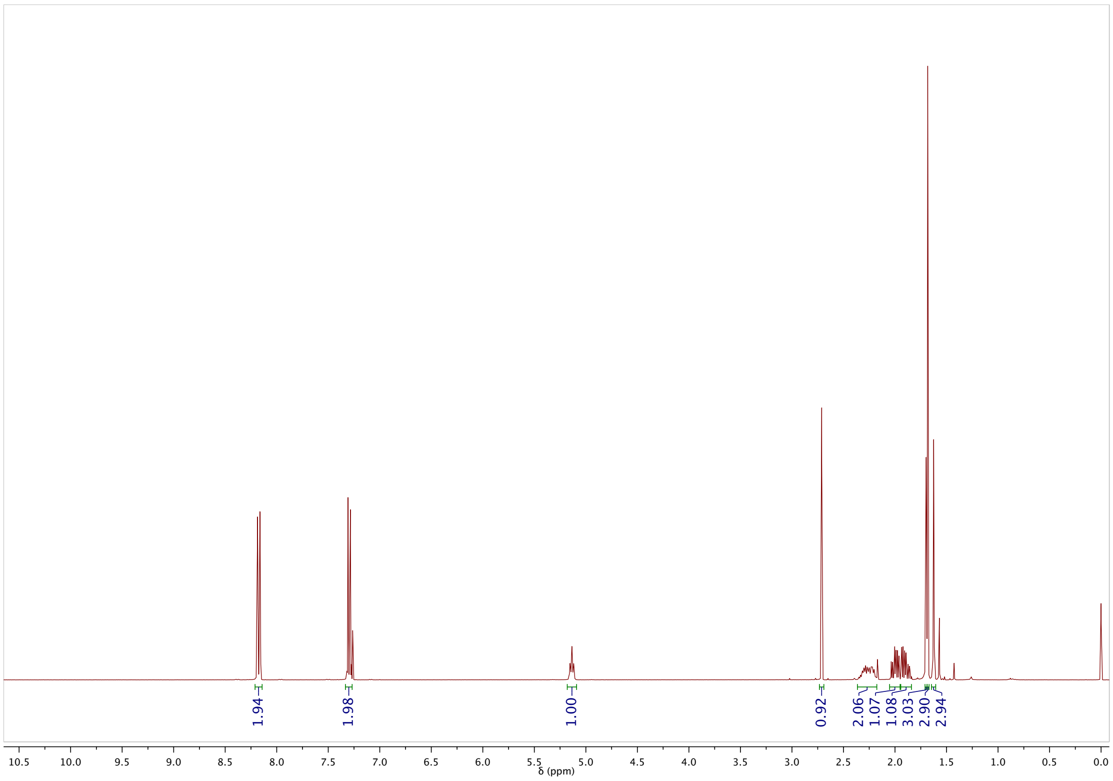
1.94
1.98
1.00
0.92
2.06
1.07
1.08
3.03
2.90
2.94
δ (ppm)

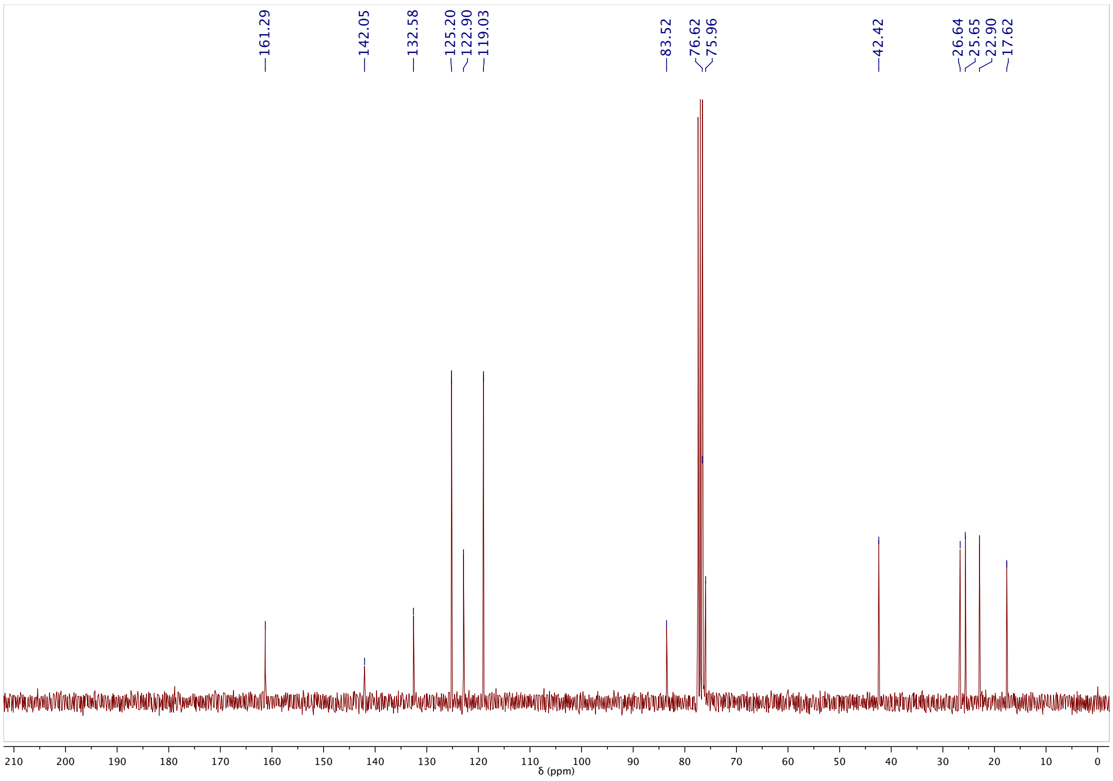
161.29
142.05
132.58
125.20
122.90
119.03
83.52
76.62
75.96
42.42
26.64
25.65
22.90
17.62
δ (ppm)

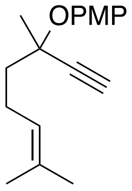
OPMP

1e

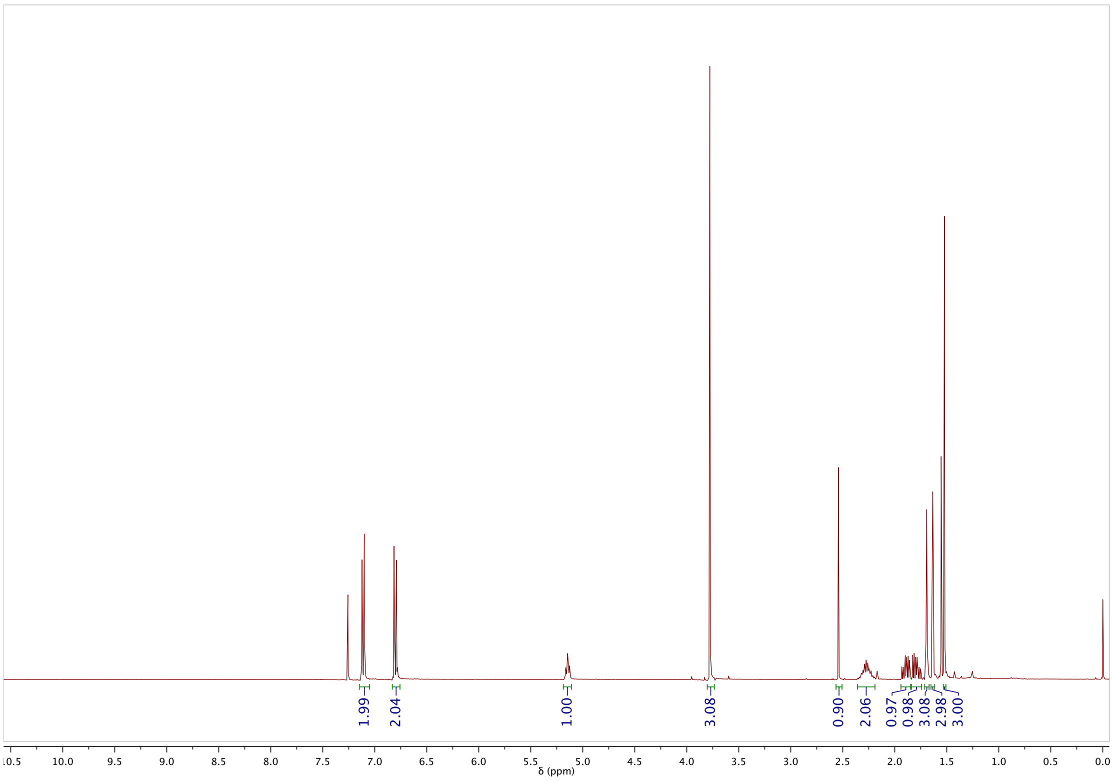
1.99
2.04
1.00
3.08
0.90
2.06
0.97
0.98
3.08
2.98
3.00
10.5
10.0
9.5
9.0
8.5
8.0
7.5
7.0
6.5
6.0
5.5
5.0
4.5
4.0
3.5
3.0
2.5
2.0
1.5
1.0
0.5
0.0
δ (ppm)

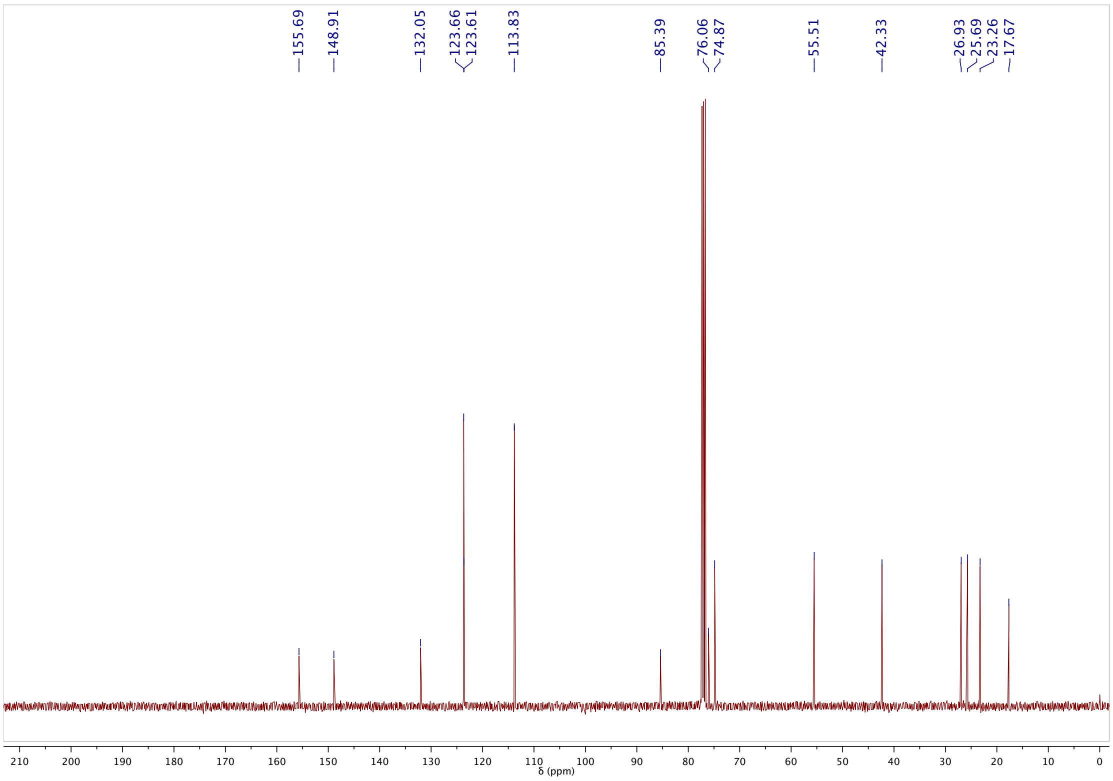
155.69
148.91
132.05
123.66
123.61
113.83
85.39
76.06
74.87
55.51
42.33
26.93
25.69
23.26
17.67
210
200
190
180
170
160
150
140
130
120
110
100
90
80
70
60
50
40
30
20
10
0
δ (ppm)

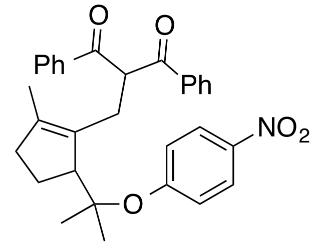

14a

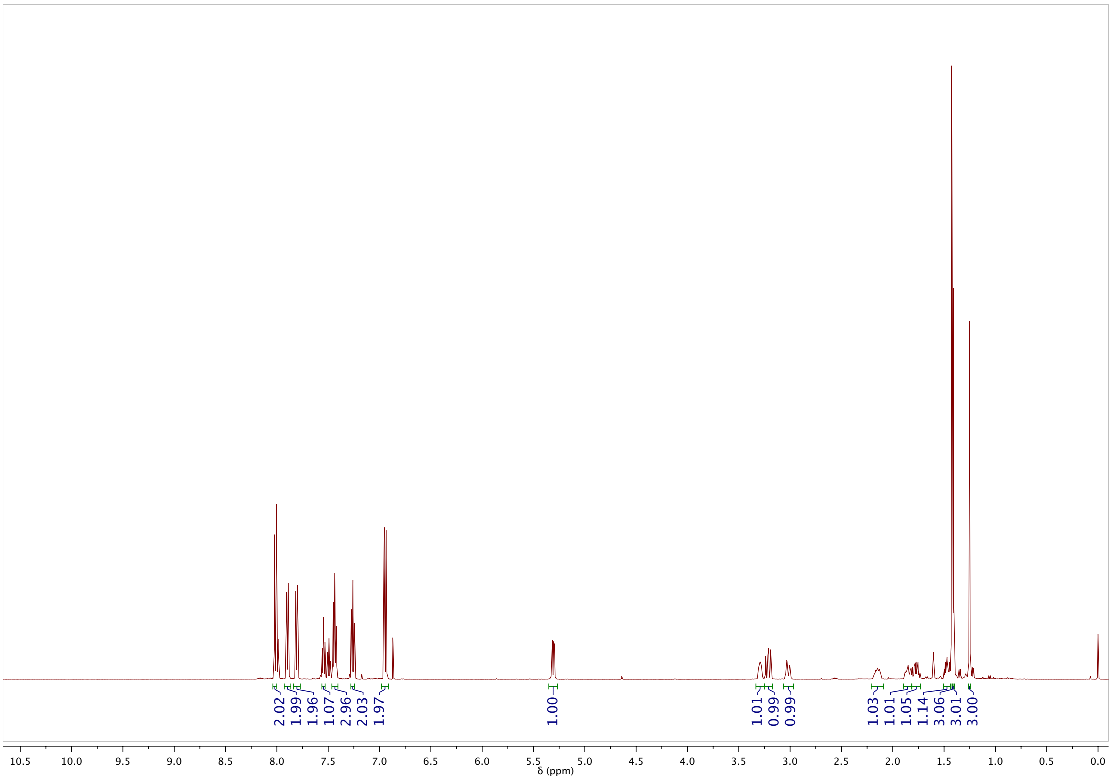

2.02
1.99
1.96
1.07
2.96
2.03
1.97
1.00
1.01
0.99
0.99
1.03
1.01
1.05
1.14
3.06
3.01
3.00
δ (ppm)

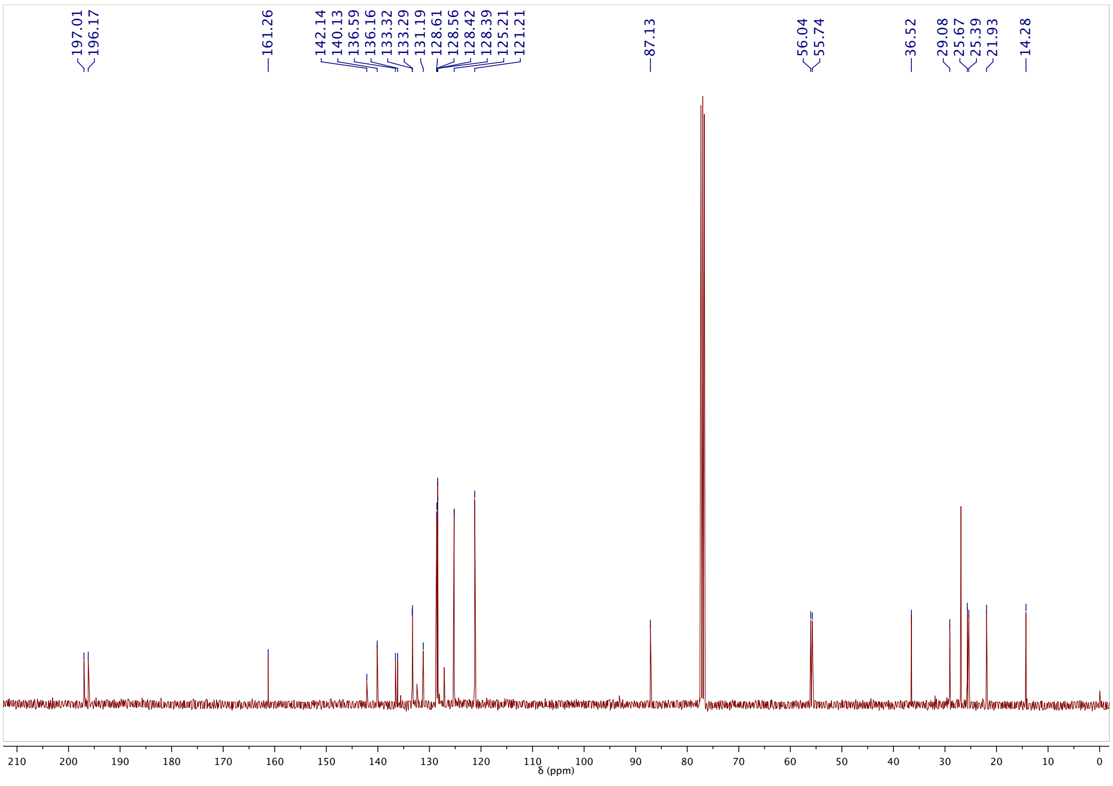

197.01
196.17
161.26
142.14
140.13
136.59
136.16
133.32
133.29
131.19
128.61
128.56
128.42
128.39
125.21
121.21
87.13
56.04
55.74
36.52
29.08
25.67
25.39
21.93
14.28
δ (ppm)

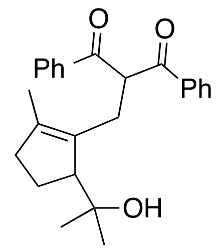

14b

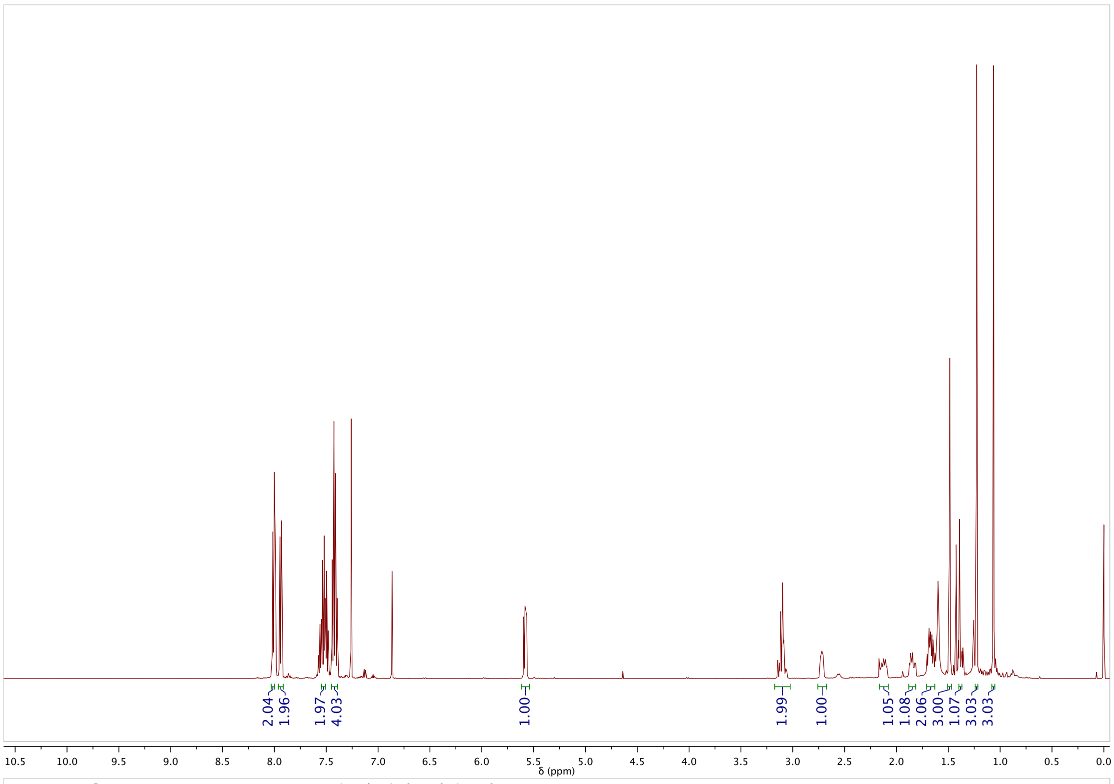

2.04
1.96
1.97
4.03
1.00
1.99
1.00
1.05
1.08
2.06
3.00
1.07
3.03
3.03
δ (ppm)

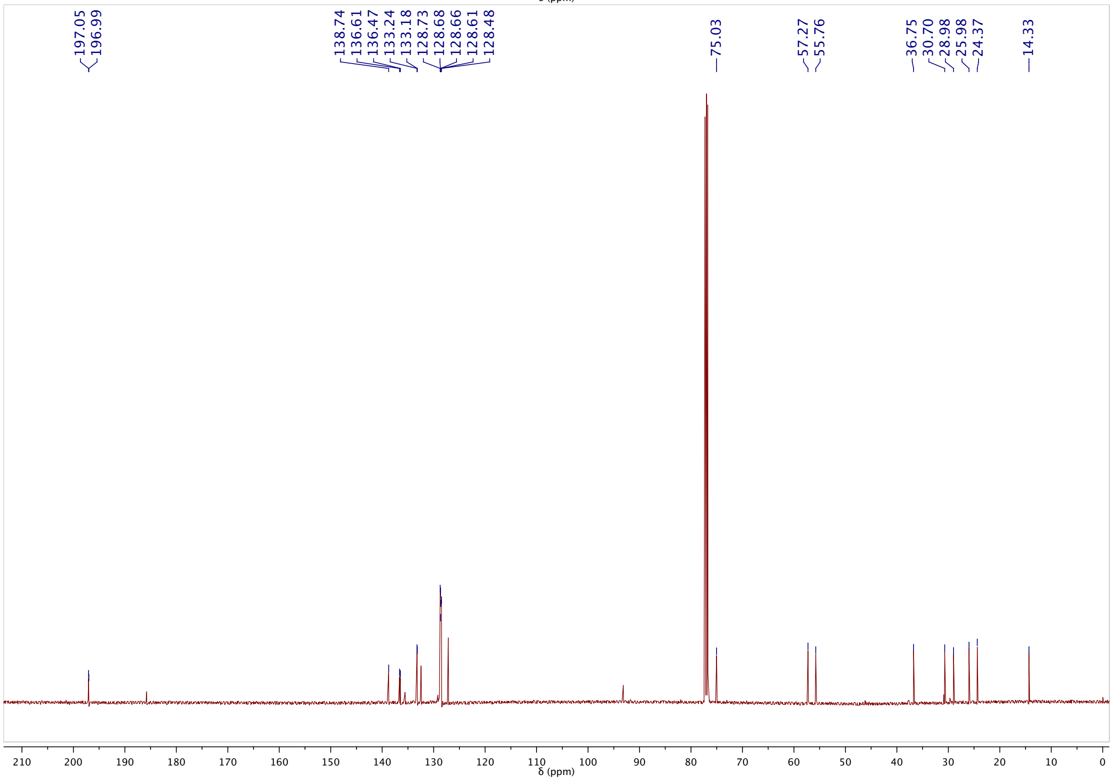

197.05
196.99
138.74
136.61
136.47
133.24
133.18
128.73
128.68
128.66
128.61
128.48
75.03
57.27
55.76
36.75
30.70
28.98
25.98
24.37
14.33
δ (ppm)

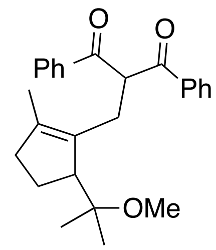
O
O
Ph
Ph
OMe

14c

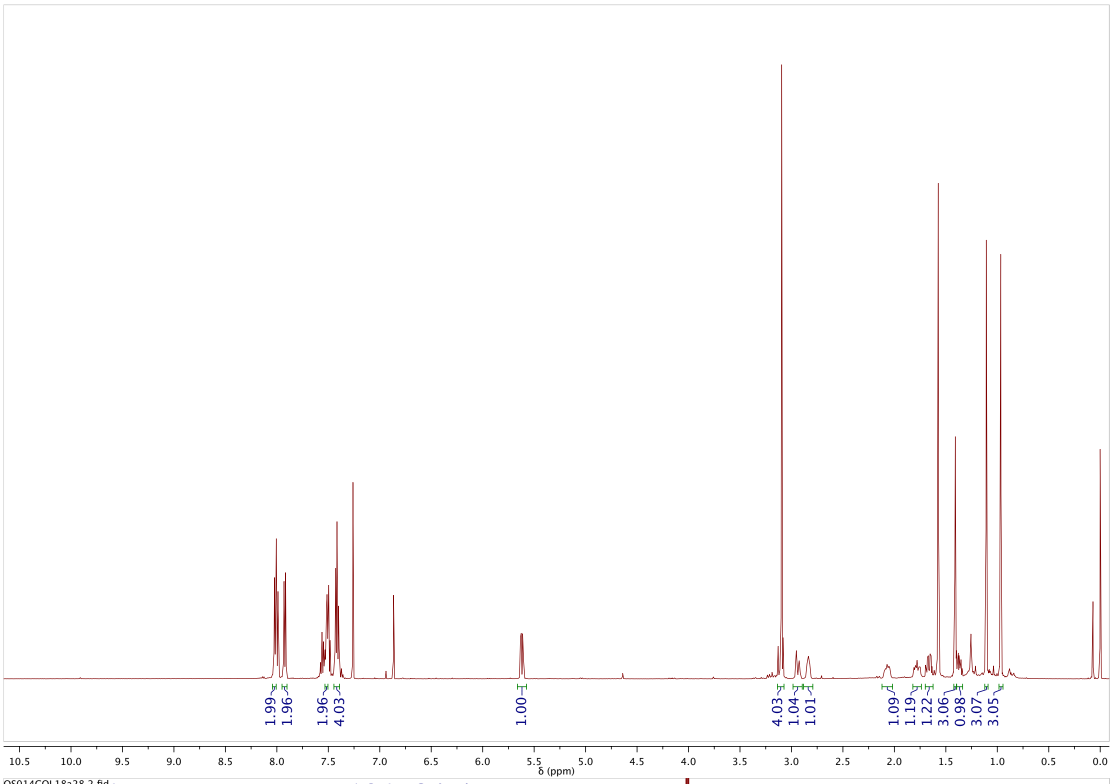
1.99
1.96
1.96
4.03
1.00
4.03
1.04
1.01
1.09
1.19
1.22
3.06
0.98
3.07
3.05
10.5 10.0 9.5 9.0 8.5 8.0 7.5 7.0 6.5 6.0 5.5 5.0 4.5 4.0 3.5 3.0 2.5 2.0 1.5 1.0 0.5 0.0
δ (ppm)

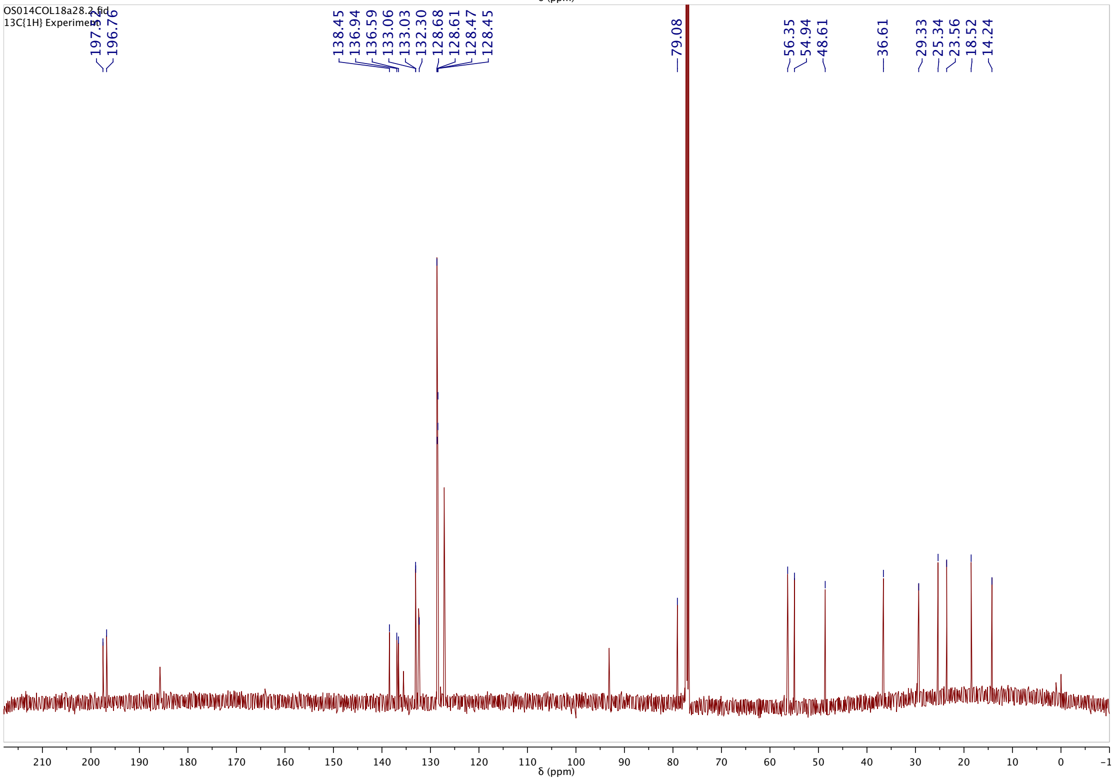
13C{1H} Experiment
196.76
138.45
136.94
136.59
133.06
133.03
132.30
128.68
128.61
128.47
128.45
79.08
56.35
54.94
48.61
36.61
29.33
25.34
23.56
18.52
14.24
210 200 190 180 170 160 150 140 130 120 110 100 90 80 70 60 50 40 30 20 10 0 −1
δ (ppm)

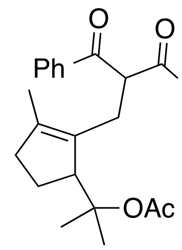

Ph
O
O
Ph
OAc

14d

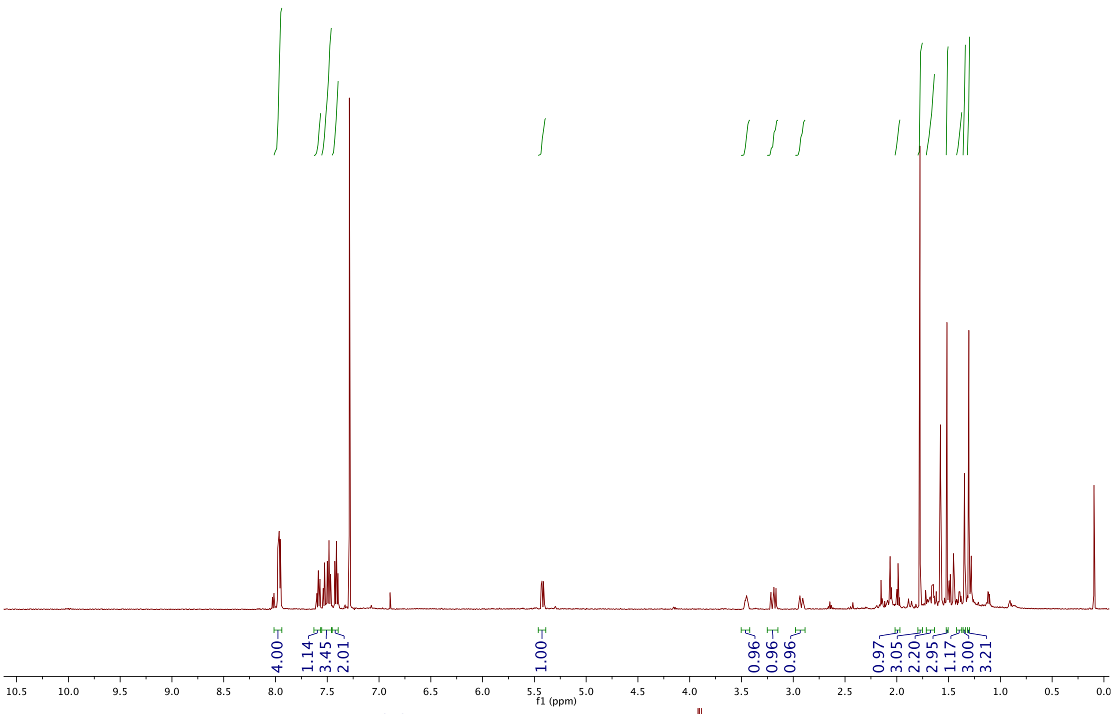

4.00
1.14
3.45
2.01
1.00
0.96
0.96
0.96
0.97
3.05
2.20
2.95
1.17
3.00
3.21
f1 (ppm)

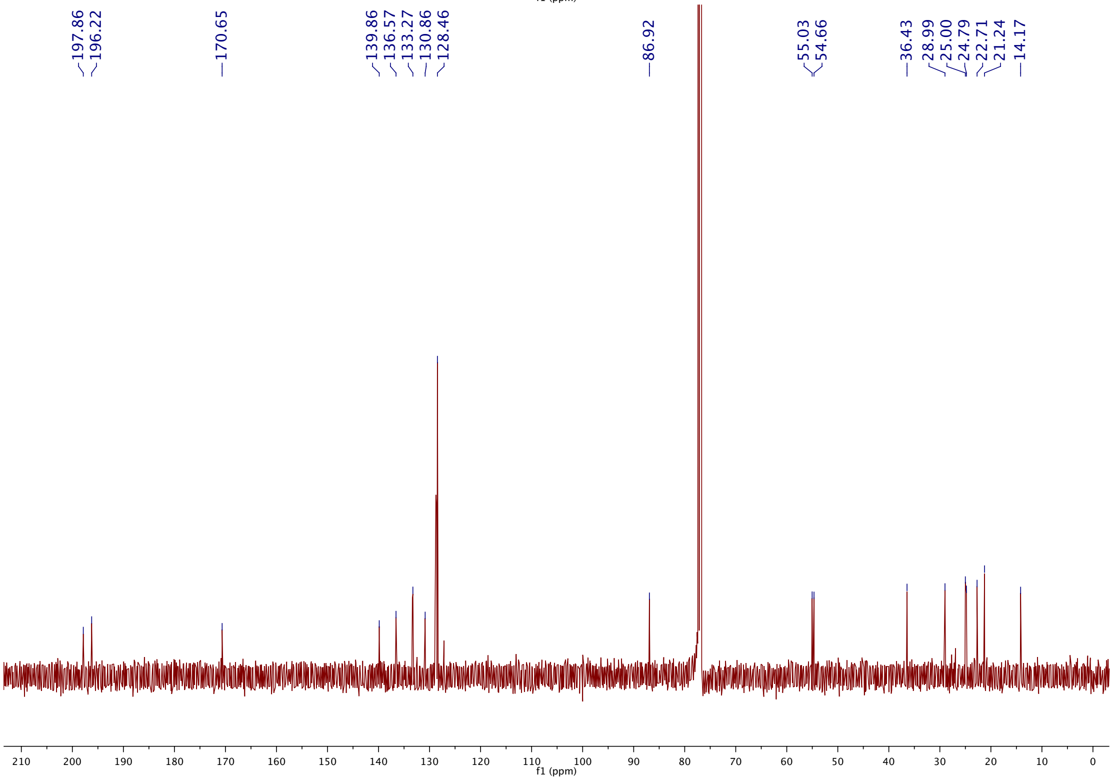

197.86
196.22
170.65
139.86
136.57
133.27
130.86
128.46
86.92
55.03
54.66
36.43
28.99
25.00
24.79
22.71
21.24
14.17
f1 (ppm)

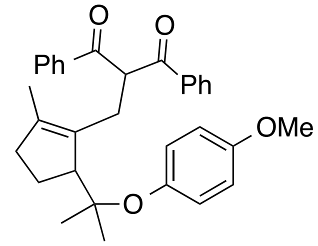
O
O
Ph
Ph
OMe
O

14e

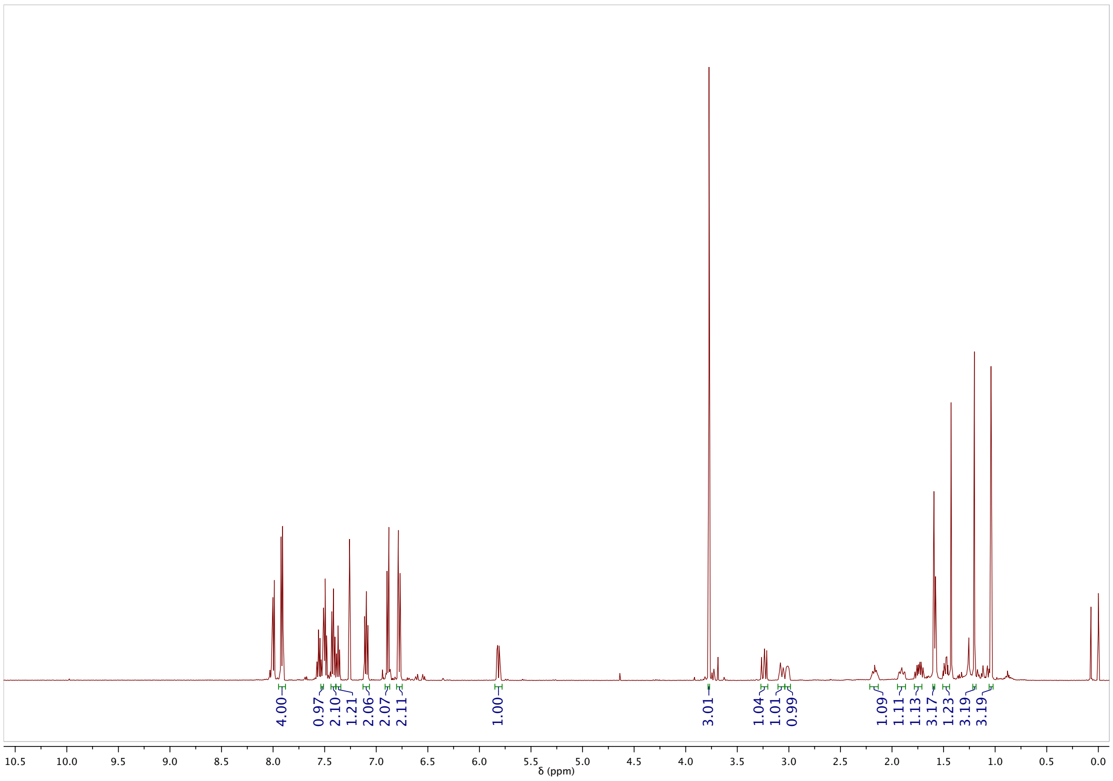
4.00
0.97
2.10
1.21
2.06
2.07
2.11
1.00
3.01
1.04
1.01
0.99
1.09
1.11
1.13
3.17
1.23
3.19
3.19
10.5 10.0 9.5 9.0 8.5 8.0 7.5 7.0 6.5 6.0 5.5 5.0 4.5 4.0 3.5 3.0 2.5 2.0 1.5 1.0 0.5 0.0
δ (ppm)

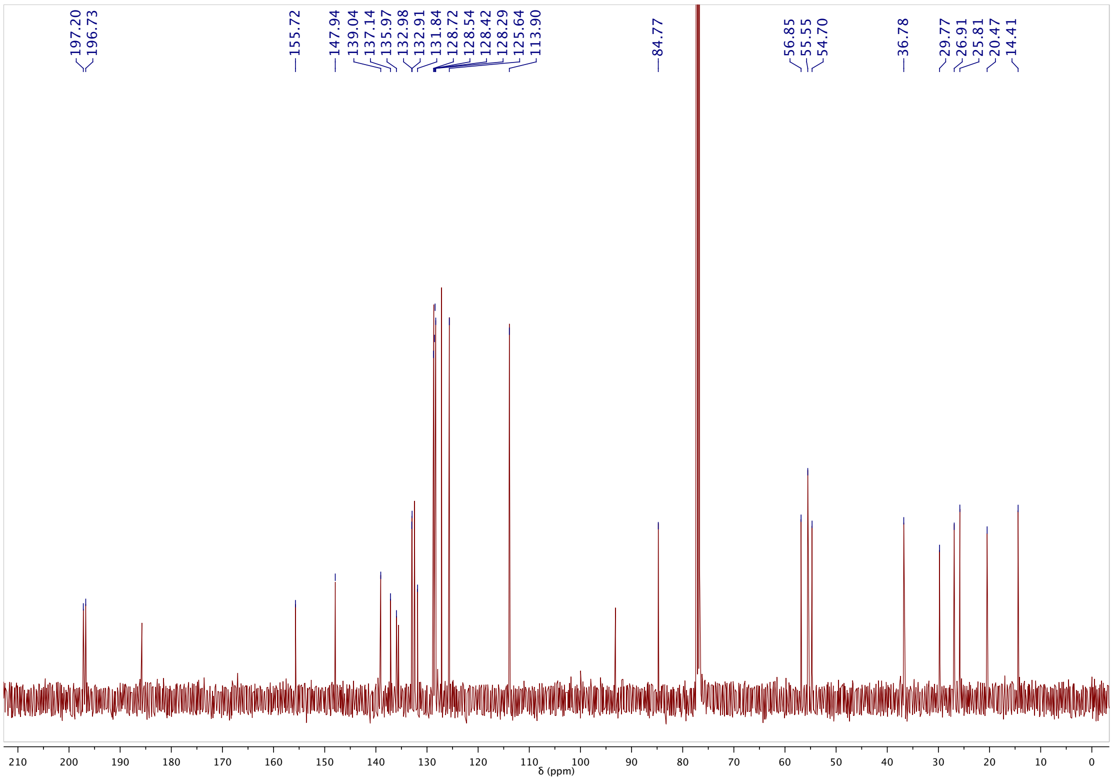
197.20
196.73
155.72
147.94
139.04
137.14
135.97
132.98
132.91
131.84
128.72
128.54
128.42
128.29
125.64
113.90
84.77
56.85
55.55
54.70
36.78
29.77
26.91
25.81
20.47
14.41
210 200 190 180 170 160 150 140 130 120 110 100 90 80 70 60 50 40 30 20 10 0
δ (ppm)

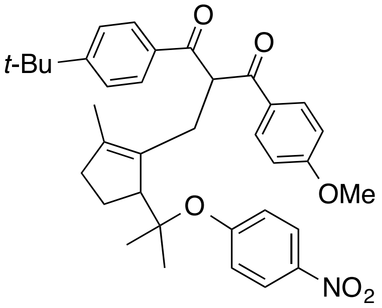

14h

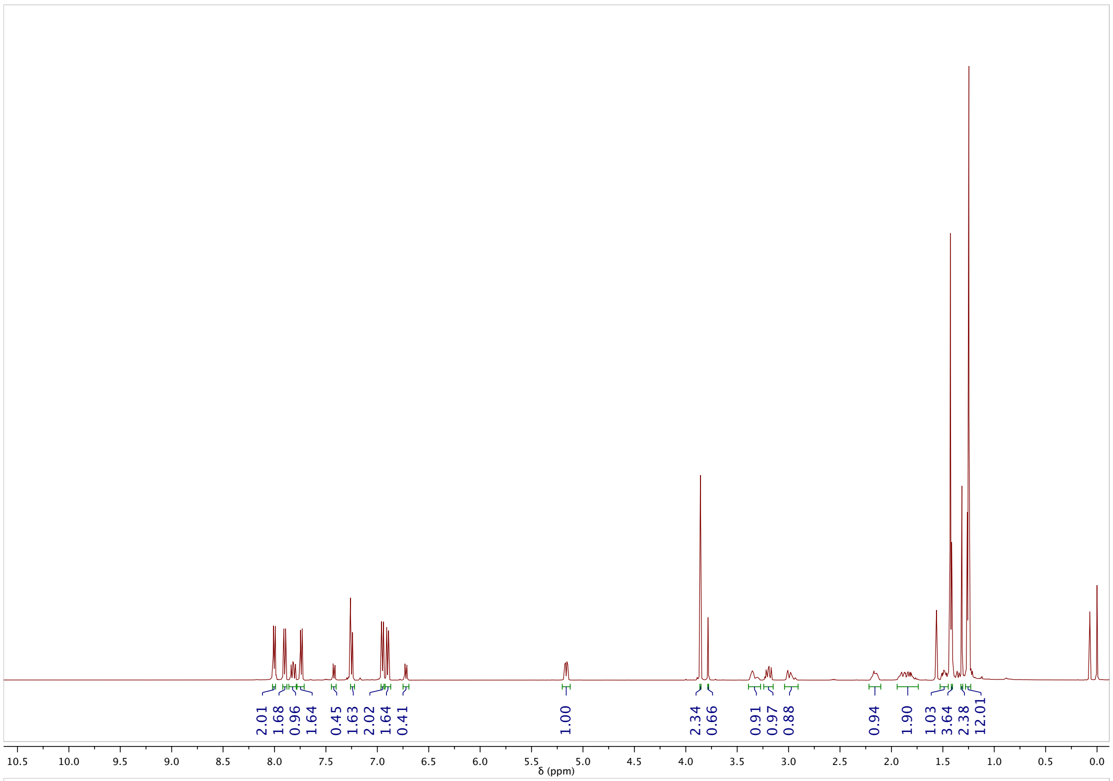

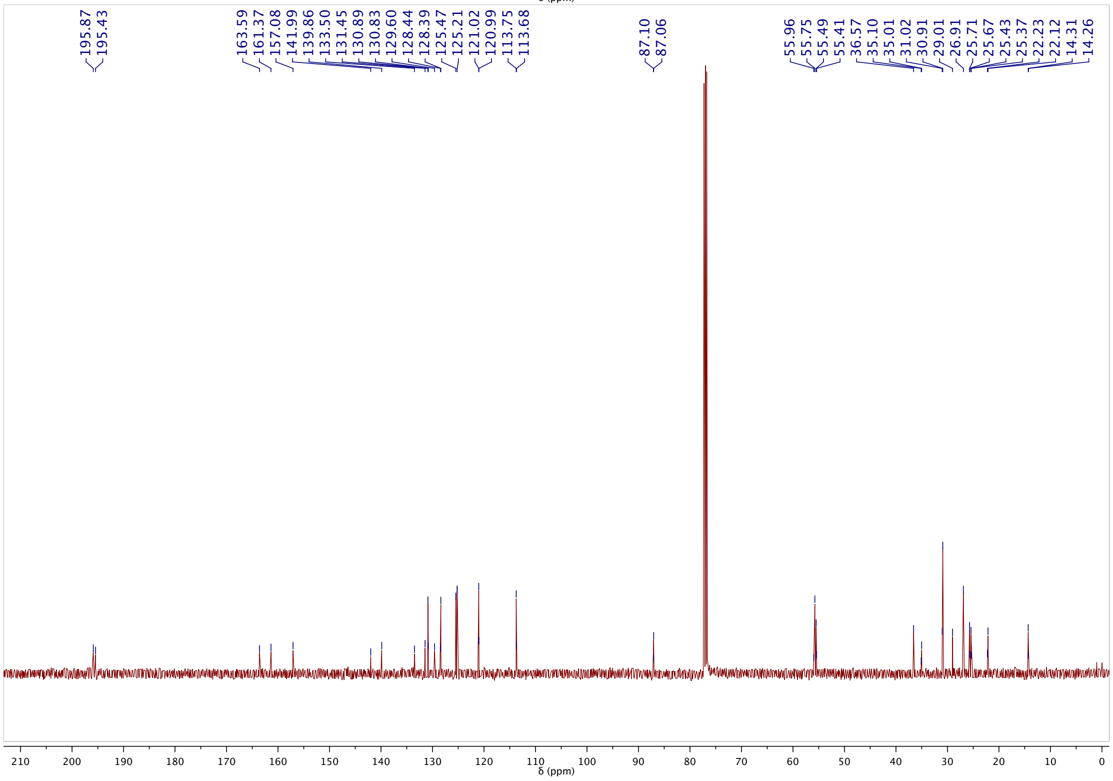

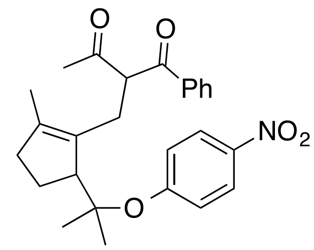

14i

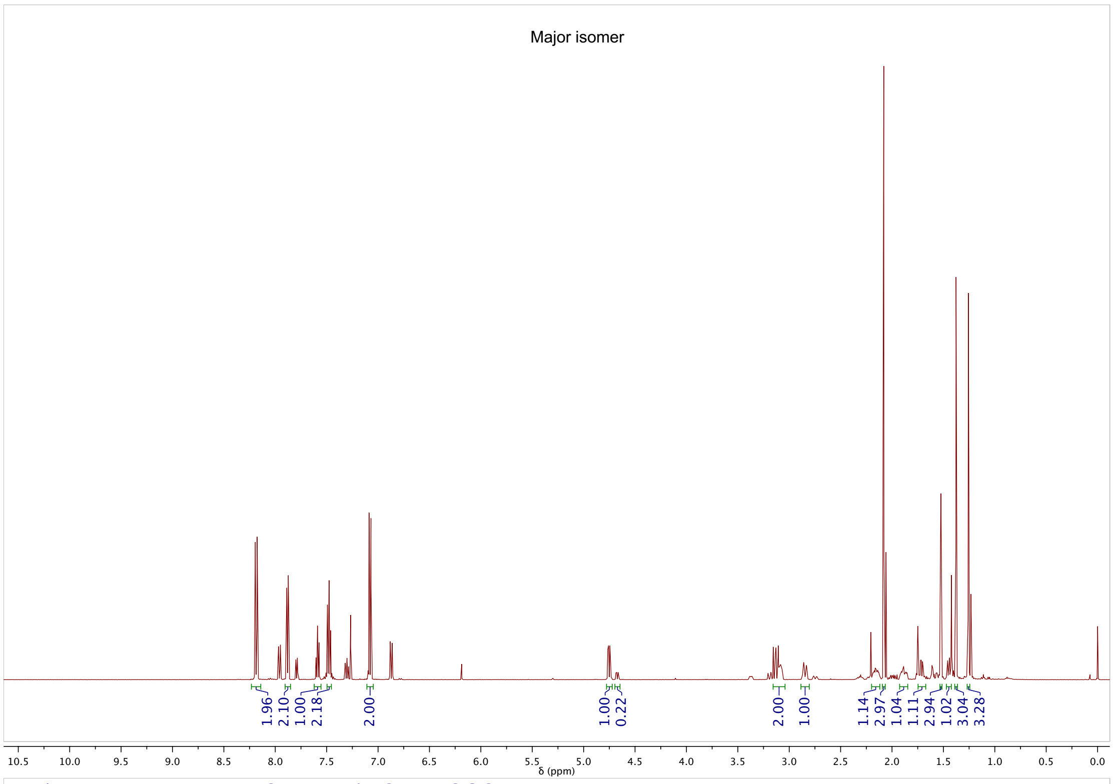

Major isomer
1.96
2.10
1.00
2.18
2.00
1.00
0.22
2.00
1.00
1.14
2.97
1.04
1.11
2.94
1.02
3.04
3.28
δ (ppm)

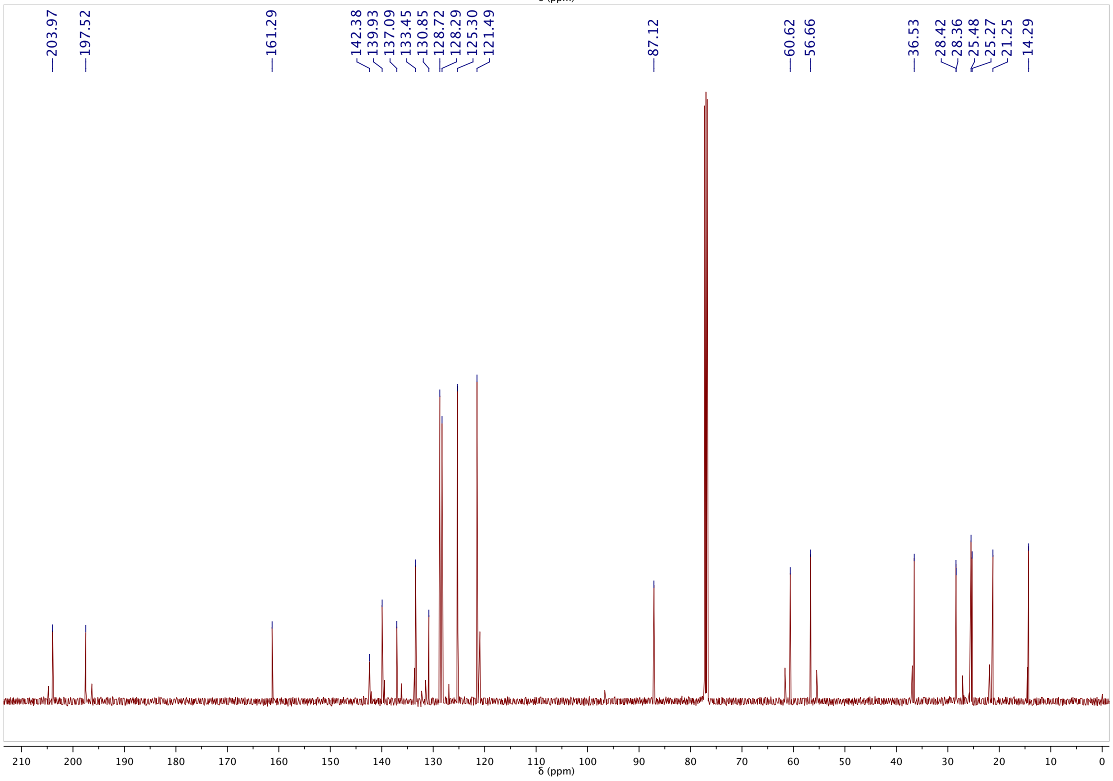

203.97
197.52
161.29
142.38
139.93
137.09
133.45
130.85
128.72
128.29
125.30
121.49
87.12
60.62
56.66
36.53
28.42
28.36
25.48
25.27
21.25
14.29
δ (ppm)

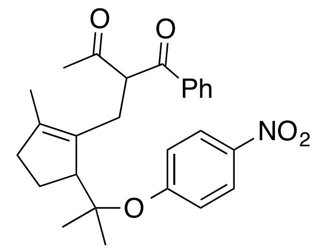
O
O
Ph
NO2
O

14i

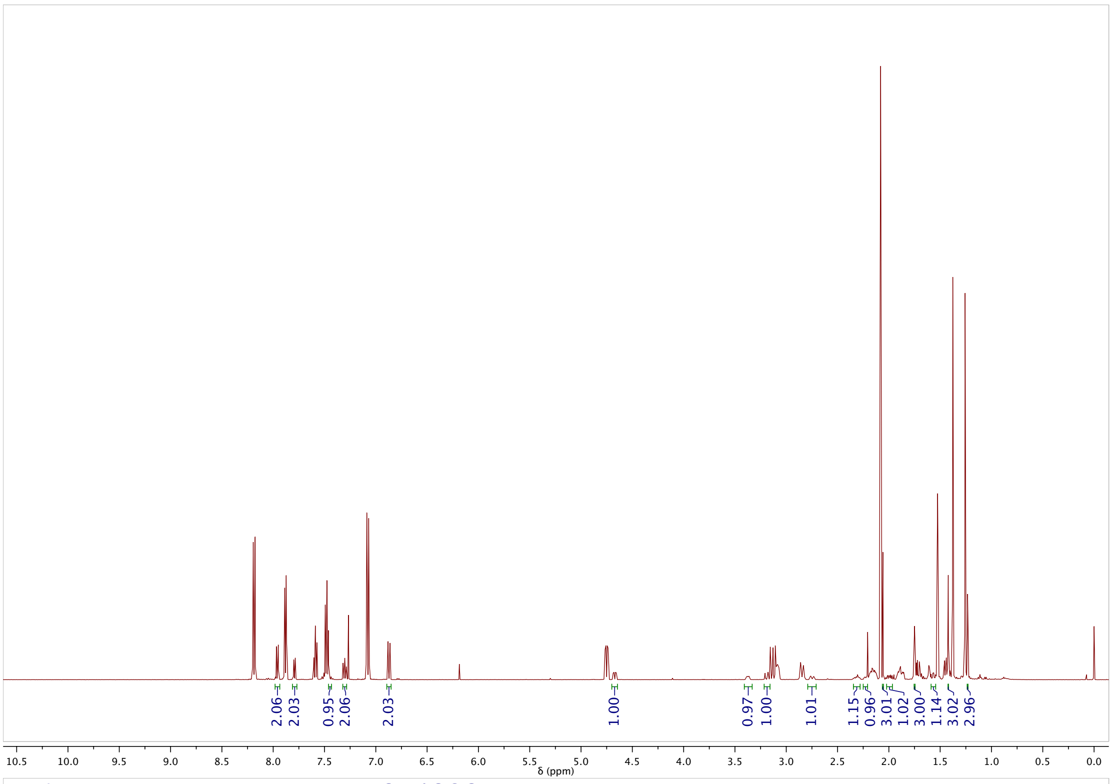
2.06
2.03
0.95
2.06
2.03
1.00
0.97
1.00
1.01
1.15
0.96
3.01
1.02
3.00
1.14
3.02
2.96
δ (ppm)

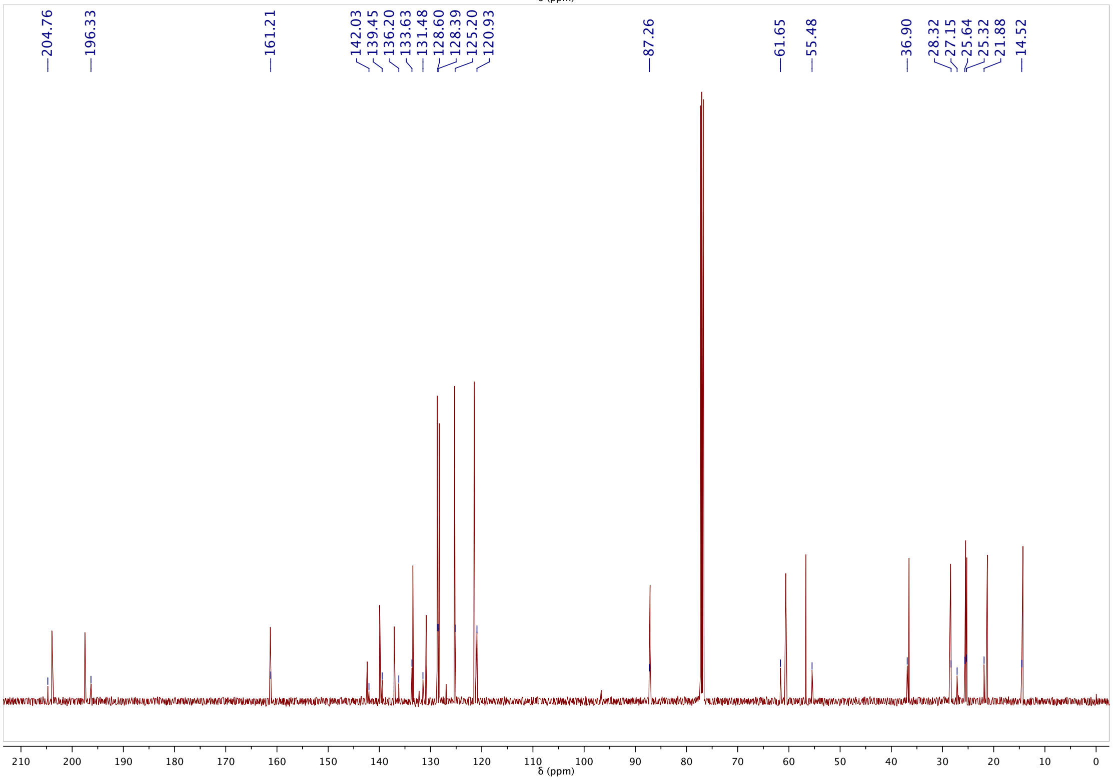
204.76
196.33
161.21
142.03
139.45
136.20
133.63
131.48
128.60
128.39
125.20
120.93
87.26
61.65
55.48
36.90
28.32
27.15
25.64
25.32
21.88
14.52
δ (ppm)

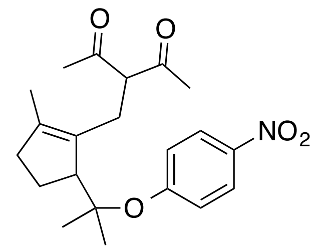

O
O
NO2
O

14j

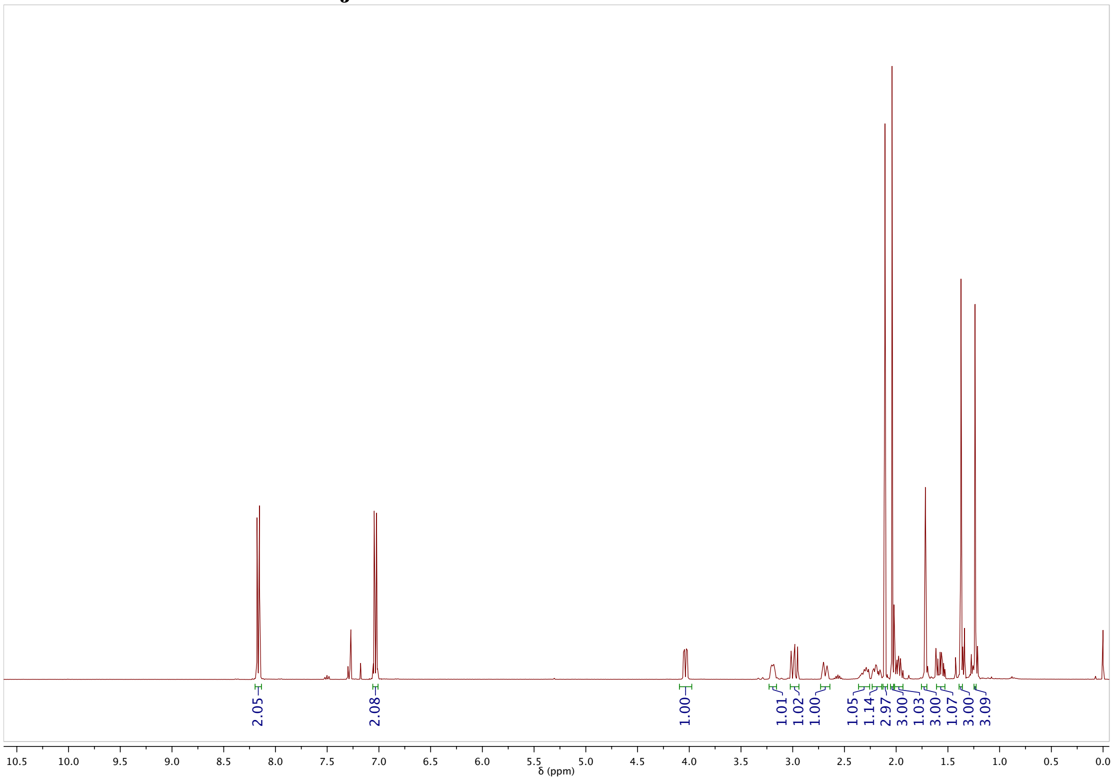

2.05
2.08
1.00
1.01
1.02
1.00
1.05
1.14
2.97
3.00
1.03
3.00
1.07
3.00
3.09
10.5
10.0
9.5
9.0
8.5
8.0
7.5
7.0
6.5
6.0
5.5
5.0
4.5
4.0
3.5
3.0
2.5
2.0
1.5
1.0
0.5
0.0
δ (ppm)

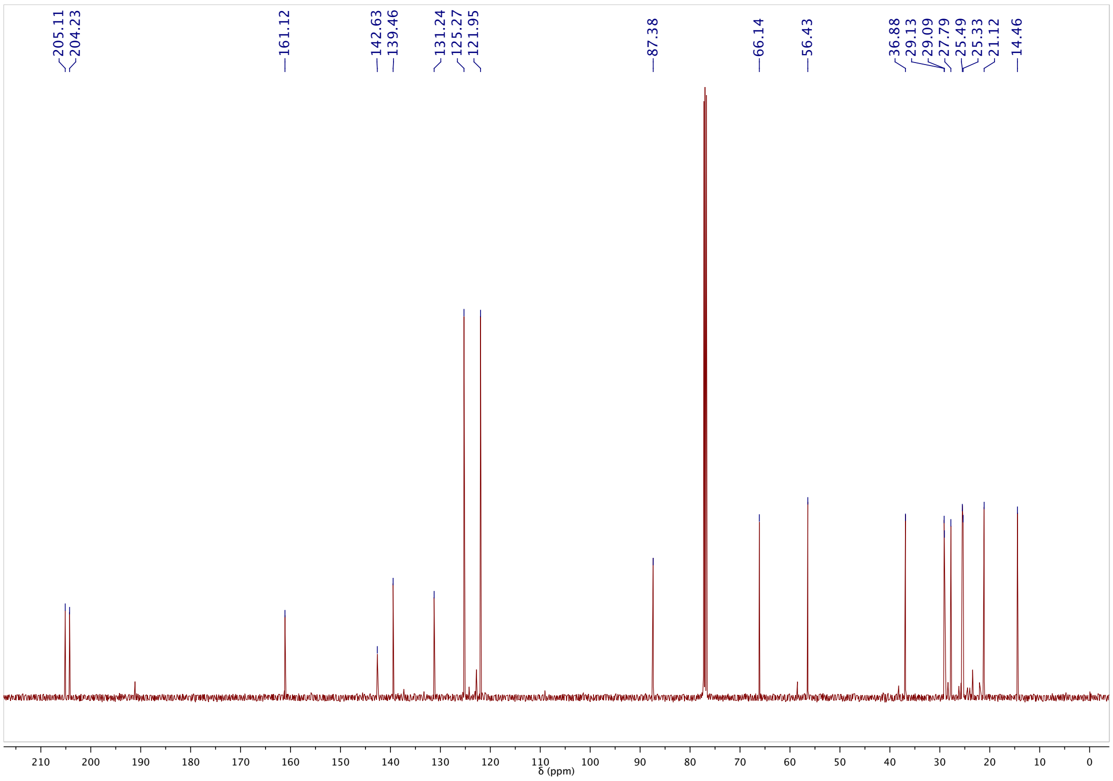

205.11
204.23
161.12
142.63
139.46
131.24
125.27
121.95
87.38
66.14
56.43
36.88
29.13
29.09
27.79
25.49
25.33
21.12
14.46
210
200
190
180
170
160
150
140
130
120
110
100
90
80
70
60
50
40
30
20
10
0
δ (ppm)

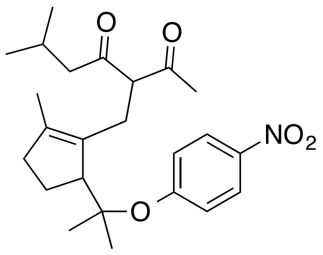

14k

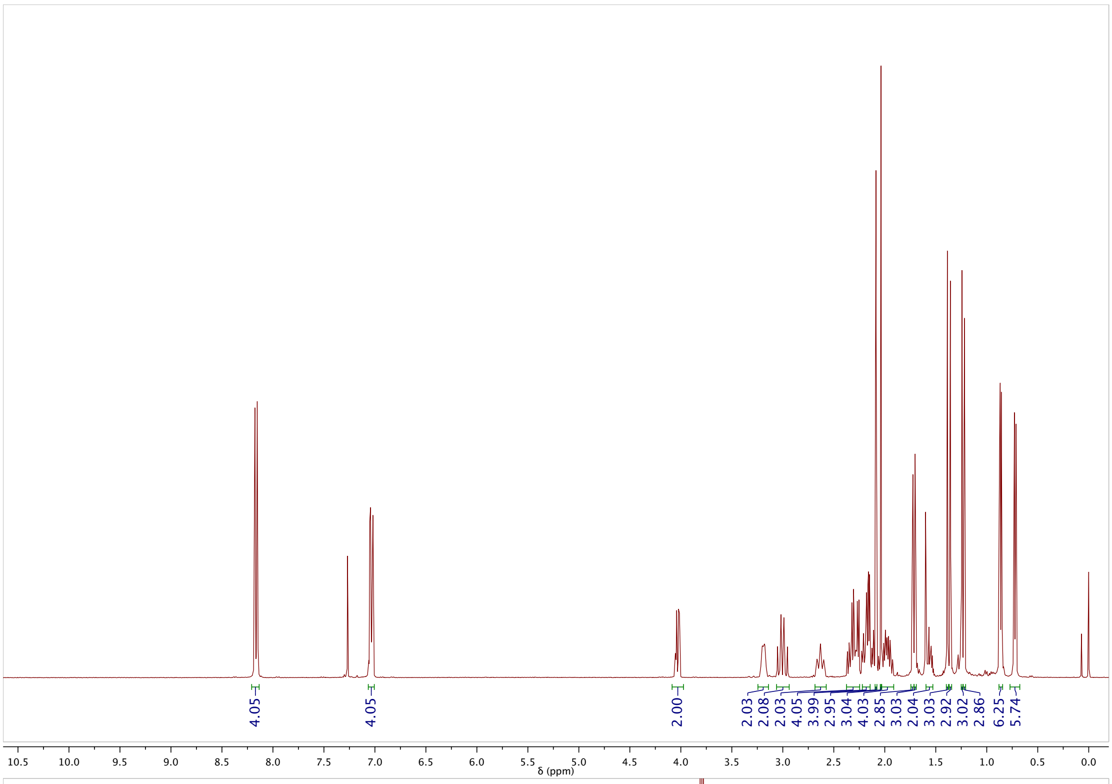

4.05
4.05
2.00
2.03
2.08
2.03
4.05
3.99
2.95
3.04
4.03
2.85
3.03
2.04
3.03
2.92
3.02
2.86
6.25
5.74
δ (ppm)

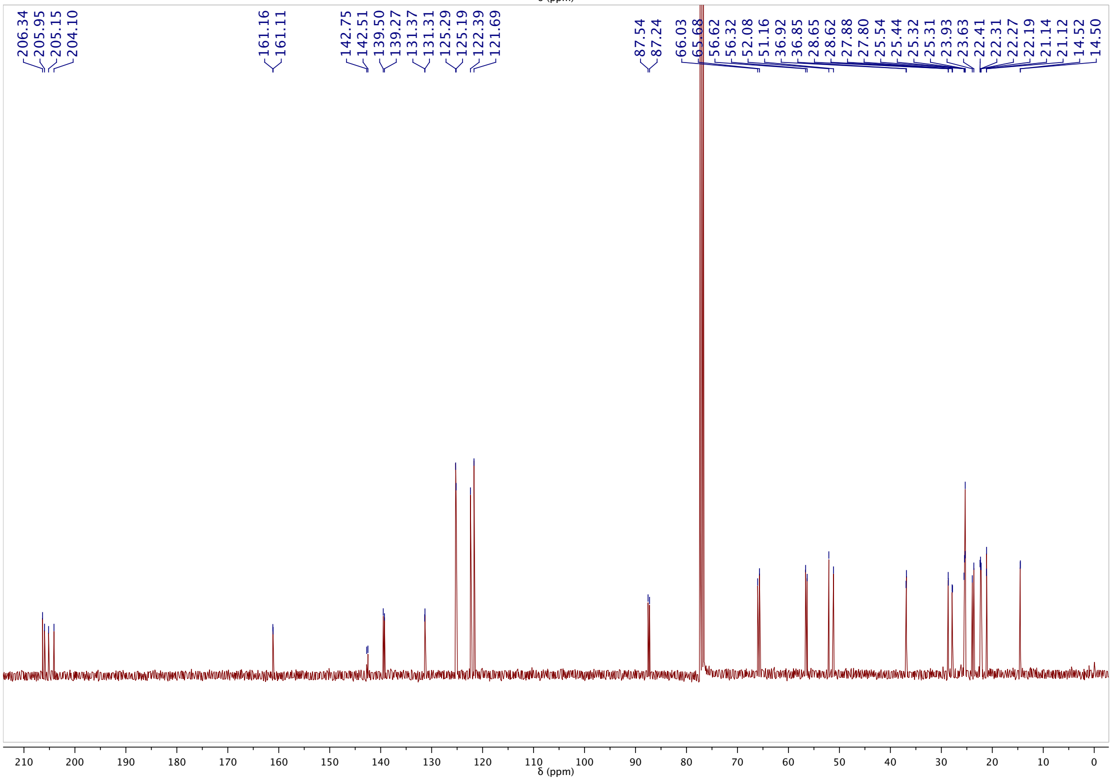

206.34
205.95
205.15
204.10
161.16
161.11
142.75
142.51
139.50
139.27
131.37
131.31
125.29
125.19
122.39
121.69
87.54
87.24
66.03
65.68
56.62
56.32
52.08
51.16
36.92
36.85
28.65
28.62
27.88
27.80
25.54
25.44
25.32
25.31
23.93
23.63
22.41
22.31
22.27
22.19
21.14
21.12
14.52
14.50
δ (ppm)

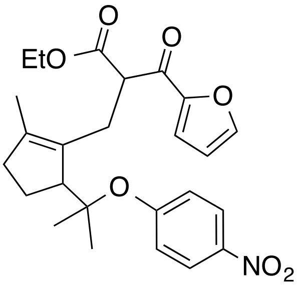
EtO
O
O
O
O
NO2

14n

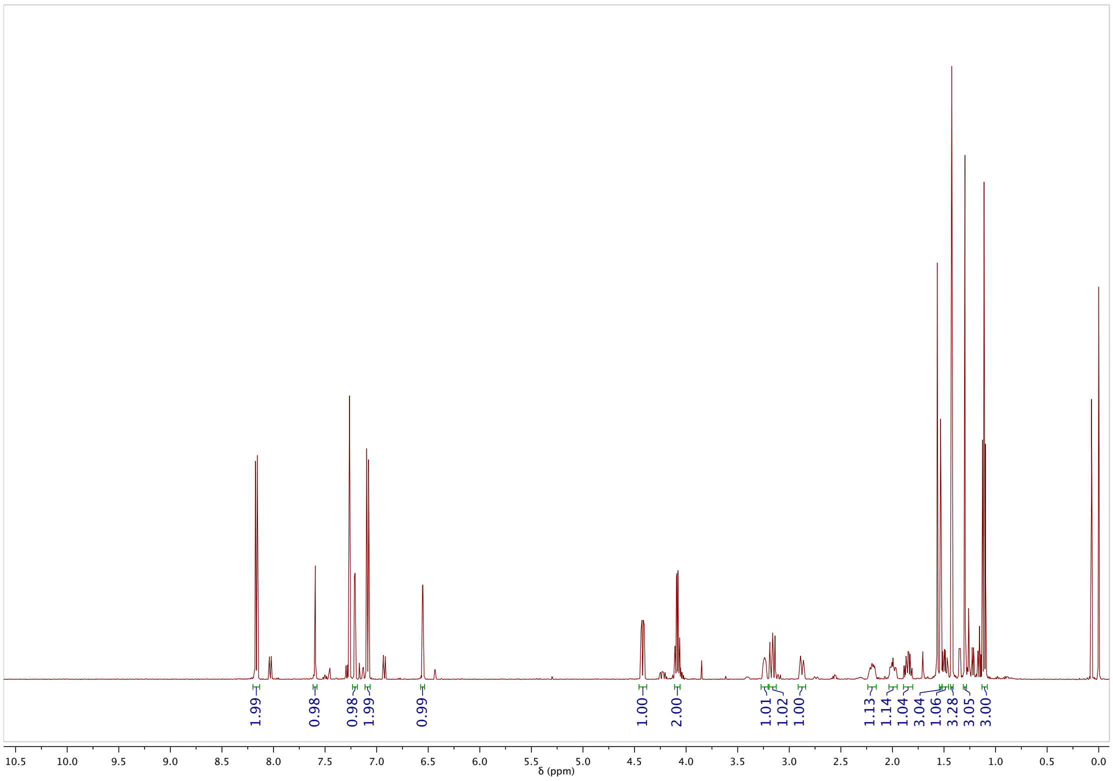
1.99
0.98
0.98
1.99
0.99
1.00
2.00
1.01
1.02
1.00
1.13
1.14
1.04
3.04
1.06
3.28
3.05
3.00
δ (ppm)

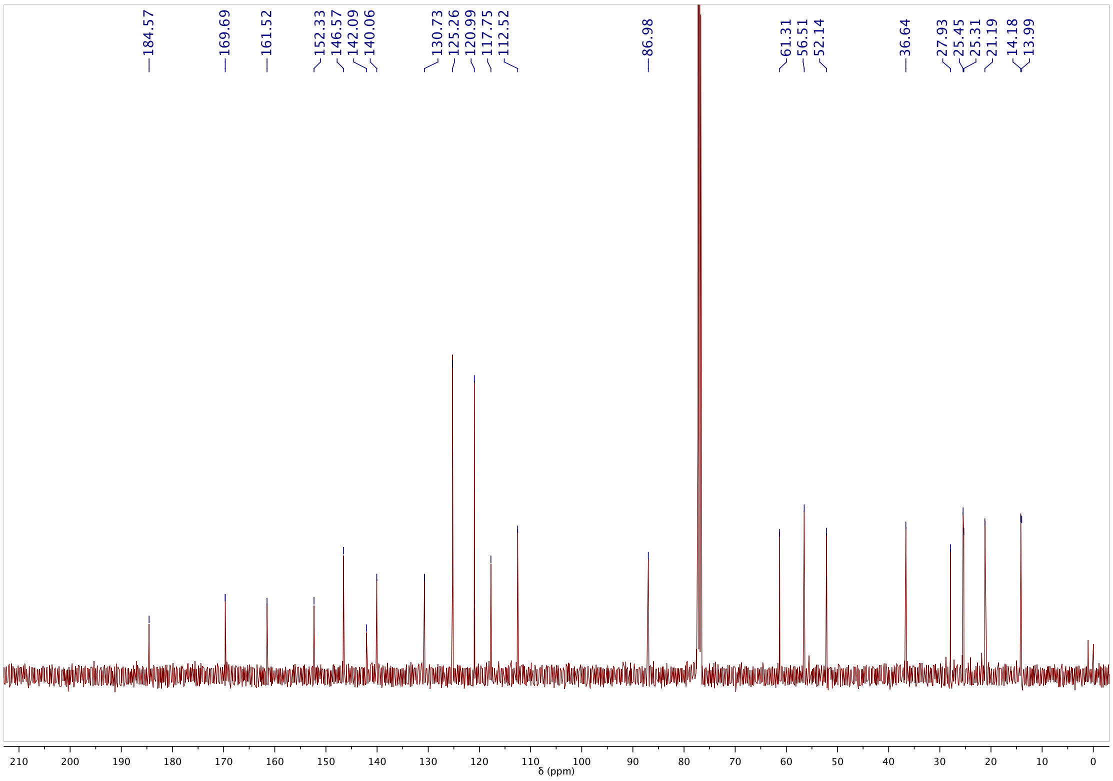
184.57
169.69
161.52
152.33
146.57
142.09
140.06
130.73
125.26
120.99
117.75
112.52
86.98
61.31
56.51
52.14
36.64
27.93
25.45
25.31
21.19
14.18
13.99
δ (ppm)

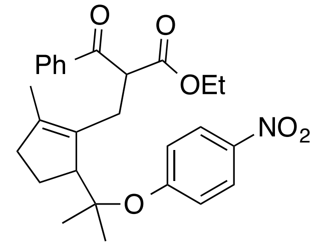

14m

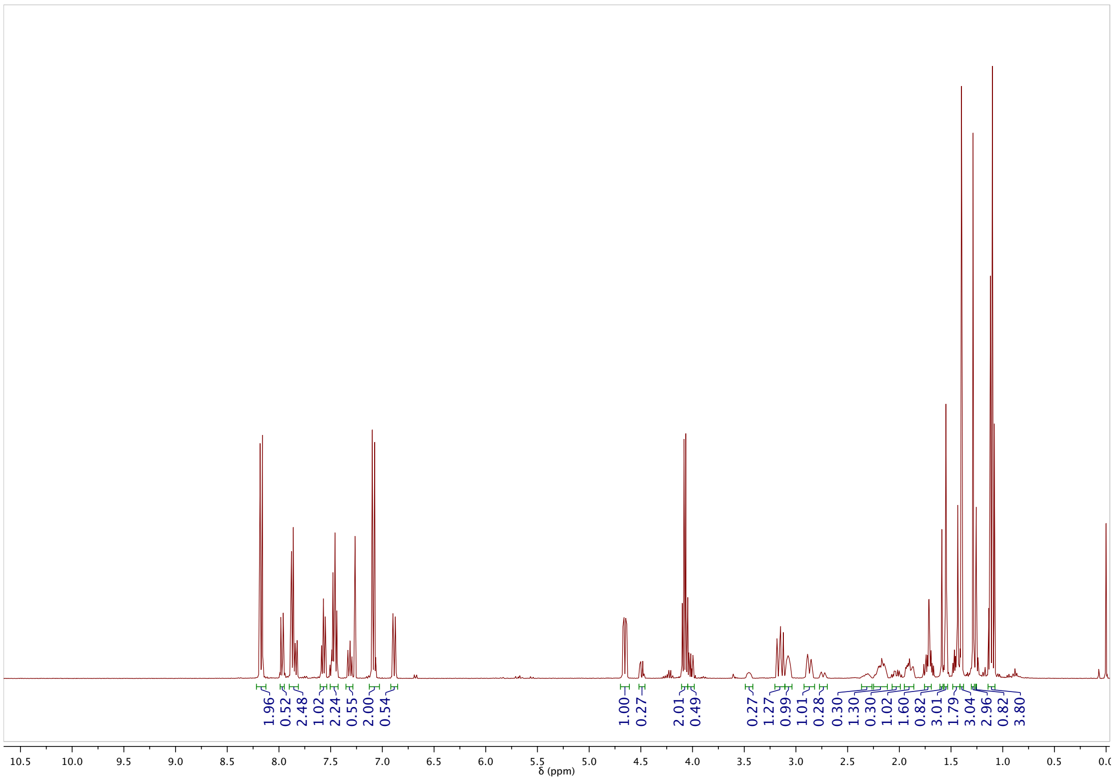

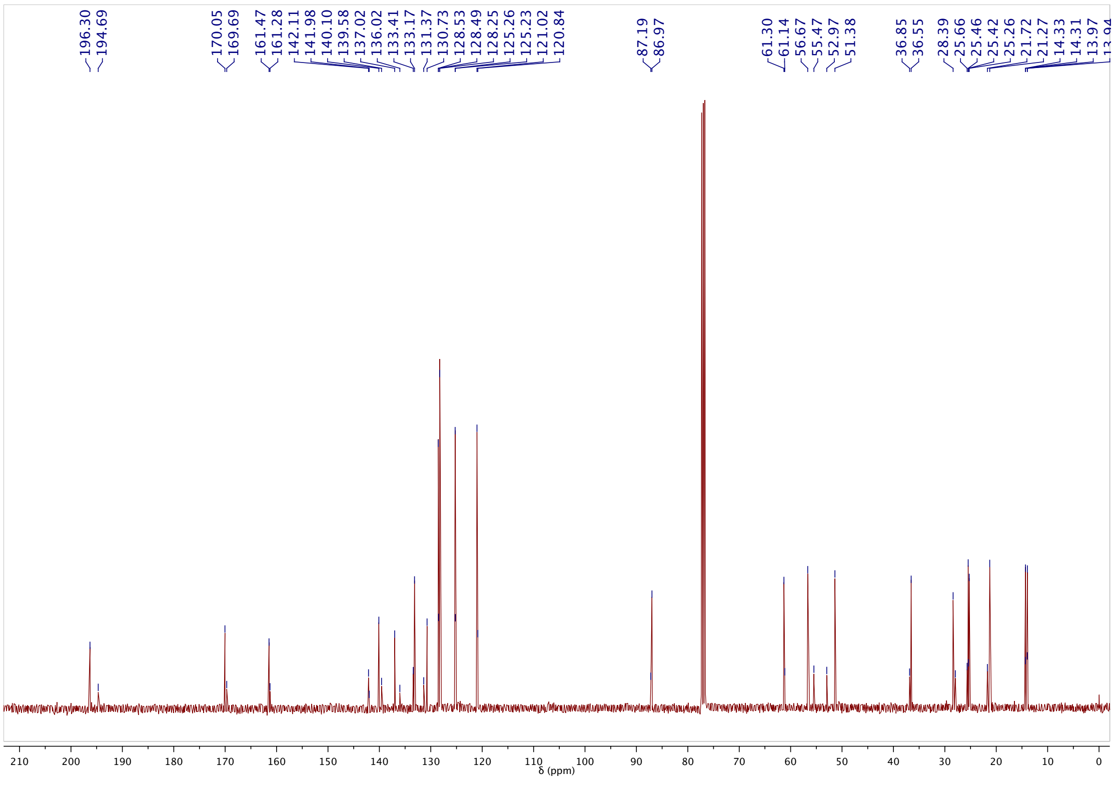

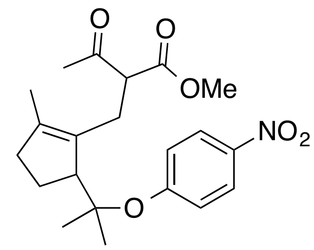
O
O
OMe
NO2
O

14l

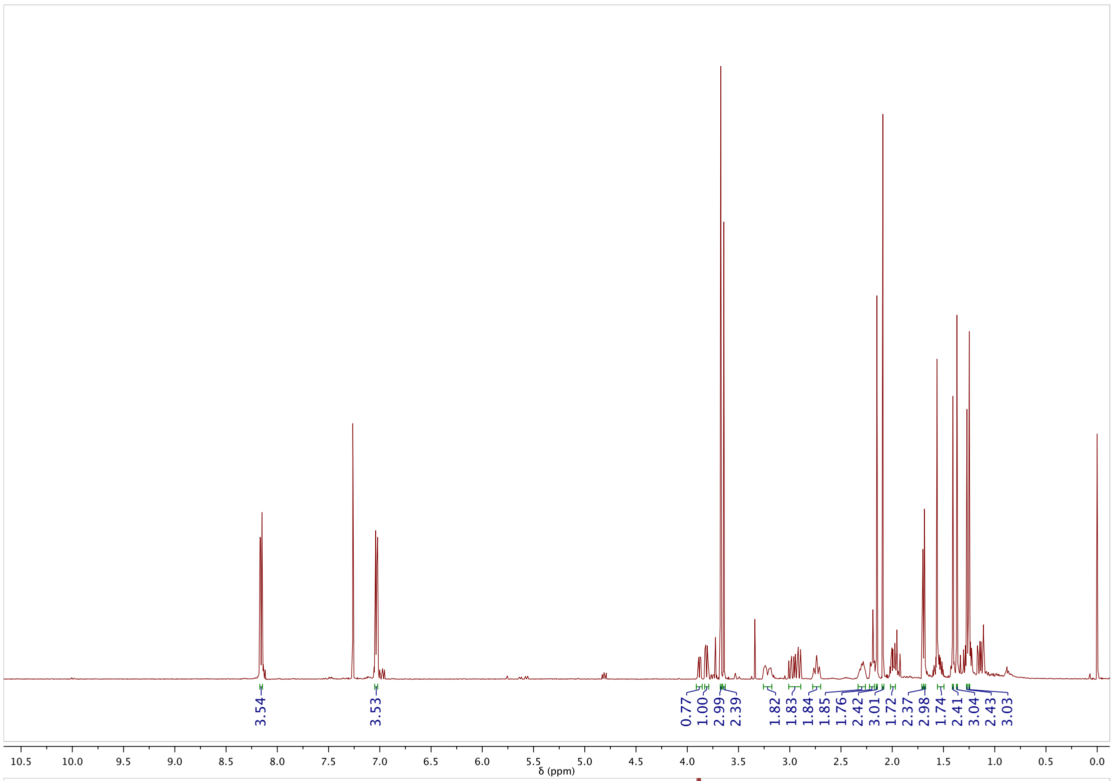
3.54
3.53
0.77
1.00
2.99
2.39
1.82
1.83
1.84
1.85
1.76
2.42
3.01
1.72
2.37
2.98
1.74
2.41
3.04
2.43
3.03
δ (ppm)

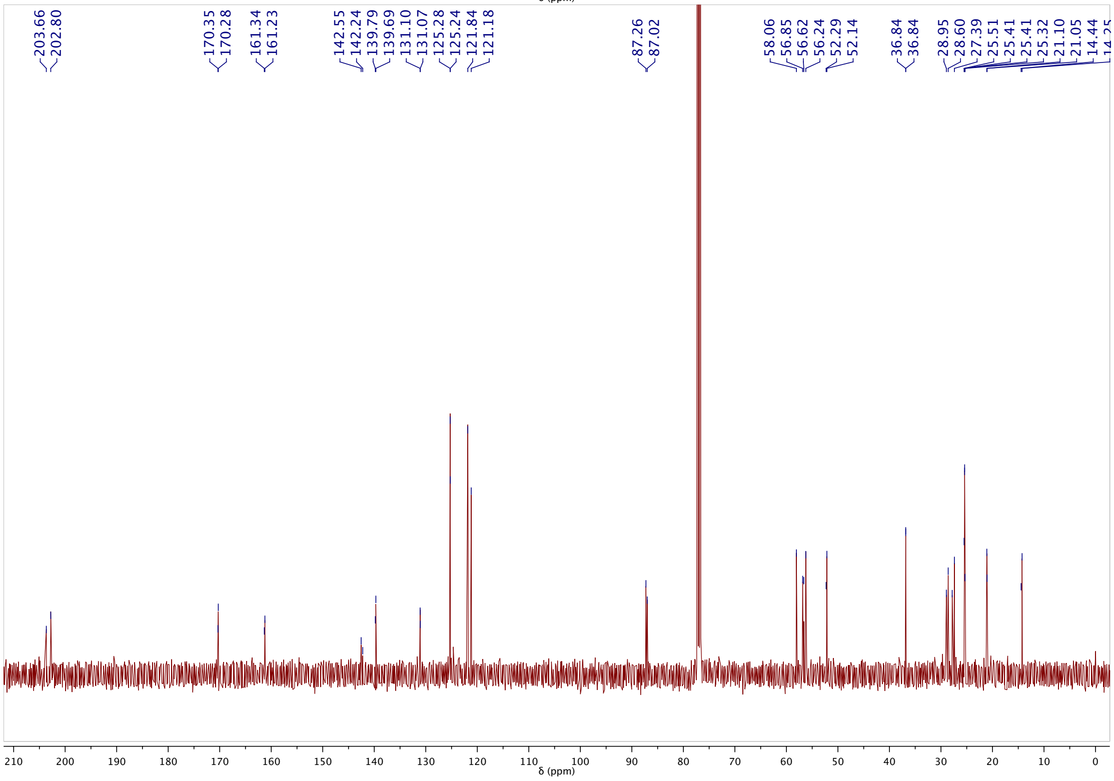
203.66
202.80
170.35
170.28
161.34
161.23
142.55
142.24
139.79
139.69
131.10
131.07
125.28
125.24
121.84
121.18
87.26
87.02
58.06
56.85
56.62
56.24
52.29
52.14
36.84
36.84
28.95
28.60
27.39
25.51
25.41
25.41
25.32
21.10
21.05
14.44
δ (ppm)

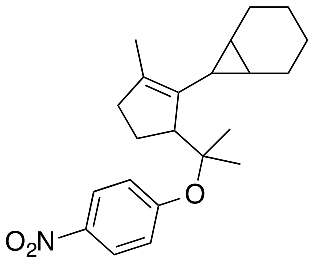

15a

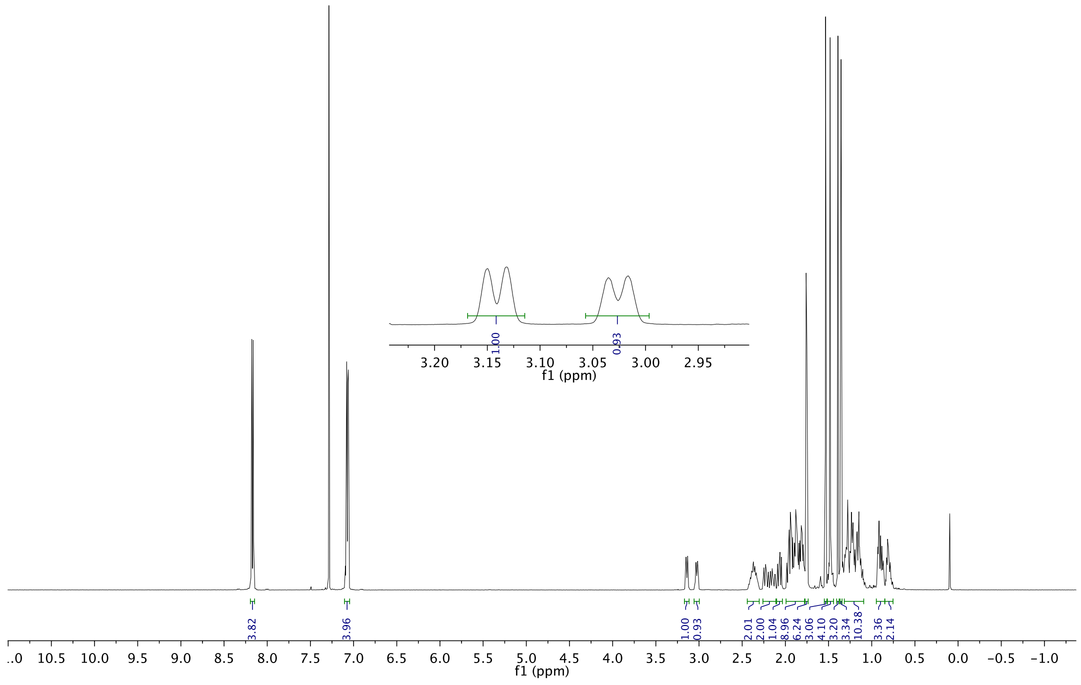

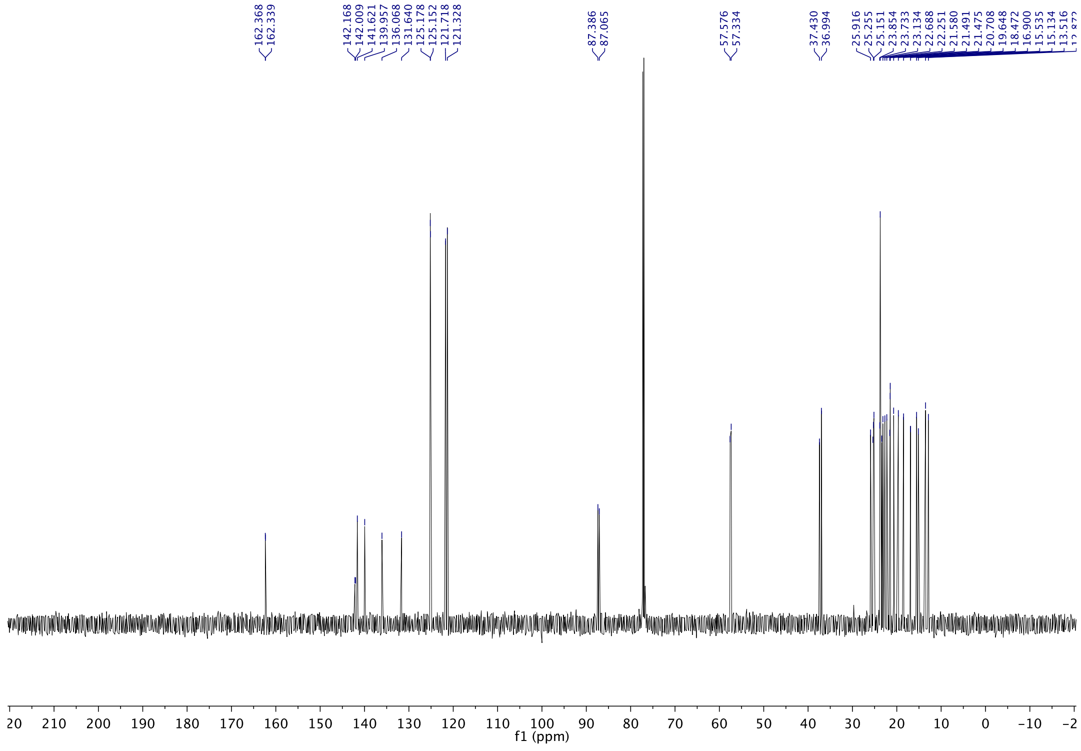

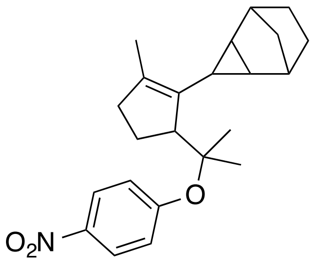

15b

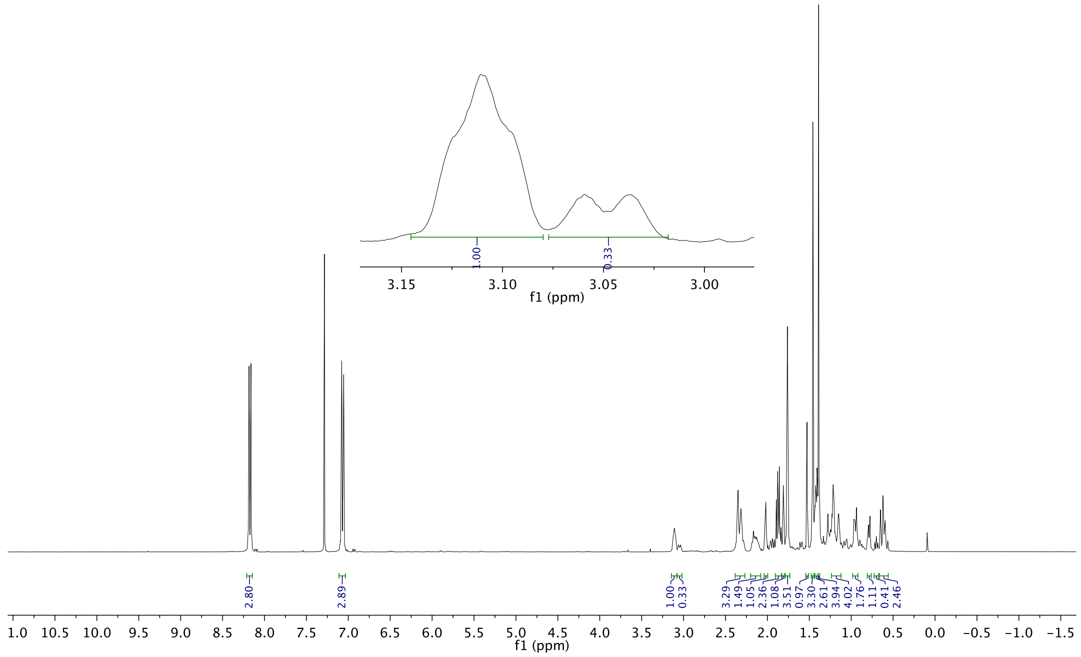

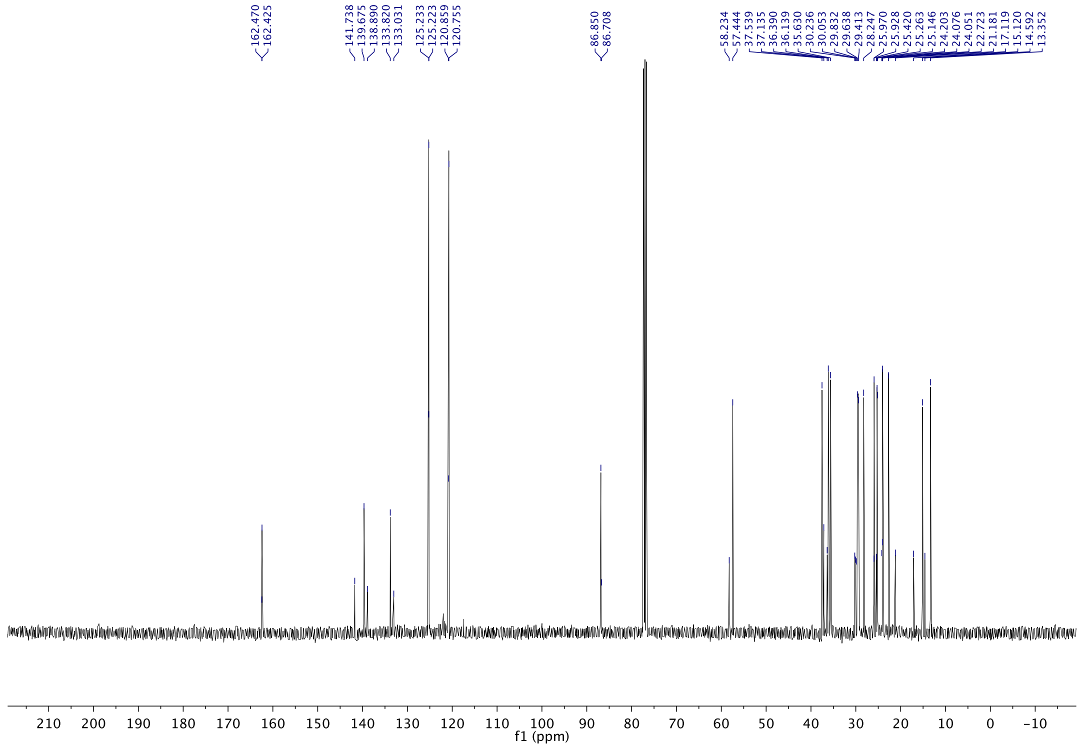

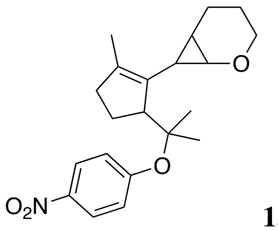

15c

Fraction enriched in the major isomer; only the values for the peaks of the major isomer are shown.

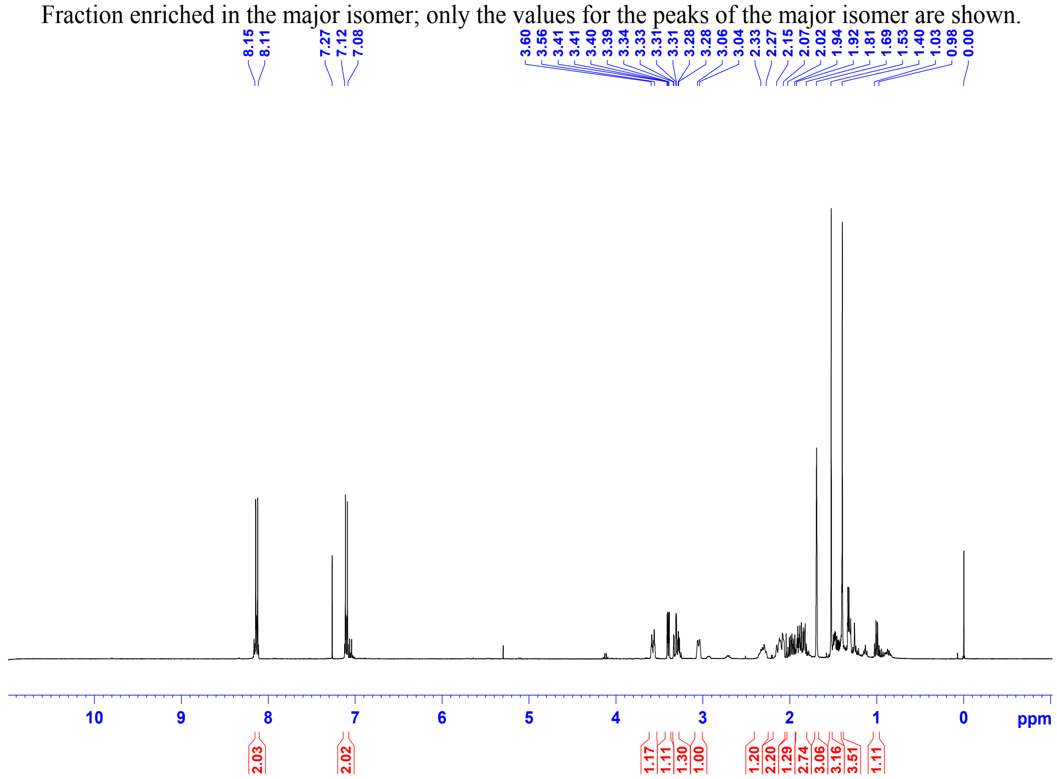

Fraction enriched in the major isomer; only the values for the peaks of the major isomer are shown.

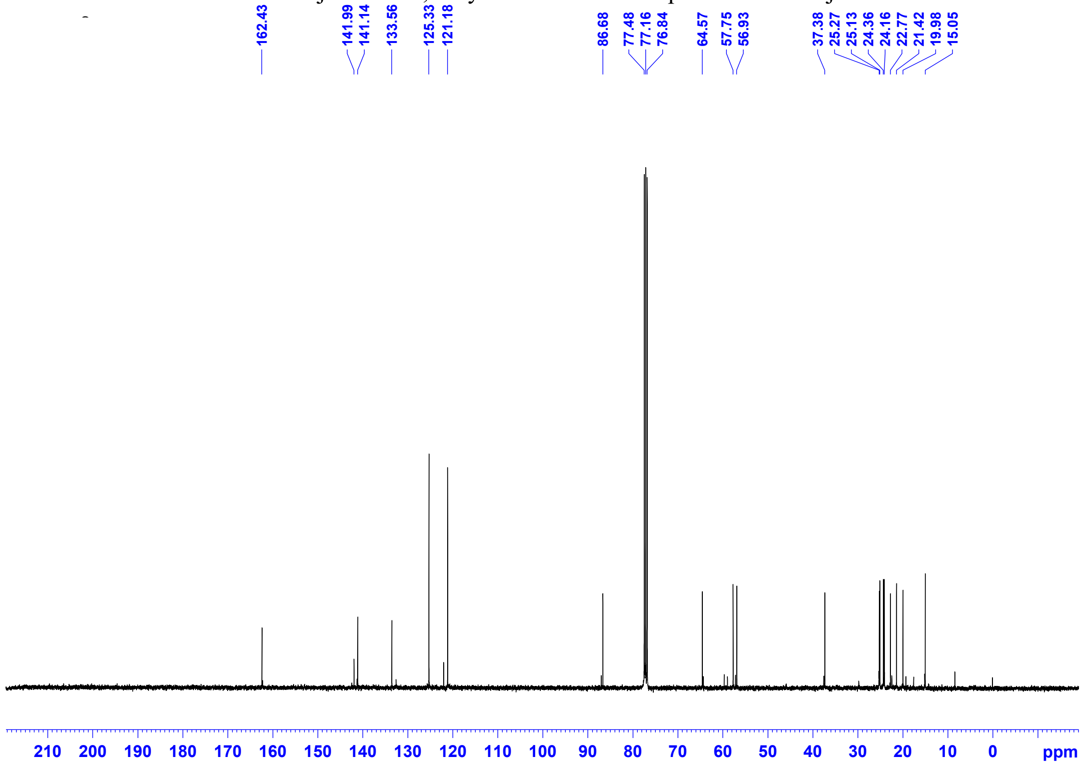

Fraction enriched in the minor isomer; only selected values for the peaks of the minor isomer are shown.

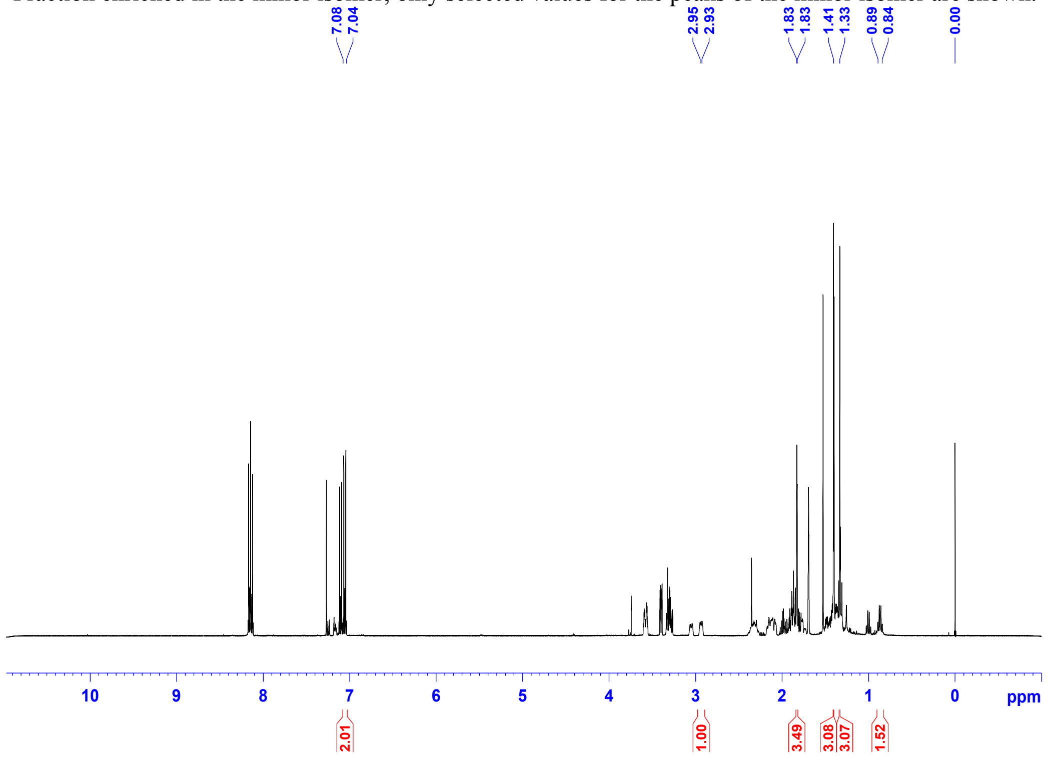

Fraction enriched in the minor isomer; peaks values are shown for both isomers.

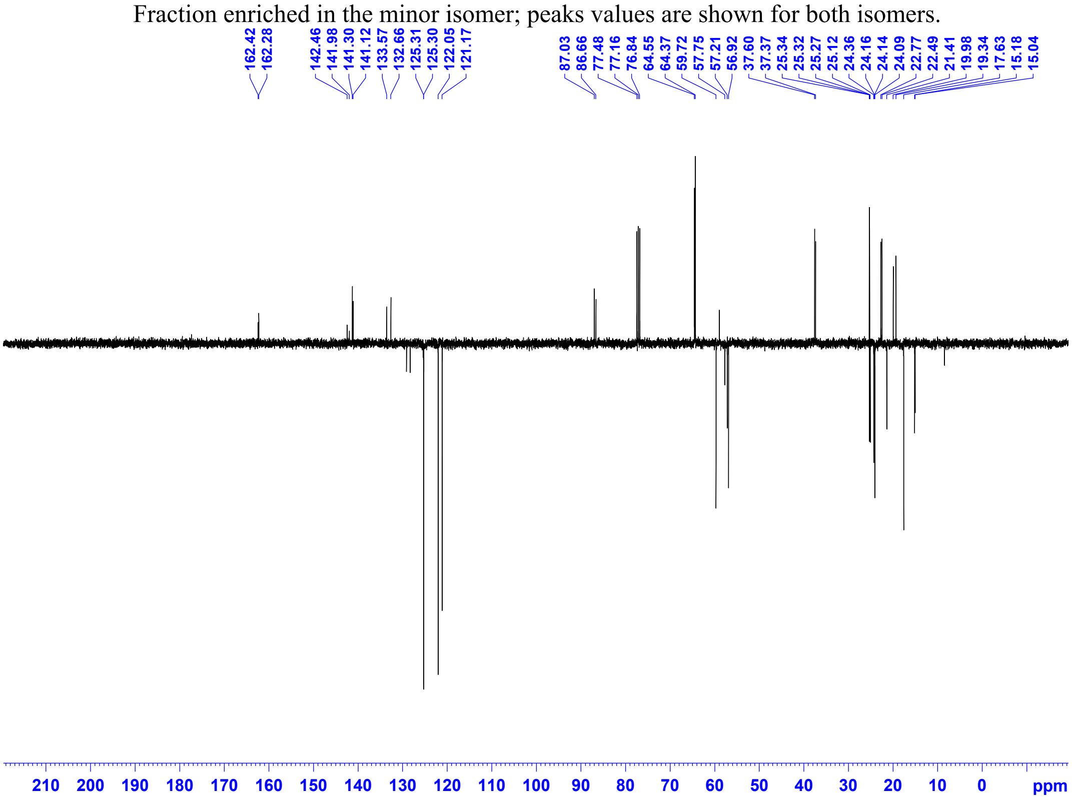

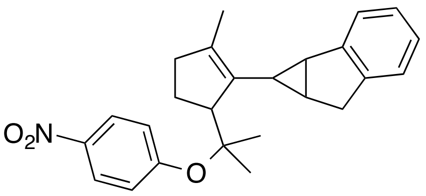

15d

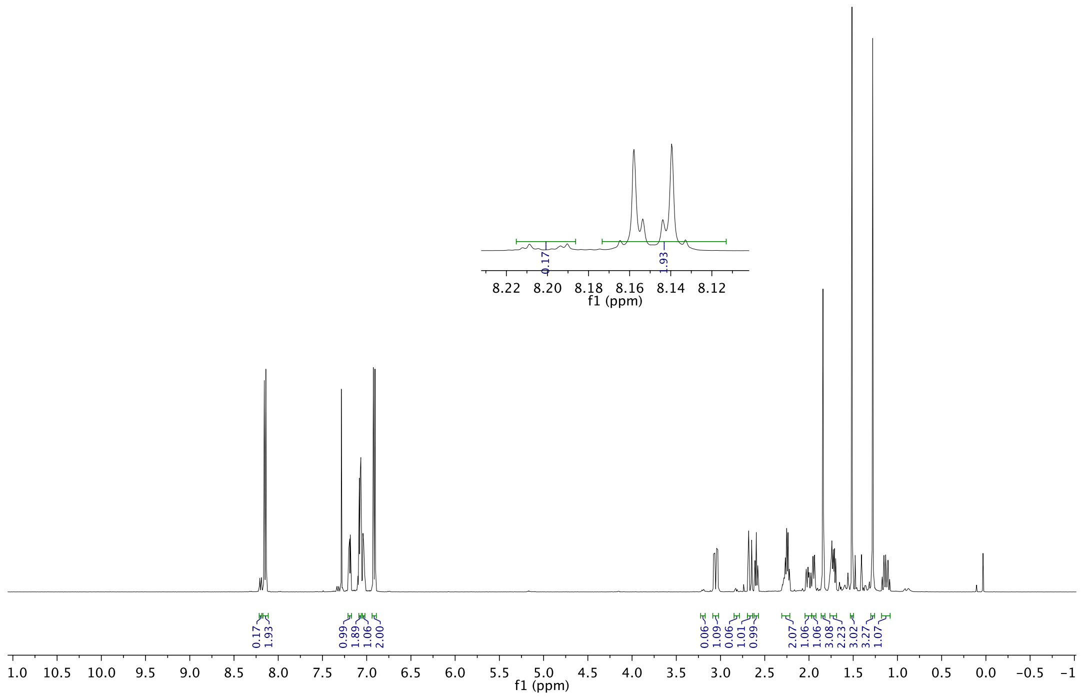

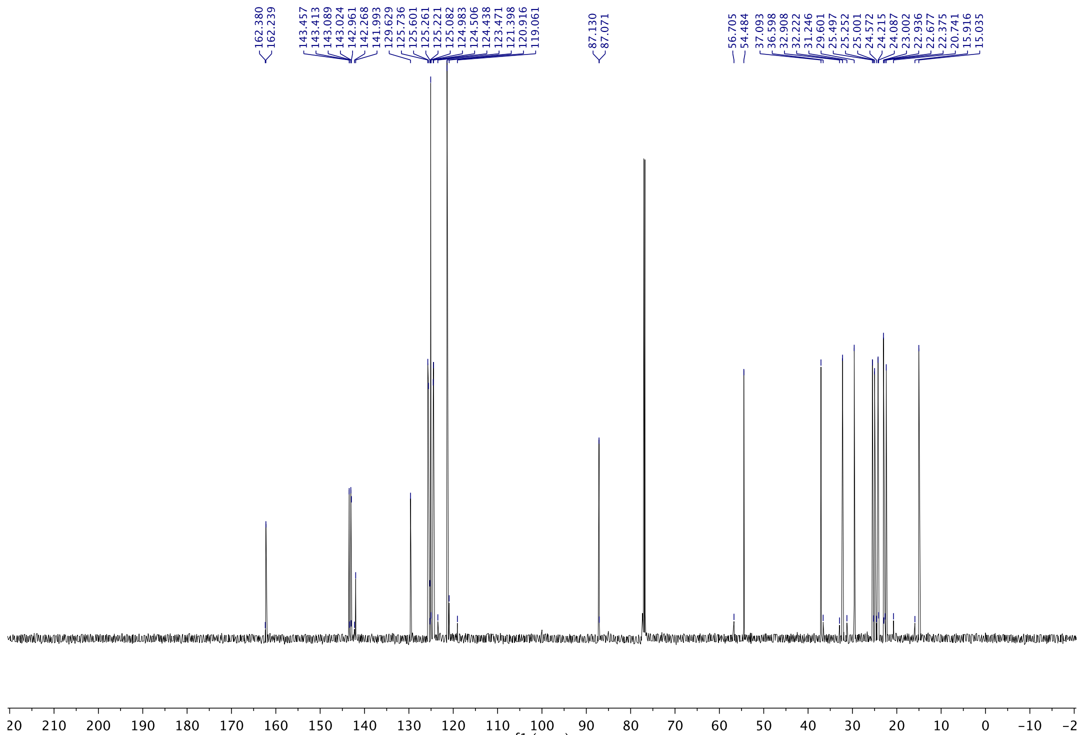

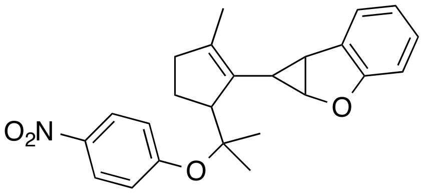

15e

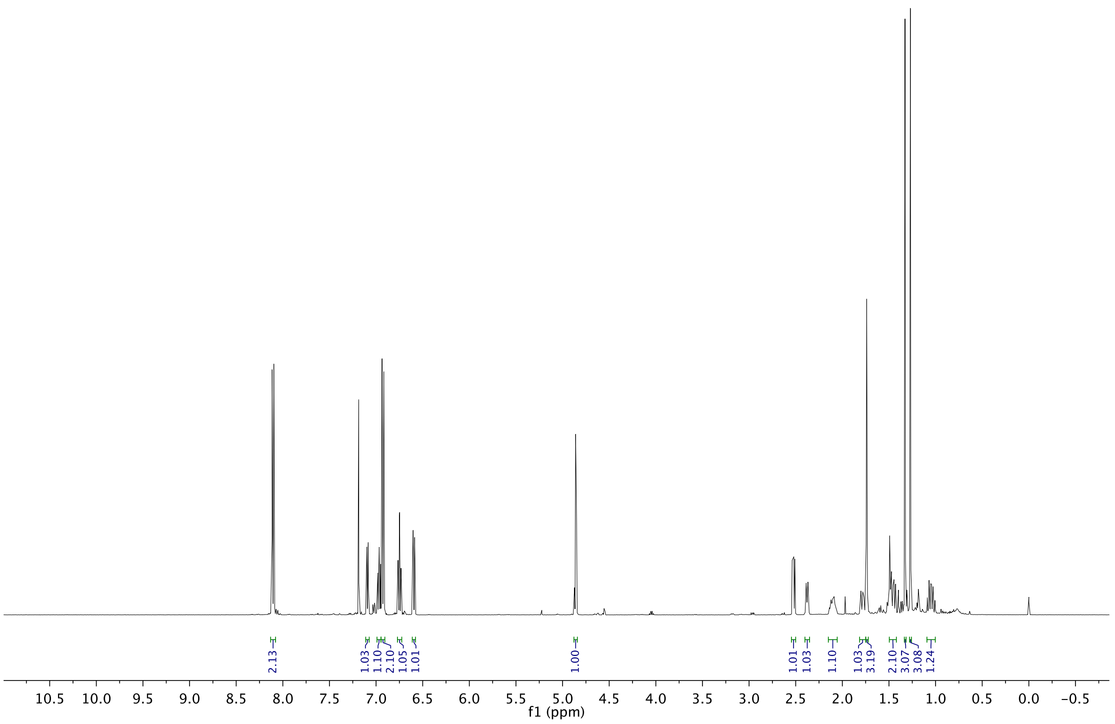

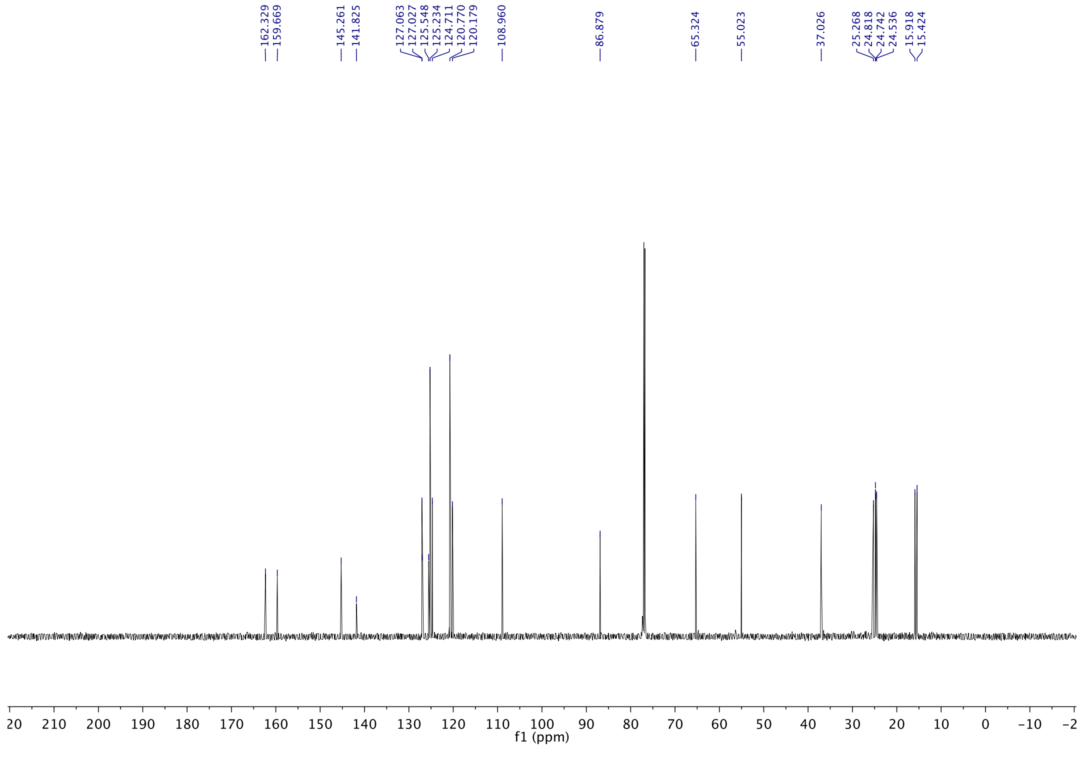

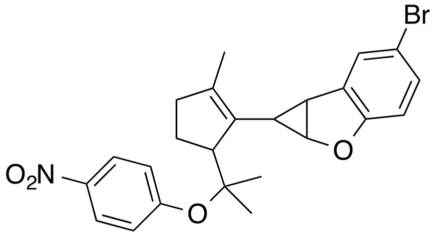

15f

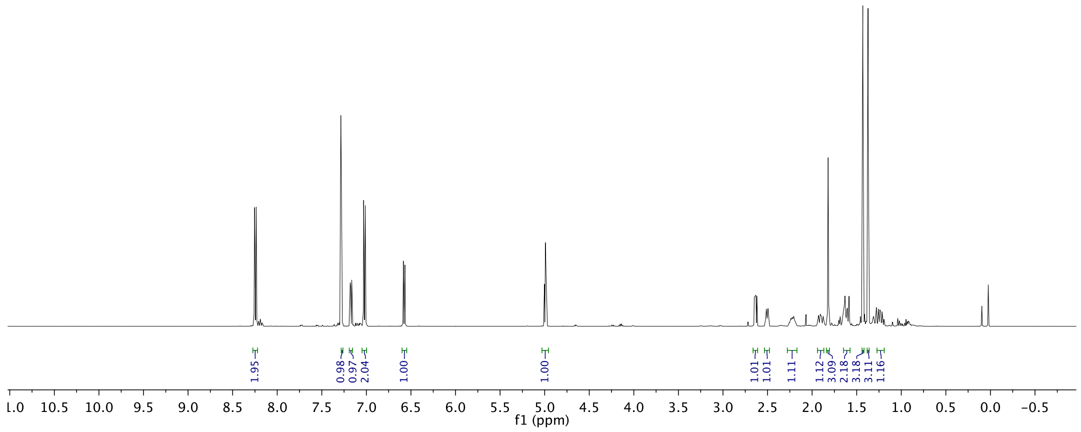

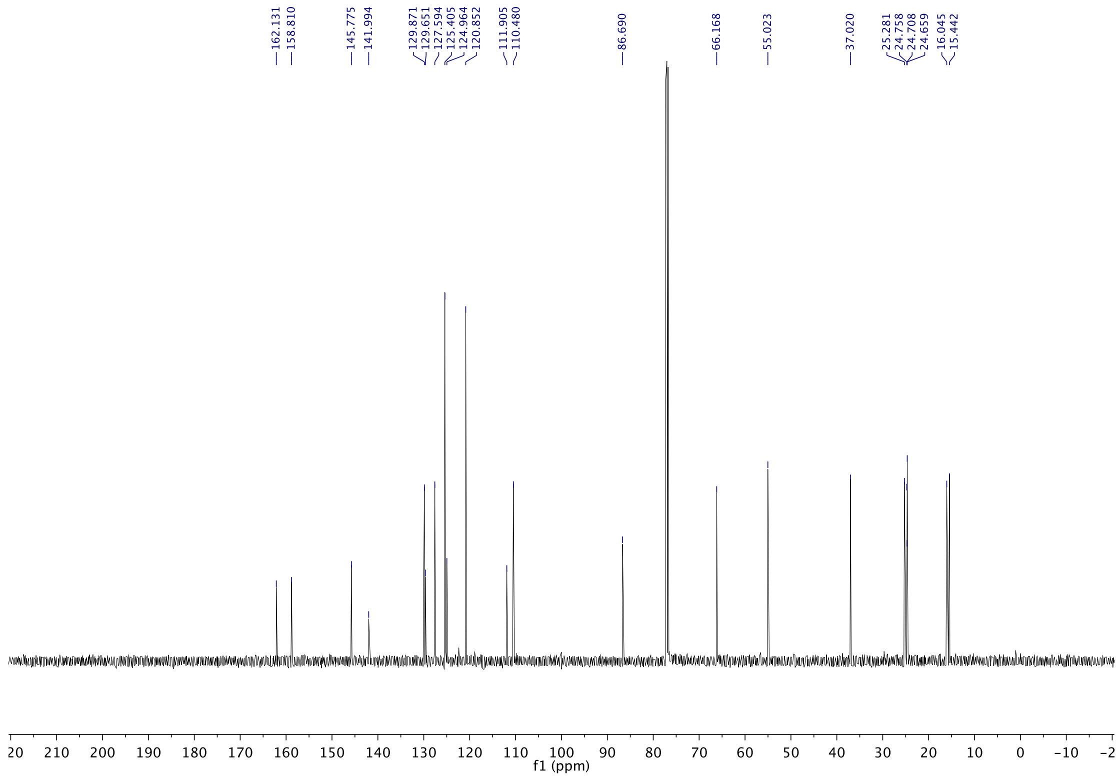

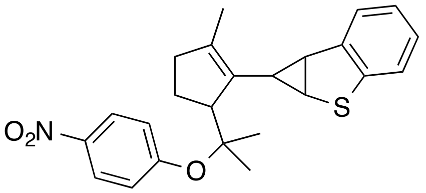

O2N
O
S

15g

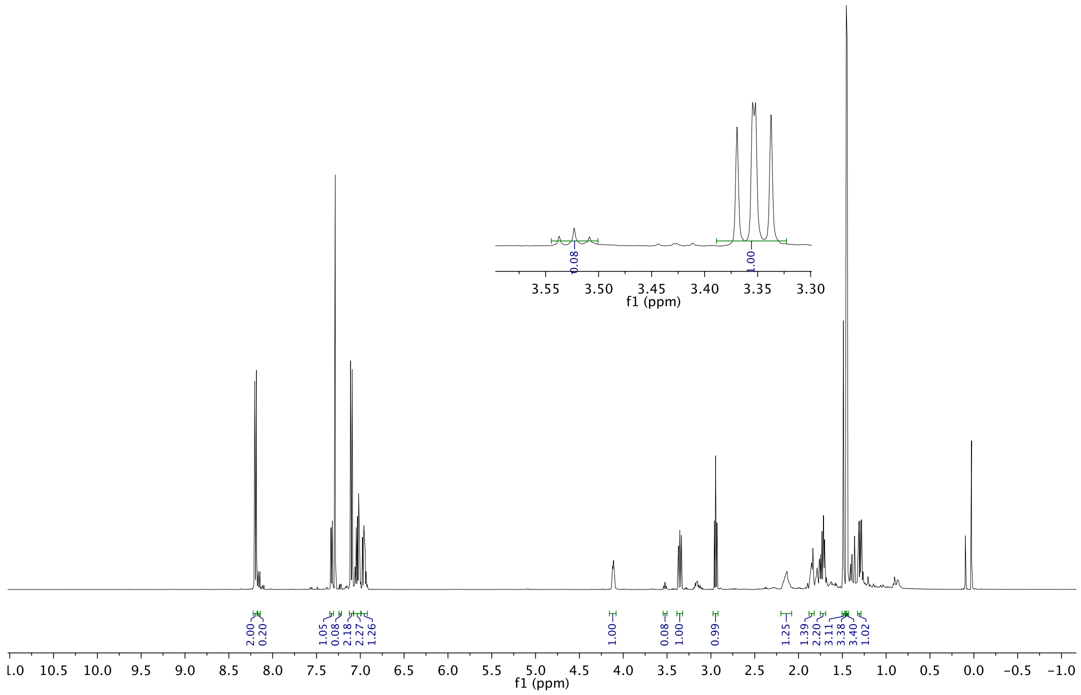

f1 (ppm)

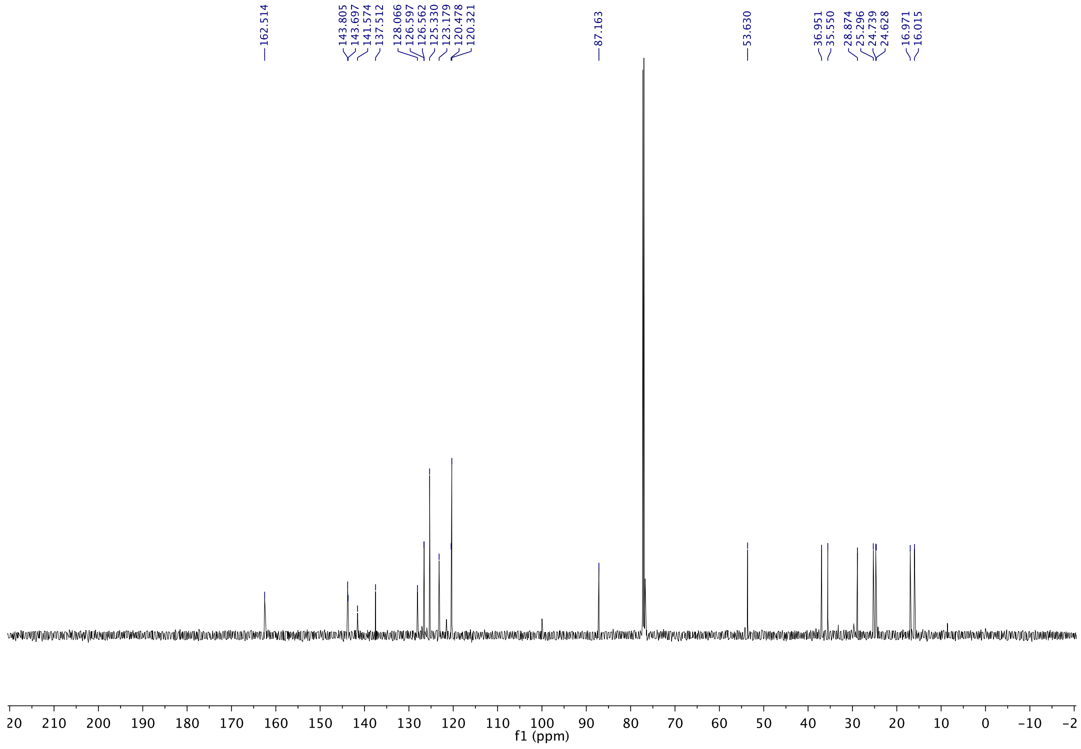

162.514
143.805
143.697
141.574
137.512
128.066
126.597
126.562
125.330
123.179
120.478
120.321
87.163
53.630
36.951
35.550
28.874
25.296
24.739
24.628
16.971
16.015
f1 (ppm)

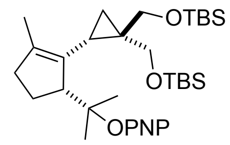
OTBS
OTBS
OPNP

(±)-6a

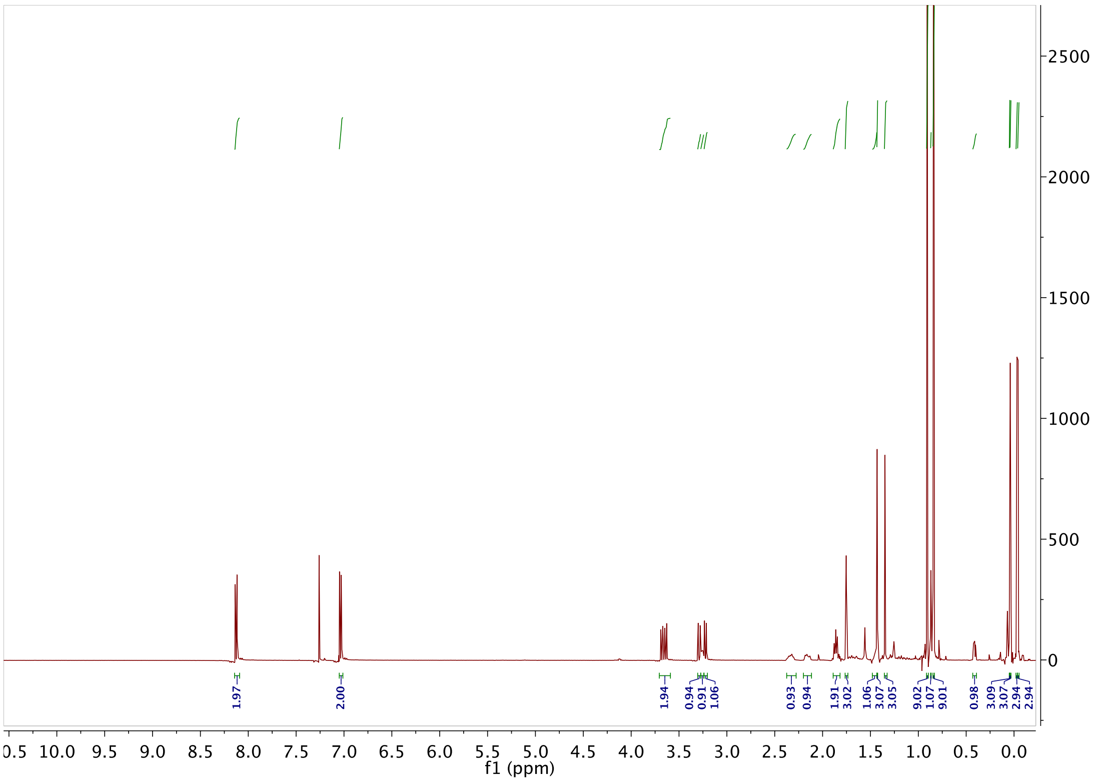
f1 (ppm)

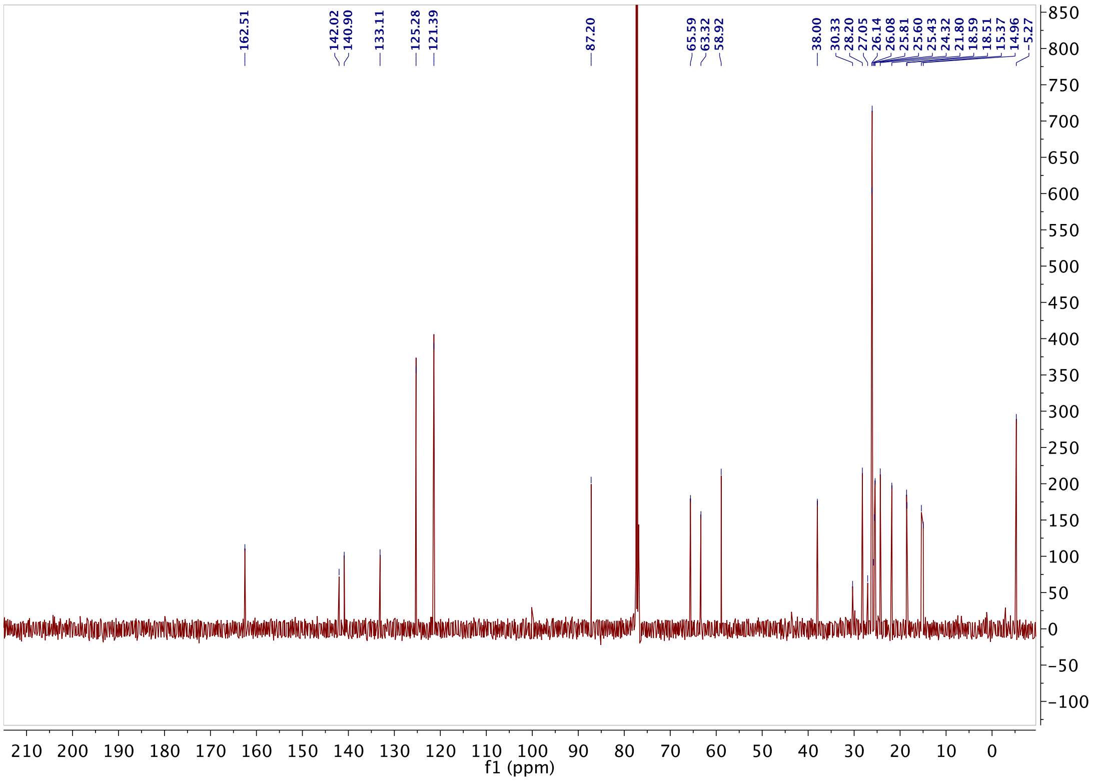
162.51
142.02
140.90
133.11
125.28
121.39
87.20
65.59
63.32
58.92
38.00
30.33
28.20
27.05
26.14
26.08
25.81
25.60
25.43
24.32
21.80
18.59
18.51
15.37
14.96
-5.27
f1 (ppm)

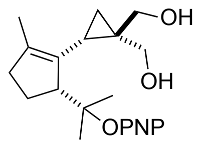
OH
OH
OPNP

(±)-6c

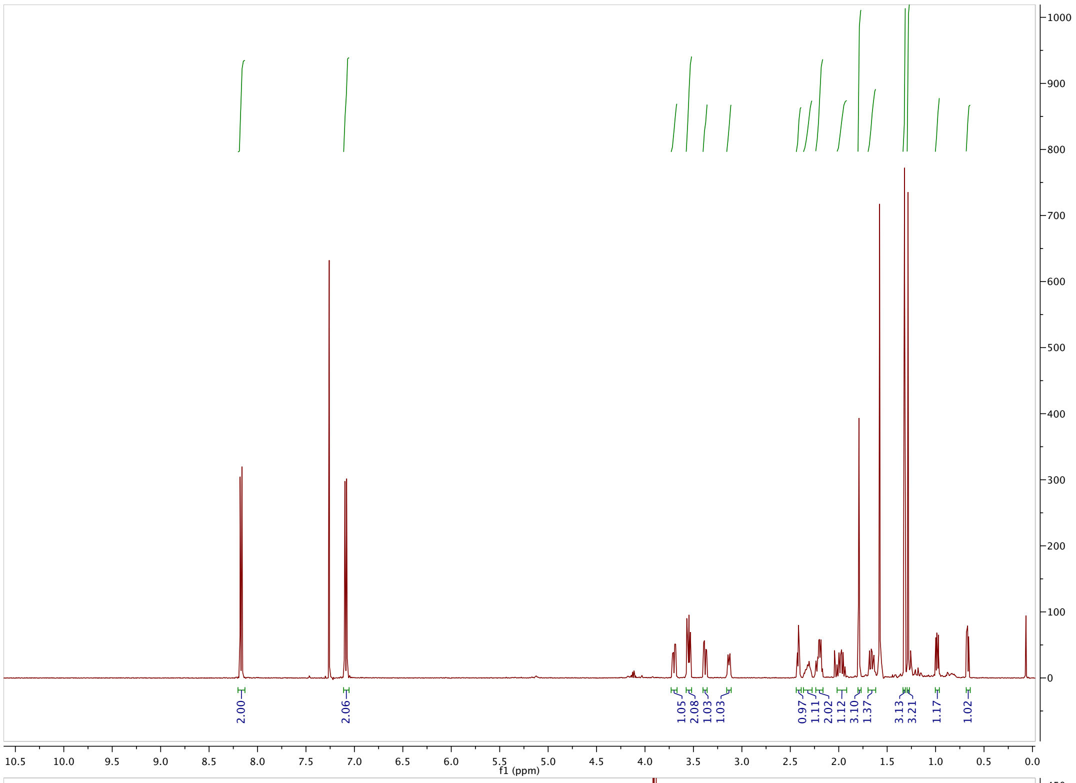
2.00
2.06
1.05
2.08
1.03
1.03
0.97
1.11
2.02
1.12
3.10
1.37
3.13
3.21
1.17
1.02
f1 (ppm)

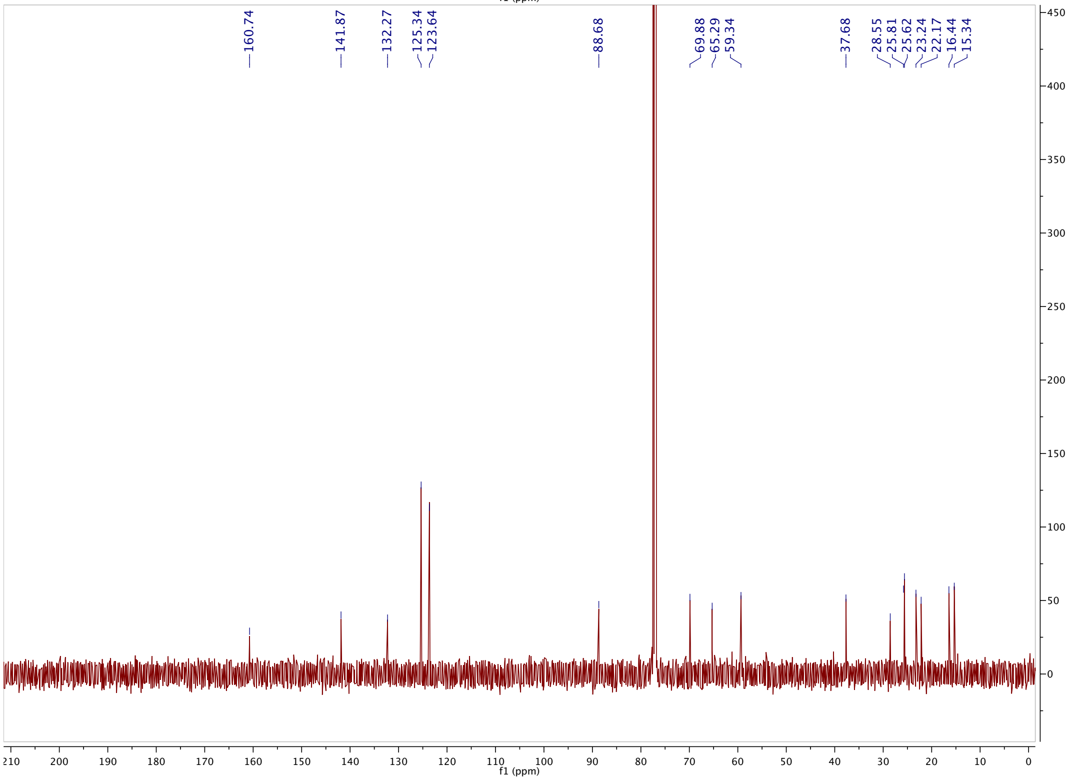
160.74
141.87
132.27
125.34
123.64
88.68
69.88
65.29
59.34
37.68
28.55
25.81
25.62
23.24
22.17
16.44
15.34
f1 (ppm)

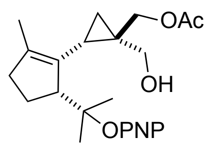
OAc
OH
OPNP

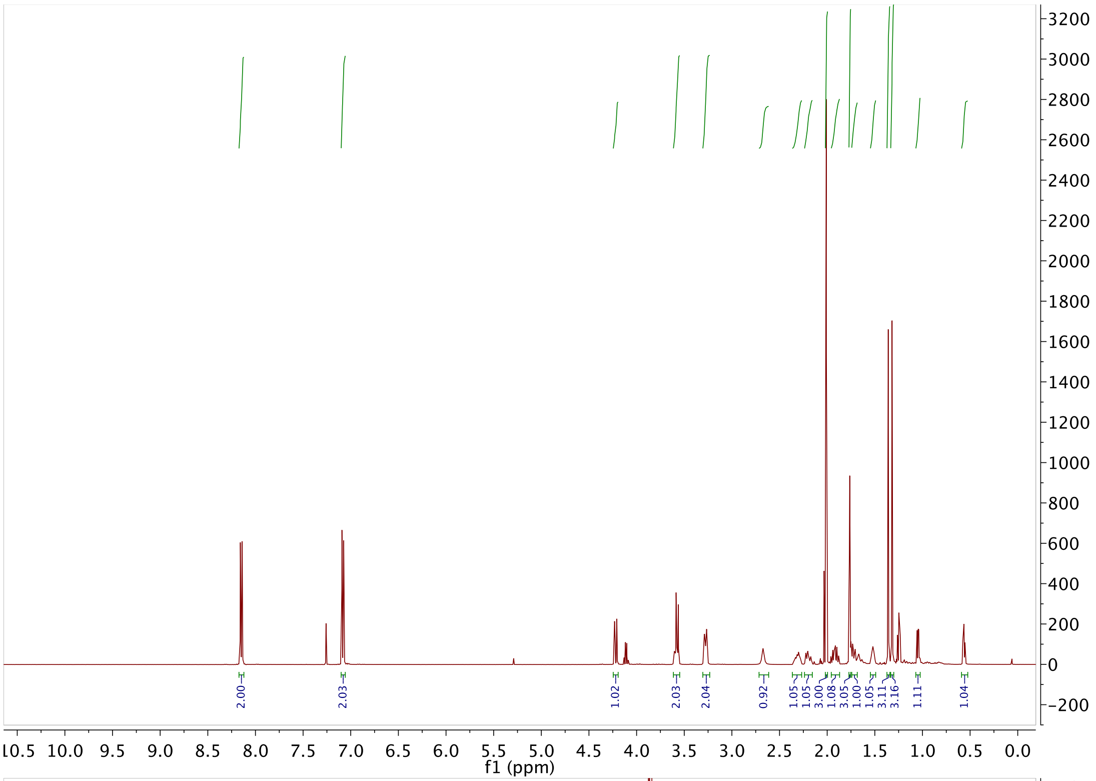
2.00
2.03
1.02
2.03
2.04
0.92
1.05
1.05
3.00
1.08
3.05
1.00
1.05
3.11
3.16
1.11
1.04
f1 (ppm)

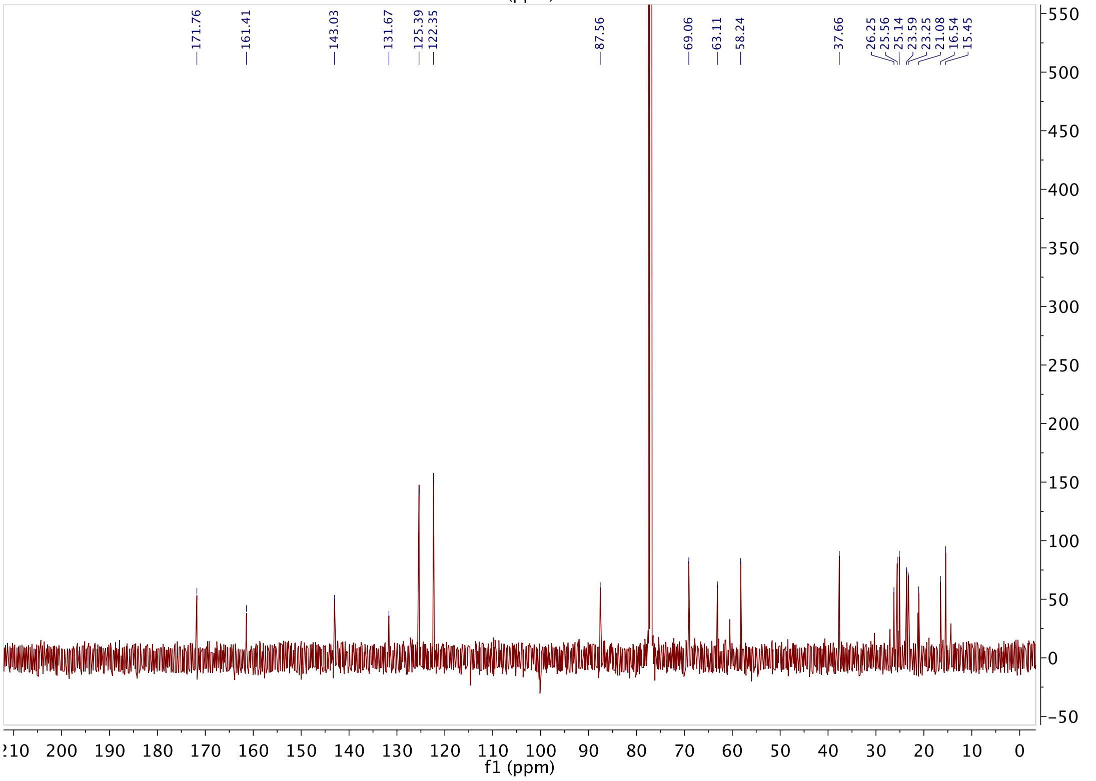
171.76
161.41
143.03
131.67
125.39
122.35
87.56
69.06
63.11
58.24
37.66
26.25
25.56
25.14
23.59
23.25
21.08
16.54
15.45
f1 (ppm)

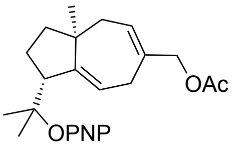

(±)-16b

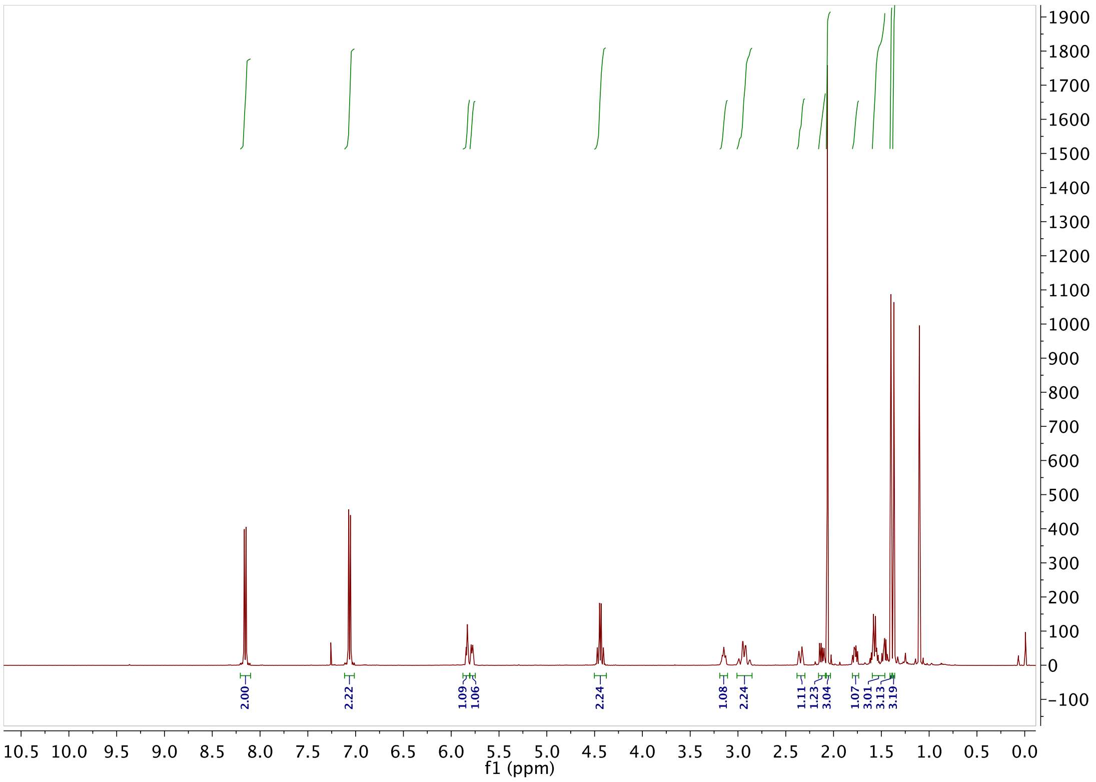

2.00
2.22
1.09
1.06
2.24
1.08
2.24
1.11
1.23
3.04
1.07
3.01
3.13
3.19
f1 (ppm)

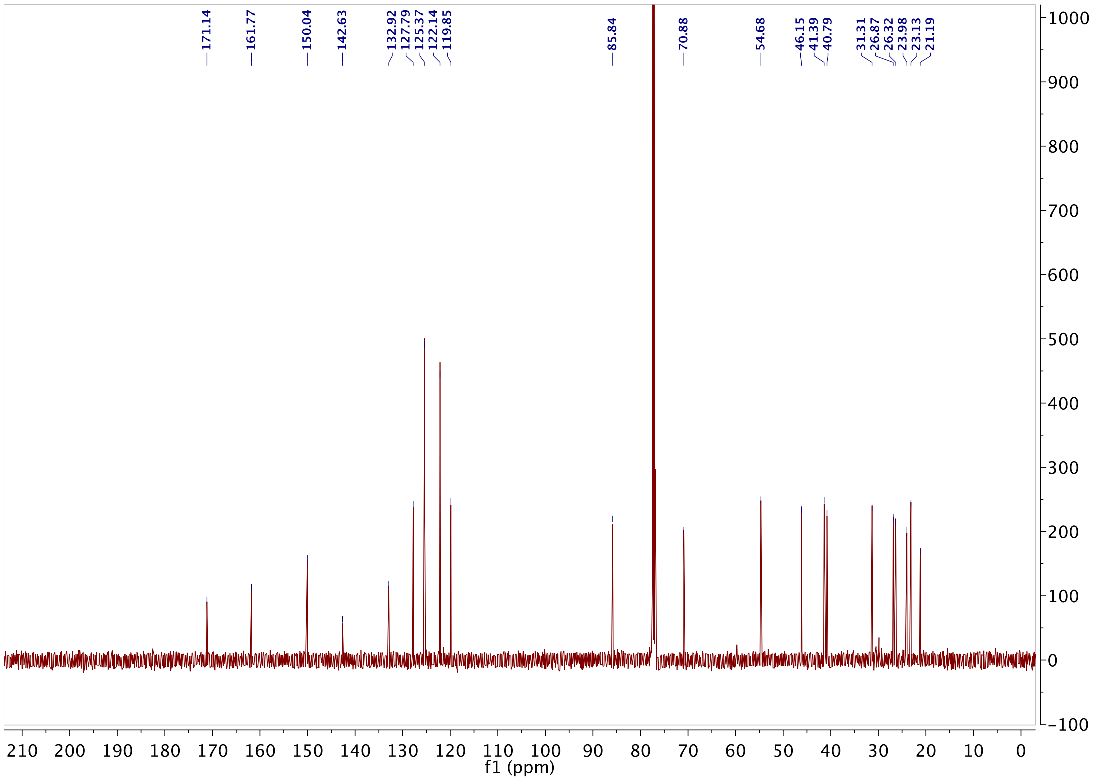

171.14
161.77
150.04
142.63
132.92
127.79
125.37
122.14
119.85
85.84
70.88
54.68
46.15
41.39
40.79
31.31
26.87
26.32
23.98
23.13
21.19
f1 (ppm)

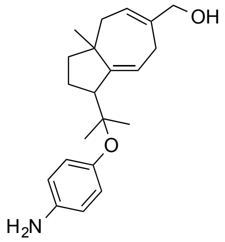

(±)-S-16b

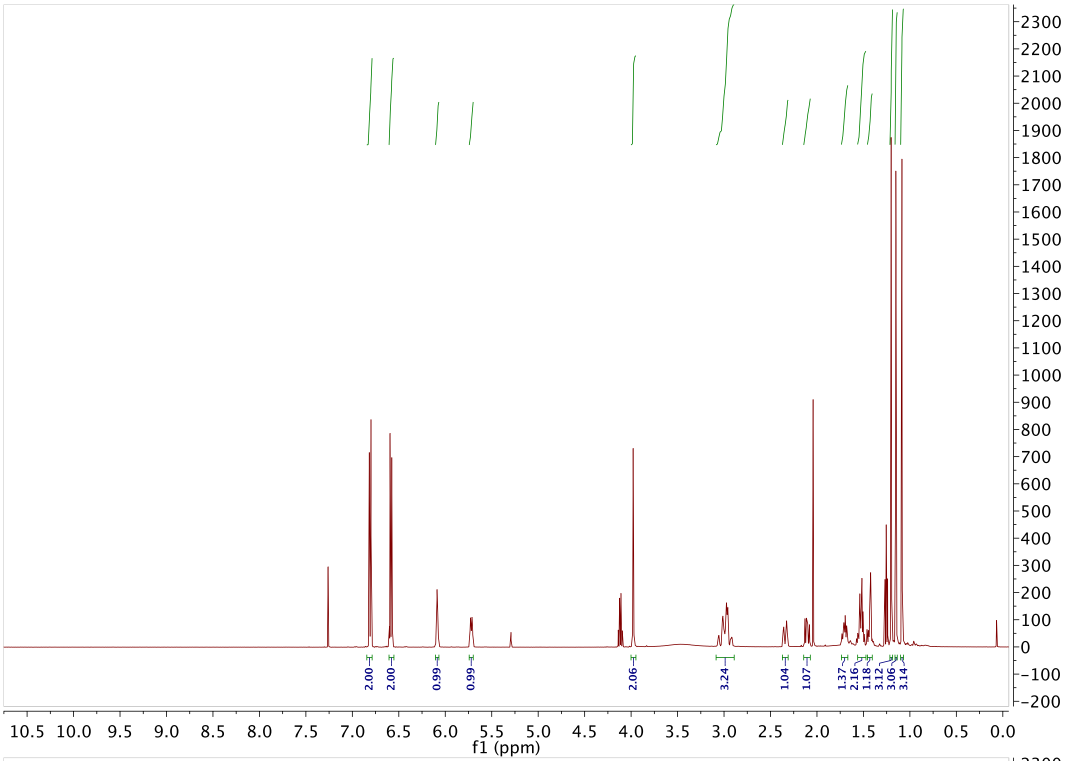

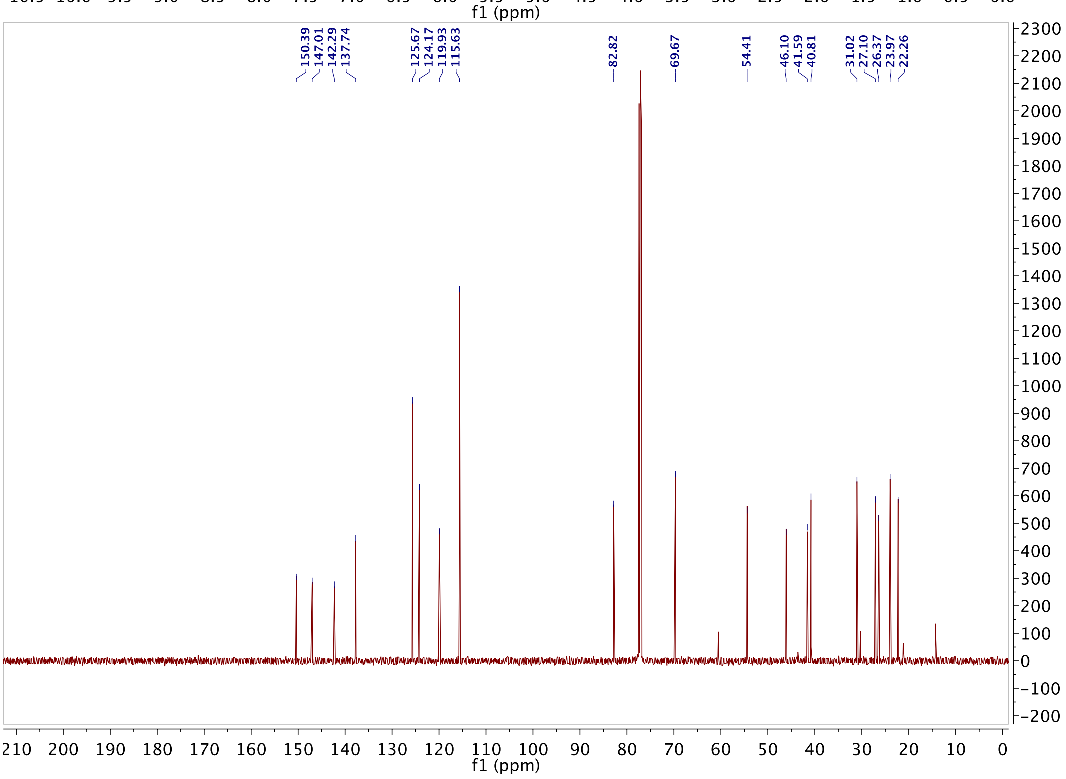

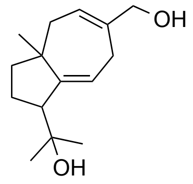
OH
OH

(±)-16a

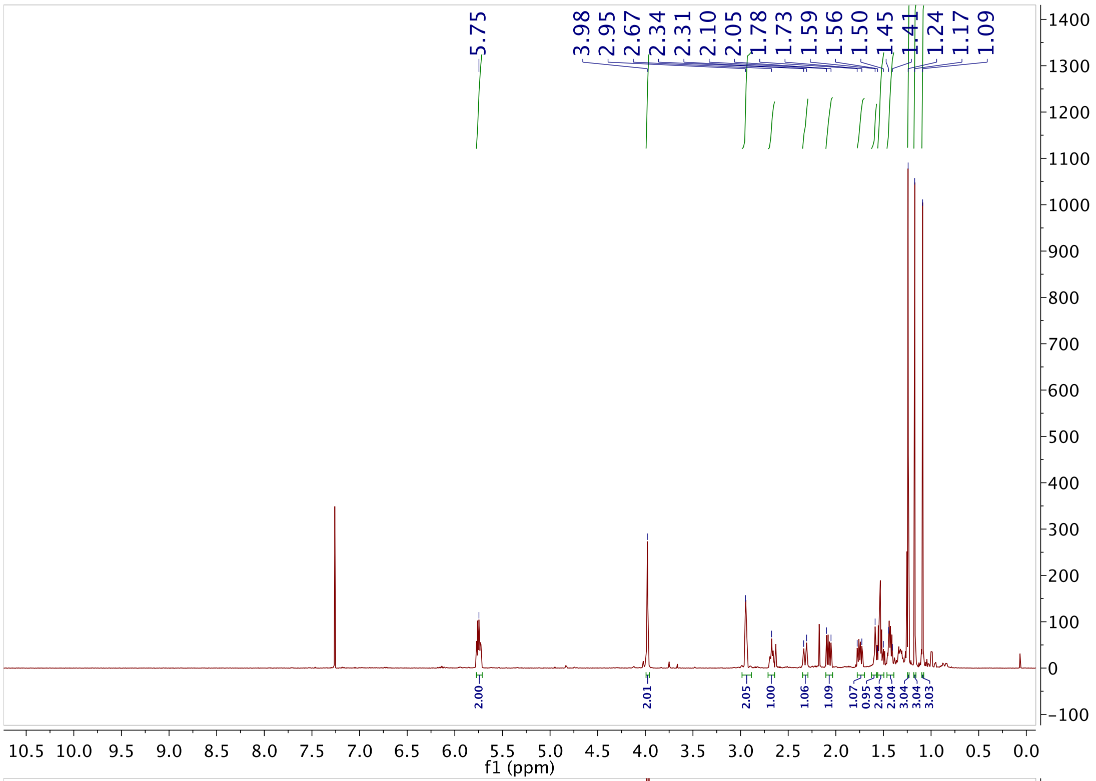
5.75
3.98
2.95
2.67
2.34
2.31
2.10
2.05
1.78
1.73
1.59
1.56
1.50
1.45
1.41
1.24
1.17
1.09
2.00
2.01
2.05
1.00
1.06
1.09
1.07
0.95
2.04
2.04
3.04
3.04
3.03
f1 (ppm)

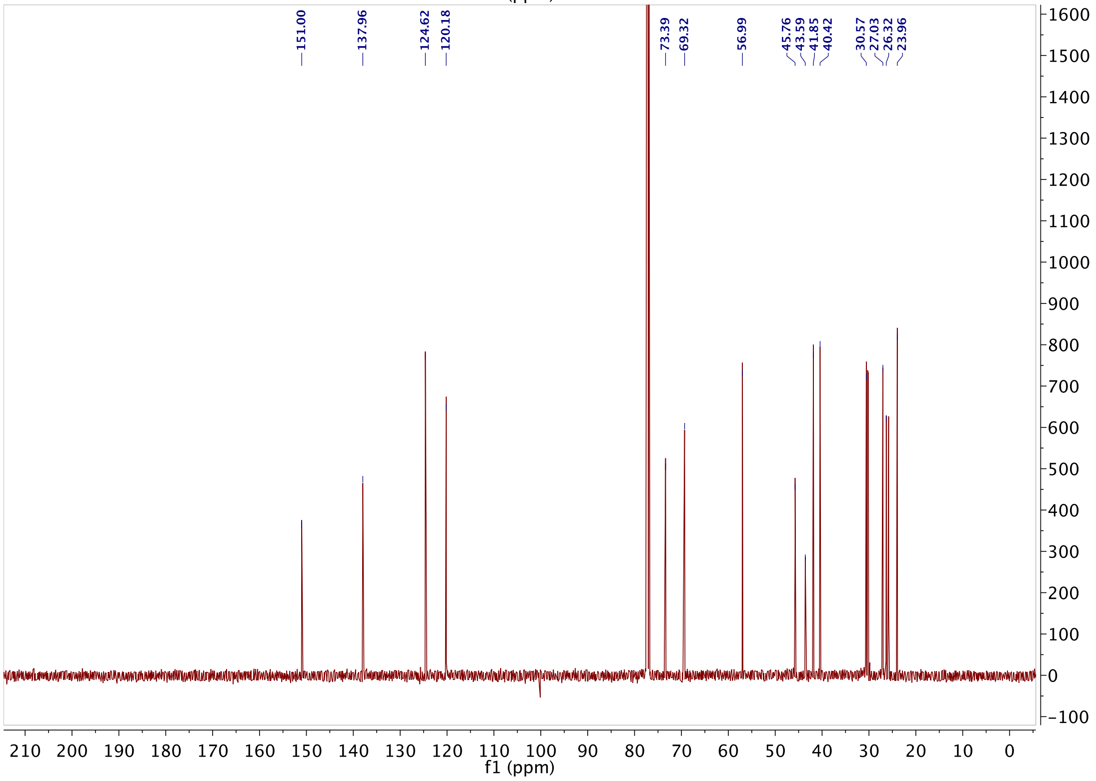
151.00
137.96
124.62
120.18
73.39
69.32
56.99
45.76
43.59
41.85
40.42
30.57
27.03
26.32
23.96
f1 (ppm)

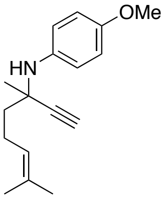
OMe
HN

17a

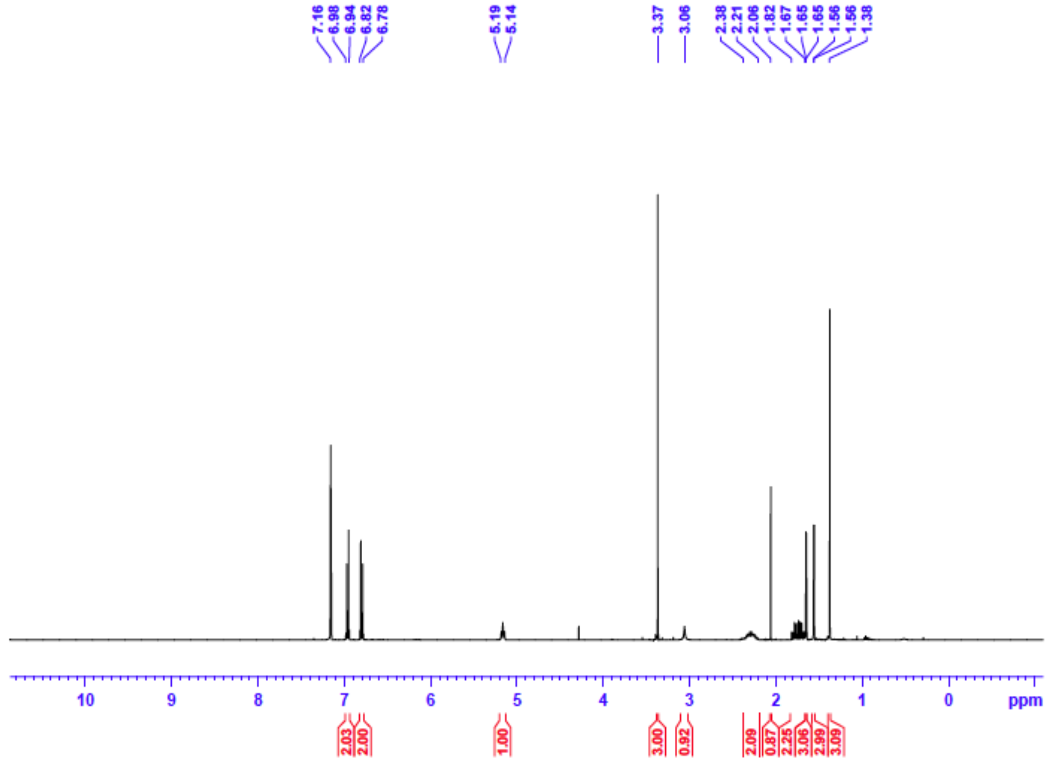
7.16
6.98
6.94
6.82
6.78
5.19
5.14
3.37
3.06
2.38
2.21
2.06
1.82
1.67
1.65
1.65
1.56
1.56
1.38
10 9 8 7 6 5 4 3 2 1 0 ppm
2.03
2.00
1.00
3.00
0.92
2.09
0.87
2.25
3.06
2.99
3.09

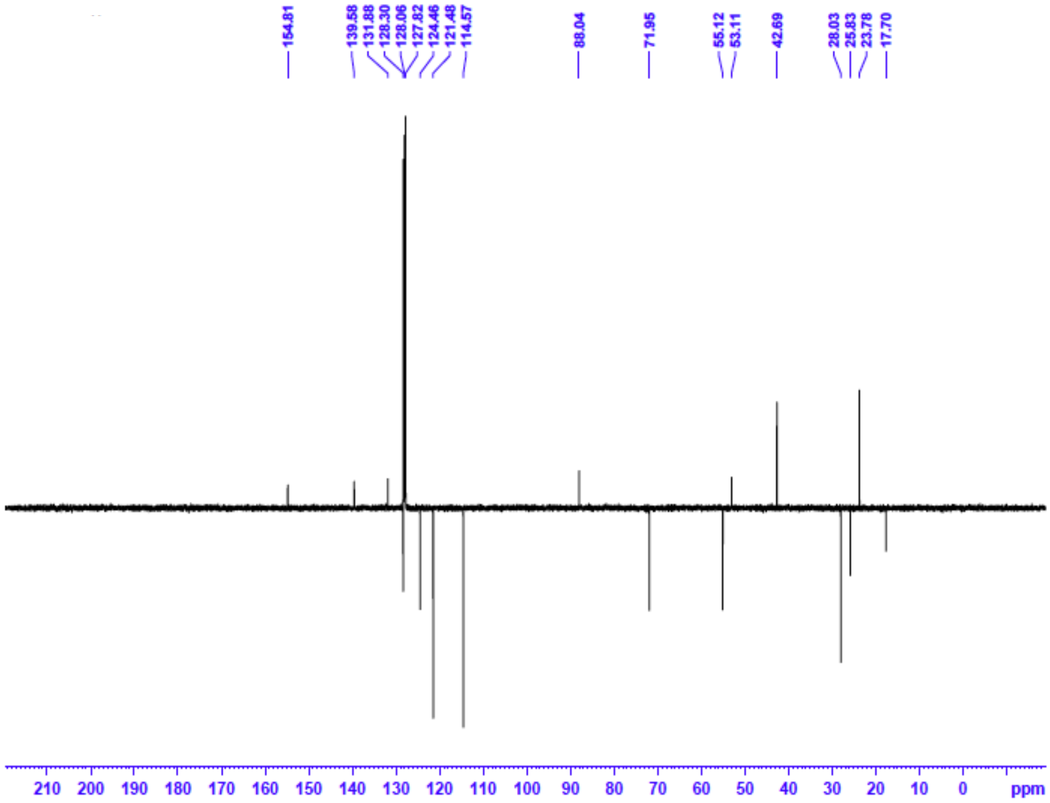
154.81
139.58
131.88
128.30
128.06
127.82
124.46
121.48
114.57
88.04
71.95
55.12
53.11
42.69
28.03
25.83
23.78
17.70
210 200 190 180 170 160 150 140 130 120 110 100 90 80 70 60 50 40 30 20 10 0 ppm

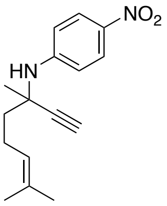

17b

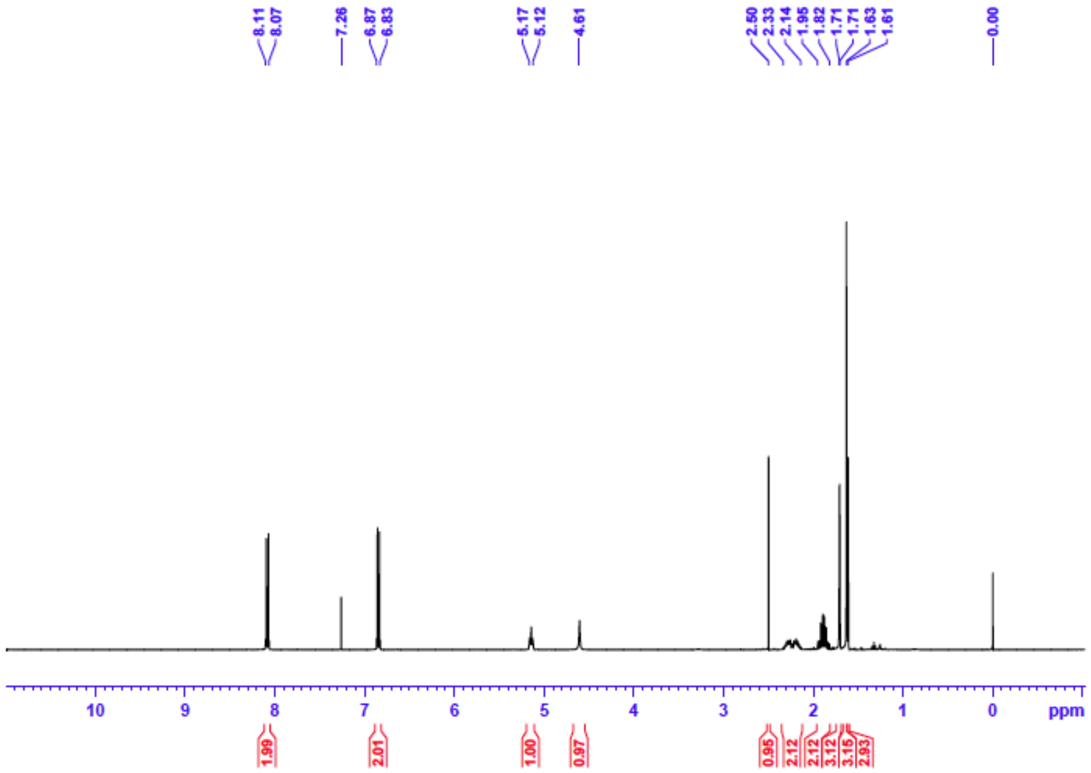

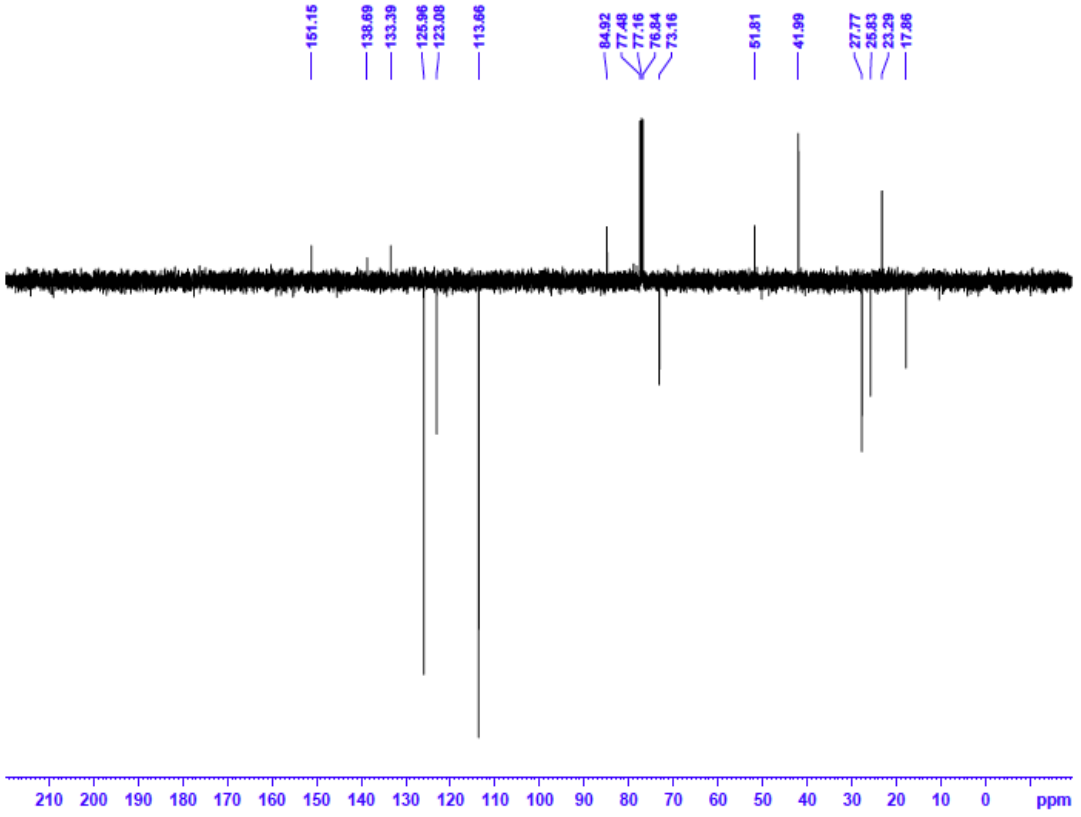

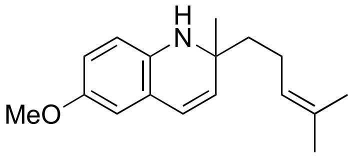
H
N
MeO

18

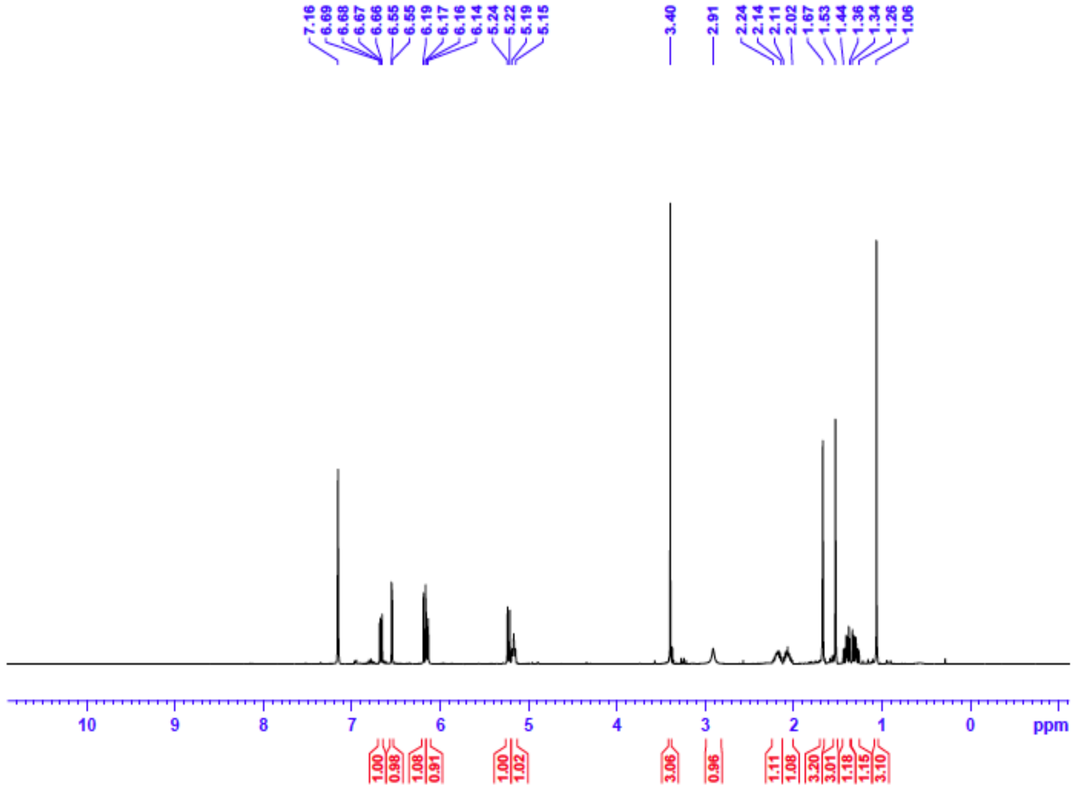
7.16
6.69
6.68
6.67
6.66
6.55
6.55
6.19
6.17
6.16
6.14
5.24
5.22
5.19
5.15
3.40
2.91
2.24
2.14
2.11
2.02
1.67
1.53
1.44
1.36
1.34
1.26
1.06
10 9 8 7 6 5 4 3 2 1 0 ppm
1.00
0.98
1.08
0.91
1.00
1.02
3.06
0.96
1.11
1.08
3.20
3.01
1.18
1.15
3.10

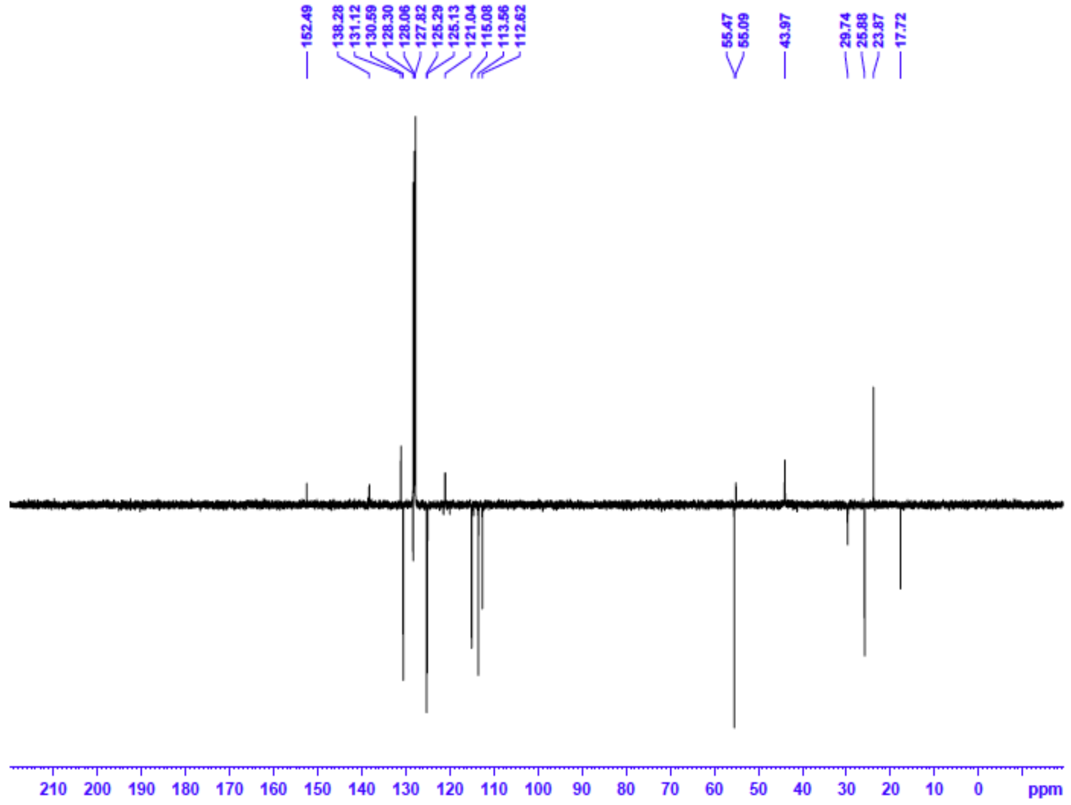
152.49
138.28
131.12
130.59
128.30
128.06
127.82
125.29
125.13
121.04
115.08
113.56
112.62
55.47
55.09
43.97
29.74
25.88
23.87
17.72
210 200 190 180 170 160 150 140 130 120 110 100 90 80 70 60 50 40 30 20 10 0 ppm

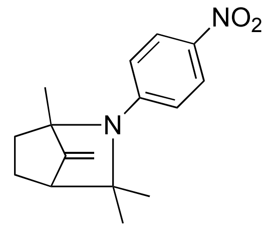

19

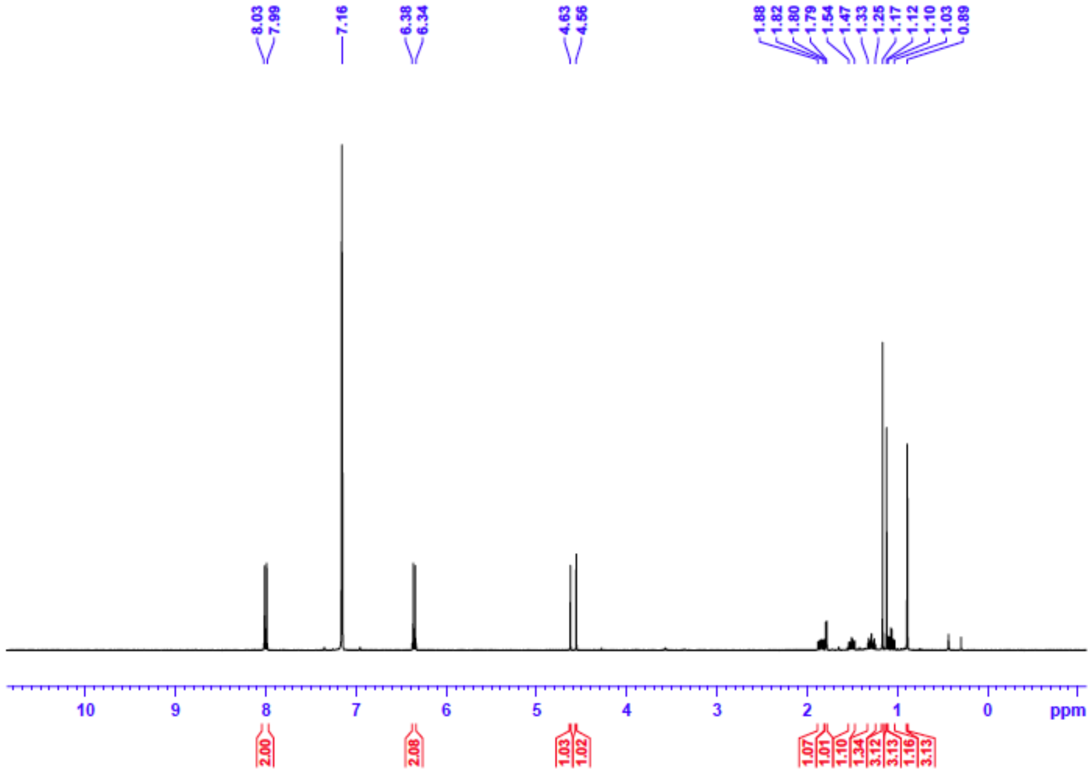
8.03
7.99
7.16
6.38
6.34
4.63
4.56
1.88
1.82
1.80
1.79
1.54
1.47
1.33
1.25
1.17
1.12
1.10
1.03
0.89
10
9
8
7
6
5
4
3
2
1
0
ppm
2.00
2.08
1.03
1.02
1.07
1.01
1.10
1.34
3.12
3.13
1.16
3.13

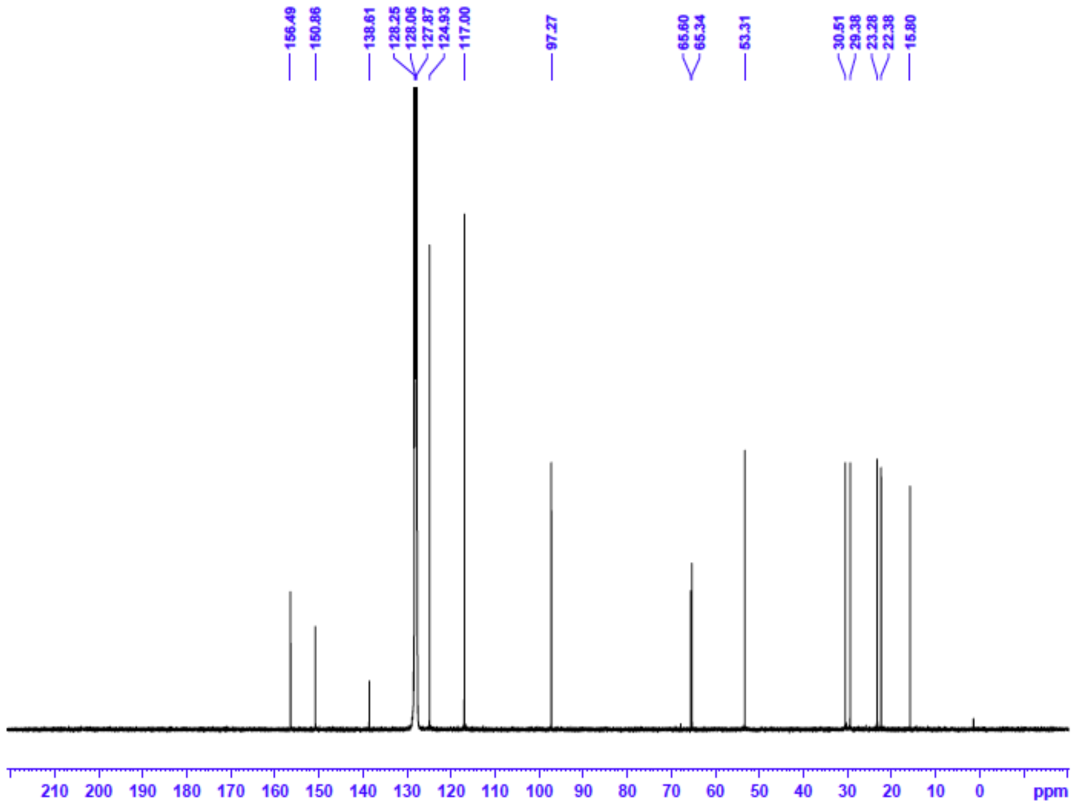
156.49
150.86
138.61
128.25
128.06
127.87
124.93
117.00
97.27
65.60
65.34
53.31
30.51
29.38
23.28
22.38
15.80
210
200
190
180
170
160
150
140
130
120
110
100
90
80
70
60
50
40
30
20
10
0
ppm

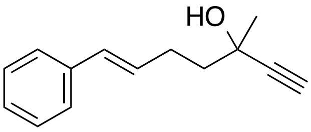

26a

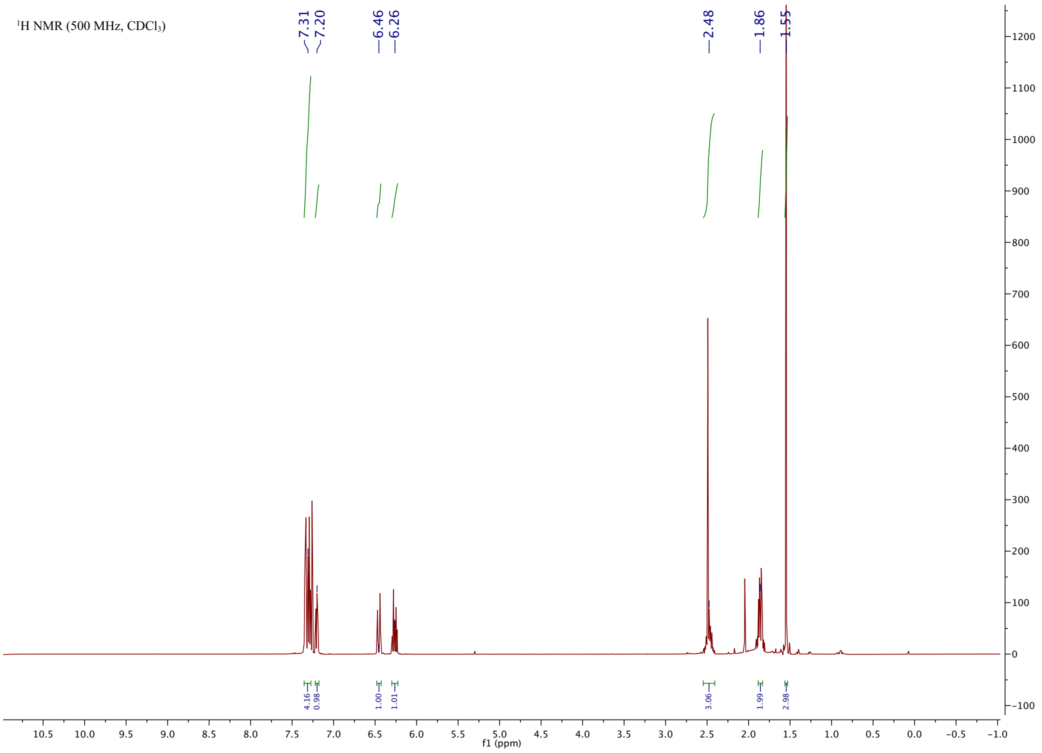

^{1}H NMR (500 MHz, CDCl$_3$)
7.31
7.20
6.46
6.26
2.48
1.86
1.55
4.16
0.98
1.00
1.01
3.06
1.99
2.98
f1 (ppm)

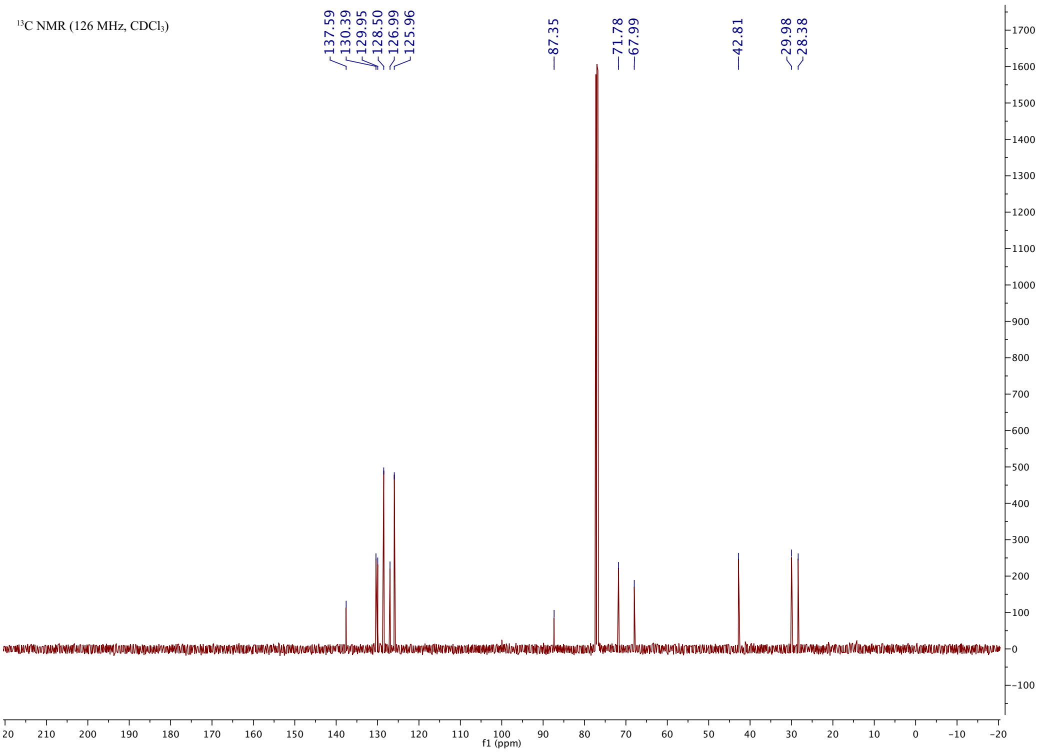

^{13}C NMR (126 MHz, CDCl$_3$)
137.59
130.39
129.95
128.50
126.99
125.96
87.35
71.78
67.99
42.81
29.98
28.38
f1 (ppm)

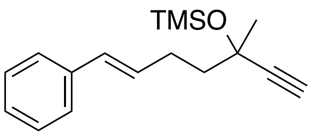
TMSO

26b

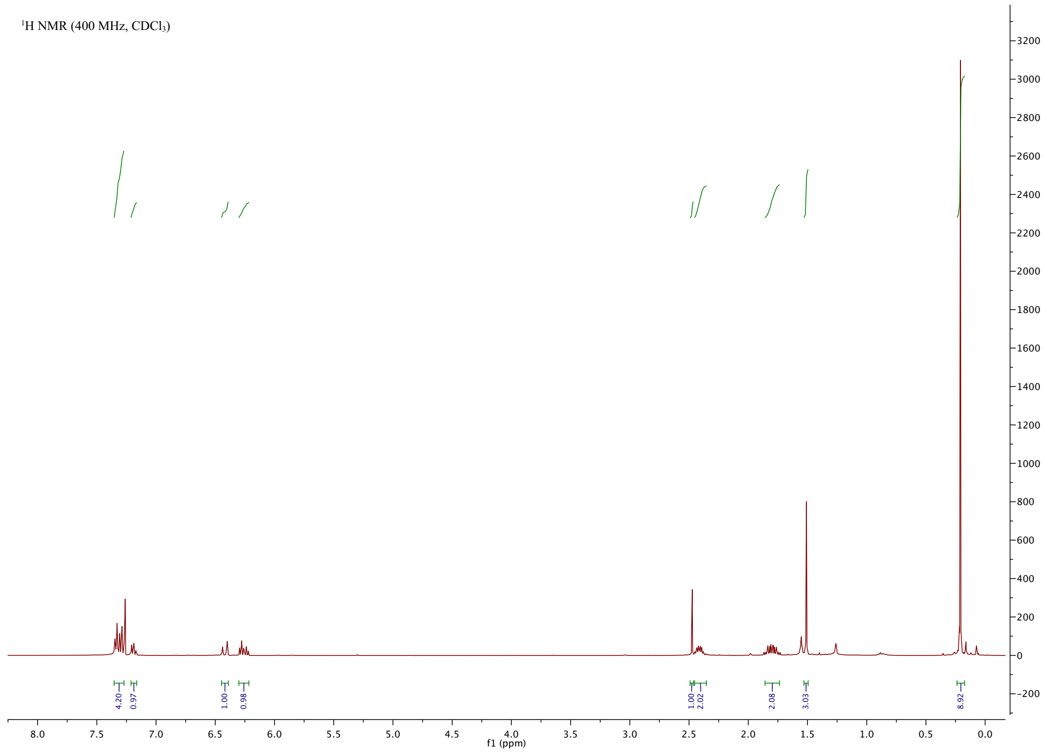
^{1}H NMR (400 MHz, CDCl$_3$)
4.20
0.97
1.00
0.98
1.00
2.02
2.08
3.03
8.92
f1 (ppm)

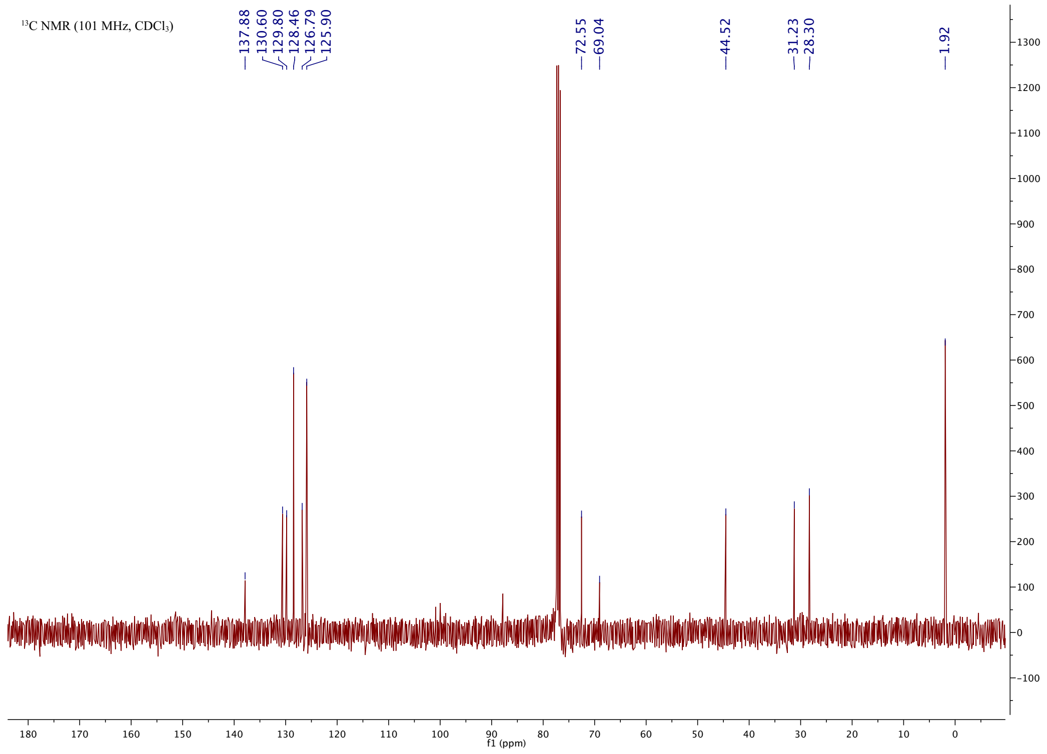
^{13}C NMR (101 MHz, CDCl$_3$)
137.88
130.60
129.80
128.46
126.79
125.90
72.55
69.04
44.52
31.23
28.30
1.92
f1 (ppm)

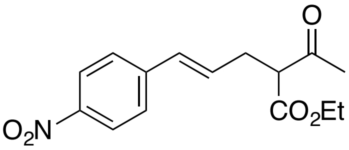
O
CO2Et
O2N

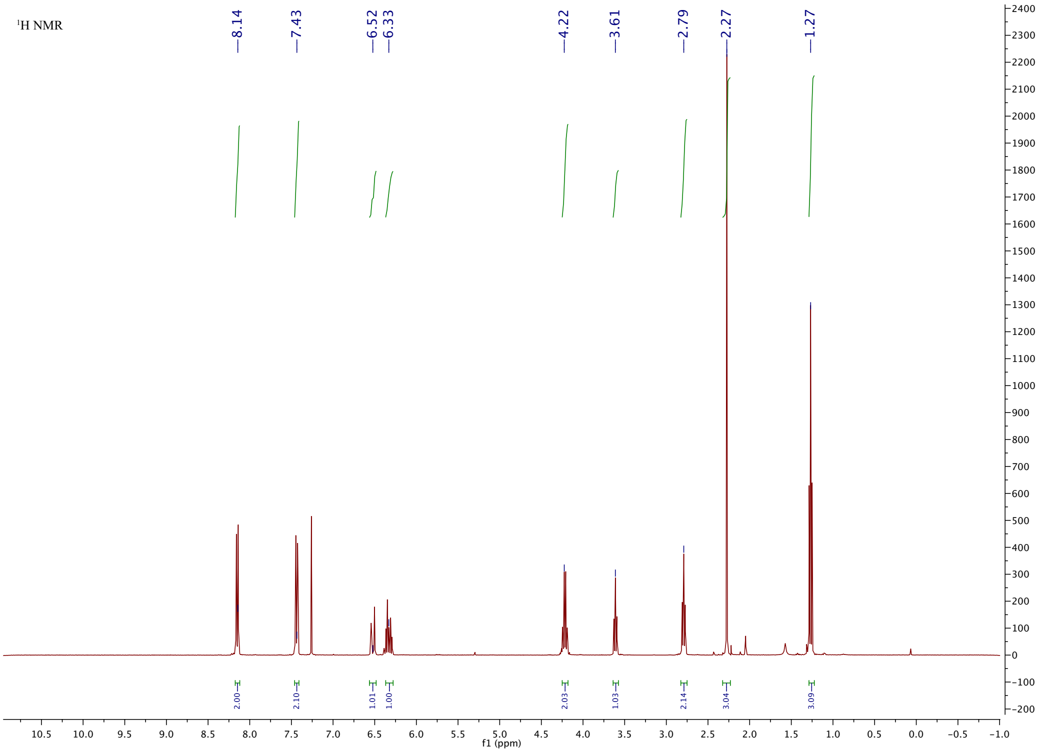
1H NMR
8.14
7.43
6.52
6.33
4.22
3.61
2.79
2.27
1.27
2.00
2.10
1.01
1.00
2.03
1.03
2.14
3.04
3.09
f1 (ppm)

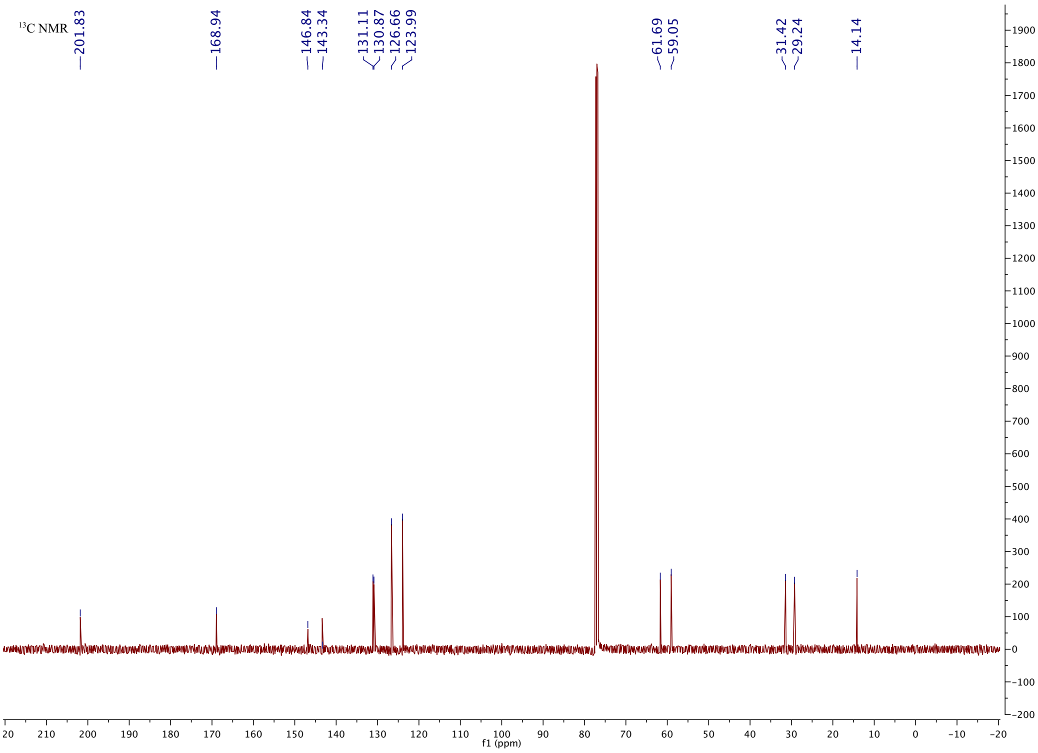
13C NMR
201.83
168.94
146.84
143.34
131.11
130.87
126.66
123.99
61.69
59.05
31.42
29.24
14.14
f1 (ppm)

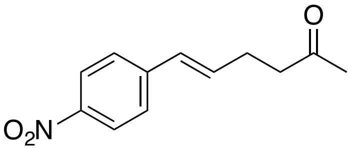

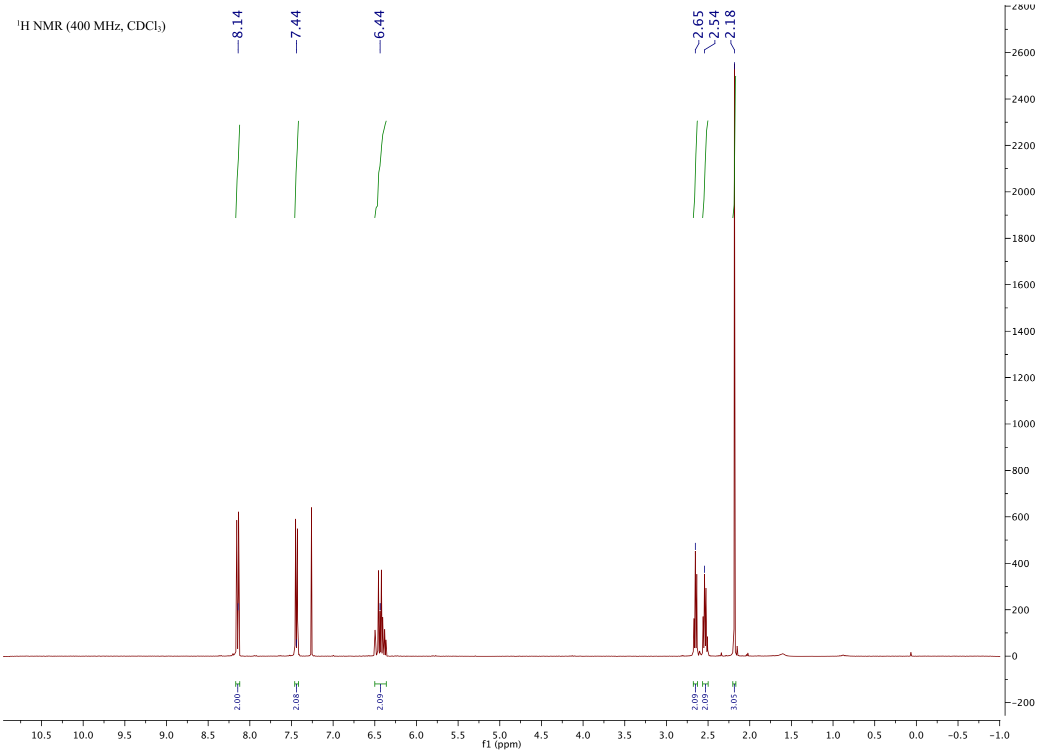

^{1}H NMR (400 MHz, CDCl$_3$)
8.14
7.44
6.44
2.65
2.54
2.18
2.00
2.08
2.09
2.09
2.09
3.05
f1 (ppm)

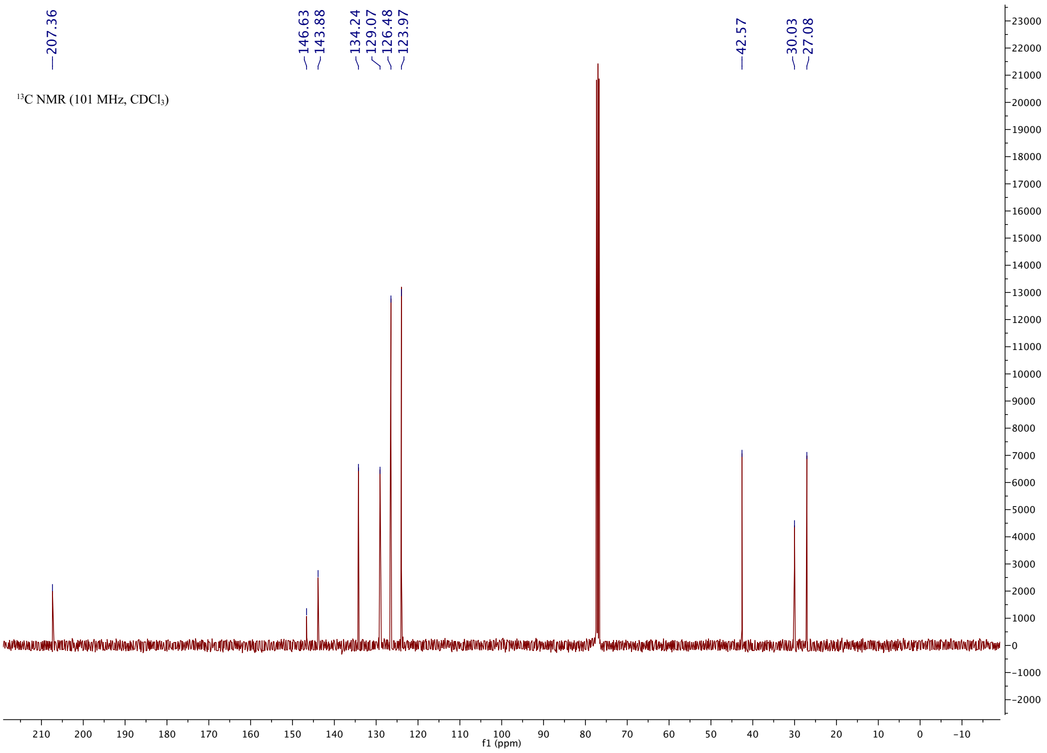

^{13}C NMR (101 MHz, CDCl$_3$)
207.36
146.63
143.88
134.24
129.07
126.48
123.97
42.57
30.03
27.08
f1 (ppm)

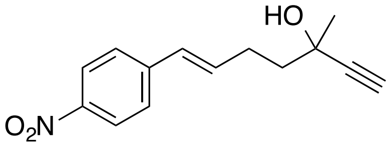

26c

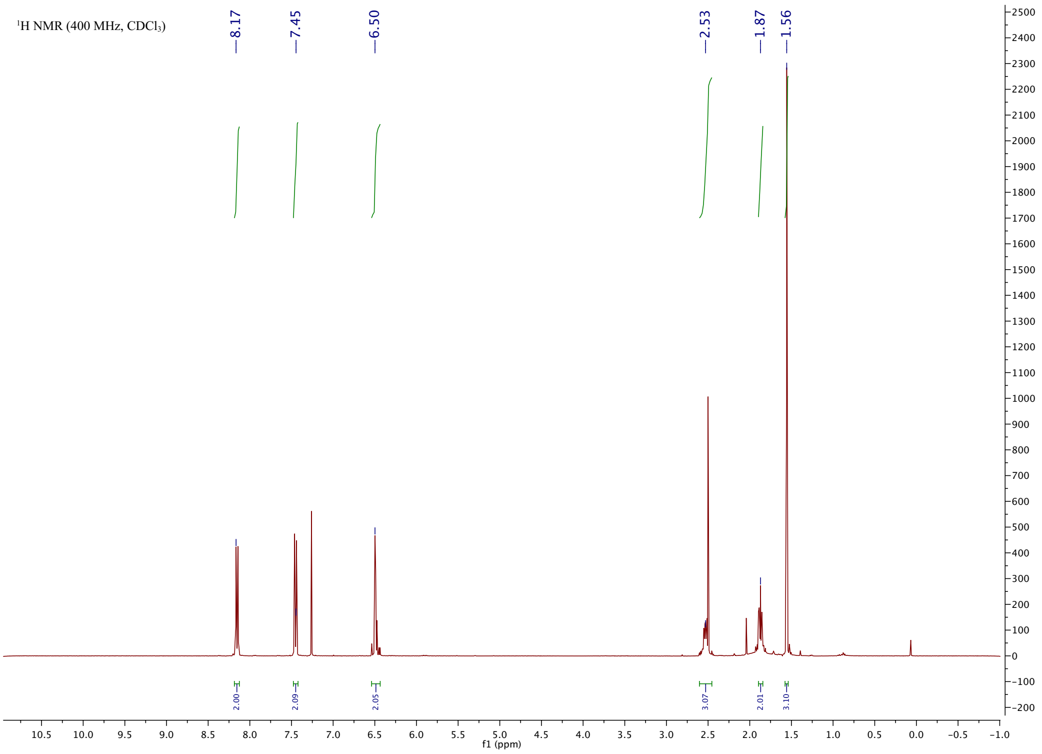

^{1}H NMR (400 MHz, CDCl$_3$)
8.17
7.45
6.50
2.53
1.87
1.56
2.00
2.09
2.05
3.07
2.01
3.10
f1 (ppm)

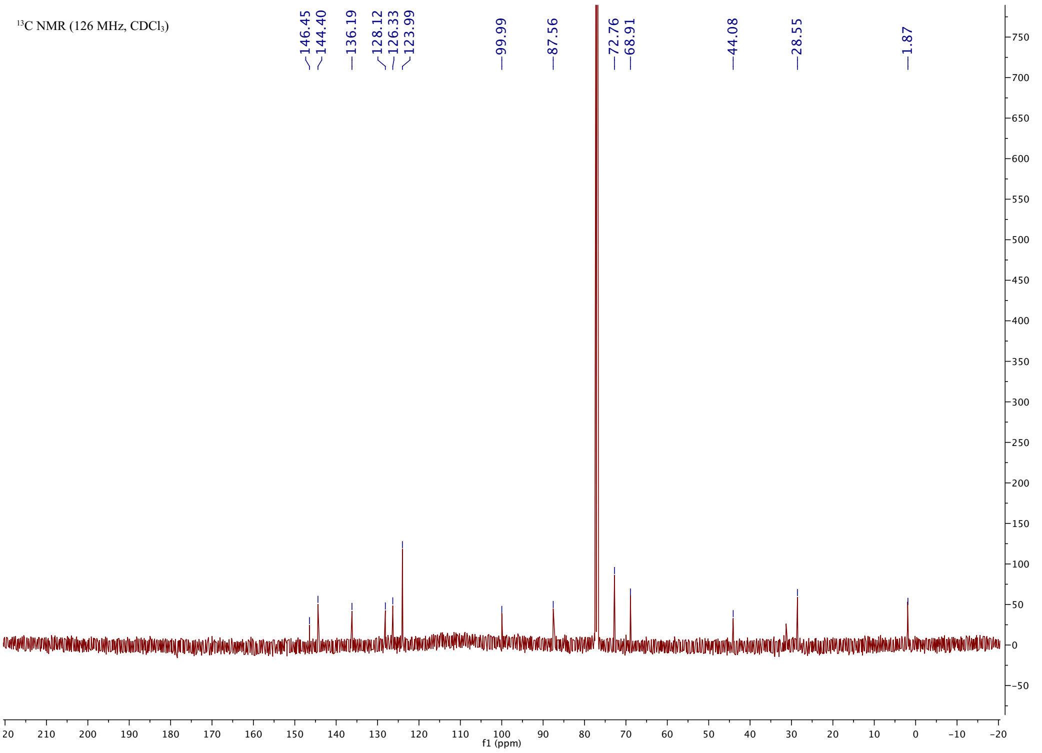

^{13}C NMR (126 MHz, CDCl$_3$)
146.45
144.40
136.19
128.12
126.33
123.99
99.99
87.56
72.76
68.91
44.08
28.55
1.87
f1 (ppm)

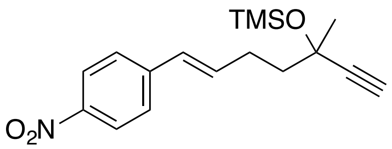
TMSO
O2N

26d

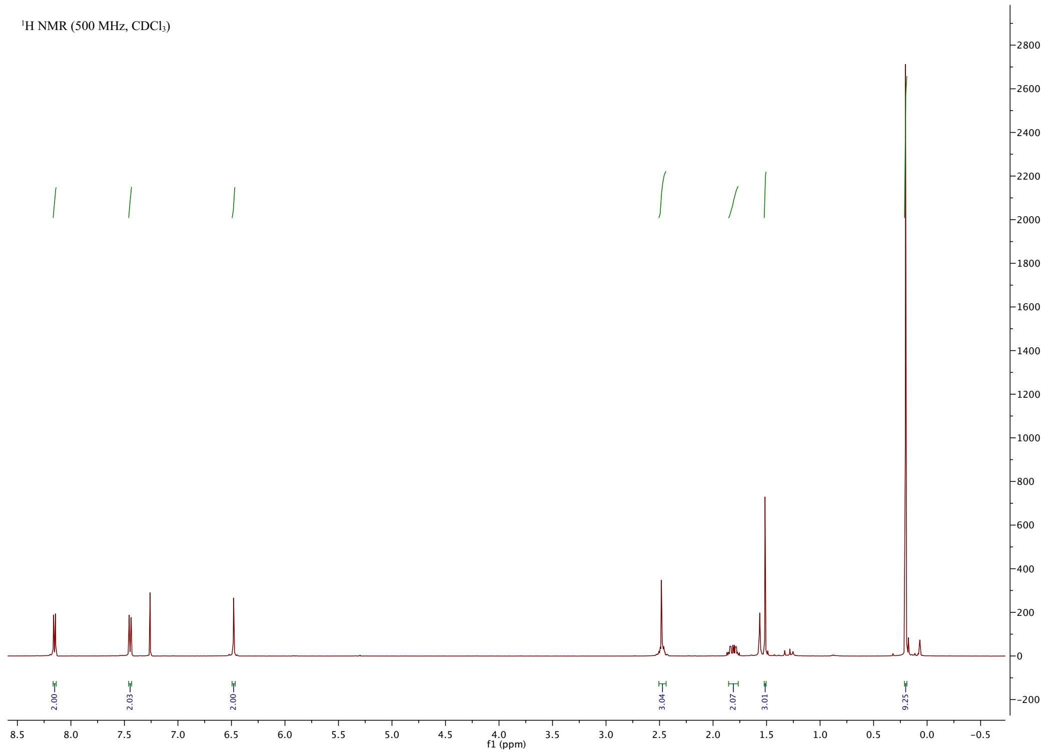
1H NMR (500 MHz, CDCl3)
2.00
2.03
2.00
3.04
2.07
3.01
9.25
f1 (ppm)

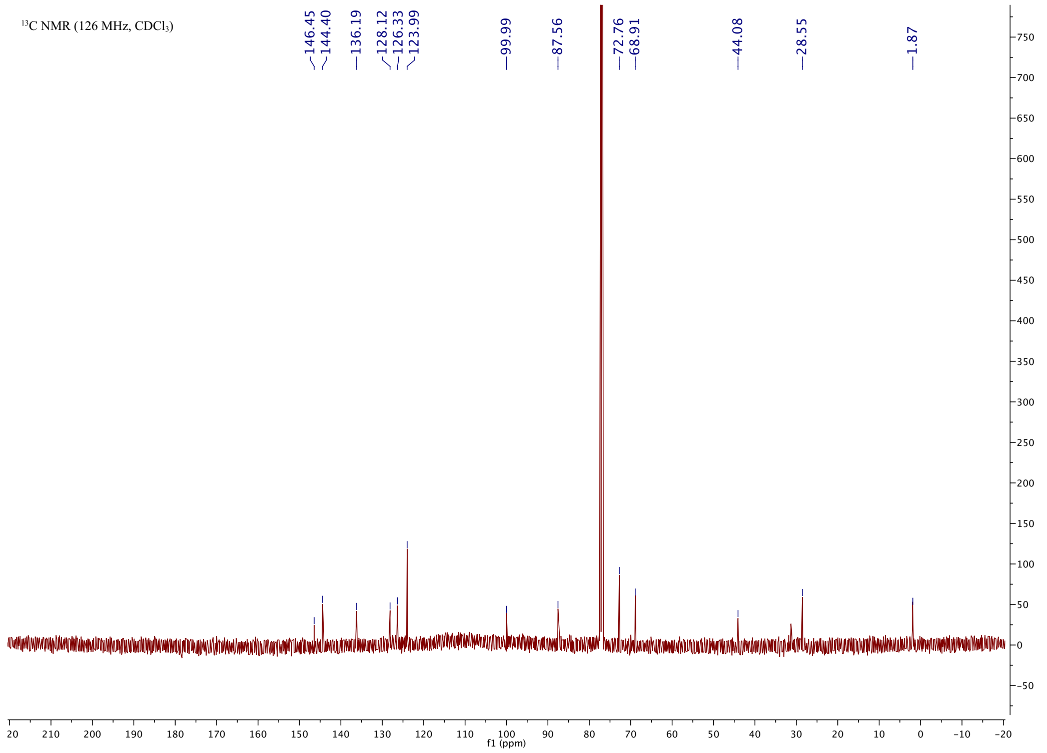
13C NMR (126 MHz, CDCl3)
146.45
144.40
136.19
128.12
126.33
123.99
99.99
87.56
72.76
68.91
44.08
28.55
1.87
f1 (ppm)

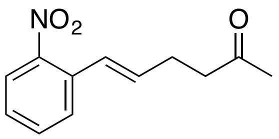

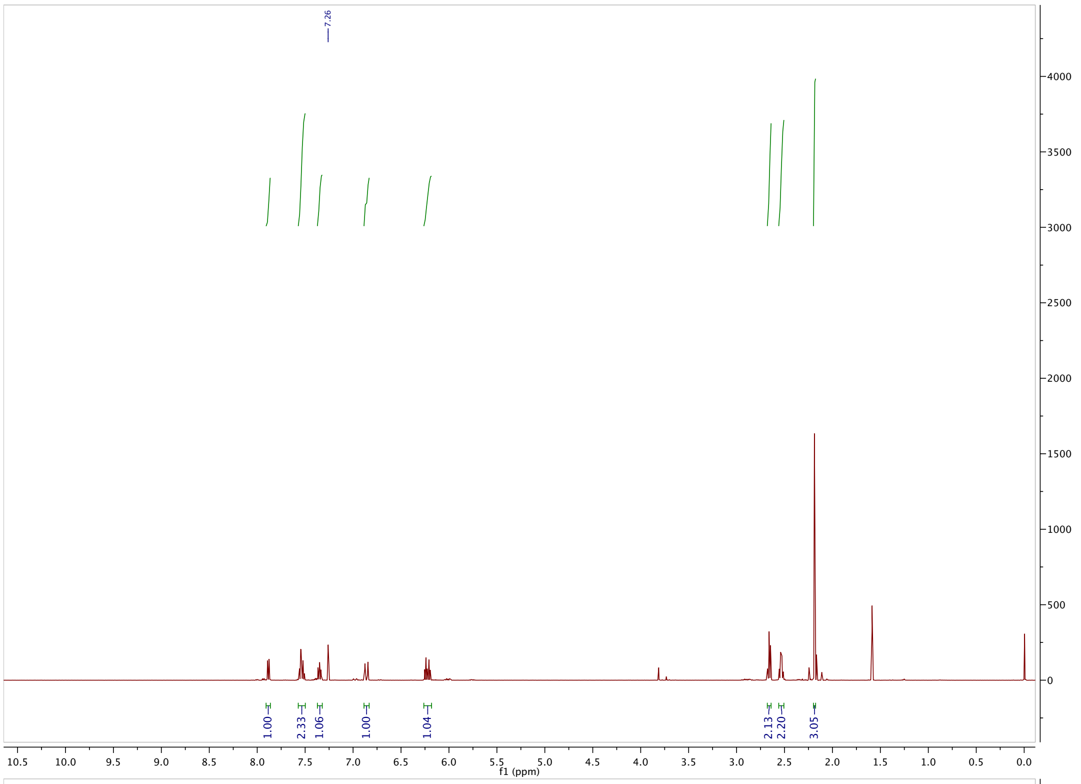

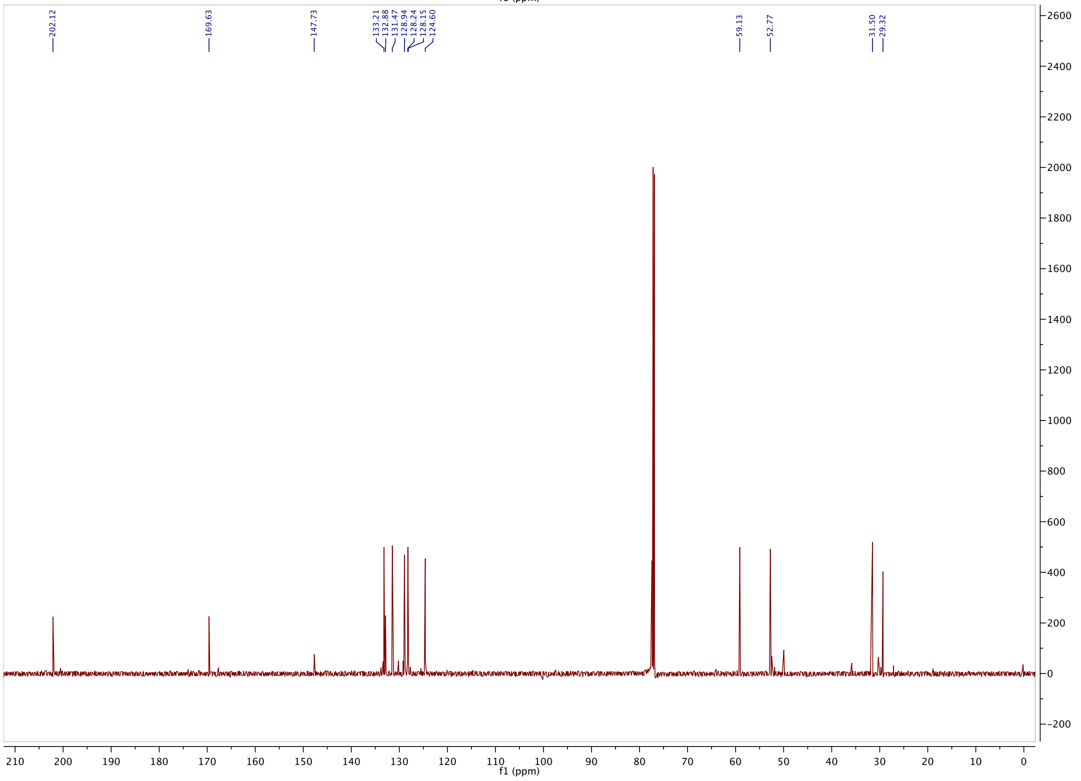

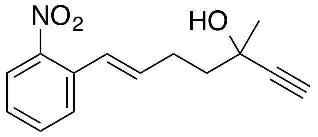
NO2
HO

26e

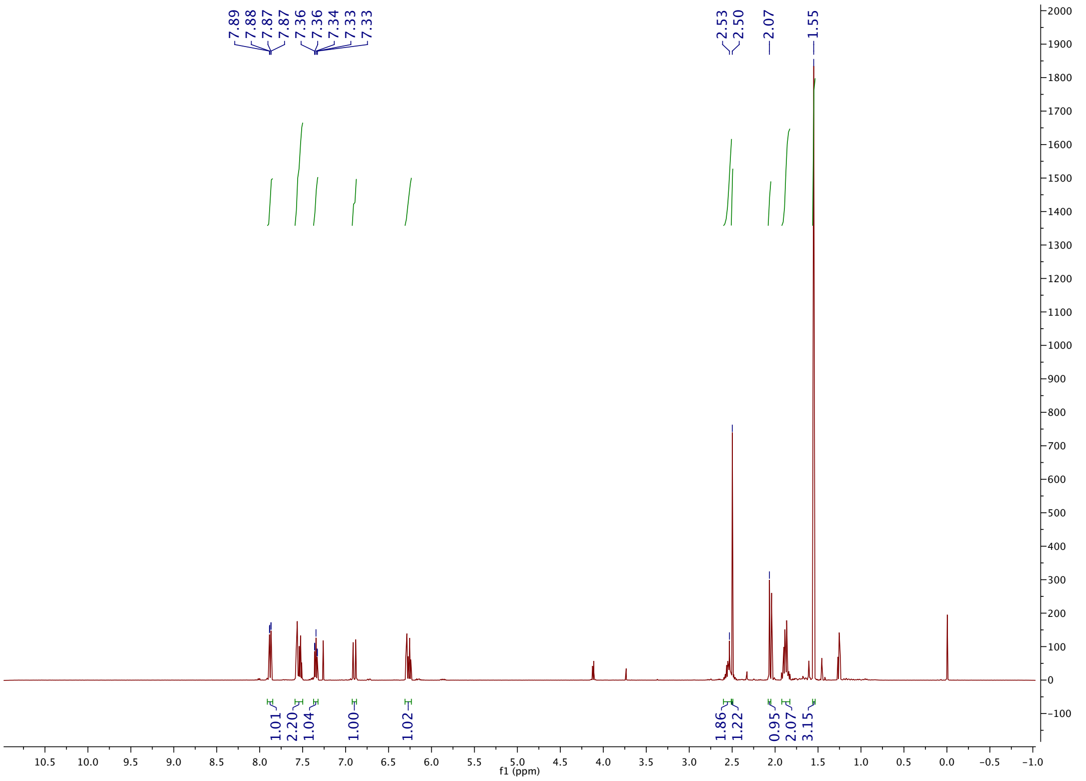
7.89
7.88
7.87
7.87
7.36
7.36
7.34
7.33
7.33
2.53
2.50
2.07
1.55
1.01
2.20
1.04
1.00
1.02
1.86
1.22
0.95
2.07
3.15
f1 (ppm)

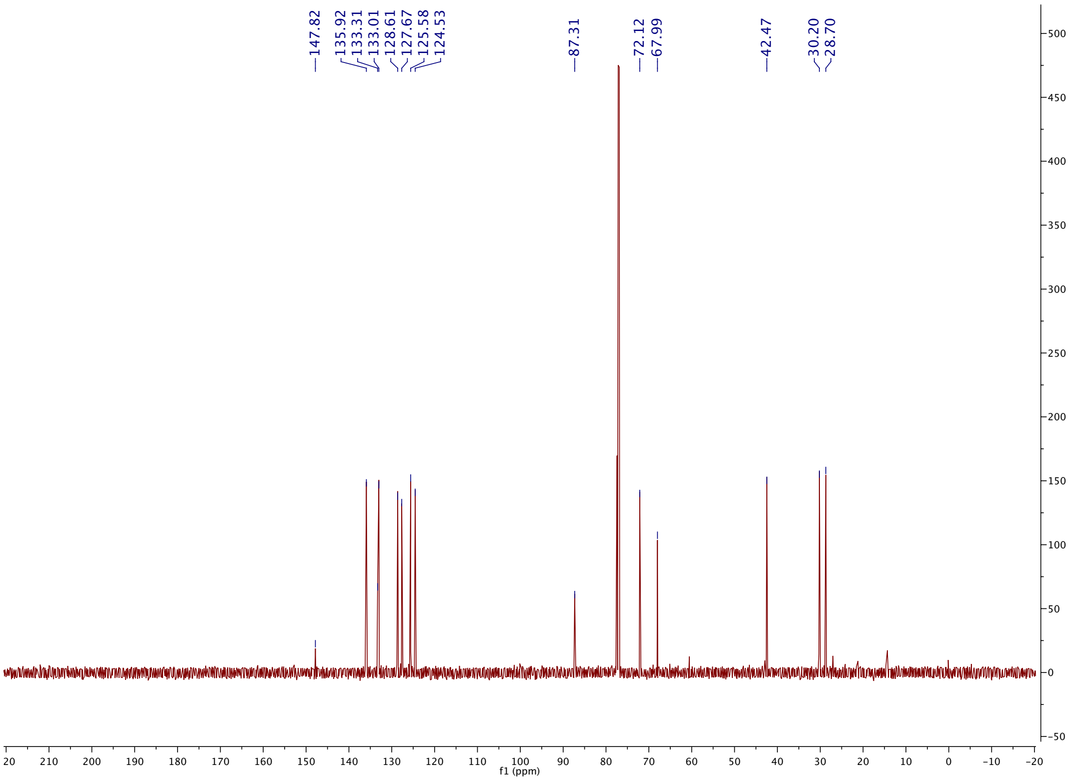
147.82
135.92
133.31
133.01
128.61
127.67
125.58
124.53
87.31
72.12
67.99
42.47
30.20
28.70
f1 (ppm)

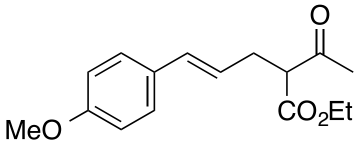

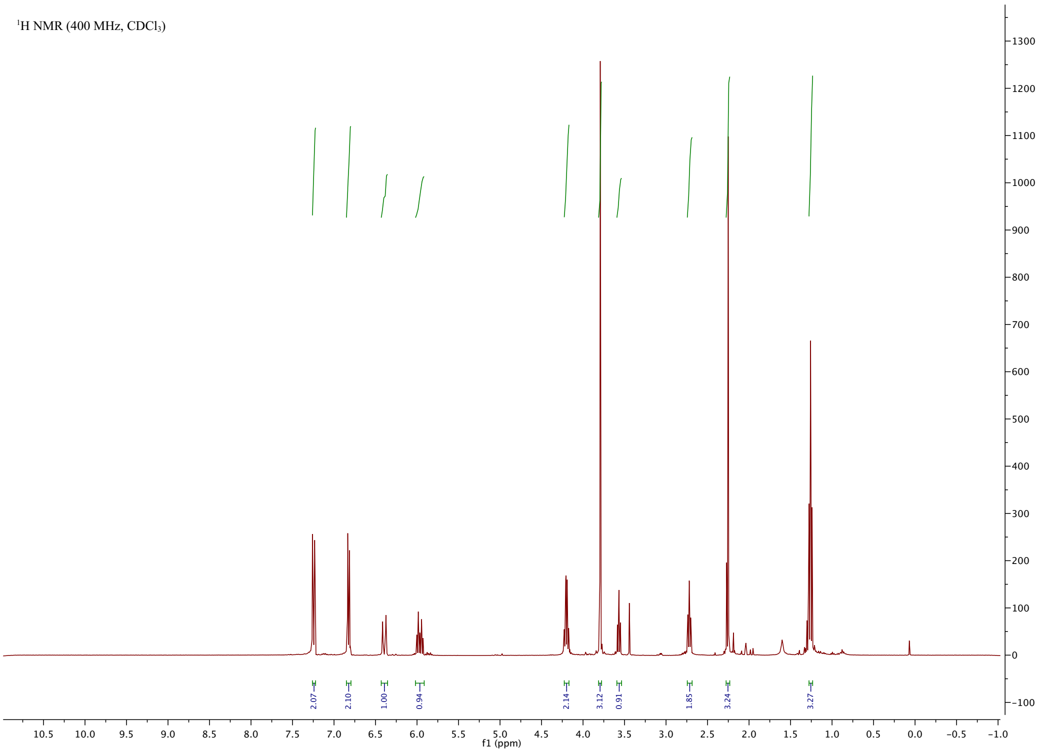
1H NMR (400 MHz, CDCl3)
2.07
2.10
1.00
0.94
2.14
3.12
0.91
1.85
3.24
3.27
f1 (ppm)

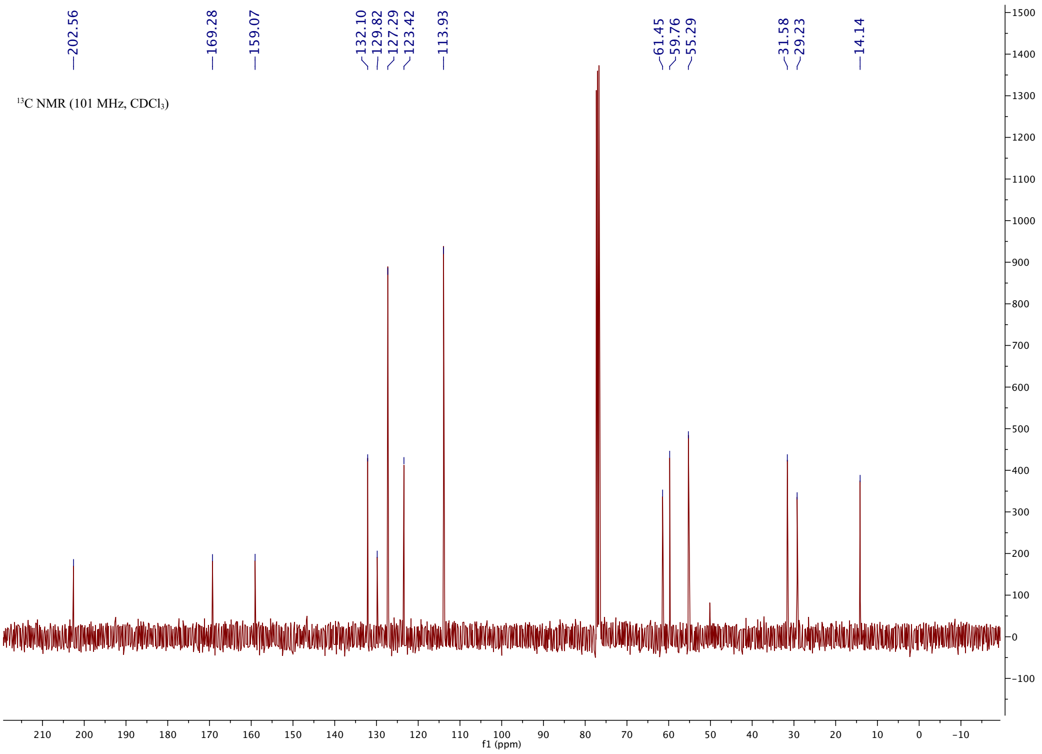
202.56
169.28
159.07
132.10
129.82
127.29
123.42
113.93
61.45
59.76
55.29
31.58
29.23
14.14
13C NMR (101 MHz, CDCl3)
f1 (ppm)

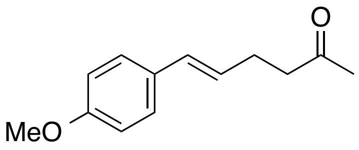
O
MeO

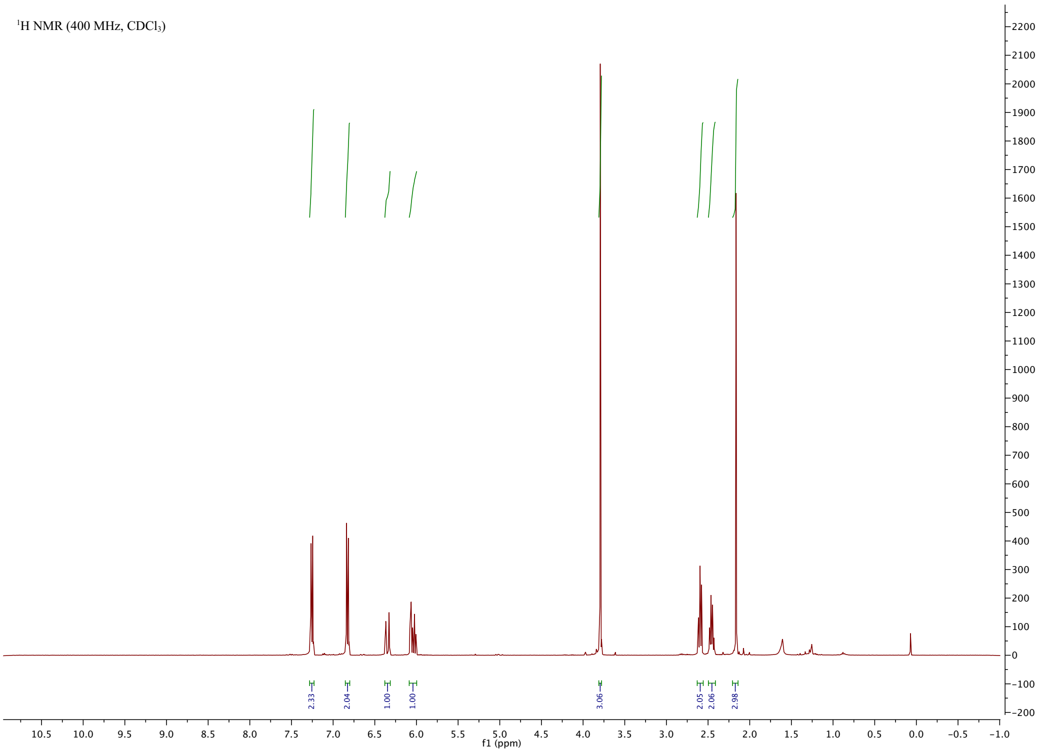
^{1}H NMR (400 MHz, CDCl$_3$)
2.33
2.04
1.00
1.00
3.06
2.05
2.06
2.98
f1 (ppm)

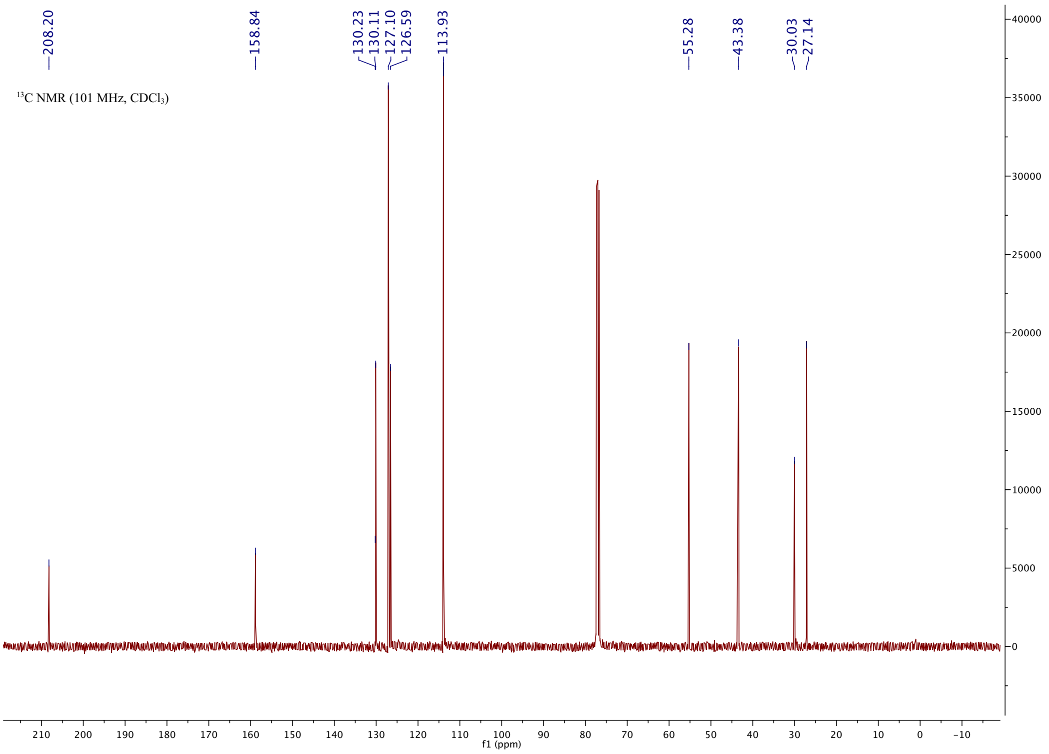
208.20
158.84
130.23
130.11
127.10
126.59
113.93
55.28
43.38
30.03
27.14
^{13}C NMR (101 MHz, CDCl$_3$)
f1 (ppm)

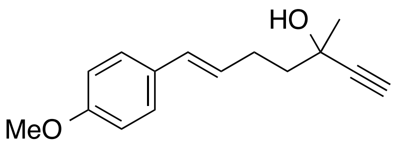

26f

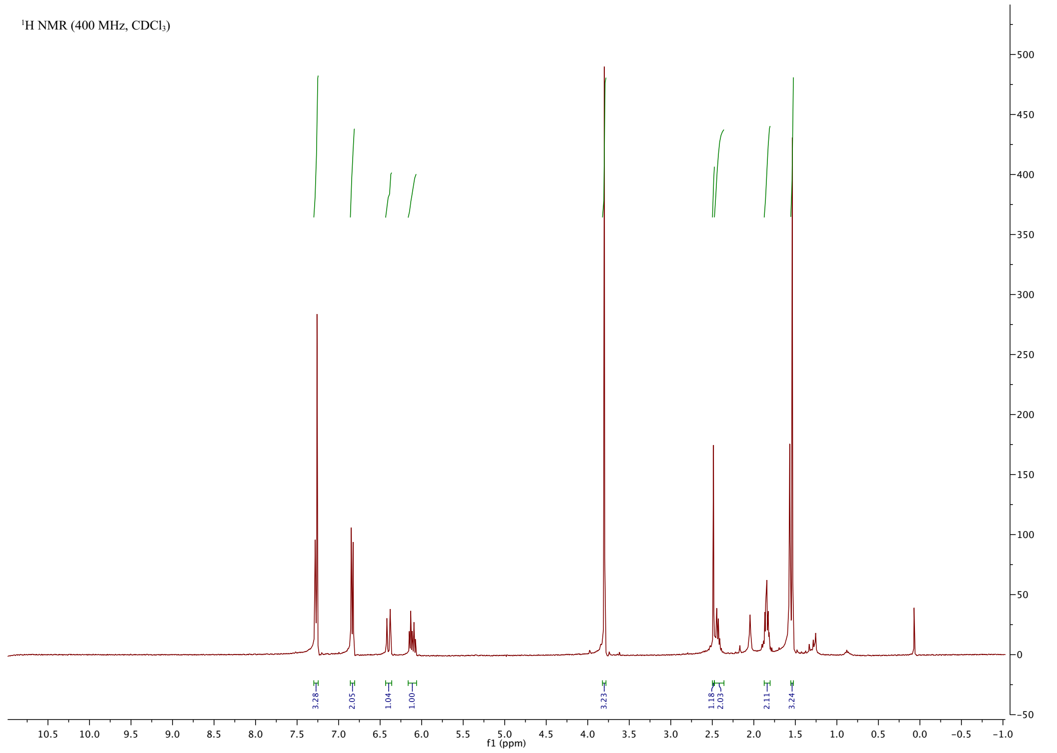

^{1}H NMR (400 MHz, CDCl$_3$)

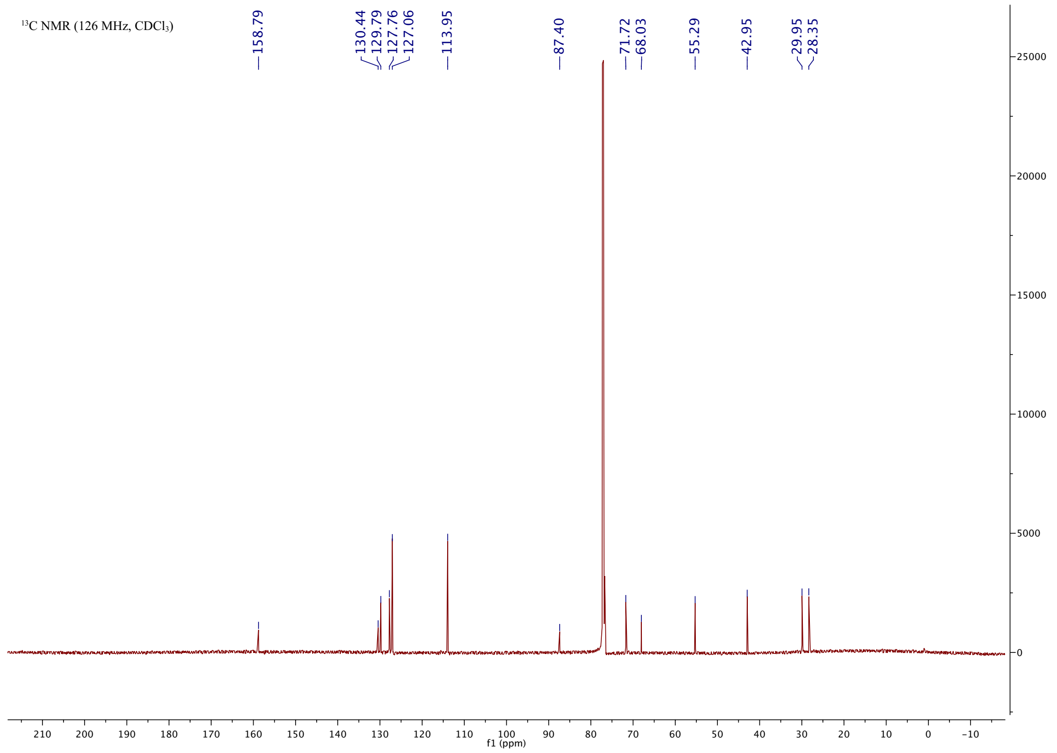

^{13}C NMR (126 MHz, CDCl$_3$)
158.79
130.44
129.79
127.76
127.06
113.95
87.40
71.72
68.03
55.29
42.95
29.95
28.35
f1 (ppm)

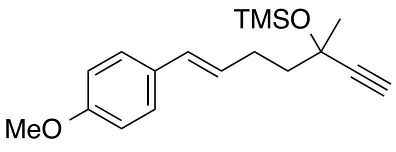

26g

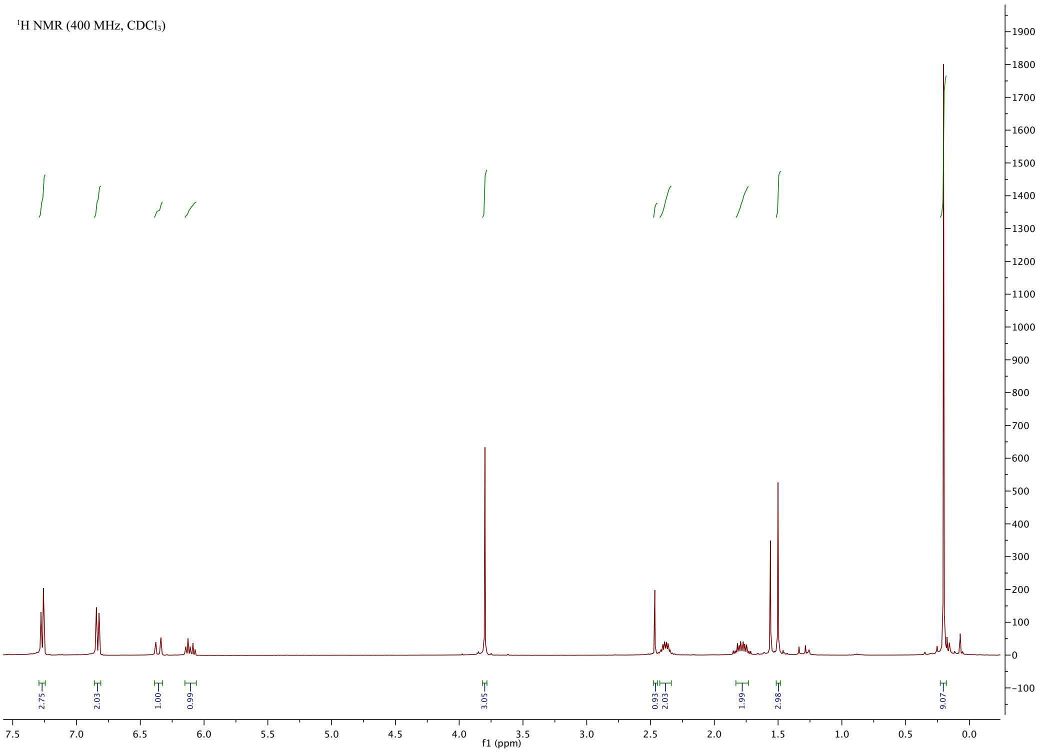

^{1}H NMR (400 MHz, CDCl$_3$)
f1 (ppm)

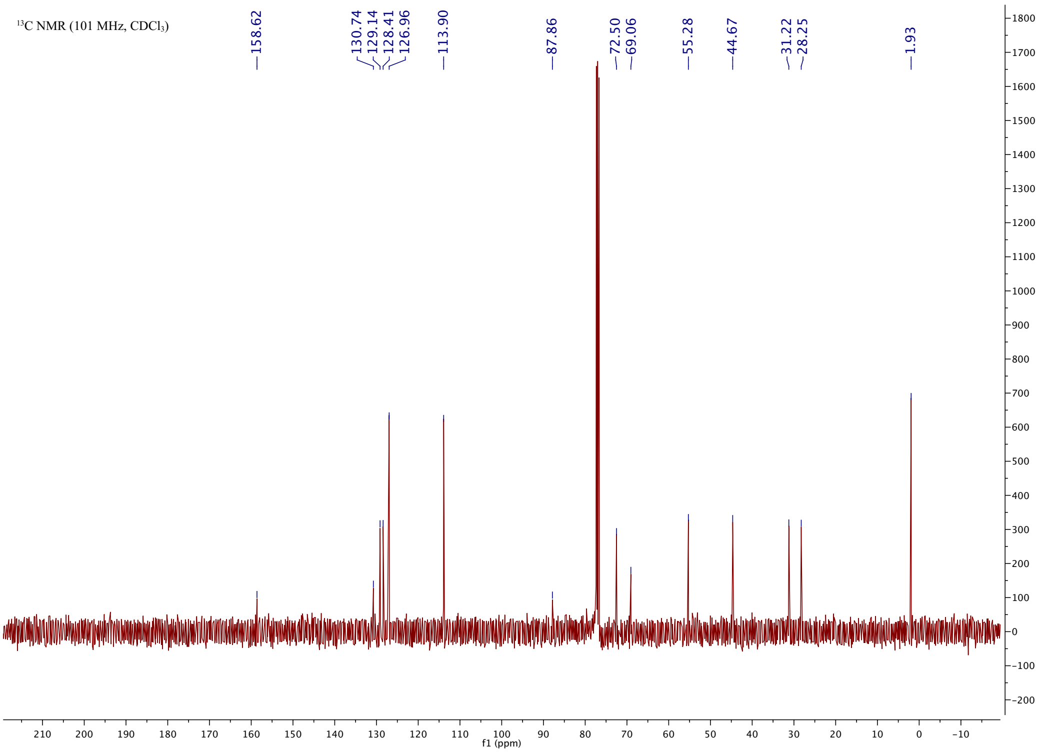

^{13}C NMR (101 MHz, CDCl$_3$)
158.62
130.74
129.14
128.41
126.96
113.90
87.86
72.50
69.06
55.28
44.67
31.22
28.25
1.93
f1 (ppm)

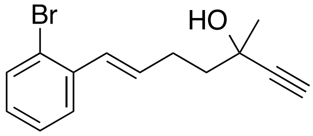

26h

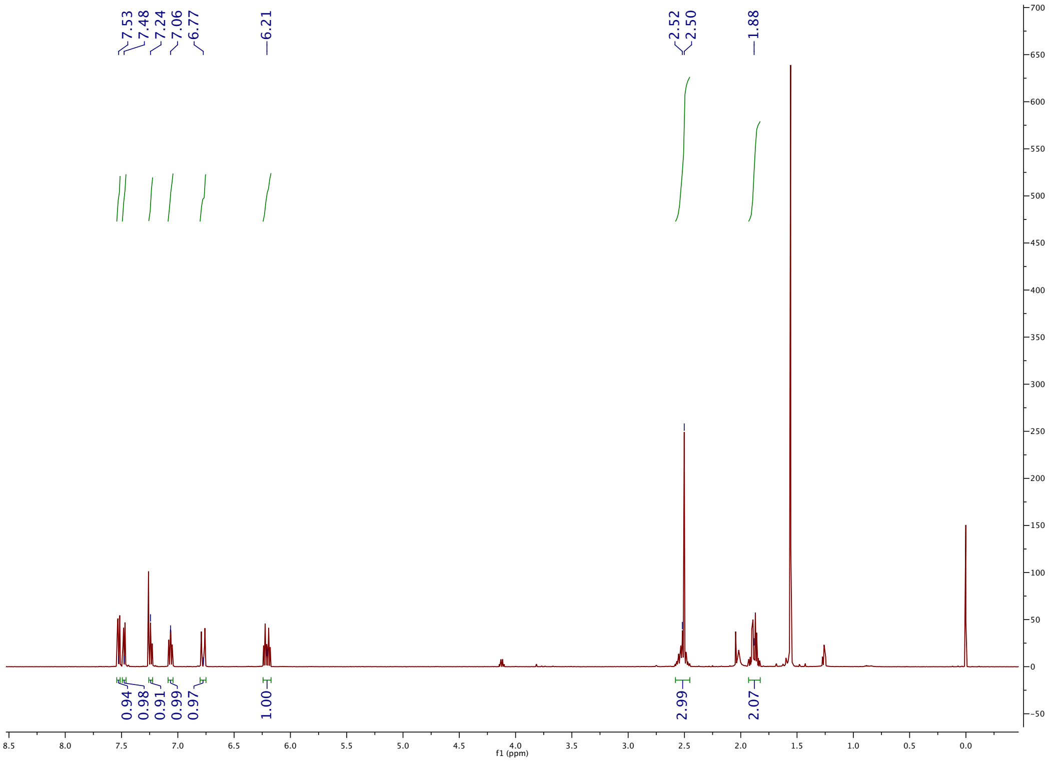

7.53
7.48
7.24
7.06
6.77
6.21
2.52
2.50
1.88
0.94
0.98
0.91
0.99
0.97
1.00
2.99
2.07
f1 (ppm)

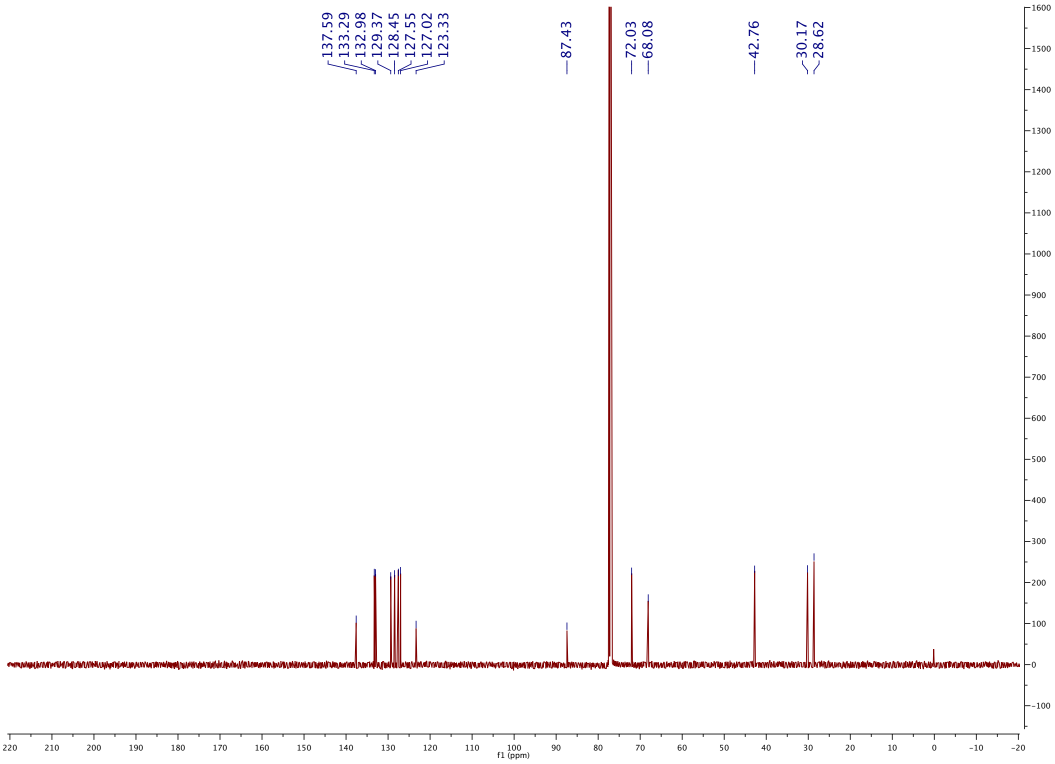

137.59
133.29
132.98
129.37
128.45
127.55
127.02
123.33
87.43
72.03
68.08
42.76
30.17
28.62
f1 (ppm)

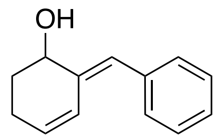

22

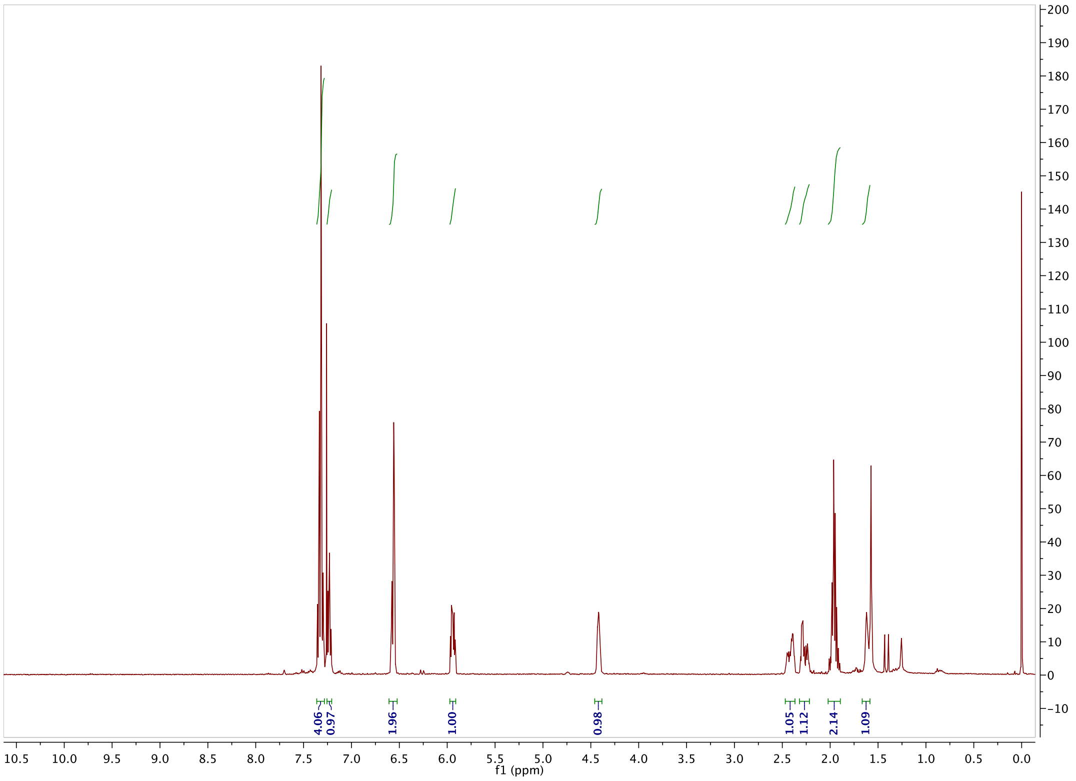

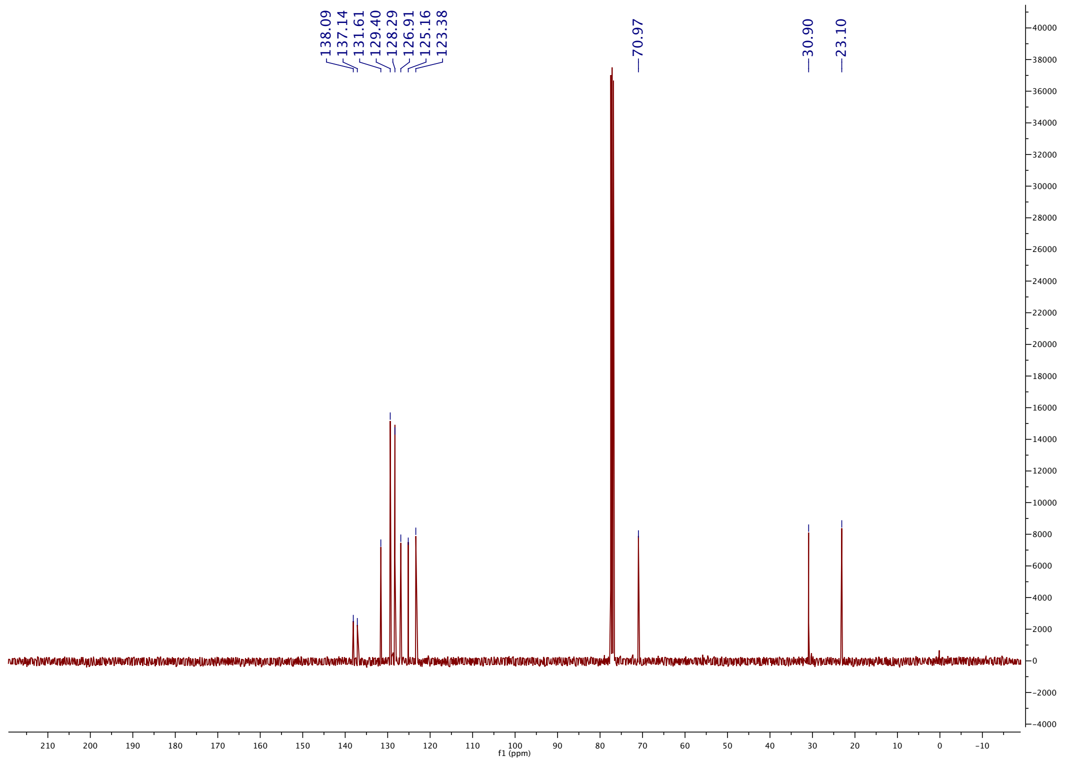

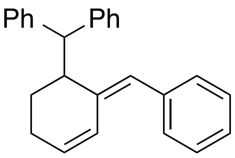

Ph
Ph

23

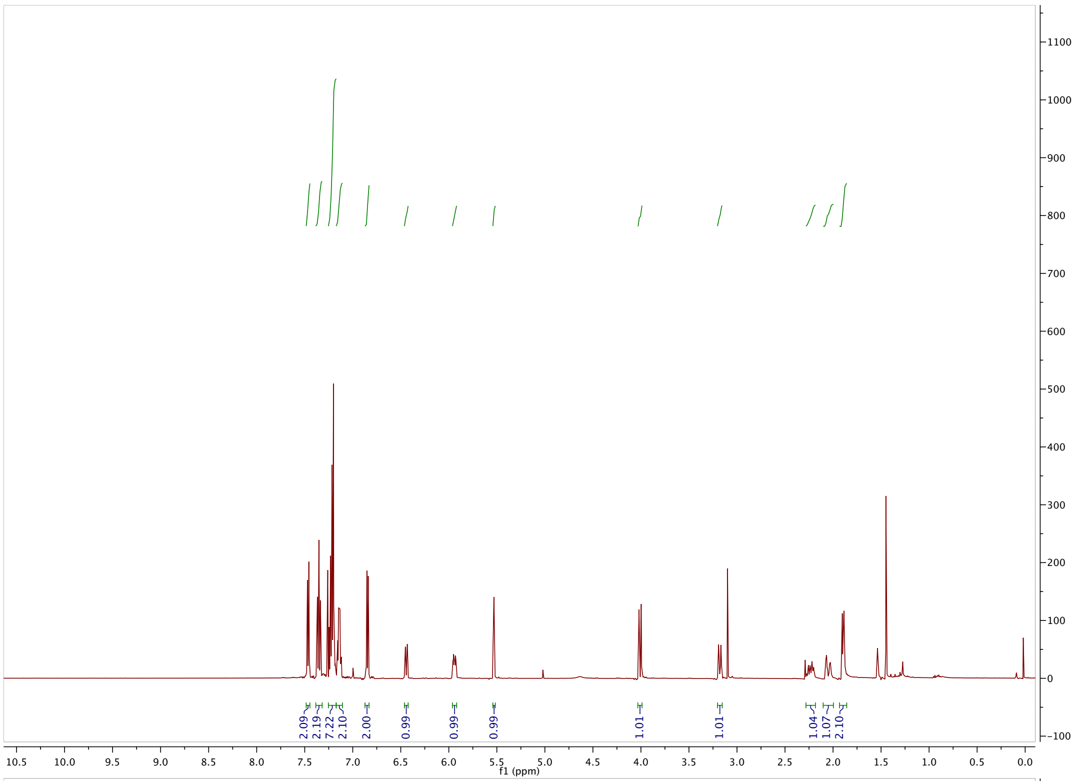

2.09
2.19
7.22
2.10
2.00
0.99
0.99
0.99
1.01
1.01
1.04
1.07
2.10
f1 (ppm)

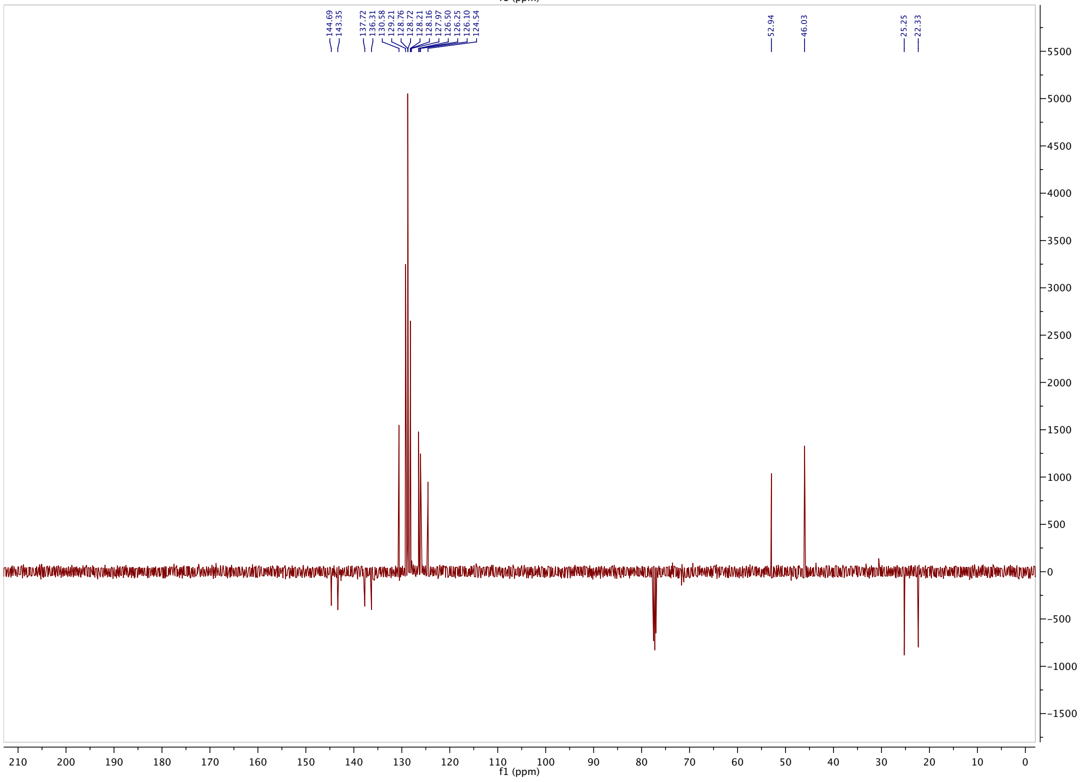

144.69
143.35
137.72
136.31
130.58
129.21
128.76
128.72
128.21
128.16
127.97
126.50
126.25
126.10
124.54
52.94
46.03
25.25
22.33
f1 (ppm)

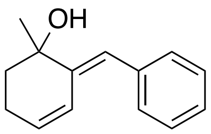

27a

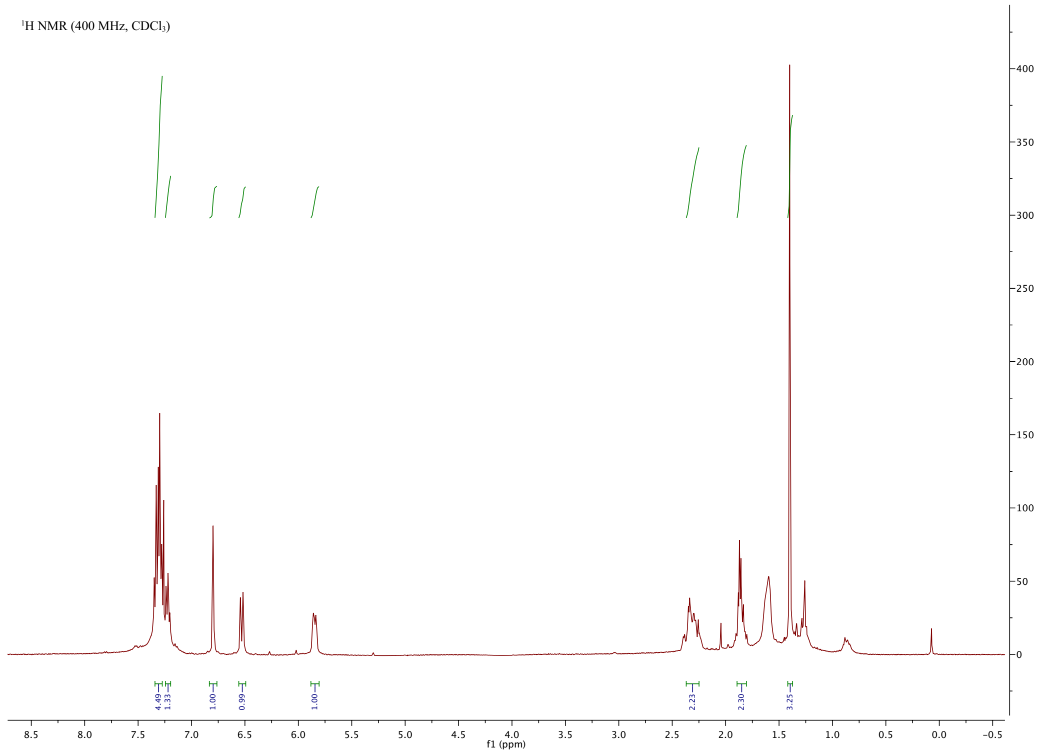

^{1}H NMR (400 MHz, $CDCl_3$)
4.49
1.33
1.00
0.99
1.00
2.23
2.30
3.25
f1 (ppm)

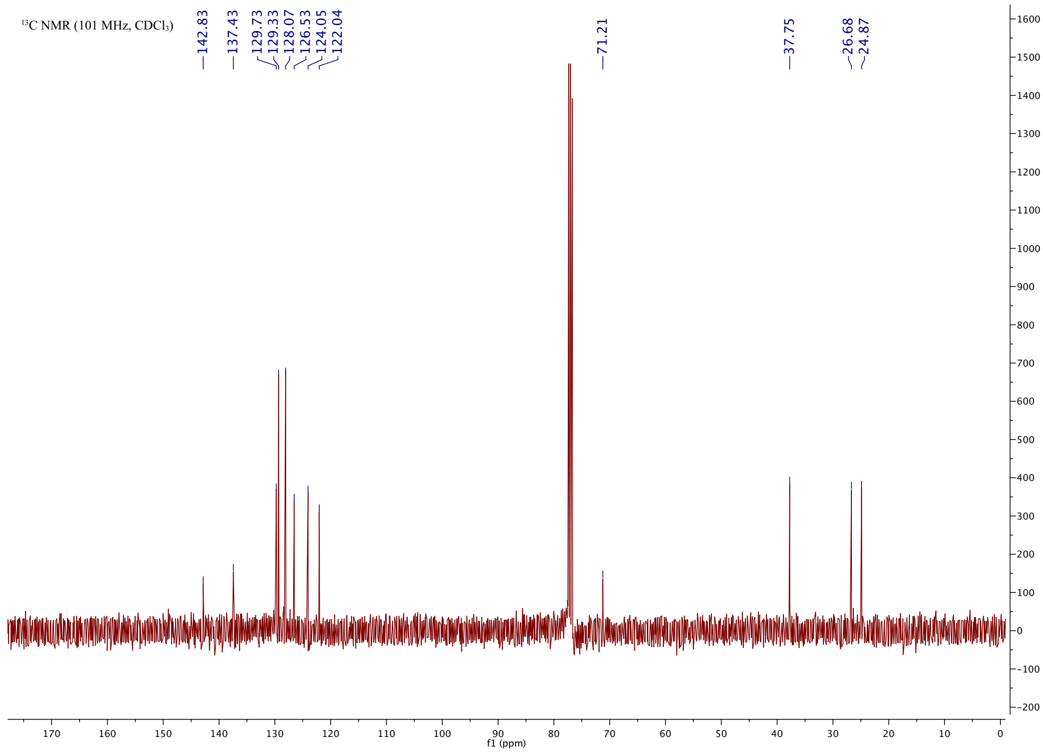

^{13}C NMR (101 MHz, $CDCl_3$)
142.83
137.43
129.73
129.33
128.07
126.53
124.05
122.04
71.21
37.75
26.68
24.87
f1 (ppm)

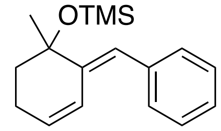

27b

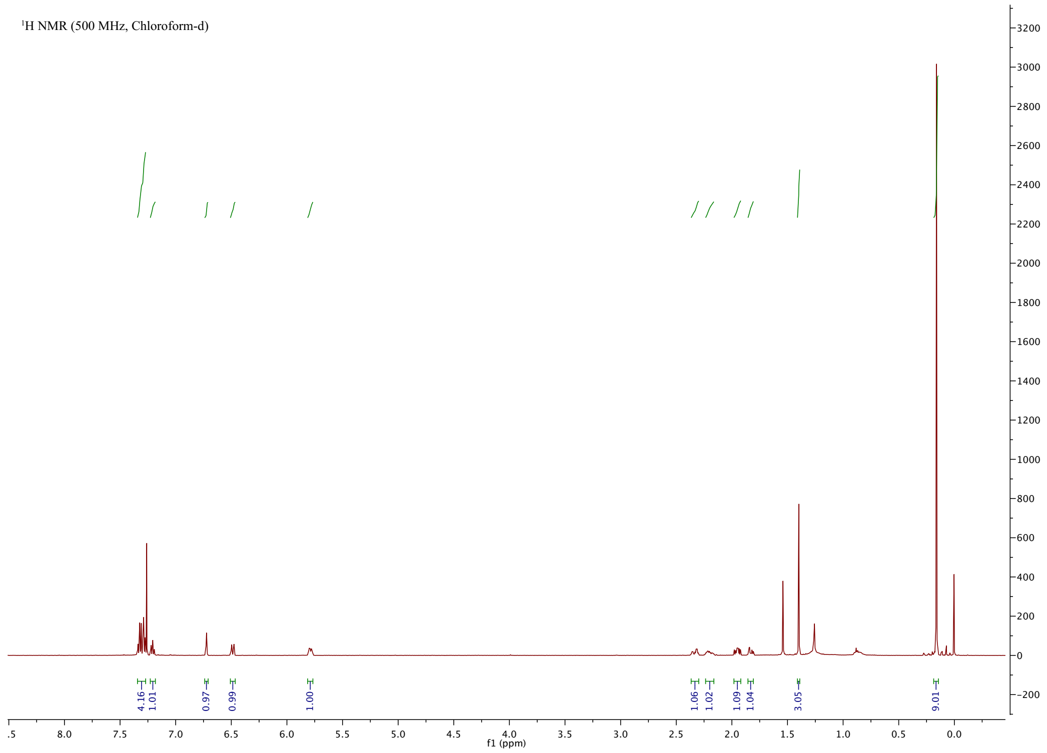

1H NMR (500 MHz, Chloroform-d)
4.16
1.01
0.97
0.99
1.00
1.06
1.02
1.09
1.04
3.05
9.01
f1 (ppm)

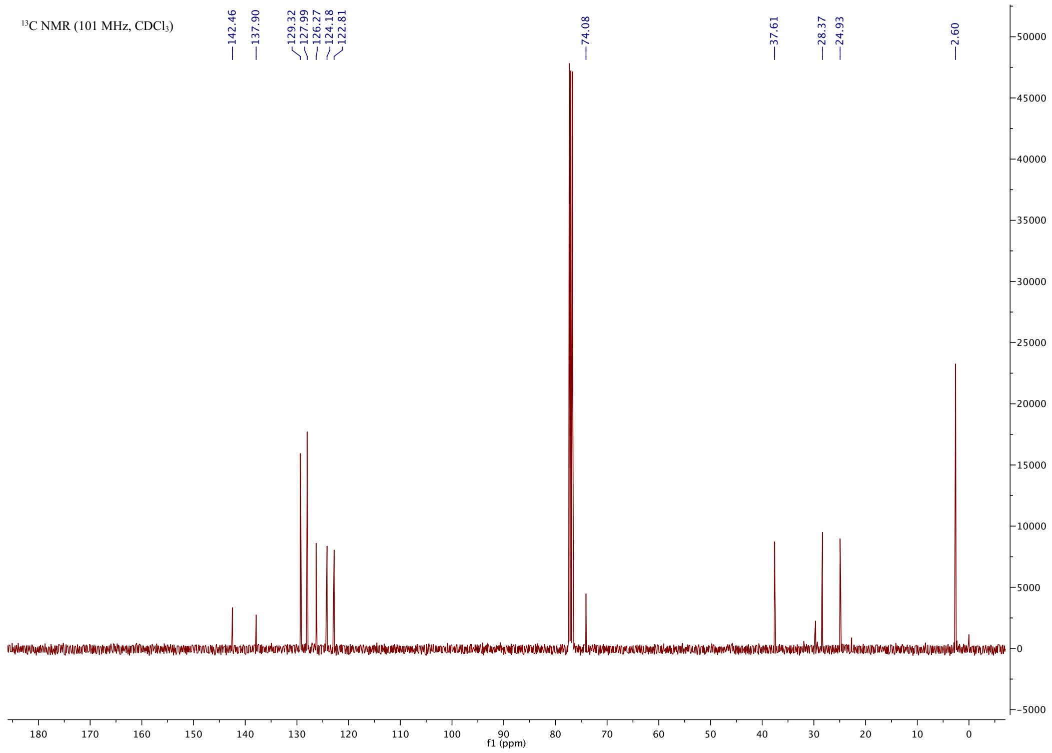

13C NMR (101 MHz, CDCl3)
142.46
137.90
129.32
127.99
126.27
124.18
122.81
74.08
37.61
28.37
24.93
2.60
f1 (ppm)

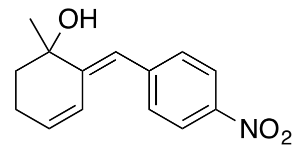

OH
NO2

27c

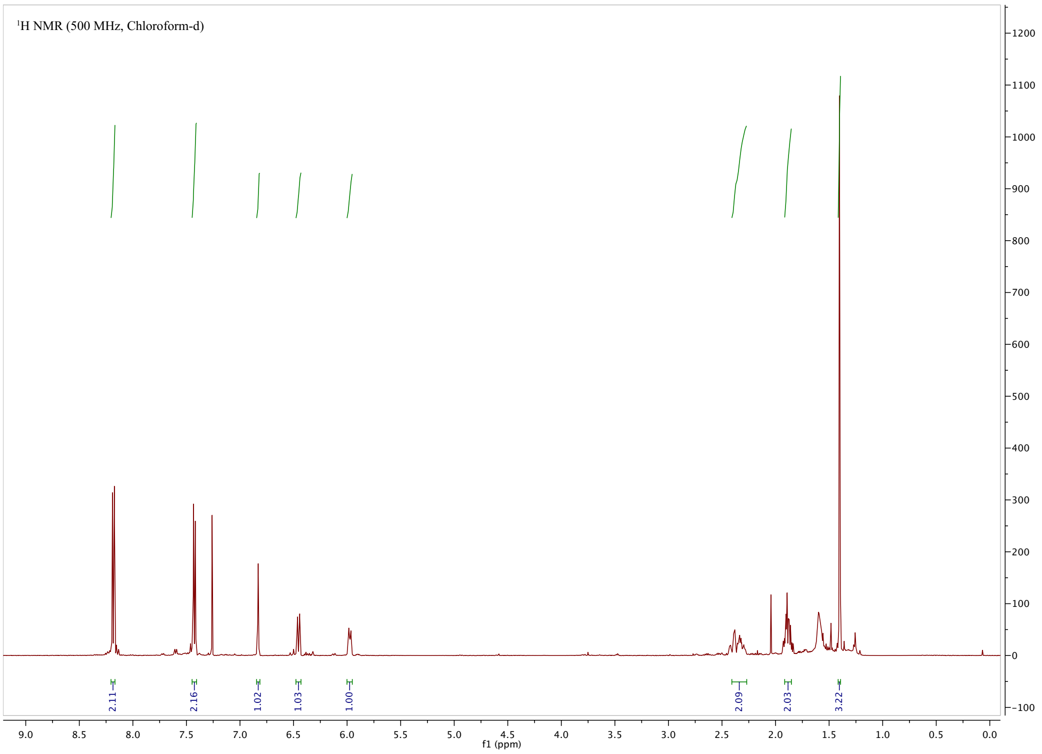

1H NMR (500 MHz, Chloroform-d)
2.11
2.16
1.02
1.03
1.00
2.09
2.03
3.22
f1 (ppm)

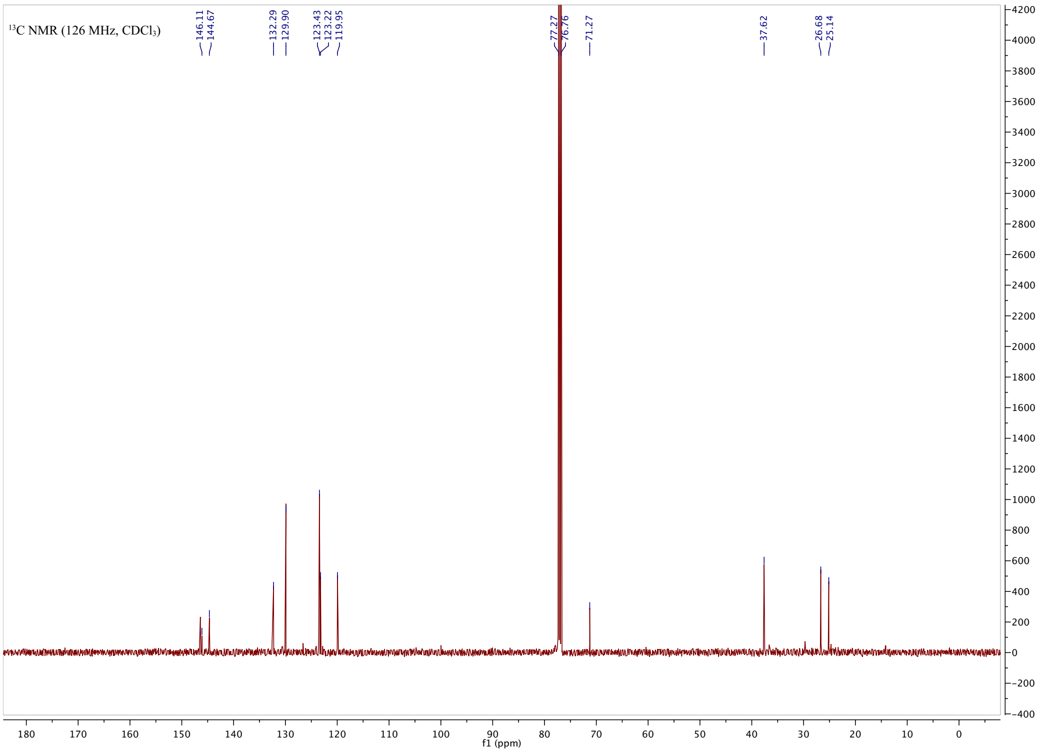

13C NMR (126 MHz, CDCl3)
146.11
144.67
132.29
129.90
123.43
123.22
119.95
77.27
76.76
71.27
37.62
26.68
25.14
f1 (ppm)

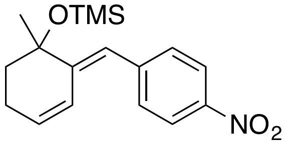
OTMS
NO2

27d

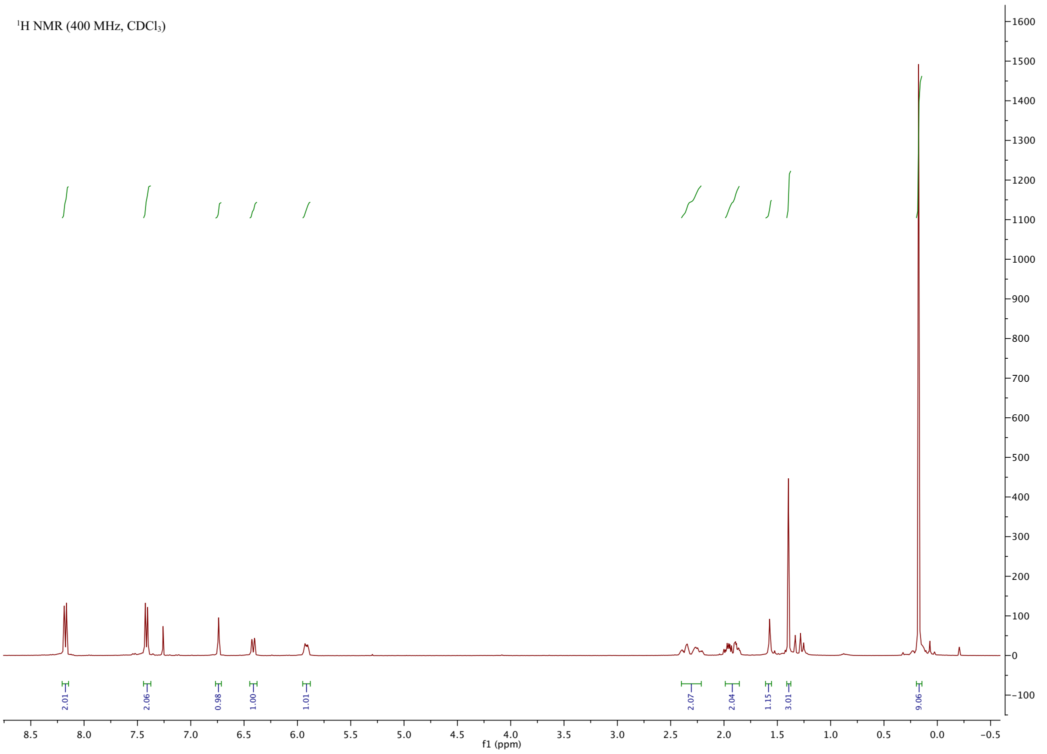
1H NMR (400 MHz, CDCl3)
2.01
2.06
0.98
1.00
1.01
2.07
2.04
1.15
3.01
9.06
f1 (ppm)

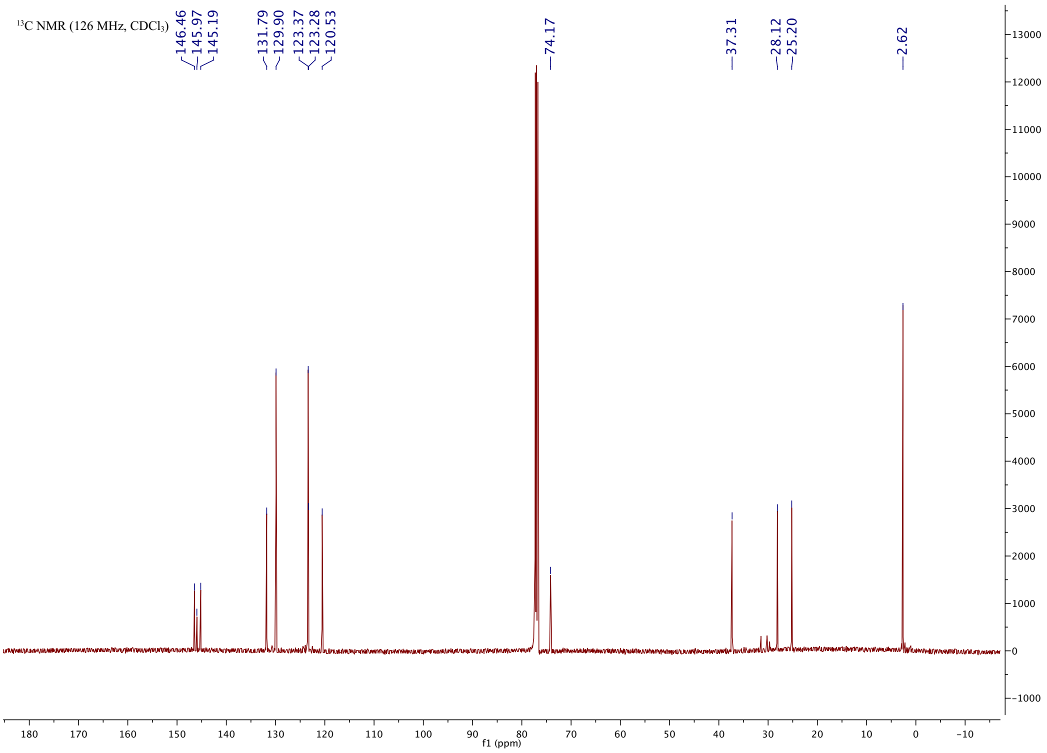
13C NMR (126 MHz, CDCl3)
146.46
145.97
145.19
131.79
129.90
123.37
123.28
120.53
74.17
37.31
28.12
25.20
2.62
f1 (ppm)

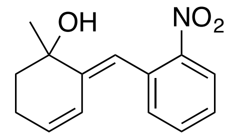
OH
NO2

27e

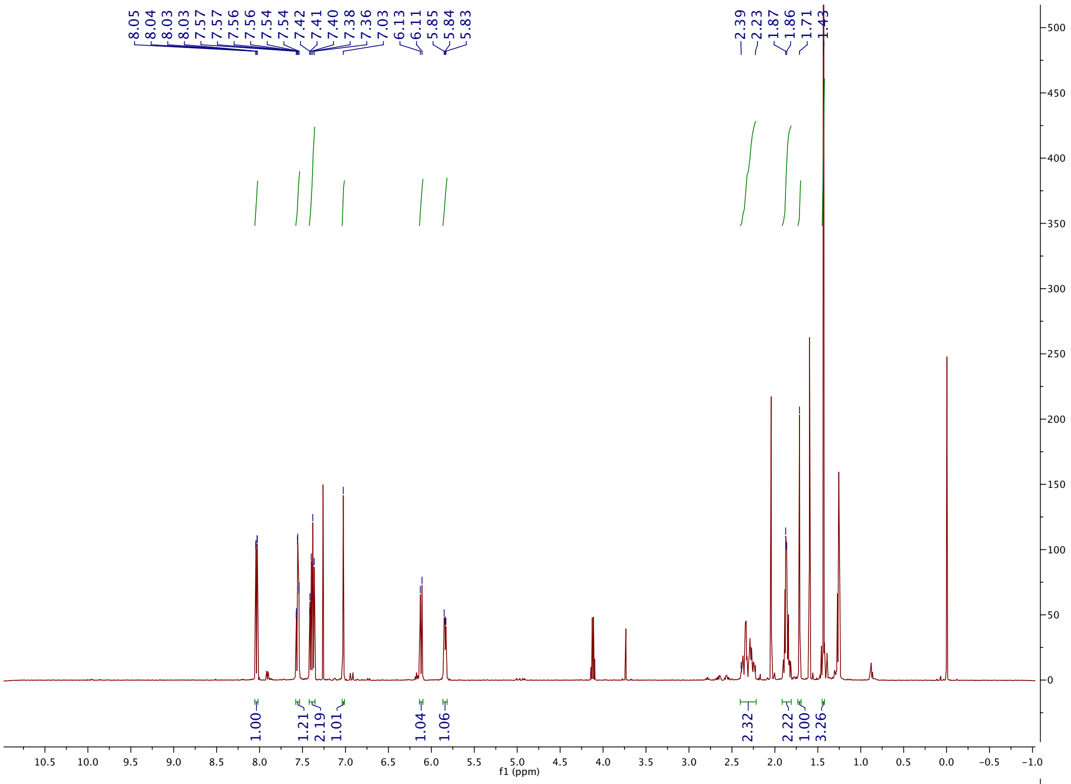
8.05 8.04 8.03 8.03 7.57 7.57 7.56 7.56 7.54 7.54 7.42 7.41 7.40 7.38 7.36 7.03 6.13 6.11 5.85 5.84 5.83
2.39 2.23 1.87 1.86 1.71 1.43
1.00 1.21 2.19 1.01 1.04 1.06 2.32 2.22 1.00 3.26
f1 (ppm)

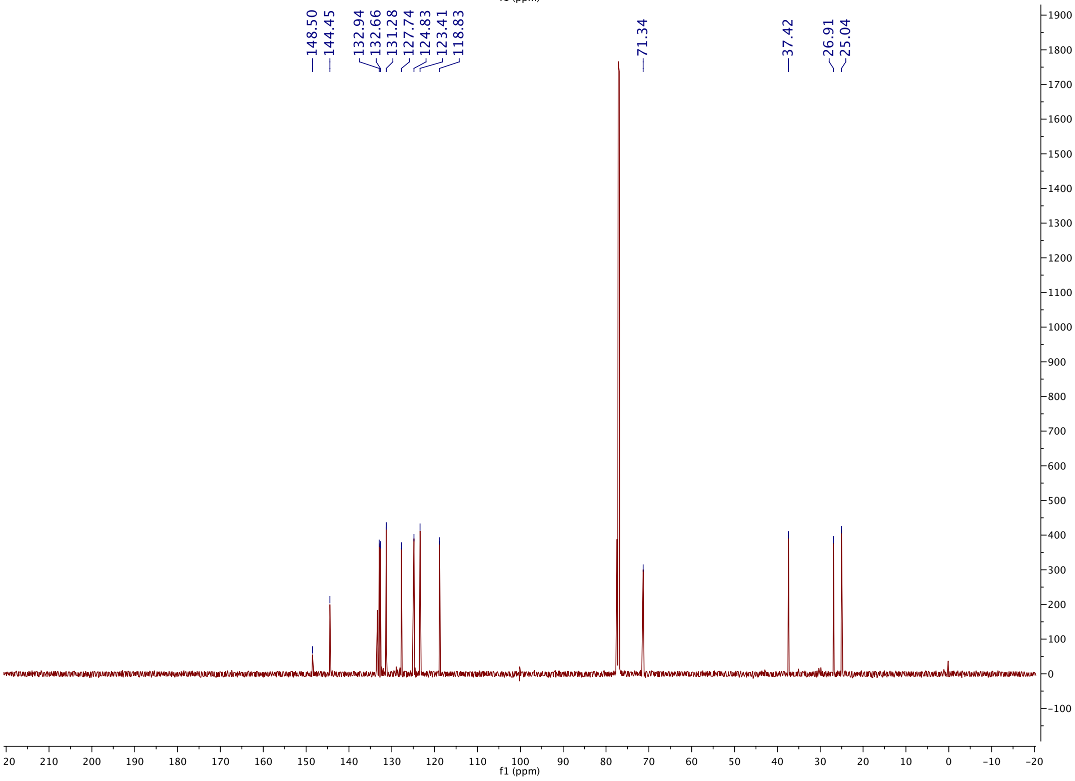
148.50 144.45 132.94 132.66 131.28 127.74 124.83 123.41 118.83
71.34
37.42
26.91 25.04
f1 (ppm)

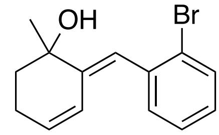
OH
Br

27h

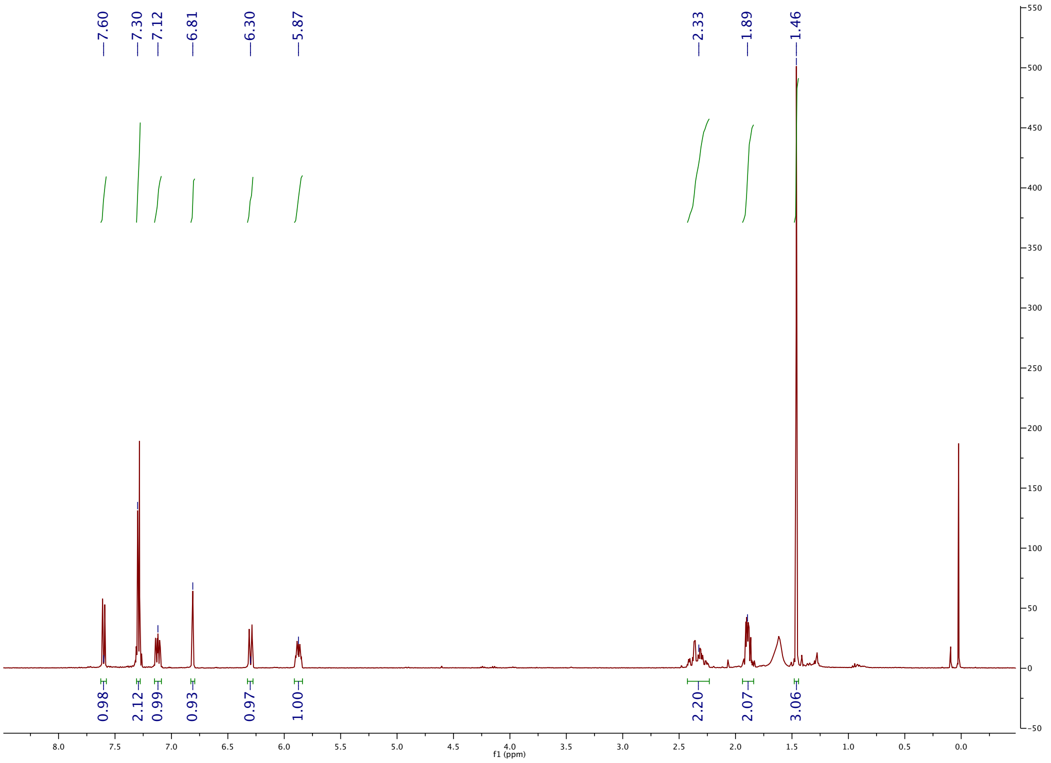
7.60
7.30
7.12
6.81
6.30
5.87
2.33
1.89
1.46
0.98
2.12
0.99
0.93
0.97
1.00
2.20
2.07
3.06
f1 (ppm)

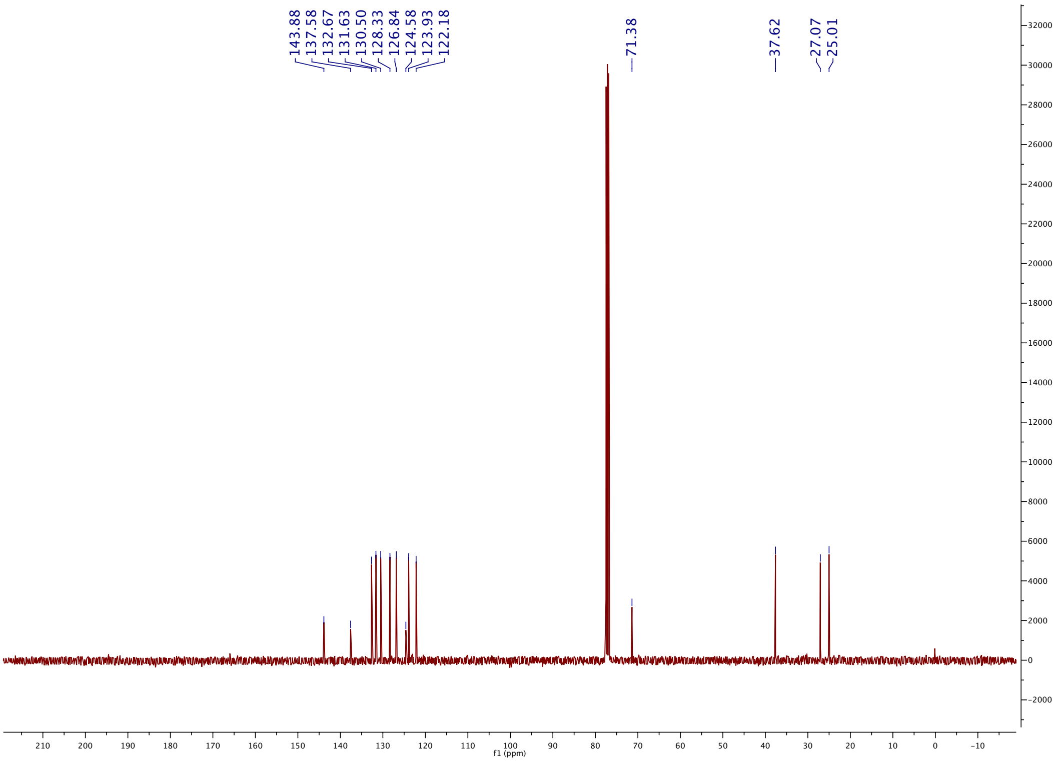
143.88
137.58
132.67
131.63
130.50
128.33
126.84
124.58
123.93
122.18
71.38
37.62
27.07
25.01
f1 (ppm)

9. DFT calculations

All calculations were performed with DFT using the B3LYP functional as implemented in Gaussian 09.[7] The 6-31G(d,p) basis set was used for all atoms except gold, which was treated with SDD and the associated effective core potential[8]. The solvent effect was taken into account using the polarizable continuum model[9] in particular IEF-PCM as implemented in Gaussian 09. Frequency calculations were performed to characterize the stationary points as minima.

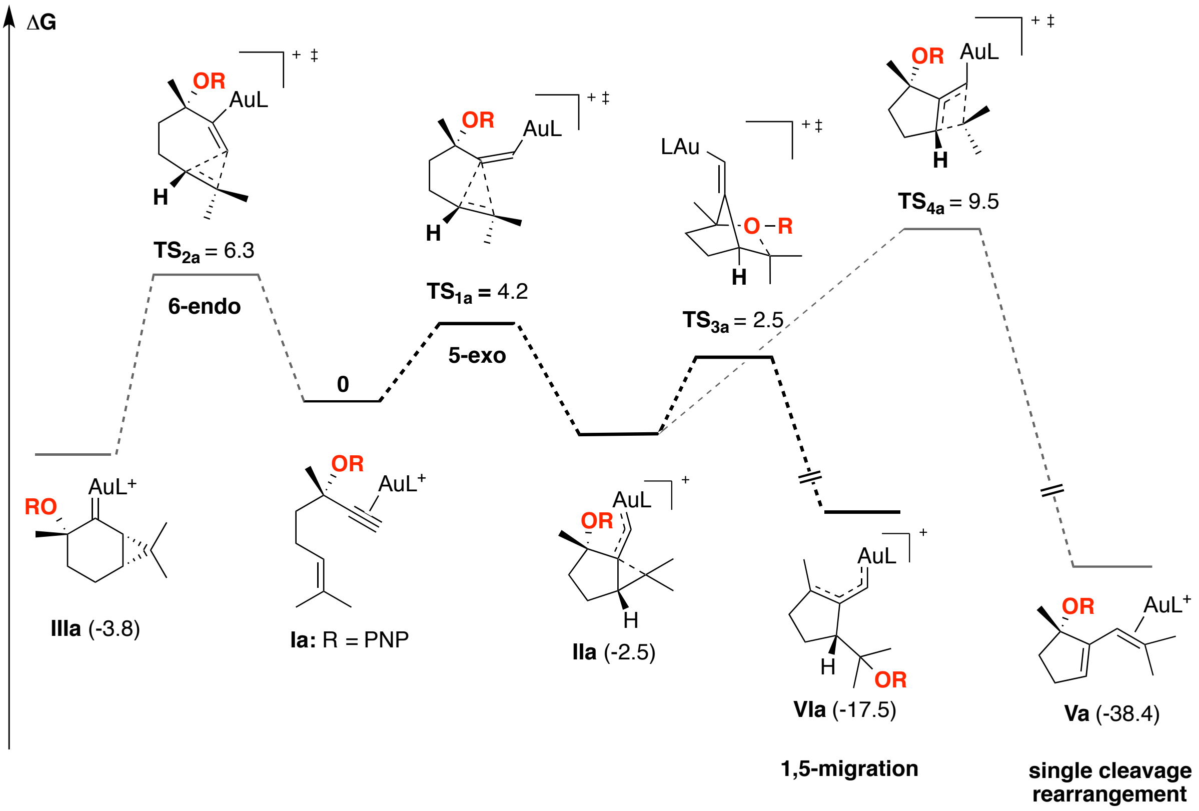

Scheme S1. Reaction pathways and energies for the the cyclization of **Ia** calculated at the B3LYP/6-31G(d,p) (C, H, P, O, N), SDD(Au) level taking into account solvent effect of CH_2Cl_2 (IEF-PCM) and employing PMe_3 as the phosphine. ΔG energies are given in kcal·mol-1.

<u>Cartesian Coordinates (in Å)</u>

Table S6: Optimized geometry for Ia

Free energy G = -1497.830742 Hartree/particle.

C	0.81486500	0.32324500	1.35721700
C	0.83460400	-0.97447100	2.18561000
H	1.33140400	-0.77102700	3.13711800
H	-0.19059700	-1.29422600	2.38791600
H	1.35548700	-1.78965500	1.68410800
O	2.13638900	0.89225800	1.21903900
C	0.02279200	1.40915600	2.12985100
H	-1.00394400	1.04769500	2.25732300
H	0.47648100	1.43501400	3.12562700
C	0.02364700	2.83625900	1.54414600
H	-0.22826200	3.50393900	2.38020300
H	1.04044800	3.10019500	1.24601400

C	-0.97013200	3.07716800	0.43747400
H	-1.99856900	2.81336400	0.69347300
C	-0.75929500	3.62099000	-0.77294200
C	0.58316800	4.06731600	-1.29747900
H	1.40904100	3.85482500	-0.61677500
H	0.80067800	3.57932800	-2.25638300
H	0.57869600	5.14692500	-1.49584700
C	-1.90621200	3.83708300	-1.73196400
H	-1.74294100	3.28919700	-2.66988700
H	-2.86185900	3.51547900	-1.30797100
H	-1.99496700	4.89618900	-2.00691900
C	0.26297700	0.08186600	-0.01130300
C	0.04115700	-0.10373300	-1.20708100
H	0.05529300	-0.19032600	-2.27575000
P	-4.19148500	-1.23404300	0.02207000
C	-4.65029900	-2.67561500	-1.01181600
H	-4.51462000	-2.42855500	-2.06705200
H	-4.01193700	-3.52576600	-0.76171400
H	-5.69629700	-2.93946700	-0.83172500
C	-4.55464600	-1.72060900	1.75140100
H	-3.90485700	-2.54735400	2.04615200
H	-4.37650800	-0.87308100	2.41703900
H	-5.59980600	-2.03275300	1.83276400
C	-5.40063900	0.07947500	-0.38928000
H	-5.23249700	0.94722500	0.25229300
H	-5.27660200	0.37999600	-1.43205200
H	-6.41771100	-0.29320000	-0.23707800
Au	-1.97559300	-0.58524300	-0.28935700
C	3.23260200	0.25450400	0.71443900
C	4.44292900	0.92725300	0.96628200
C	3.23423300	-0.93870800	-0.02497900
C	5.64047300	0.41742200	0.49458200
H	4.41622300	1.84830700	1.53747000
C	4.43842700	-1.45269600	-0.49652800
H	2.31957300	-1.46776000	-0.25069500
C	5.62757900	-0.77582300	-0.23441000
H	6.57815000	0.92378400	0.68420100
H	4.45936800	-2.37162000	-1.06831400
N	6.88317400	-1.32204500	-0.73313000
O	6.84625700	-2.37705400	-1.37322400
O	7.92281400	-0.70245200	-0.49116200

Table S7: Optimized geometry for TS_{1a}

Free energy G = -1497.824114 Hartree/particle.

C	-1.13405200	-1.68214200	1.41242600
C	-0.31641100	-0.84361200	2.42016800
H	-0.91754100	-0.73220200	3.32603300
H	0.61091500	-1.36214000	2.66909500
H	-0.07264700	0.14582700	2.03351000
O	-2.43688300	-1.09751000	1.17995300
C	-1.46292400	-3.06013300	2.02577800
H	-0.51594500	-3.55610000	2.2614730
H	-2.00034600	-2.89817900	2.96435500
C	-2.29080500	-3.91276600	1.04844700
H	-2.35764800	-4.93286000	1.44343300
H	-3.30683300	-3.52043200	0.98969800
C	-1.62314500	-3.93343300	-0.29558500
H	-0.70139400	-4.51193100	-0.34609200
C	-2.08576600	-3.40600000	-1.46473500
C	-3.38669200	-2.66912900	-1.62470800

H	-3.92167200	-2.51766100	-0.68847300
H	-3.21420700	-1.68949800	-2.08524600
H	-4.03676600	-3.22417400	-2.31231400
C	-1.29535300	-3.58184700	-2.73122500
H	-1.05457200	-2.60904800	-3.17919300
H	-0.37069100	-4.14206500	-2.57327900
H	-1.89405800	-4.12063200	-3.47602600
C	-0.36112300	-1.87763200	0.15787000
C	0.61414300	-1.71938800	-0.63915400
H	0.83682900	-2.24522000	-1.56088300
P	3.76848100	1.34870000	0.09460200
C	3.12450100	3.04612000	0.36218500
H	2.52343800	3.34965900	-0.49795100
H	2.49592900	3.06237500	1.25557600
H	3.95433700	3.74719400	0.49007800
C	4.84531000	1.00518600	1.54030900
H	4.24230800	0.99337600	2.45118600
H	5.32145200	0.02948400	1.41922800
H	5.61597800	1.77666100	1.62517800
C	4.90901200	1.48436600	-1.33586400
H	5.39739900	0.52258200	-1.50804200
H	4.34525300	1.75911700	-2.23031600
H	5.66795500	2.24653000	-1.13679100
Au	2.08162600	-0.24299000	-0.21470200
C	-2.67838300	0.17403500	0.75158900
C	-4.00130400	0.60440800	0.96649700
C	-1.76050700	1.02308400	0.11286700
C	-4.40482600	1.86492900	0.56006600
H	-4.69255000	-0.06955400	1.45981400
C	-2.16500600	2.29154100	-0.29215400
H	-0.74498500	0.71156700	-0.08541700
C	-3.47685000	2.70344000	-0.06544500
H	-5.41798700	2.20896900	0.72376600
H	-1.47174700	2.96021300	-0.78656800
N	-3.88957000	4.03433700	-0.49067400
O	-3.04993700	4.75960900	-1.03261200
O	-5.05915100	4.37256300	-0.28793000

Table S8: Optimized geometry for IIa

Free energy G = -1497.834746 Hartree/particle.

C	-0.46037500	4.32710200	-1.20903900
C	0.78028600	3.94621900	-0.40061000
C	-0.58671200	1.87727500	-0.87495200
C	-1.44750200	3.14992400	-1.10367800
H	-0.89591900	5.26914900	-0.86440300
H	-0.14834800	4.47584400	-2.24627800
H	-2.05096700	3.07359600	-2.01031500
H	-2.13802300	3.28594100	-0.26971800
C	-0.34803600	1.10040500	-2.17821100
H	0.32996100	0.26101500	-2.0058830
H	-1.28422700	0.71965200	-2.59067000
H	0.10491600	1.76296000	-2.92114400
C	0.77653500	2.36945400	-0.33659700
C	1.84001000	1.59250000	-0.00121200
H	2.71286700	2.14423500	0.34651700
P	2.48689300	-2.77859900	-0.04014800
C	1.67814600	-3.68000800	-1.42268100
H	1.90520100	-4.74860100	-1.36728600
H	0.59614000	-3.53762300	-1.36845300
H	2.03611100	-3.28360400	-2.37600800

C	4.26699400	-3.21238300	-0.18286000
H	4.66891000	-2.80080200	-1.11191300
H	4.81654500	-2.78061100	0.65706000
H	4.39838300	-4.29825600	-0.18140400
C	1.91663800	-3.64762200	1.47546700
H	2.41493700	-3.22266800	2.35014900
H	0.83792200	-3.51553700	1.58813600
H	2.14530400	-4.71539600	1.41101900
Au	2.08536300	-0.44578000	-0.03307400
O	-1.12114900	1.00532700	0.16100300
C	0.72986600	3.84710600	1.04630900
C	1.98145400	4.07994700	1.82860500
H	1.86461600	5.09672700	2.23561900
H	2.07550100	3.40838500	2.68502200
H	2.88986600	4.07372400	1.22705800
C	-0.53591400	3.78083000	1.85166800
H	-1.11168600	2.87583800	1.65497200
H	-0.30832500	3.81244500	2.91787200
H	-1.16987600	4.64007500	1.60003400
H	1.71646500	4.35480000	-0.77630200
C	-2.30512600	0.34247200	0.08530900
C	-3.31770200	0.55609800	-0.86607200
C	-2.49604800	-0.61517600	1.10232400
C	-4.49264800	-0.18584100	-0.80296200
H	-3.21159400	1.29291700	-1.64904100
C	-3.66604000	-1.35149300	1.16669600
H	-1.70806300	-0.76083600	1.83303500
C	-4.65947700	-1.13243700	0.20637700
H	-5.28100400	-0.03191600	-1.52873800
H	-3.82096000	-2.09026100	1.94261300
N	-5.89131200	-1.90503200	0.26385100
O	-6.75261200	-1.69552500	-0.59606400
O	-6.01607400	-2.73437100	1.17047200

Table S9: Optimized geometry for TS_{3a}

Free energy G = -1497.826759 Hartree/particle.

C	-1.34208800	-4.19587700	0.57980000
C	-1.27804700	-3.30555100	-0.67344700
C	-0.98706800	-1.80110100	1.15371200
C	-1.27114300	-3.19517500	1.76799800
H	-2.21832100	-4.84594200	0.61498700
H	-0.46414000	-4.84571600	0.57692000
H	-0.45864900	-3.45771500	2.44928700
H	-2.19275000	-3.15719200	2.35415000
C	-0.26995900	-0.86918800	2.11463500
H	-0.07863900	0.11878700	1.69604400
H	-0.86359500	-0.75828800	3.02700600
H	0.69230400	-1.31144700	2.38143700
C	-0.36535000	-2.12852800	-0.19789000
C	0.72937400	-1.67791500	-0.82046400
H	0.91933400	-2.15466700	-1.78637400
P	3.83706000	1.31234600	0.17695500
C	5.06584700	1.51280000	-1.17646400
H	5.81815800	2.25902400	-0.90510100
H	5.55812300	0.55620900	-1.36800000
H	4.55494700	1.83095600	-2.08856300
C	3.21676300	3.01673700	0.48194900
H	2.66828900	3.36876300	-0.39517700
H	2.53868500	3.01089600	1.33890100
H	4.04848800	3.69782000	0.68468400

C	4.85223600	0.92660300	1.66144700
H	4.20787500	0.88171100	2.54289700
H	5.33487200	-0.04517800	1.53194300
H	5.61827100	1.69274500	1.81313600
Au	2.14688200	-0.27506000	-0.29849500
O	-2.36702100	-1.30556800	0.83939600
C	-2.56515400	-2.74757600	-1.15422100
C	-2.54915200	-1.82067800	-2.30312500
H	-3.22584600	-2.20094900	-3.07910200
H	-2.98615900	-0.86142300	-1.98912200
H	-1.55343400	-1.64473800	-2.70655400
C	-3.88376000	-3.23062700	-0.69633000
H	-3.90971900	-3.49409600	0.35952000
H	-4.68833300	-2.53691100	-0.94657600
H	-4.06467400	-4.15915300	-1.26610300
H	-0.81415000	-3.78117100	-1.54793900
C	-2.66859900	0.00532000	0.58196100
C	-1.81938400	0.90326000	-0.08652700
C	-3.95772600	0.40521500	0.97435400
C	-2.25578700	2.20072400	-0.33575900
H	-0.83551600	0.59362200	-0.41399400
C	-4.39567000	1.69626200	0.71806900
H	-4.59415700	-0.30753800	1.48635700
C	-3.53413000	2.58333000	0.06854400
H	-1.61923100	2.91178400	-0.84682800
H	-5.38201400	2.02481400	1.01917200
N	-3.98641000	3.94727200	-0.19892900
O	-3.20346500	4.71883600	-0.75821900
O	-5.12850900	4.25678200	0.14792300

Table S10: Optimized geometry for VIa

Free energy G = -1497.824083 Hartree/particle.

C	1.29637200	3.54189000	0.59010000
C	0.90781100	2.59181700	-0.57407100
C	-0.20152800	1.85658900	1.43673600
C	0.86748200	2.79881200	1.86816200
H	2.35455200	3.80169400	0.60703000
H	0.72922100	4.47311000	0.50155500
H	0.54009200	3.44101400	2.69330200
H	1.68594500	2.18002900	2.26820400
C	-1.05369900	1.18307900	2.43790900
H	-1.67140800	0.38817200	2.02000000
H	-0.42564900	0.79262700	3.24722600
H	-1.70758200	1.93410100	2.90304400
C	-0.24379600	1.77171100	0.03214300
C	-1.24690800	1.14594900	-0.69917300
H	-1.15707500	1.35348900	-1.77007700
P	-4.73226900	-1.42753600	0.12027000
C	-5.54099100	-1.18145700	1.75137300
H	-6.40530800	-1.84400000	1.85294100
H	-4.82585100	-1.39575600	2.54925700
H	-5.86932600	-0.14344000	1.84429200
C	-6.07540600	-1.19896600	-1.11220400
H	-6.41665400	-0.16112200	-1.09549300
H	-5.69626000	-1.42650200	-2.11136800
H	-6.91680100	-1.86003900	-0.88494600
C	-4.33487200	-3.22027700	0.05616300
H	-3.91274200	-3.46564000	-0.92130700
H	-3.59805400	-3.45928600	0.82671900
H	-5.23701200	-3.81623800	0.22102100

Au	-2.84996300	-0.03044300	-0.25038100
O	2.58773500	1.05553500	0.11940500
C	2.08722900	1.71497600	-1.09728500
C	1.62598800	0.66168900	-2.10966000
H	1.16869800	1.14566300	-2.97782200
H	2.47812900	0.07890400	-2.46914600
H	0.90342800	-0.03398600	-1.67468100
C	3.17148700	2.59310100	-1.73393800
H	3.56703600	3.33506300	-1.03735500
H	4.00462400	1.98237200	-2.09149800
H	2.75481400	3.12164100	-2.59623100
H	0.54893100	3.15872000	-1.43873800
C	3.68514400	0.23497200	0.08047000
C	4.96070000	0.76357300	0.33163300
C	3.52020500	-1.14696700	-0.10029400
C	6.06701000	-0.07621400	0.38294200
H	5.07137000	1.82881800	0.49814000
C	4.62131100	-1.99394300	-0.05054800
H	2.52676300	-1.54759700	-0.26597200
C	5.88295100	-1.44584400	0.18605000
H	7.05911800	0.31105100	0.57639000
H	4.51609800	-3.06263700	-0.18667700
N	7.04376000	-2.33328800	0.24055700
O	8.14953200	-1.83034300	0.45391400
O	6.85982400	-3.54095800	0.06984300

Table S11: Optimized geometry for TS_{2a}

Free energy G = -1497.820742 Hartree/particle.

C	1.67317000	0.99020200	-1.58233700
C	0.85920300	0.57980700	-2.81038400
H	0.91485500	1.36196800	-3.57249600
H	1.26748500	-0.34379400	-3.23017500
H	-0.18849300	0.40182300	-2.55734100
O	1.18943100	2.24097900	-1.01121500
C	3.12512400	1.29862300	-1.98328300
H	3.52761600	0.42457200	-2.50645900
H	3.07976800	2.11651700	-2.70883500
C	4.05840100	1.70673800	-0.83071500
H	4.97586500	2.10929100	-1.28018600
H	3.60262300	2.52649000	-0.27190300
C	4.46405600	0.57146100	0.07311500
H	4.89921500	-0.28851300	-0.43757400
C	4.57641100	0.59956500	1.43272300
C	4.13525500	1.74566500	2.29974400
H	3.56054200	1.37746600	3.15722300
H	5.01599900	2.25422700	2.71282100
H	3.53587800	2.48634600	1.76992000
C	5.17948200	-0.56610800	2.16766900
H	4.46598700	-0.98647800	2.88981000
H	5.50959800	-1.35983700	1.49328700
H	6.04239300	-0.23312800	2.75717700
C	1.64287100	-0.09279400	-0.51127100
C	2.43071300	-0.53678000	0.37487200
H	2.70424200	-1.21779700	1.15666300
P	-1.90048700	-2.79380400	0.11819700
C	-2.08005700	-4.02235500	-1.23238700
H	-2.18156000	-3.50334600	-2.18832300
H	-1.19247700	-4.65802600	-1.26917400
H	-2.96430000	-4.64296600	-1.06060200
C	-1.88091800	-3.78002300	1.66595800

H	-0.99533300	-4.41957000	1.68108000
H	-1.84753000	-3.10919400	2.52734800
H	-2.77869300	-4.40236600	1.72506600
C	-3.48873500	-1.87708300	0.14817300
H	-3.49129500	-1.17332600	0.98366100
H	-3.60770200	-1.31941100	-0.78352300
H	-4.32272200	-2.57586500	0.25961700
Au	-0.03066800	-1.41670300	-0.13957200
C	-0.10299700	2.36896200	-0.56260900
C	-0.37799300	2.18670900	0.80109800
C	-1.11130700	2.79857700	-1.43738900
C	-1.66252000	2.40636700	1.28704300
H	0.42254300	1.89240700	1.46965500
C	-2.39816700	3.01940900	-0.95883300
H	-0.87584300	2.97398600	-2.48047500
C	-2.65801100	2.81239800	0.39686900
H	-1.89755600	2.27782000	2.33589100
H	-3.19231000	3.35384200	-1.61392100
N	-4.01261300	3.03979200	0.90240600
O	-4.87065900	3.42827200	0.10706800
O	-4.22806000	2.82685800	2.09787300

Table S12: Optimized geometry for IIIa

Free energy G = -1497.836875 Hartree/particle.

P	3.76020900	-1.96230900	0.21154800
C	5.23327900	-1.80982200	-0.87766300
H	5.94648500	-2.61167500	-0.66574700
H	4.92412800	-1.86807800	-1.92397900
H	5.71426800	-0.84342100	-0.70823700
C	4.44742300	-1.97656500	1.91589200
H	4.90472100	-1.00804000	2.13247300
H	3.64218600	-2.15188200	2.63334100
H	5.20084700	-2.76286700	2.01792800
C	3.15498200	-3.67550300	-0.06327300
H	2.32532400	-3.88344900	0.61680300
H	2.79812300	-3.77586900	-1.09127100
H	3.95697100	-4.39818300	0.11295100
Au	2.11745200	-0.29683700	-0.14938400
C	1.03670900	2.47044900	-0.14283000
C	-0.70576300	0.85808800	-0.99834000
C	0.11667700	3.70826800	-0.47528700
H	2.06710100	2.69713300	0.10121900
C	-1.15133700	2.02050400	-1.90745200
C	-1.20041800	3.36355400	-1.17770500
H	0.72073500	4.45438000	-0.99462800
H	-2.12703400	1.79347700	-2.34766300
H	-0.44250800	2.07338900	-2.74192300
H	-2.02368000	3.36244400	-0.45986500
H	-1.41462600	4.16067700	-1.89502500
C	0.68150100	1.17398600	-0.41157800
C	-0.70605800	-0.45482000	-1.78049600
H	-0.43899700	-1.29526800	-1.13472500
H	-1.68692400	-0.64595000	-2.22528100
H	0.02750600	-0.39793000	-2.58901900
O	-1.56820400	0.74961700	0.19188000
C	0.19008800	3.80509400	0.96768100
C	1.27960500	4.62598300	1.58694800
H	0.80330700	5.54340300	1.96017700
H	1.72681000	4.12616100	2.45020900

H	2.05507100	4.91538900	0.87644400
C	-0.87152000	3.31755700	1.89872100
H	-0.44582800	3.02290600	2.86059800
H	-1.53498100	4.17495800	2.08710300
H	-1.46197000	2.50191000	1.49007900
C	-2.76227500	0.06848300	0.14343100
C	-2.83352100	-1.18696400	0.76359200
C	-3.91214700	0.64928000	-0.41205300
C	-4.04203500	-1.87045400	0.81937700
H	-1.93659300	-1.61064000	1.20173500
C	-5.12402300	-0.03156600	-0.36516000
H	-3.86294000	1.63468100	-0.85948600
C	-5.17242600	-1.28386600	0.24793200
H	-4.11964500	-2.84069800	1.29288100
H	-6.02518800	0.39686300	-0.78467700
N	-6.44772600	-1.99872200	0.30214400
O	-7.43320700	-1.46670600	-0.21396000
O	-6.47269700	-3.09767200	0.86086600

Table S13: Optimized geometry for TS_{4a}

Free energy G = -1497.815575 Hartree/particle.

C	0.04359900	4.56657000	0.07417100
C	1.31364100	3.78262000	-0.17055700
C	-0.49870900	2.28707400	-0.74289400
C	-1.10161400	3.53020700	-0.02842900
H	0.05861700	5.08416600	1.03777400
H	-0.04187800	5.33893600	-0.69692300
H	-1.95255600	3.93893900	-0.57515100
H	-1.45568600	3.23632500	0.96187100
C	-0.69355100	2.28621400	-2.27621200
H	-0.14884800	1.45336200	-2.72803400
H	-1.75175800	2.18381600	-2.52290100
H	-0.33543800	3.22272700	-2.71185400
C	1.01946100	2.45469600	-0.60385100
C	2.04927700	1.49409400	-0.29881200
H	3.01046100	1.76563900	-0.73778400
P	1.85775500	-2.93797900	-0.06716100
C	0.60533200	-3.72866800	-1.15674900
H	0.66317000	-4.81799200	-1.07711200
H	-0.39521000	-3.39751600	-0.86817500
H	0.78441300	-3.43293200	-2.19333400
C	3.45128000	-3.70106400	-0.57411600
H	3.68998100	-3.40380200	-1.59820000
H	4.24920600	-3.35194600	0.08566000
H	3.38698100	-4.79167700	-0.51946300
C	1.52651900	-3.63981700	1.59988100
H	2.28549100	-3.28692000	2.30249400
H	0.54600900	-3.30807900	1.94957400
H	1.54655700	-4.73310500	1.56584600
Au	1.87257100	-0.58505000	-0.15803800
O	-0.88025600	1.03259000	-0.17334800
C	1.89784500	2.57388500	0.92956400
C	3.29293800	3.07625800	1.35101800
H	3.18760500	3.93179400	2.02426000
H	3.79155000	2.26434900	1.88849800
H	3.91474800	3.36513800	0.50377600
C	1.03668100	2.21778500	2.13329300
H	0.11777600	1.69777300	1.86969400
H	1.61771300	1.55498900	2.78159700
H	0.79265900	3.11463000	2.71171100

H	2.20790300	4.31148500	-0.47897500
C	-2.16118600	0.57360900	-0.09987800
C	-3.31338200	1.26590800	-0.50428500
C	-2.27557300	-0.71400700	0.45837200
C	-4.56129200	0.66671500	-0.36077900
H	-3.26111400	2.26104600	-0.92115700
C	-3.51683400	-1.30916900	0.60236100
H	-1.37217400	-1.22248500	0.77535000
C	-4.65501000	-0.61103400	0.18629800
H	-5.46006200	1.18558600	-0.66861200
H	-3.61833300	-2.29819500	1.03030700
N	-5.96550500	-1.23054000	0.33223500
O	-6.95596000	-0.59290700	-0.03656700
O	-6.02068800	-2.36404500	0.81812200

Table S14: Optimized geometry for Va

Free energy G = -1497.891967 Hartree/particle.

C	0.41774600	3.85225100	1.95908100
C	-0.54994100	2.49339700	0.15273000
C	-0.85789400	3.07889400	1.55531800
H	0.63865200	3.77769200	3.02947400
H	0.32917100	4.92497800	1.73774000
H	-1.74504900	3.71590500	1.56488200
H	-1.02404500	2.24964100	2.24908600
C	-1.08434500	3.34960300	-1.00381500
H	-0.74393800	2.96707200	-1.97015700
H	-2.17628400	3.37608100	-1.01527200
H	-0.72296200	4.37571300	-0.89414500
C	0.98549800	2.44132100	0.13765700
P	1.50147600	-2.66538300	0.68277900
C	0.13808000	-2.77400000	1.90433900
H	0.05953600	-3.79656200	2.28468200
H	0.33750700	-2.09233600	2.73416400
H	-0.80440900	-2.48930500	1.43162900
C	1.12577800	-3.92920500	-0.59059400
H	0.19715700	-3.66789400	-1.10240000
H	1.93559100	-3.96542500	-1.32284300
H	1.01557200	-4.91066700	-0.12062500
C	2.99282700	-3.27420400	1.55911400
H	3.84037400	-3.29215400	0.87050900
H	3.22610500	-2.60411200	2.38959800
H	2.81566700	-4.28274900	1.94389700
Au	1.79117200	-0.52568400	-0.18690700
O	-0.98376400	1.10753500	-0.01332900
C	1.48739800	3.22969900	1.10746200
H	2.53586400	3.45792400	1.25197000
C	3.01363200	1.37952500	-1.08050600
C	4.08363000	1.70975300	-0.06928700
H	4.45084600	2.72809800	-0.25619200
H	3.72941800	1.66709100	0.96177700
H	4.93602500	1.03430800	-0.17761100
C	3.52637200	0.93976900	-2.43340000
H	2.71868600	0.68996600	-3.12485000
H	4.10764200	1.76362000	-2.86847100
H	4.20352100	0.08492800	-2.34716900
C	1.66742600	1.69315900	-0.92998000
H	1.05287600	1.52765300	-1.81415000
C	-2.27688500	0.70391700	-0.07769300
C	-3.38542700	1.42891900	0.39443800
C	-2.46936800	-0.57716900	-0.63469200

C	-4.65929600	0.87850400	0.30852100
H	-3.26681200	2.40804100	0.83570900
C	-3.73684400	-1.12852500	-0.71445200
H	-1.60506800	-1.11697500	-1.00709400
C	-4.82765000	-0.39227400	-0.24075800
H	-5.52178600	1.42414900	0.66958800
H	-3.89610700	-2.10974100	-1.14300100
N	-6.16355800	-0.96285200	-0.32386500
O	-7.11302800	-0.28915800	0.08811100
O	-6.28421100	-2.09527700	-0.80235600

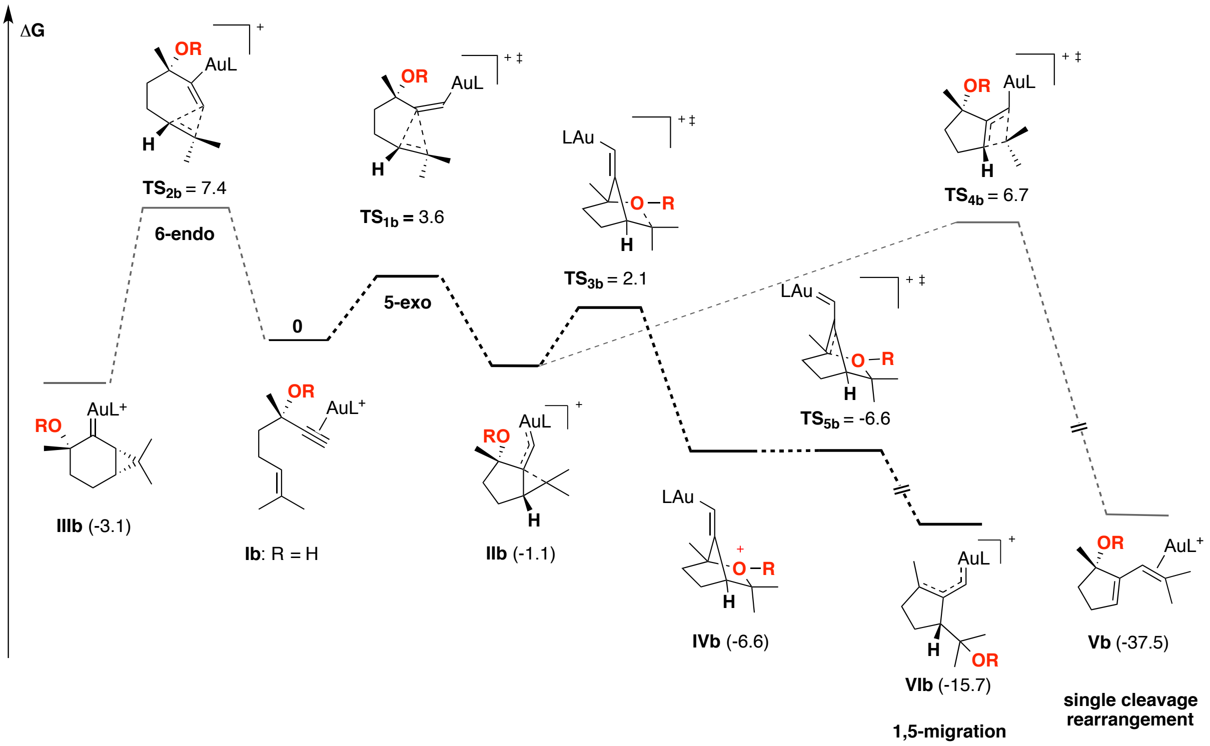

Scheme S2. Reaction pathways and energies for the the cyclization of **Ib** calculated at the B3LYP/6-31G(d,p) (C, H, P, O), SDD(Au) level taking into account solvent effect of CH_2Cl_2 (IEF-PCM) and employing PMe_3 as the phosphine. ΔG energies are given in kcal·mol-1.

Table S15: Optimized geometry for Ib

Free energy G = -1062.362318 Hartree/particle.

C	-2.46743700	-1.44743000	0.43923900
C	-2.93760200	-2.91325900	0.36541100
H	-3.55757000	-3.12630500	1.24020300
H	-3.52622900	-3.09315400	-0.53765700
H	-2.08277000	-3.59687000	0.36015500
O	-1.75056100	-1.20874000	1.64811800
H	-1.16378300	-1.96161900	1.81026300
C	-3.68691000	-0.49538000	0.43275400
H	-4.17956700	-0.57563700	-0.54279600
H	-4.38227300	-0.90574400	1.17299200
C	-3.42293900	0.98065500	0.78272000
H	-4.41267900	1.42888700	0.95525800
H	-2.89829300	1.03162300	1.73935200
C	-2.71679100	1.77889300	-0.28221100
H	-3.08819200	1.61434300	-1.29534100
C	-1.74777600	2.69718600	-0.12531900
C	-1.13728900	3.08772600	1.19868400
H	-1.49430900	2.48556200	2.03589500
H	-0.04338600	3.00245100	1.15951600

H	-1.35460200	4.13967100	1.42607900
C	-1.20195000	3.45428300	-1.31332200
H	-0.11965600	3.29749000	-1.41792700
H	-1.68297100	3.15235100	-2.24796000
H	-1.34526300	4.53592300	-1.19037500
C	-1.58819100	-1.20260300	-0.75257900
C	-1.02358900	-1.14282100	-1.84493600
H	-0.73366400	-1.13381700	-2.87673000
P	2.69374800	0.13592800	0.43357900
C	3.33352800	1.66487300	-0.34805100
H	3.44725000	1.50595400	-1.42262700
H	2.62693600	2.48149700	-0.18392700
H	4.30268400	1.92755600	0.08552700
C	2.62909900	0.48413700	2.23220700
H	1.89670800	1.27077400	2.42621900
H	2.33023400	-0.41833800	2.77009100
H	3.61317900	0.80722100	2.58378600
C	4.00925100	-1.12118100	0.21599000
H	3.72230000	-2.04301600	0.72683800
H	4.14331800	-1.33226800	-0.84736300
H	4.94957000	-0.75060500	0.63402000
Au	0.63775800	-0.51979000	-0.43361600

Table S16: Optimized geometry for TS_{1b}

Free energy G = -1062.356538 Hartree/particle.

C	2.23881400	1.56564300	0.12289200
C	1.03408000	2.51920700	-0.04658500
H	1.36509900	3.52525000	0.22399600
H	0.67120300	2.52426400	-1.07672600
H	0.21165200	2.22720200	0.61222000
O	2.72969700	1.61193200	1.46126300
H	2.02031000	1.33413400	2.05888600
C	3.40205700	2.00541000	-0.77906200
H	3.04196100	2.05236800	-1.81189000
H	3.71007500	3.01273700	-0.48396200
C	4.56404500	1.00534300	-0.65891700
H	5.30805100	1.21945400	-1.43457800
H	5.05622000	1.12618200	0.30694000
C	4.03897500	-0.38965000	-0.83888300
H	3.74764500	-0.65306800	-1.85513300
C	4.03047800	-1.39284300	0.08861600
C	4.50998800	-1.24986100	1.50711500
H	4.58750200	-0.21356800	1.83195300
H	3.83641600	-1.77790400	2.19048500
H	5.49301500	-1.72821100	1.60754900
C	3.57410000	-2.76917100	-0.29992800
H	2.73684700	-3.09571500	0.33061300
H	3.27978800	-2.83401900	-1.34986300
H	4.38218700	-3.49078500	-0.12601900
C	1.81084300	0.17649100	-0.22874100
C	0.92908600	-0.72922000	-0.40440700
H	1.09694900	-1.76739700	-0.67071400
P	-3.42372300	0.03589000	0.13418300
C	-3.84502800	1.38570500	1.30527500
H	-3.42532300	1.16123100	2.28858000
H	-3.42014700	2.32643500	0.94744400
H	-4.93080300	1.48822000	1.38985400
C	-4.28361700	0.47280300	-1.42803900
H	-3.86411300	1.39865600	-1.82847900
H	-4.13882500	-0.32438300	-2.16093100

H	-5.35363300	0.60801600	-1.24580200
C	-4.30481800	-1.44307200	0.76987800
H	-4.16181200	-2.27795400	0.08008500
H	-3.89846300	-1.71958100	1.74557400
H	-5.37367000	-1.23248000	0.86814000
Au	-1.12220100	-0.29447600	-0.13666300

Table S17: Optimized geometry for IIb

Free energy G = -1062.364012 Hartree/particle.

C	4.38564000	0.90734000	-0.79509400
C	3.57473900	-0.38766800	-0.87174900
C	2.15960500	1.42299200	0.16689800
C	3.65994200	1.82616800	0.20348400
H	5.42577800	0.71536300	-0.51551200
H	4.39619600	1.35115700	-1.79454400
H	3.79481600	2.88053200	-0.05110100
H	4.04336200	1.69319800	1.21633500
C	1.37040400	2.37904000	-0.74239300
H	0.33133900	2.05399900	-0.83979400
H	1.38745900	3.38636600	-0.31108100
H	1.81599500	2.43318000	-1.74063100
C	2.11072000	-0.00319200	-0.42001900
C	0.99199100	-0.74668200	-0.63966400
H	1.17101200	-1.72949200	-1.07414400
P	-3.25844700	0.10804200	0.21023300
C	-3.74565700	1.86982500	0.01419200
H	-4.81026800	2.00102500	0.22859500
H	-3.16006500	2.48828400	0.69890800
H	-3.54157000	2.19271900	-1.00960900
C	-4.41798600	-0.81617000	-0.87596800
H	-4.23111400	-0.55198700	-1.91968300
H	-4.25563400	-1.88968400	-0.75308300
H	-5.45420400	-0.57538300	-0.62145100
C	-3.77810700	-0.33002600	1.91771300
H	-3.60355500	-1.39486700	2.08987200
H	-3.18699600	0.24166500	2.63703400
H	-4.83945800	-0.10906900	2.06329000
Au	-0.97206000	-0.31042700	-0.23740500
O	1.57599800	1.39116800	1.47660200
H	1.56086100	2.29826700	1.81487800
C	3.46232400	-1.28005400	0.26664200
C	3.21950500	-2.73707300	0.01538700
H	4.19998200	-3.21427300	0.16446600
H	2.53614100	-3.18153500	0.74247400
H	2.89912300	-2.96388100	-1.00132600
C	3.85261300	-0.92637300	1.67606100
H	3.23855500	-0.12656200	2.09282800
H	3.76022500	-1.80089300	2.32181900
H	4.89717800	-0.59113000	1.68743000
H	3.61186400	-0.90201200	-1.83023600

Table S18: Optimized geometry for TS_{3b}

Free energy G = -1.062.358949 Hartree/particle.

C	4.03443700	-1.15192200	0.18880000
C	3.42636300	-0.44730000	-0.93448100
H	3.24269500	-1.16336000	-1.73939700
C	5.18869400	-0.63213300	0.95259600
H	5.22717400	-1.02750100	1.96911500

H	6.07270900	-1.03040400	0.42228400
H	5.26376000	0.45149300	0.95593800
C	3.58142600	-2.52438100	0.51467600
H	4.42575500	-3.20141000	0.31556900
H	3.37843400	-2.61843900	1.58696200
H	2.72118000	-2.85190300	-0.06613000
O	2.87884600	1.04220500	1.54516000
C	2.25587200	1.34636600	0.27629200
C	4.06012300	0.87801700	-1.39061700
H	5.14595500	0.88609100	-1.27234500
H	3.85983500	0.99261100	-2.45859400
C	3.35730700	2.00487300	-0.58474700
H	2.90680800	2.73277500	-1.26366700
H	4.04300000	2.55020600	0.06928700
H	2.20555900	0.62579700	2.10459900
C	1.97872300	0.00245300	-0.39770900
C	0.86246700	-0.70602200	-0.58349400
H	1.00470700	-1.68703300	-1.04406000
Au	-1.11918700	-0.28246900	-0.19604700
C	-3.85973900	1.70766800	0.88212400
H	-4.94105500	1.79218500	1.02458100
H	-3.35771400	1.83617800	1.84419400
H	-3.52326500	2.49580400	0.20407000
C	-4.19116700	-1.14287200	1.33621000
H	-5.26011900	-0.94151400	1.45226000
H	-4.05426400	-2.15530800	0.94860900
H	-3.70279200	-1.07284800	2.31119800
C	-4.44429800	-0.06090000	-1.34855900
H	-4.32164100	-1.05448700	-1.78656300
H	-5.50132800	0.10717600	-1.12288800
H	-4.10613300	0.68378000	-2.07313000
P	-3.42737200	0.06454000	0.17856200
C	1.05081800	2.25720900	0.49191000
H	0.56098400	2.49295200	-0.45610200
H	0.31095100	1.79060000	1.14940100
H	1.38819600	3.18957200	0.95477200

Table S19: Optimized geometry for IVb

Free energy G = -1062.372835 Hartree/particle.

C	4.07573500	-0.68568300	0.39439800
C	3.35159500	-0.43051900	-0.95282200
H	3.46402900	-1.28767500	-1.61853300
C	5.55987700	-0.37815300	0.47337500
H	5.94275800	-0.58556000	1.47569600
H	6.08962600	-1.02385100	-0.23386300
H	5.78155600	0.65982300	0.22510200
C	3.72912300	-2.02932900	1.01568200
H	4.26562900	-2.81048500	0.46902400
H	4.05694800	-2.07927300	2.05918400
H	2.65975300	-2.24283600	0.95519200
O	3.37833200	0.43335600	1.26087600
C	2.23134100	1.09355200	0.31460900
C	3.76301300	0.93019600	-1.56819500
H	4.84118700	1.06724400	-1.66654000
H	3.33104100	1.00893300	-2.56844700
C	3.10360500	1.95991700	-0.59961800
H	2.45635700	2.66689400	-1.12565300
H	3.82420500	2.54066400	-0.01765700
H	2.95469200	0.02809200	2.04082100
C	1.92177700	-0.12523800	-0.50847400

C	0.76052900	-0.72631000	-0.80494300
H	0.88070300	-1.58913900	-1.46783800
Au	-1.17893800	-0.28688800	-0.27973400
C	-4.26716500	1.37067400	-0.82922900
H	-5.31478500	1.50322700	-0.54340500
H	-3.74471900	2.32639400	-0.73990600
H	-4.21578800	1.04196000	-1.87016500
C	-3.75151600	0.73595600	1.95754800
H	-4.82158300	0.88193300	2.13151000
H	-3.36490800	0.01105000	2.67814200
H	-3.23022400	1.68522300	2.10344400
C	-4.52307100	-1.36680100	0.11305700
H	-4.15908300	-2.14005400	0.79422700
H	-5.55923500	-1.12207600	0.36475600
H	-4.48120900	-1.75458900	-0.90770900
P	-3.45196900	0.12277300	0.24904400
C	1.25364100	1.77096200	1.22992400
H	0.48217400	2.23940700	0.61258700
H	0.75443900	1.06348400	1.89758500
H	1.74453000	2.55154300	1.81687800

Table S20: Optimized geometry for TS_{5b}

Free energy G = -1062.372866 Hartree/particle.

C	4.03956500	-0.74613200	0.38588300
C	3.35932200	-0.31776000	-0.95313500
H	3.45761100	-1.10387800	-1.70385600
C	5.55968600	-0.62184800	0.41830500
H	5.95282700	-0.99727300	1.36701300
H	5.99408900	-1.21753400	-0.39045600
H	5.88704500	0.41235500	0.30155100
C	3.58978600	-2.13021200	0.84598500
H	2.50348800	-2.23998200	0.80593400
H	4.03594000	-2.89248300	0.20109900
H	3.92991400	-2.32387100	1.86910900
O	3.50291000	0.28628700	1.34206300
C	2.14566000	1.19395900	0.27969900
C	3.84882900	1.07177200	-1.43094700
H	4.93393200	1.17998100	-1.43936400
H	3.49544200	1.24023900	-2.45115900
C	3.15463300	2.05496000	-0.44542400
H	2.60046700	2.84745600	-0.96330200
H	3.83501100	2.54745200	0.25336600
H	3.06642700	-0.15965500	2.08722900
C	1.92637500	-0.00427400	-0.53255500
C	0.76888000	-0.63762900	-0.82519900
H	0.90905800	-1.48586800	-1.50287500
Au	-1.17782300	-0.25338300	-0.29753000
C	-4.21444300	1.58546200	-0.48789400
H	-5.26625000	1.66892700	-0.19913700
H	-3.67629000	2.47297200	-0.14631000
H	-4.14216400	1.53168000	-1.57691600
C	-3.78762100	0.24517400	2.05526800
H	-4.85780500	0.37752900	2.23960000
H	-3.44111700	-0.65309600	2.57215500
H	-3.24409100	1.10700500	2.45052600
C	-4.55435600	-1.29362800	-0.28520500
H	-4.22445300	-2.22600500	0.17936600
H	-5.59092600	-1.09405400	0.00135800
H	-4.49468600	-1.40468100	-1.37055200
P	-3.45856300	0.08130900	0.25314500

C	1.21878800	1.77867900	1.28305300
H	0.49551700	2.39647200	0.73618100
H	0.66010600	1.01753900	1.82966000
H	1.75345000	2.43496000	1.97392100

Table S21: Optimized geometry for VIb

Free energy G = -1062.387406 Hartree/particle.

C	4.10560000	1.31392900	-0.90592400
C	3.38579300	-0.05387400	-0.77172300
C	1.97041800	1.66715100	0.14331500
C	3.33588200	2.25155700	0.04157300
H	5.16803600	1.27458400	-0.66615800
H	4.01096000	1.67514600	-1.93469900
H	3.31478200	3.30668800	-0.25207000
H	3.76079200	2.21581400	1.05699200
C	0.86065400	2.44829300	0.72723800
H	-0.04571600	1.86413600	0.88850300
H	1.19350700	2.89991000	1.66962400
H	0.63084100	3.28933300	0.05846100
C	1.95674100	0.36371900	-0.38974100
C	0.81495200	-0.39019600	-0.62945400
H	1.04414000	-1.30623600	-1.18330200
P	-3.50692900	-0.08714100	0.18805500
C	-4.22045200	1.60532600	0.15228200
H	-5.29513300	1.56850700	0.35270400
H	-3.73277900	2.22459300	0.90891900
H	-4.05119300	2.05266800	-0.83003100
C	-4.50070800	-1.03909800	-1.02887500
H	-4.32897700	-0.63894600	-2.03100300
H	-4.19268200	-2.08728000	-1.01279400
H	-5.56579200	-0.97036600	-0.78974200
C	-3.99157900	-0.76362800	1.82619100
H	-3.68421500	-1.80972800	1.89551600
H	-3.49305600	-0.19821700	2.61709300
H	-5.07500600	-0.69442100	1.95963000
Au	-1.17121400	-0.17797300	-0.22557700
O	4.10363400	-0.23391400	1.49695800
H	4.51815600	-0.78358200	2.17644900
C	4.03605800	-1.00327500	0.28275800
C	3.19136100	-2.26391100	0.52472300
H	3.05819100	-2.84458900	-0.39375700
H	3.69444300	-2.91000400	1.25259400
H	2.20682000	-2.01519000	0.93111100
C	5.44860900	-1.41704900	-0.16068100
H	6.10522800	-0.55260300	-0.28034300
H	5.89629000	-2.07605800	0.59114300
H	5.42055000	-1.96517900	-1.10768200
H	3.38260900	-0.59435900	-1.72318100

Table S22: Optimized geometry for TS_{2b}

Free energy G = -1062.350514 Hartree/particle.

C	1.65532300	1.87621900	0.04237300
C	0.73069200	2.93127300	-0.57382900
H	1.13180300	3.92943400	-0.37606700
H	0.64152600	2.79531000	-1.65563900
H	-0.27325600	2.86371100	-0.14013000
O	1.75037400	2.05901700	1.45687700
H	0.85263400	2.12597900	1.81309500

C	3.08101200	2.02269200	-0.51281000
H	3.03511100	2.00265500	-1.60740200
H	3.42482200	3.02020600	-0.22163100
C	4.09227300	0.98051600	-0.00588100
H	5.09637000	1.33144000	-0.27909000
H	4.06043300	0.94908900	1.08540200
C	3.91759200	-0.38992100	-0.60873000
H	3.88656600	-0.41121600	-1.69876600
C	4.04405300	-1.59277100	0.02438100
C	4.21396100	-1.75922900	1.50812200
H	4.08118500	-0.83350800	2.06777900
H	3.50891200	-2.50538900	1.89257100
H	5.21947600	-2.14465900	1.72066900
C	4.01628100	-2.87191200	-0.76618800
H	3.19700900	-3.52271300	-0.43013000
H	3.91054000	-2.69706300	-1.83954200
H	4.93957000	-3.43926300	-0.59706900
C	1.13086900	0.46550400	-0.22033100
C	1.62284600	-0.68484800	-0.42689400
H	1.53552300	-1.73092600	-0.64506700
P	-3.25978900	-0.41697500	0.09499300
C	-4.11596400	0.84225700	1.11929000
H	-3.69873700	0.83844800	2.12903400
H	-3.96823900	1.83186500	0.68048500
H	-5.18691000	0.62528200	1.16938500
C	-4.12908500	-0.39711200	-1.52122300
H	-3.98339700	0.57334200	-2.00118800
H	-3.71645400	-1.17488200	-2.16796000
H	-5.19886800	-0.57428700	-1.37797800
C	-3.68412000	-2.03251000	0.85508000
H	-3.26103100	-2.84079700	0.25417000
H	-3.26242300	-2.08423600	1.86151600
H	-4.77008700	-2.15120700	0.90956400
Au	-0.95902100	-0.04221600	-0.08995000

Table S23: Optimized geometry for IIIb

Free energy G = -1062.36726 Hartree/particle.

P	3.21481700	0.29810200	0.06298100
C	4.02434100	0.69923800	-1.53805700
H	5.09912500	0.84459600	-1.39553900
H	3.86099700	-0.11669500	-2.24611400
H	3.58734900	1.61244100	-1.94915900
C	3.69514000	1.67596000	1.18067600
H	3.24395100	2.60623300	0.82724300
H	3.32960900	1.47145700	2.18988700
H	4.78297800	1.78835700	1.20526800
C	4.13271100	-1.16207400	0.69708200
H	3.77011700	-1.41438000	1.69657800
H	3.95937800	-2.01525100	0.03670200
H	5.20522500	-0.95273000	0.74359700
Au	0.87552700	-0.03709500	-0.09470500
C	-1.93351000	0.77369200	-0.63730800
C	-1.84338900	-1.62766000	0.15574900
C	-3.47976300	0.65303300	-0.93212900
H	-1.42594800	1.62906600	-1.06564700
C	-3.03811500	-1.85346200	-0.80074300
C	-4.07929600	-0.73725100	-0.71316200
H	-3.68711600	1.09517400	-1.90736200
H	-3.50569700	-2.81663800	-0.56471900
H	-2.65334900	-1.93327600	-1.82415200

H	-4.58037700	-0.78469200	0.25595900
H	-4.84953900	-0.89280600	-1.47415000
C	-1.17535600	-0.28269300	-0.18142400
C	-0.86019100	-2.79721500	0.04293000
H	-0.02845700	-2.66678500	0.74060600
H	-1.37113300	-3.73896300	0.27556300
H	-0.45564900	-2.87705900	-0.97043500
O	-2.28556500	-1.50732500	1.52041900
H	-2.61797000	-2.37285900	1.80119500
C	-3.38739700	1.61313000	0.14775300
C	-3.30606600	3.07705200	-0.18653400
H	-4.29104300	3.50941500	0.03363700
H	-2.57846900	3.59631800	0.44284600
H	-3.08316800	3.26097400	-1.23903200
C	-3.66773000	1.29574700	1.58541200
H	-3.10998500	1.96369600	2.24636100
H	-4.73682300	1.49578500	1.74638000
H	-3.45667400	0.26125500	1.84667000

Table S24: Optimized geometry for TS_{4b}

Free energy G = -1062.351564 Hartree/particle.

C	4.54487900	0.24952200	0.23682900
C	3.53339600	-0.56260100	-0.54756100
C	2.40420500	1.48981700	0.07798000
C	3.73434600	1.40807400	0.86646000
H	5.06916700	-0.35793200	0.98070100
H	5.30494600	0.61685700	-0.46095200
H	4.27332500	2.35701700	0.83029200
H	3.50106800	1.20414200	1.91328200
C	2.47144300	2.50165800	-1.09010900
H	1.55143900	2.48077400	-1.68343000
H	2.58935700	3.49984500	-0.65966700
H	3.31789600	2.30601100	-1.75472300
C	2.28921500	0.13415600	-0.63133600
C	1.13686400	-0.71211900	-0.79506600
H	1.17822400	-1.33608100	-1.68847500
P	-3.09650900	0.21254800	0.21845500
C	-3.63010400	1.92745200	-0.16965200
H	-4.68689700	2.06494300	0.07681800
H	-3.03005500	2.63644700	0.40567800
H	-3.47911700	2.12404300	-1.23395000
C	-4.27991300	-0.85697900	-0.69448400
H	-4.14779600	-0.71273400	-1.76951700
H	-4.08513900	-1.90536900	-0.45595500
H	-5.30913000	-0.60865800	-0.41986500
C	-3.52821200	-0.02279800	1.98938500
H	-3.31311300	-1.05322600	2.28275000
H	-2.92554200	0.64884700	2.60571000
H	-4.58869100	0.18657600	2.15675300
Au	-0.83410600	-0.23719300	-0.28416600
O	1.35706700	1.81783700	0.96689300
H	0.51063300	1.75474500	0.49081200
C	2.23614800	-1.41824300	0.21125900
C	2.43523200	-2.88618700	-0.21199600
H	3.29187900	-3.30749500	0.32206100
H	1.53666200	-3.44333900	0.06892900
H	2.59408800	-3.00227200	-1.28413000
C	2.05776500	-1.27261000	1.71592400
H	1.71927700	-0.28008200	2.00861000
H	1.30456400	-1.99607300	2.04279900

H	2.99096300	-1.50843400	2.23768600
H	3.88588500	-1.23609300	-1.32083400

Table S25: Optimized geometry for Vb

Free energy G = -1062.42211 Hartree/particle.

C	-4.13359600	-0.76185200	1.46078300
C	-2.65118400	-1.28101200	-0.43929000
C	-3.46611500	-1.93458500	0.70846200
H	-4.19366400	-0.92273200	2.54309300
H	-5.16403800	-0.58997500	1.11866200
H	-4.18157600	-2.67817700	0.34631200
H	-2.75553100	-2.44128200	1.36870400
C	-3.40943500	-1.28867000	-1.77520700
H	-2.86401200	-0.73969700	-2.54877200
H	-3.54721200	-2.31880200	-2.12437000
H	-4.40046300	-0.83832700	-1.66456200
C	-2.42531800	0.14144000	0.08179100
P	2.71132600	-0.77388300	0.22617000
C	2.77614600	-2.46694000	-0.47673600
H	3.74906700	-2.91947100	-0.26421800
H	1.98651300	-3.07973000	-0.03648500
H	2.62656500	-2.41963800	-1.55783100
C	4.13199300	0.11079400	-0.52372500
H	3.97564200	0.20310600	-1.60099100
H	4.21261200	1.11057800	-0.09119800
H	5.05698700	-0.44217500	-0.33675300
C	3.13250300	-0.96662800	2.00045100
H	3.18033500	0.01671700	2.47340800
H	2.36078000	-1.55959700	2.49676100
H	4.09955400	-1.46763200	2.10276400
Au	0.67327700	0.29539400	-0.12451300
O	-1.36401900	-1.89948900	-0.61421100
H	-1.49000400	-2.73509200	-1.08532200
C	-3.26351400	0.41278900	1.09986600
H	-3.36935500	1.37788300	1.58223500
C	-0.86255200	2.18536900	-0.13482400
C	-0.97025100	2.66956000	1.28991900
H	-1.80619700	3.37771000	1.36694300
H	-1.15043300	1.85964500	1.99850000
H	-0.06578600	3.21002500	1.58223800
C	-0.26666000	3.17687500	-1.10839600
H	-0.16269300	2.76178400	-2.11332500
H	-0.93145900	4.04917300	-1.16560000
H	0.70653300	3.54152000	-0.76737600
C	-1.48869600	1.03977300	-0.61201800
H	-1.43867300	0.89071900	-1.69159200

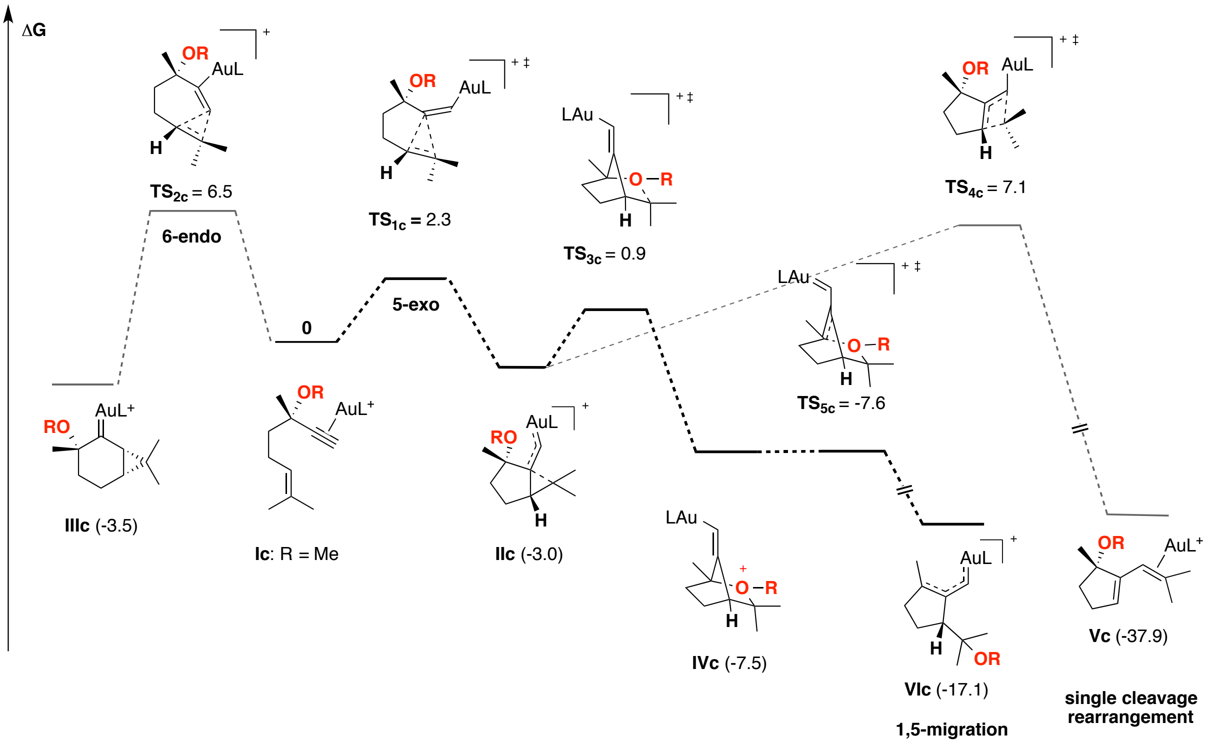

Scheme S3. Reaction pathways and energies for the the cyclization of **Ic** calculated at the B3LYP/6-31G(d,p) (C, H, P, O), SDD(Au) level taking into account solvent effect of CH_2Cl_2 (IEF-PCM) and employing PMe_3 as the phosphine. ΔG energies are given in kcal·mol-1.

Table S26: Optimized geometry for Ic

Free energy G = -1062.016988 Hartree/particle.

C	-2.46743700	-1.44743000	0.43923900
C	-2.93760200	-2.91325900	0.36541100
H	-3.55757000	-3.12630500	1.24020300
H	-3.52622900	-3.09315400	-0.53765700
H	-2.08277000	-3.59687000	0.36015500
O	-1.75056100	-1.20874000	1.64811800
H	-1.16378300	-1.96161900	1.81026300
C	-3.68691000	-0.49538000	0.43275400
H	-4.17956700	-0.57563700	-0.54279600
H	-4.38227300	-0.90574400	1.17299200
C	-3.42293900	0.98065500	0.78272000
H	-4.41267900	1.42888700	0.95525800
H	-2.89829300	1.03162300	1.73935200
C	-2.71679100	1.77889300	-0.28221100
H	-3.08819200	1.61434300	-1.29534100
C	-1.74777600	2.69718600	-0.12531900
C	-1.13728900	3.08772600	1.19868400
H	-1.49430900	2.48556200	2.03589500
H	-0.04338600	3.00245100	1.15951600
H	-1.35460200	4.13967100	1.42607900
C	-1.20195000	3.45428300	-1.31332200
H	-0.11965600	3.29749000	-1.41792700
H	-1.68297100	3.15235100	-2.24796000
H	-1.34526300	4.53592300	-1.19037500
C	-1.58819100	-1.20260300	-0.75257900
C	-1.02358900	-1.14282100	-1.84493600
H	-0.73366400	-1.13381700	-2.87673000
P	2.69374800	0.13592800	0.43357900
C	3.33352800	1.66487300	-0.34805100
H	3.44725000	1.50595400	-1.42262700
H	2.62693600	2.48149700	-0.18392700

H	4.30268400	1.92755600	0.08552700
C	2.62909900	0.48413700	2.23220700
H	1.89670800	1.27077400	2.42621900
H	2.33023400	-0.41833800	2.77009100
H	3.61317900	0.80722100	2.58378600
C	4.00925100	-1.12118100	0.21599000
H	3.72230000	-2.04301600	0.72683800
H	4.14331800	-1.33226800	-0.84736300
H	4.94957000	-0.75060500	0.63402000
Au	0.63775800	-0.51979000	-0.43361600

Table S27: Optimized geometry for TS_{1c}

Free energy G = -1062.005315 Hartree/particle.

C	2.23881400	1.56564300	0.12289200
C	1.03408000	2.51920700	-0.04658500
H	1.36509900	3.52525000	0.22399600
H	0.67120300	2.52426400	-1.07672600
H	0.21165200	2.22720200	0.61222000
O	2.72969700	1.61193200	1.46126300
H	2.02031000	1.33413400	2.05888600
C	3.40205700	2.00541000	-0.77906200
H	3.04196100	2.05236800	-1.81189000
H	3.71007500	3.01273700	-0.48396200
C	4.56404500	1.00534300	-0.65891700
H	5.30805100	1.21945400	-1.43457800
H	5.05622000	1.12618200	0.30694000
C	4.03897500	-0.38965000	-0.83888300
H	3.74764500	-0.65306800	-1.85513300
C	4.03047800	-1.39284300	0.08861600
C	4.50998800	-1.24986100	1.50711500
H	4.58750200	-0.21356800	1.83195300
H	3.83641600	-1.77790400	2.19048500
H	5.49301500	-1.72821100	1.60754900
C	3.57410000	-2.76917100	-0.29992800
H	2.73684700	-3.09571500	0.33061300
H	3.27978800	-2.83401900	-1.34986300
H	4.38218700	-3.49078500	-0.12601900
C	1.81084300	0.17649100	-0.22874100
C	0.92908600	-0.72922000	-0.40440700
H	1.09694900	-1.76739700	-0.67071400
P	-3.42372300	0.03589000	0.13418300
C	-3.84502800	1.38570500	1.30527500
H	-3.42532300	1.16123100	2.28858000
H	-3.42014700	2.32643500	0.94744400
H	-4.93080300	1.48822000	1.38985400
C	-4.28361700	0.47280300	-1.42803900
H	-3.86411300	1.39865600	-1.82847900
H	-4.13882500	-0.32438300	-2.16093100
H	-5.35363300	0.60801600	-1.24580200
C	-4.30481800	-1.44307200	0.76987800
H	-4.16181200	-2.27795400	0.08008500
H	-3.89846300	-1.71958100	1.74557400
H	-5.37367000	-1.23248000	0.86814000
Au	-1.12220100	-0.29447600	-0.13666300

Table S28: Optimized geometry for IIc

Free energy G = -1062.016335 Hartree/particle.

C	4.38564000	0.90734000	-0.79509400

C	3.57473900	-0.38766800	-0.87174900
C	2.15960500	1.42299200	0.16689800
C	3.65994200	1.82616800	0.20348400
H	5.42577800	0.71536300	-0.51551200
H	4.39619600	1.35115700	-1.79454400
H	3.79481600	2.88053200	-0.05110100
H	4.04336200	1.69319800	1.21633500
C	1.37040400	2.37904000	-0.74239300
H	0.33133900	2.05399900	-0.83979400
H	1.38745900	3.38636600	-0.31108100
H	1.81599500	2.43318000	-1.74063100
C	2.11072000	-0.00319200	-0.42001900
C	0.99199100	-0.74668200	-0.63966400
H	1.17101200	-1.72949200	-1.07414400
P	-3.25844700	0.10804200	0.21023300
C	-3.74565700	1.86982500	0.01419200
H	-4.81026800	2.00102500	0.22859500
H	-3.16006500	2.48828400	0.69890800
H	-3.54157000	2.19271900	-1.00960900
C	-4.41798600	-0.81617000	-0.87596800
H	-4.23111400	-0.55198700	-1.91968300
H	-4.25563400	-1.88968400	-0.75308300
H	-5.45420400	-0.57538300	-0.62145100
C	-3.77810700	-0.33002600	1.91771300
H	-3.60355500	-1.39486700	2.08987200
H	-3.18699600	0.24166500	2.63703400
H	-4.83945800	-0.10906900	2.06329000
Au	-0.97206000	-0.31042700	-0.23740500
O	1.57599800	1.39116800	1.47660200
H	1.56086100	2.29826700	1.81487800
C	3.46232400	-1.28005400	0.26664200
C	3.21950500	-2.73707300	0.01538700
H	4.19998200	-3.21427300	0.16446600
H	2.53614100	-3.18153500	0.74247400
H	2.89912300	-2.96388100	-1.00132600
C	3.85261300	-0.92637300	1.67606100
H	3.23855500	-0.12656200	2.09282800
H	3.76022500	-1.80089300	2.32181900
H	4.89717800	-0.59113000	1.68743000
H	3.61186400	-0.90201200	-1.83023600

Table S29: Optimized geometry for TS_{3c}

Free energy G = -1062.007814 Hartree/particle.

C	4.03443700	-1.15192200	0.18880000
C	3.42636300	-0.44730000	-0.93448100
H	3.24269500	-1.16336000	-1.73939700
C	5.18869400	-0.63213300	0.95259600
H	5.22717400	-1.02750100	1.96911500
H	6.07270900	-1.03040400	0.42228400
H	5.26376000	0.45149300	0.95593800
C	3.58142600	-2.52438100	0.51467600
H	4.42575500	-3.20141000	0.31556900
H	3.37843400	-2.61843900	1.58696200
H	2.72118000	-2.85190300	-0.06613000
O	2.87884600	1.04220500	1.54516000
C	2.25587200	1.34636600	0.27629200
C	4.06012300	0.87801700	-1.39061700
H	5.14595500	0.88609100	-1.27234500
H	3.85983500	0.99261100	-2.45859400
C	3.35730700	2.00487300	-0.58474700

H	2.90680800	2.73277500	-1.26366700
H	4.04300000	2.55020600	0.06928700
H	2.20555900	0.62579700	2.10459900
C	1.97872300	0.00245300	-0.39770900
C	0.86246700	-0.70602200	-0.58349400
H	1.00470700	-1.68703300	-1.04406000
Au	-1.11918700	-0.28246900	-0.19604700
C	-3.85973900	1.70766800	0.88212400
H	-4.94105500	1.79218500	1.02458100
H	-3.35771400	1.83617800	1.84419400
H	-3.52326500	2.49580400	0.20407000
C	-4.19116700	-1.14287200	1.33621000
H	-5.26011900	-0.94151400	1.45226000
H	-4.05426400	-2.15530800	0.94860900
H	-3.70279200	-1.07284800	2.31119800
C	-4.44429800	-0.06090000	-1.34855900
H	-4.32164100	-1.05448700	-1.78656300
H	-5.50132800	0.10717600	-1.12288800
H	-4.10613300	0.68378000	-2.07313000
P	-3.42737200	0.06454000	0.17856200
C	1.05081800	2.25720900	0.49191000
H	0.56098400	2.49295200	-0.45610200
H	0.31095100	1.79060000	1.14940100
H	1.38819600	3.18957200	0.95477200

Table S30: Optimized geometry for IVc

Free energy G = -1062.019293 Hartree/particle.

C	4.07572000	-0.69810800	0.39455100
C	3.34742100	-0.45006600	-0.94885000
H	3.44861400	-1.31175900	-1.61043400
C	5.56340900	-0.40080400	0.46535000
H	5.94393400	-0.56172600	1.47790700
H	6.08597300	-1.09478000	-0.19965400
H	5.81273000	0.61309400	0.14647900
C	3.72354700	-2.02476300	1.04437300
H	4.23525700	-2.82573400	0.50201800
H	4.06067900	-2.04736800	2.08379100
H	2.64916000	-2.21127100	1.01105900
O	3.33948300	0.39237300	1.26461300
C	2.24014600	1.09187600	0.31696500
C	3.75319100	0.90572600	-1.58148800
H	4.83016600	1.04856500	-1.68613900
H	3.31970600	0.96836600	-2.58200700
C	3.08766100	1.95047200	-0.63124600
H	2.42280900	2.63268000	-1.16810300
H	3.80564900	2.57418400	-0.08649800
H	3.97163300	1.04541200	1.61891800
C	1.92310600	-0.13548700	-0.49503300
C	0.75775700	-0.72572000	-0.79323000
H	0.87430700	-1.58869900	-1.45664600
Au	-1.18163700	-0.28320300	-0.27217500
C	-4.27535900	1.35612900	-0.84426200
H	-5.32411600	1.48736900	-0.56201800
H	-3.75650300	2.31445500	-0.76240500
H	-4.22047900	1.01839200	-1.88213300
C	-3.76718200	0.74399200	1.94934200
H	-4.83885200	0.88269500	2.11937900
H	-3.37646000	0.02759100	2.67618800
H	-3.25381600	1.69842900	2.08954600
C	-4.52059000	-1.37535800	0.11620500

H	-4.15591100	-2.14158800	0.80485300
H	-5.55925000	-1.13412900	0.36091100
H	-4.47165700	-1.77048700	-0.90144300
P	-3.45760100	0.12060700	0.24639300
C	1.25736600	1.76086800	1.23290700
H	0.45593500	2.18499600	0.62137900
H	0.81448200	1.04722000	1.92999900
H	1.72634200	2.57651600	1.79054700

Table S31: Optimized geometry for TS_{5c}

Free energy G = -1062.018638 Hartree/particle.

C	4.02798500	-0.75255500	0.39969000
C	3.35877700	-0.33039300	-0.94693100
H	3.45419500	-1.12268300	-1.69159400
C	5.54765900	-0.62029200	0.45082400
H	5.93065400	-0.96634000	1.41528900
H	5.99383000	-1.24259600	-0.33015200
H	5.88729900	0.40655500	0.29243700
C	3.58619700	-2.14005200	0.85553000
H	2.49941900	-2.24042600	0.82906500
H	4.02379000	-2.89852200	0.19948100
H	3.92912600	-2.32871900	1.87647600
O	3.41983800	0.22189400	1.37091400
C	2.13746100	1.19884400	0.26330300
C	3.85309000	1.05308800	-1.43690100
H	4.93808300	1.16468900	-1.43535800
H	3.51290400	1.20802400	-2.46351600
C	3.14421800	2.05134100	-0.47639300
H	2.57882900	2.82064700	-1.01775000
H	3.81477500	2.59167300	0.19802200
H	4.10572900	0.81313200	1.72325900
C	1.92478900	-0.00860000	-0.53552900
C	0.76774900	-0.64443300	-0.82475900
H	0.90943000	-1.49942300	-1.49352800
Au	-1.17809200	-0.25675200	-0.29797600
C	-4.22206500	1.56439600	-0.51697500
H	-5.27284300	1.65138700	-0.22555200
H	-3.68548200	2.46243300	-0.20142900
H	-4.15481500	1.48179900	-1.60441300
C	-3.77204500	0.29769300	2.05863000
H	-4.84122500	0.43102300	2.24809800
H	-3.41668800	-0.58298200	2.59915800
H	-3.22940900	1.17355200	2.42307600
C	-4.55196400	-1.30833400	-0.2335710
H	-4.21571700	-2.22731000	0.25259400
H	-5.58689900	-1.10416400	0.05573700
H	-4.50035500	-1.44745400	-1.31613900
P	-3.45620900	0.08341800	0.25952000
C	1.20269900	1.78311600	1.25793400
H	0.44528300	2.34802300	0.69952500
H	0.69228700	1.01500500	1.83928800
H	1.71419800	2.48679100	1.91884300

Table S32: Optimized geometry for VIc

Free energy G = -1062.03665 Hartree/particle.

C	4.10560000	1.31392900	-0.90592400
C	3.38579300	-0.05387400	-0.77172300
C	1.97041800	1.66715100	0.14331500

C	3.33588200	2.25155700	0.04157300
H	5.16803600	1.27458400	-0.66615800
H	4.01096000	1.67514600	-1.93469900
H	3.31478200	3.30668800	-0.25207000
H	3.76079200	2.21581400	1.05699200
C	0.86065400	2.44829300	0.72723800
H	-0.04571600	1.86413600	0.88850300
H	1.19350700	2.89991000	1.66962400
H	0.63084100	3.28933300	0.05846100
C	1.95674100	0.36371900	-0.38974100
C	0.81495200	-0.39019600	-0.62945400
H	1.04414000	-1.30623600	-1.18330200
P	-3.50692900	-0.08714100	0.18805500
C	-4.22045200	1.60532600	0.15228200
H	-5.29513300	1.56850700	0.35270400
H	-3.73277900	2.22459300	0.90891900
H	-4.05119300	2.05266800	-0.83003100
C	-4.50070800	-1.03909800	-1.02887500
H	-4.32897700	-0.63894600	-2.03100300
H	-4.19268200	-2.08728000	-1.01279400
H	-5.56579200	-0.97036600	-0.78974200
C	-3.99157900	-0.76362800	1.82619100
H	-3.68421500	-1.80972800	1.89551600
H	-3.49305600	-0.19821700	2.61709300
H	-5.07500600	-0.69442100	1.95963000
Au	-1.17121400	-0.17797300	-0.22557700
O	4.10363400	-0.23391400	1.49695800
H	4.51815600	-0.78358200	2.17644900
C	4.03605800	-1.00327500	0.28275800
C	3.19136100	-2.26391100	0.52472300
H	3.05819100	-2.84458900	-0.39375700
H	3.69444300	-2.91000400	1.25259400
H	2.20682000	-2.01519000	0.93111100
C	5.44860900	-1.41704900	-0.16068100
H	6.10522800	-0.55260300	-0.28034300
H	5.89629000	-2.07605800	0.59114300
H	5.42055000	-1.96517900	-1.10768200
H	3.38260900	-0.59435900	-1.72318100

Table S33: Optimized geometry for TS_{2c}

Free energy G = -1062.000076 Hartree/particle.

C	1.65532300	1.87621900	0.04237300
C	0.73069200	2.93127300	-0.57382900
H	1.13180300	3.92943400	-0.37606700
H	0.64152600	2.79531000	-1.65563900
H	-0.27325600	2.86371100	-0.14013000
O	1.75037400	2.05901700	1.45687700
H	0.85263400	2.12597900	1.81309500
C	3.08101200	2.02269200	-0.51281000
H	3.03511100	2.00265500	-1.60740200
H	3.42482200	3.02020600	-0.22163100
C	4.09227300	0.98051600	-0.00588100
H	5.09637000	1.33144000	-0.27909000
H	4.06043300	0.94908900	1.08540200
C	3.91759200	-0.38992100	-0.60873000
H	3.88656600	-0.41121600	-1.69876600
C	4.04405300	-1.59277100	0.02438100
C	4.21396100	-1.75922900	1.50812200
H	4.08118500	-0.83350800	2.06777900

H	3.50891200	-2.50538900	1.89257100
H	5.21947600	-2.14465900	1.72066900
C	4.01628100	-2.87191200	-0.76618800
H	3.19700900	-3.52271300	-0.43013000
H	3.91054000	-2.69706300	-1.83954200
H	4.93957000	-3.43926300	-0.59706900
C	1.13086900	0.46550400	-0.22033100
C	1.62284600	-0.68484800	-0.42689400
H	1.53552300	-1.73092600	-0.64506700
P	-3.25978900	-0.41697500	0.09499300
C	-4.11596400	0.84225700	1.11929000
H	-3.69873700	0.83844800	2.12903400
H	-3.96823900	1.83186500	0.68048500
H	-5.18691000	0.62528200	1.16938500
C	-4.12908500	-0.39711200	-1.52122300
H	-3.98339700	0.57334200	-2.00118800
H	-3.71645400	-1.17488200	-2.16796000
H	-5.19886800	-0.57428700	-1.37797800
C	-3.68412000	-2.03251000	0.85508000
H	-3.26103100	-2.84079700	0.25417000
H	-3.26242300	-2.08423600	1.86151600
H	-4.77008700	-2.15120700	0.90956400
Au	-0.95902100	-0.04221600	-0.08995000

Table S34: Optimized geometry for IIIc

Free energy G = -1062.019139 Hartree/particle.

P	3.21481700	0.29810200	0.06298100
C	4.02434100	0.69923800	-1.53805700
H	5.09912500	0.84459600	-1.39553900
H	3.86099700	-0.11669500	-2.24611400
H	3.58734900	1.61244100	-1.94915900
C	3.69514000	1.67596000	1.18067600
H	3.24395100	2.60623300	0.82724300
H	3.32960900	1.47145700	2.18988700
H	4.78297800	1.78835700	1.20526800
C	4.13271100	-1.16207400	0.69708200
H	3.77011700	-1.41438000	1.69657800
H	3.95937800	-2.01525100	0.03670200
H	5.20522500	-0.95273000	0.74359700
Au	0.87552700	-0.03709500	-0.09470500
C	-1.93351000	0.77369200	-0.63730800
C	-1.84338900	-1.62766000	0.15574900
C	-3.47976300	0.65303300	-0.93212900
H	-1.42594800	1.62906600	-1.06564700
C	-3.03811500	-1.85346200	-0.80074300
C	-4.07929600	-0.73725100	-0.71316200
H	-3.68711600	1.09517400	-1.90736200
H	-3.50569700	-2.81663800	-0.56471900
H	-2.65334900	-1.93327600	-1.82415200
H	-4.58037700	-0.78469200	0.25595900
H	-4.84953900	-0.89280600	-1.47415000
C	-1.17535600	-0.28269300	-0.18142400
C	-0.86019100	-2.79721500	0.04293000
H	-0.02845700	-2.66678500	0.74060600
H	-1.37113300	-3.73896300	0.27556300
H	-0.45564900	-2.87705900	-0.97043500
O	-2.28556500	-1.50732500	1.52041900
H	-2.61797000	-2.37285900	1.80119500
C	-3.38739700	1.61313000	0.14775300
C	-3.30606600	3.07705200	-0.18653400

H	-4.29104300	3.50941500	0.03363700
H	-2.57846900	3.59631800	0.44284600
H	-3.08316800	3.26097400	-1.23903200
C	-3.66773000	1.29574700	1.58541200
H	-3.10998500	1.96369600	2.24636100
H	-4.73682300	1.49578500	1.74638000
H	-3.45667400	0.26125500	1.84667000

Table S35: Optimized geometry for TS_{4c}

Free energy G = -1062.010532 Hartree/particle.

C	4.54487900	0.24952200	0.23682900
C	3.53339600	-0.56260100	-0.54756100
C	2.40420500	1.48981700	0.07798000
C	3.73434600	1.40807400	0.86646000
H	5.06916700	-0.35793200	0.98070100
H	5.30494600	0.61685700	-0.46095200
H	4.27332500	2.35701700	0.83029200
H	3.50106800	1.20414200	1.91328200
C	2.47144300	2.50165800	-1.09010900
H	1.55143900	2.48077400	-1.68343000
H	2.58935700	3.49984500	-0.65966700
H	3.31789600	2.30601100	-1.75472300
C	2.28921500	0.13415600	-0.63133600
C	1.13686400	-0.71211900	-0.79506600
H	1.17822400	-1.33608100	-1.68847500
P	-3.09650900	0.21254800	0.21845500
C	-3.63010400	1.92745200	-0.16965200
H	-4.68689700	2.06494300	0.07681800
H	-3.03005500	2.63644700	0.40567800
H	-3.47911700	2.12404300	-1.23395000
C	-4.27991300	-0.85697900	-0.69448400
H	-4.14779600	-0.71273400	-1.76951700
H	-4.08513900	-1.90536900	-0.45595500
H	-5.30913000	-0.60865800	-0.41986500
C	-3.52821200	-0.02279800	1.98938500
H	-3.31311300	-1.05322600	2.28275000
H	-2.92554200	0.64884700	2.60571000
H	-4.58869100	0.18657600	2.15675300
Au	-0.83410600	-0.23719300	-0.28416600
O	1.35706700	1.81783700	0.96689300
H	0.51063300	1.75474500	0.49081200
C	2.23614800	-1.41824300	0.21125900
C	2.43523200	-2.88618700	-0.21199600
H	3.29187900	-3.30749500	0.32206100
H	1.53666200	-3.44333900	0.06892900
H	2.59408800	-3.00227200	-1.28413000
C	2.05776500	-1.27261000	1.71592400
H	1.71927700	-0.28008200	2.00861000
H	1.30456400	-1.99607300	2.04279900
H	2.99096300	-1.50843400	2.23768600
H	3.88588500	-1.23609300	-1.32083400

Table S36: Optimized geometry for Vc

Free energy G = -1062.074679 Hartree/particle.

C	-4.13359600	-0.76185200	1.46078300
C	-2.65118400	-1.28101200	-0.43929000
C	-3.46611500	-1.93458500	0.70846200
H	-4.19366400	-0.92273200	2.54309300

H	-5.16403800	-0.58997500	1.11866200
H	-4.18157600	-2.67817700	0.34631200
H	-2.75553100	-2.44128200	1.36870400
C	-3.40943500	-1.28867000	-1.77520700
H	-2.86401200	-0.73969700	-2.54877200
H	-3.54721200	-2.31880200	-2.12437000
H	-4.40046300	-0.83832700	-1.66456200
C	-2.42531800	0.14144000	0.08179100
P	2.71132600	-0.77388300	0.22617000
C	2.77614600	-2.46694000	-0.47673600
H	3.74906700	-2.91947100	-0.26421800
H	1.98651300	-3.07973000	-0.03648500
H	2.62656500	-2.41963800	-1.55783100
C	4.13199300	0.11079400	-0.52372500
H	3.97564200	0.20310600	-1.60099100
H	4.21261200	1.11057800	-0.09119800
H	5.05698700	-0.44217500	-0.33675300
C	3.13250300	-0.96662800	2.00045100
H	3.18033500	0.01671700	2.47340800
H	2.36078000	-1.55959700	2.49676100
H	4.09955400	-1.46763200	2.10276400
Au	0.67327700	0.29539400	-0.12451300
O	-1.36401900	-1.89948900	-0.61421100
H	-1.49000400	-2.73509200	-1.08532200
C	-3.26351400	0.41278900	1.09986600
H	-3.36935500	1.37788300	1.58223500
C	-0.86255200	2.18536900	-0.13482400
C	-0.97025100	2.66956000	1.28991900
H	-1.80619700	3.37771000	1.36694300
H	-1.15043300	1.85964500	1.99850000
H	-0.06578600	3.21002500	1.58223800
C	-0.26666000	3.17687500	-1.10839600
H	-0.16269300	2.76178400	-2.11332500
H	-0.93145900	4.04917300	-1.16560000
H	0.70653300	3.54152000	-0.76737600
C	-1.48869600	1.03977300	-0.61201800
H	-1.43867300	0.89071900	-1.69159200

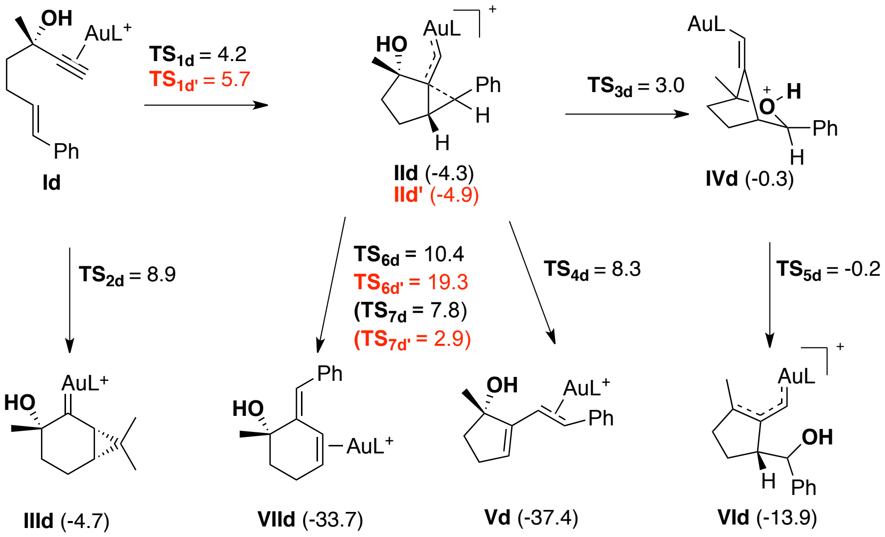

Scheme S4. Reaction pathways and energies for the the cyclization of **Id** calculated at the B3LYP/6-31G(d,p) (C, H, P, O), SDD(Au) level taking into account solvent effect of CH_2Cl_2 (IEF-PCM) and employing PMe_3 as the phosphine. ΔG energies are given in kcal·mol-1.

Table S37: Optimized geometry for Id

Free energy G = -1214.764723 Hartree/particle.

C	-3.17810200	0.99257100	0.16754400
C	-4.59339200	0.72565300	-0.38139000
H	-4.80940500	1.36472000	-1.24122600
H	-4.69988200	-0.31775500	-0.69356200
H	-5.31982000	0.93601400	0.40792100
C	-3.04421200	2.47340600	0.59363200
H	-3.92858200	2.68898600	1.20258600
H	-3.11819100	3.09685600	-0.30433600
C	-1.79405300	2.85433200	1.40909800
H	-1.96722600	3.88135900	1.76247700
H	-1.73031300	2.22467500	2.30153300
C	-2.20706100	0.65461000	-0.92590600
C	-1.58001900	0.45364000	-1.96575600
H	-1.16838500	0.42850400	-2.95500400
O	-2.92555300	0.19647200	1.32261300
H	-3.23493500	-0.70471700	1.14891600
C	-0.49392900	2.82078900	0.65615600
H	-0.48342300	3.35207700	-0.29551600
C	0.62438100	2.22828000	1.10682400
H	0.57838700	1.72260100	2.07195400
C	1.94151600	2.18051300	0.45097800
C	3.01475600	1.57370000	1.13108000
C	2.19090900	2.70970300	-0.83139400
C	4.28555300	1.49549700	0.55869700
H	2.84863300	1.17103000	2.12742000
C	3.45862200	2.63035900	-1.40408300
H	1.39058500	3.19051100	-1.38566400
C	4.51331100	2.02279300	-0.71405400
H	5.09718300	1.02897300	1.10970500
H	3.62676400	3.04781200	-2.39283500
H	5.50032700	1.96608500	-1.16304400
P	0.96916700	-2.43553700	0.45589800
C	2.67621400	-2.31248200	-0.19779500
H	2.66673300	-2.47656700	-1.27773800
H	3.07279100	-1.31526900	0.00785700
H	3.31174900	-3.06481100	0.27832700
C	1.11762600	-2.24563100	2.27311300
H	1.49416200	-1.24716300	2.50581200
H	0.13720100	-2.37129000	2.73742900
H	1.80631700	-2.99702400	2.66986100
C	0.44714500	-4.17087200	0.17918700
H	-0.54349000	-4.32770900	0.61195100
H	0.40040300	-4.37246500	-0.89337300
H	1.16089100	-4.85514000	0.64705200
Au	-0.47464700	-0.88114200	-0.49422100

Table S38: Optimized geometry for TS_{1d}

Free energy G = -1214.75796 Hartree/particle.

C	1.26992500	2.65733300	0.30178700
C	-0.17122800	3.21414700	0.28835200
H	-0.12765700	4.26310000	0.59294900
H	-0.61565700	3.14690300	-0.70720600
H	-0.80512700	2.66802400	0.99243300
O	1.85120400	2.79074700	1.59690700
H	1.30821500	2.29829500	2.22947800
C	2.17350800	3.44582500	-0.65860400

H	1.73023900	3.41464500	-1.65906600
H	2.20675500	4.49072400	-0.33670500
C	3.58140800	2.82322500	-0.66737000
H	4.16690200	3.25186000	-1.48799100
H	4.09109600	3.06290400	0.26897500
C	3.47868000	1.33293300	-0.83012400
H	3.25691200	0.96439500	-1.82939100
C	3.78654500	0.45611900	0.16953100
C	1.24014000	1.22148500	-0.10704800
C	0.68092700	0.09225100	-0.30625500
H	1.19059800	-0.83021600	-0.58217500
P	-3.70017200	-0.57144200	0.06770500
C	-4.59932800	0.65215000	1.10008900
H	-4.18551500	0.65157200	2.11128400
H	-4.47884300	1.64968700	0.67098600
H	-5.66372700	0.40347400	1.14414900
C	-4.56611500	-0.56105200	-1.55107900
H	-4.44490600	0.41672000	-2.02295300
H	-4.13126500	-1.32216700	-2.20308100
H	-5.63125800	-0.76771100	-1.41281400
C	-4.08797300	-2.20482600	0.80927800
H	-3.63968600	-2.99599000	0.20379700
H	-3.67172500	-2.25607300	1.81807000
H	-5.17078100	-2.35255900	0.85552700
Au	-1.40332800	-0.15301500	-0.10984900
H	4.04541500	0.87883100	1.13887300
C	3.79163400	-0.99864300	0.09974400
C	4.02424000	-1.72798400	1.28455100
C	3.56103500	-1.71483000	-1.09535900
C	4.02062500	-3.11953400	1.27982700
H	4.20744800	-1.18885700	2.20990000
C	3.55594300	-3.10817100	-1.09619700
H	3.41006600	-1.18415000	-2.02981800
C	3.78331100	-3.81394500	0.08913300
H	4.20303100	-3.66418900	2.20097600
H	3.38302600	-3.64468100	-2.02398100
H	3.78263800	-4.89961800	0.08348800

Table S39: Optimized geometry for IId

Free energy G = -1214.771505 Hartree/particle.

C	0.92070300	2.53487200	0.40744700
C	2.19308600	3.40800000	0.56846900
C	3.11255300	3.01936300	-0.59886900
C	2.87255900	1.51803700	-0.81563500
C	1.39244700	1.27623500	-0.33912300
H	2.65665900	3.17485400	1.53223200
H	1.96058900	4.47608000	0.57497600
H	2.82560500	3.54458400	-1.51458400
H	4.16624700	3.23850900	-0.40775500
H	3.08771800	1.14890400	-1.81518600
C	-0.14724400	3.26216000	-0.42703000
H	-0.99786800	2.60247200	-0.61851900
H	-0.49872400	4.14578900	0.11805600
H	0.25675100	3.59860500	-1.38660200
O	0.38016500	2.13356500	1.66864100
H	-0.00799400	2.91623200	2.08522900
C	3.20623000	0.62600300	0.27395100
H	3.18581900	1.04467700	1.27640000
C	3.63118700	-0.74785000	0.18394400
C	3.91481400	-1.38334800	-1.04766800

C	3.82417000	-1.46672100	1.38716700
C	4.36950300	-2.69602100	-1.06712000
H	3.79282500	-0.84816200	-1.98272200
C	4.28232000	-2.77723200	1.36016000
H	3.61040700	-0.98190200	2.33520100
C	4.55211400	-3.39394600	0.13285200
H	4.58955300	-3.17732800	-2.01424000
H	4.42987800	-3.32058200	2.28752100
H	4.91014200	-4.41855200	0.11091300
C	0.66403400	0.16294800	-0.61858500
H	1.23129600	-0.64647100	-1.08313200
Au	-1.31446300	-0.21519200	-0.24817600
P	-3.58387000	-0.75300300	0.16075500
C	-4.72209800	-0.25428900	-1.19421200
H	-5.75141300	-0.53712700	-0.95521000
H	-4.66865700	0.82777600	-1.33624100
H	-4.41912800	-0.74394200	-2.12283100
C	-3.89128700	-2.55158000	0.38368700
H	-3.30844600	-2.91871600	1.23202100
H	-4.95344200	-2.73982100	0.56603600
H	-3.57753600	-3.08941000	-0.51425100
C	-4.28921900	0.02546800	1.66926200
H	-5.33378400	-0.27067900	1.80199800
H	-3.71213600	-0.28461700	2.54371100
H	-4.23026800	1.11289300	1.58021900

Table S40: Optimized geometry for TS_{3d}

Free energy G = -1214.760013 Hartree/particle.

C	1.26451800	2.13028800	0.62344400
C	1.89839700	3.37650300	-0.03231200
C	2.75169400	2.85689700	-1.22605100
C	2.68640000	1.31456000	-1.12764900
C	1.26324000	1.08952200	-0.49140100
H	2.49183300	3.91589600	0.71077200
H	1.11174900	4.05063000	-0.37976800
H	2.31267000	3.16580800	-2.17770300
H	3.77967700	3.22957300	-1.20587600
H	2.73361400	0.79986900	-2.08994800
C	-0.04661600	2.40438900	1.34654300
H	-0.42691800	1.50136200	1.83355700
H	0.11053300	3.17312900	2.10892000
H	-0.80699500	2.75504200	0.64446800
O	2.26310500	1.66697100	1.58032300
H	1.91114800	0.86522400	2.00000500
C	3.70832200	0.77995000	-0.20386100
H	4.29309100	1.50223300	0.35688500
C	4.09807200	-0.57113400	-0.04022000
C	5.14550200	-0.85523900	0.88041100
C	3.50864300	-1.64330200	-0.76689800
C	5.58315600	-2.15640100	1.06858200
H	5.59586100	-0.03843000	1.43594300
C	3.95145300	-2.93928300	-0.57069700
H	2.71588600	-1.44334500	-1.47701600
C	4.98541800	-3.19592300	0.34467300
H	6.38250700	-2.36947700	1.76966100
H	3.50630200	-3.75700200	-1.12685400
H	5.32912000	-4.21560800	0.48900200
C	0.34396300	0.22463600	-0.92657300
H	0.66720200	-0.40835000	-1.75849200
Au	-1.59276200	-0.14047900	-0.32197500

P	-3.81797100	-0.64743000	0.30096800
C	-4.95551700	-0.93583400	-1.11607200
H	-5.96641100	-1.15652900	-0.76021000
H	-4.98216100	-0.04563600	-1.74916100
H	-4.59069200	-1.77621500	-1.71182900
C	-3.98954600	-2.16028500	1.33347200
H	-3.41336900	-2.04157100	2.25444700
H	-5.03950000	-2.33704100	1.58458600
H	-3.59898700	-3.02211500	0.78684500
C	-4.64681800	0.66684800	1.28576500
H	-5.66696300	0.36845300	1.54424200
H	-4.08083200	0.84735800	2.20296100
H	-4.67715200	1.59309100	0.70658900

Table S41: Optimized geometry for IVd

Free energy G = -1214.765179 Hartree/particle.

C	1.33129300	1.83907400	0.69277400
C	1.79245000	3.22125600	0.21686200
C	2.55980600	2.92336600	-1.11032600
C	2.61349100	1.37355100	-1.13595900
C	1.23671500	1.02522500	-0.56741800
H	2.40306200	3.71632100	0.97649000
H	0.90650900	3.83982700	0.05006500
H	2.00006500	3.28615700	-1.97506900
H	3.55240300	3.38132900	-1.13410300
H	2.85591100	0.93529100	-2.10394200
C	0.35434600	1.75397400	1.82942400
H	0.16245800	0.72037200	2.12895600
H	0.70612700	2.32937400	2.68946800
H	-0.59458900	2.18169100	1.49452100
O	2.73567400	1.24117300	1.24282900
H	2.56401500	0.39796100	1.70665900
C	3.59796800	0.92453100	-0.03835000
H	4.42511600	1.62231500	0.08789500
C	4.09720700	-0.48513200	0.02502700
C	5.38240300	-0.70817500	0.54714500
C	3.33849200	-1.58183200	-0.41909000
C	5.90138300	-1.99970100	0.62540100
H	5.97815600	0.13527500	0.88543700
C	3.86112800	-2.87188100	-0.34051800
H	2.34539500	-1.42784500	-0.82438300
C	5.14005000	-3.08331200	0.18183500
H	6.89708700	-2.15868600	1.02707000
H	3.27014700	-3.71270100	-0.69006700
H	5.54281000	-4.08995400	0.23998600
C	0.22793900	0.30639800	-1.08106100
H	0.46273000	-0.13074300	-2.05703600
Au	-1.66416500	-0.08547000	-0.37436600
P	-3.86388200	-0.59938000	0.33845400
C	-5.16777600	0.46147100	-0.40852100
H	-6.15660700	0.16969600	-0.04287300
H	-4.98217500	1.50716100	-0.15129800
H	-5.14020300	0.36030700	-1.49620200
C	-4.39105200	-2.31501800	-0.06258800
H	-3.71965900	-3.02625600	0.42491200
H	-5.41547200	-2.49425700	0.27669500
H	-4.33644200	-2.46906600	-1.14304300
C	-4.14335100	-0.45093100	2.15067800
H	-5.17815600	-0.70134500	2.40199500
H	-3.46937500	-1.12840300	2.68081000

H	-3.93238100	0.57248100	2.47064100

Table S42: Optimized geometry for TS_{5d}

Free energy G = -1214.765017 Hartree/particle.

C	-3.62151800	-0.86291400	0.18458300
C	-2.74654400	-1.36449000	-0.99875500
H	-3.01971100	-0.87288700	-1.93237300
O	-2.79331300	-1.25324900	1.36291100
C	-1.29071400	-2.03263300	0.63939900
C	-2.80398500	-2.91218300	-1.03817600
H	-3.82355800	-3.29846300	-0.95509900
H	-2.38494400	-3.27510000	-1.97921300
C	-1.90793100	-3.33501400	0.16474600
H	-1.08503600	-3.99181700	-0.14111700
H	-2.44140100	-3.86178100	0.96164700
H	-3.28883100	-1.83160400	1.96756600
C	-1.32829800	-1.10665700	-0.50873900
C	-0.37785700	-0.27689700	-0.98256100
H	-0.69546500	0.26410400	-1.87928600
Au	1.55252300	0.08063900	-0.38106600
C	4.89824000	-0.92395900	0.22219500
H	5.92001800	-0.63952600	0.49022300
H	4.52642500	-1.65555200	0.94377600
H	4.89970400	-1.38181600	-0.77004200
C	4.00743800	1.28495700	1.88738900
H	5.06476100	1.47518900	2.09396800
H	3.45394000	2.22555700	1.94445700
H	3.61063300	0.60014600	2.64088500
C	4.63489900	1.73594600	-0.91277600
H	4.08829200	2.68206700	-0.91758000
H	5.66422700	1.91450600	-0.58813200
H	4.64124700	1.33154800	-1.92788000
P	3.79715900	0.54925500	0.21466900
C	-0.30362700	-2.00729200	1.75383600
H	0.62596000	-2.45018000	1.37763700
H	-0.08667700	-0.99009800	2.08123200
H	-0.64098700	-2.61357300	2.59826900
H	-4.55311100	-1.43162000	0.24151000
C	-3.90895200	0.61609200	0.21783000
C	-5.05558200	1.08157300	-0.44165500
C	-3.05810000	1.53513200	0.84447100
C	-5.33820000	2.44644500	-0.48952900
H	-5.72875800	0.37426000	-0.91989200
C	-3.34865600	2.90103500	0.80308500
H	-2.18294600	1.18423500	1.37899400
C	-4.48408500	3.36039800	0.13343000
H	-6.22884400	2.79435000	-1.00406500
H	-2.68677000	3.60487800	1.29906100
H	-4.70755400	4.42259900	0.10369800

Table S43: Optimized geometry for VId

Free energy G = -1214.786797 Hartree/particle.

C	-3.64106700	-2.57046300	-0.75714100
C	-3.11893800	-1.11324900	-0.78608500
C	-1.63508700	-1.25708800	-0.43203800
C	-1.41127600	-2.50851400	0.16413700
C	-2.66665800	-3.31569500	0.17484500
H	-3.58955200	-3.00017000	-1.76205200

H	-3.24301200	-0.66151800	-1.77445300
H	-3.02606400	-3.31643800	1.21516900
H	-2.48290400	-4.36373500	-0.08724600
H	-4.67612900	-2.63990000	-0.41619500
C	-0.15977600	-3.06605800	0.71945800
H	-0.36648100	-3.53518400	1.68853300
H	0.18655700	-3.87531600	0.06170000
H	0.63722700	-2.32828700	0.81913000
C	-3.86469000	-0.19898100	0.22522400
H	-4.92045900	-0.19109500	-0.08569900
C	-3.35032500	1.23308000	0.21055700
C	-2.44360000	1.68885100	1.17778300
C	-3.76581800	2.11558900	-0.79752400
C	-1.95816100	3.00027600	1.13427200
H	-2.13773800	1.01801800	1.97369600
C	-3.27634400	3.42061900	-0.84601500
H	-4.48162400	1.78108400	-1.54495600
C	-2.36847200	3.86719500	0.12072800
H	-1.26585700	3.34309700	1.89807900
H	-3.61045300	4.09361300	-1.63031200
H	-1.99538500	4.88653400	0.08847600
O	-3.73684600	-0.81355400	1.50442000
H	-4.27991500	-0.31852400	2.13309300
C	-0.66841600	-0.29124600	-0.69962700
H	-1.07778100	0.53892000	-1.28214600
Au	1.31299200	-0.08510600	-0.27732200
P	3.61495200	0.29197400	0.16417600
C	4.66340400	-1.21129500	0.03122300
H	5.70876200	-0.96765500	0.24177800
H	4.31681500	-1.96225100	0.74534500
H	4.58489600	-1.62402600	-0.97744900
C	4.39270500	1.51069300	-0.96926800
H	3.87169900	2.46759200	-0.88745600
H	5.44794700	1.64936300	-0.71690400
H	4.31116100	1.15506900	-1.99912800
C	3.94284900	0.94298500	1.85133300
H	3.57232100	0.23408100	2.59566900
H	5.01597600	1.09436700	1.99878000
H	3.42217200	1.89436600	1.98377800

Table S44: Optimized geometry for TS_{2d}

Free energy G = -1214.750584 Hartree/particle.

C	-0.05769300	2.78452700	-0.04238800
C	1.22651700	3.29536900	0.61819000
H	1.28552400	4.38216200	0.51022200
H	1.24858800	3.04577800	1.68303900
H	2.10876300	2.84811300	0.14710600
O	-0.07410700	3.11731900	-1.43153300
H	0.76744900	2.83818400	-1.82025800
C	-1.28779600	3.46300500	0.58409400
H	-1.26014200	3.30579200	1.66798900
H	-1.17422100	4.53780800	0.41062900
C	-2.64487400	3.01453300	0.01788300
H	-3.40619800	3.70717100	0.40154300
H	-2.64318600	3.11969400	-1.07149700
C	-3.07308900	1.62111300	0.40153400
H	-3.11283400	1.40920400	1.46949500
C	-3.67021200	0.76598000	-0.47982700
C	-0.18870600	1.26673500	0.08759000
C	-1.14718600	0.43747100	0.15361900

H	-1.54441300	-0.55623400	0.23936000
P	3.40553400	-1.41894500	-0.04082900
C	4.84618100	-0.63736700	0.78432000
H	5.07790000	0.31189900	0.29580200
H	4.60482100	-0.44388500	1.83208700
H	5.71852200	-1.29476800	0.72839900
C	3.19985400	-3.04562200	0.78432100
H	2.92857900	-2.89082200	1.83114500
H	2.40133000	-3.60645900	0.29318000
H	4.13060500	-3.61787100	0.73122400
C	3.97142500	-1.80410900	-1.74368900
H	3.18539700	-2.34414700	-2.27671500
H	4.18340500	-0.87449100	-2.27691300
H	4.87610000	-2.41796400	-1.71272200
Au	1.48319700	-0.08934200	0.03356400
H	-3.67005700	1.05809100	-1.52959600
C	-4.27555200	-0.53053000	-0.19877100
C	-4.73749100	-1.30385100	-1.28412500
C	-4.41363800	-1.05426100	1.10502400
C	-5.31623700	-2.55356000	-1.07788900
H	-4.63900500	-0.91245600	-2.29295500
C	-4.98861900	-2.30499900	1.30744600
H	-4.08364000	-0.47774400	1.96334800
C	-5.44185900	-3.05863800	0.21869600
H	-5.66819200	-3.13286700	-1.92584800
H	-5.09057700	-2.69295100	2.31630800
H	-5.89335400	-4.03234800	0.38258100

Table S45: Optimized geometry for IIId

Free energy G = -1214.772281 Hartree/particle.

P	-3.36145500	-1.26451100	-0.01875300
C	-4.40509500	-1.10644100	-1.52373100
H	-5.29699900	-1.73367700	-1.43722200
H	-4.70775200	-0.06410900	-1.65000600
H	-3.83036100	-1.41286400	-2.40105300
C	-3.04993300	-3.06777400	0.14712900
H	-2.45552900	-3.41507200	-0.70137100
H	-2.49163600	-3.25899300	1.06676300
H	-3.99491500	-3.61803700	0.17649300
C	-4.48855000	-0.85451300	1.37316600
H	-3.96533900	-0.99496900	2.32205200
H	-4.79904900	0.19033500	1.29599100
H	-5.37301100	-1.49741000	1.34890600
Au	-1.38786700	0.04692400	-0.08633000
C	1.46050500	0.60160900	-0.78445300
C	0.48051100	2.54834400	0.45697400
C	2.75095800	1.42676400	-1.06707600
H	1.38113500	-0.34474700	-1.30559700
C	1.20681500	3.42181300	-0.59881700
C	2.63835300	2.94509400	-0.86889000
H	3.20579500	1.11146200	-2.00336300
H	1.21992600	4.46267500	-0.25509100
H	0.61866700	3.40512700	-1.52345200
H	3.28693900	3.24309700	-0.04108300
H	3.01917600	3.43929600	-1.76669100
C	0.35865400	1.13530000	-0.13410200
C	-0.87643200	3.15633000	0.81793400
H	-1.39178600	2.53088000	1.55189100
H	-0.73729400	4.15656400	1.24464100
H	-1.51273500	3.25355000	-0.06673800

O	1.27739200	2.41390900	1.65029700
H	1.34598700	3.28893200	2.06000800
C	3.00941700	0.58457800	0.08704000
C	3.67454200	-0.71957900	0.09930900
C	3.87977200	-1.34393200	1.34577600
C	4.13955300	-1.35763800	-1.06778500
C	4.53754900	-2.56832300	1.42527200
H	3.52252300	-0.85837100	2.24947500
C	4.79221700	-2.58343100	-0.98459700
H	3.99899900	-0.89676300	-2.04019000
C	4.99325300	-3.19097900	0.26003800
H	4.69388300	-3.03670500	2.39176800
H	5.15039800	-3.06534900	-1.88872200
H	5.50525300	-4.14647500	0.31971000
H	2.83932900	1.04770400	1.05277900

Table S46: Optimized geometry for TS_{4d}

Free energy G = -1214.751493 Hartree/particle.

C	3.47614900	2.62790600	0.22645200
C	2.88869800	1.47469900	-0.55651500
C	1.00533600	2.78719700	0.21962100
C	2.27398400	3.21050100	1.00161500
H	4.29832300	2.31376800	0.87459100
H	3.88937600	3.34865000	-0.48834000
H	2.33206500	4.29442600	1.11753800
H	2.20980400	2.77485300	2.00241400
C	0.55587100	3.83859900	-0.81514700
H	-0.29112000	3.47276700	-1.40428200
H	0.24562400	4.73709300	-0.27522500
H	1.36385600	4.10680900	-1.50224200
C	1.46555200	1.57652500	-0.60653000
C	0.79357100	0.31259700	-0.77953900
H	1.13365700	-0.23725300	-1.65817900
P	-3.40173700	-0.75683200	0.23053200
C	-4.58098000	-0.41501300	-1.13706100
H	-5.59249400	-0.72420300	-0.85839000
H	-4.57896400	0.65420600	-1.36265400
H	-4.26940900	-0.96144100	-2.03060700
C	-3.61462800	-2.54573800	0.59266000
H	-3.29264500	-3.13442800	-0.26967700
H	-2.99982700	-2.82000700	1.45328600
H	-4.66322500	-2.76743800	0.81163800
C	-4.11612100	0.09737600	1.69255800
H	-3.50687300	-0.11810200	2.57370500
H	-4.11744600	1.17678100	1.52338300
H	-5.14076500	-0.24184500	1.87009200
Au	-1.17830300	-0.14991900	-0.26778000
O	-0.01873700	2.50648000	1.15117900
H	-0.79468300	2.16707900	0.67278400
C	2.05521500	0.19101400	0.24928400
H	3.46872400	0.97360400	-1.32455300
H	1.81654900	0.42677800	1.28132000
C	2.86987600	-1.08062100	0.13121900
C	3.27278500	-1.67469200	1.33511900
C	3.22155200	-1.68310700	-1.08266800
C	4.00922200	-2.86095100	1.32311600
H	3.00819800	-1.21380300	2.28218400
C	3.96015000	-2.86665200	-1.08922600
H	2.92948900	-1.24176800	-2.03108000
C	4.35524800	-3.46047500	0.11191800

H	4.31078700	-3.31136100	2.26378100
H	4.22546800	-3.32368400	-2.03746200
H	4.92852000	-4.38219000	0.10197100

Table S47: Optimized geometry for Vd

Free energy G = -1214.824362 Hartree/particle.

C	3.97892500	-1.87571600	1.56653500
C	3.14531600	-1.11879600	-0.63245600
C	4.33428200	-0.97904600	0.35742700
H	4.29773400	-1.45179400	2.52557700
H	4.45364300	-2.86481500	1.49670700
H	5.29429300	-1.23408200	-0.09994800
H	4.37753100	0.06790900	0.67313500
C	3.42347800	-2.14139100	-1.74490800
H	2.54063800	-2.29752900	-2.37236500
H	4.23833100	-1.78863200	-2.38788700
H	3.72404700	-3.10543100	-1.32369200
C	2.00878300	-1.57290500	0.28282900
P	-0.54364900	2.92019200	-0.00742100
C	0.70686700	3.81452700	-1.00711500
H	0.48464800	4.88557000	-1.01551600
H	1.69816100	3.65198400	-0.57831400
H	0.69813700	3.43330900	-2.03075600
C	-2.16802300	3.35701300	-0.73637600
H	-2.22268800	2.97414300	-1.75799500
H	-2.96701400	2.90240500	-0.14637800
H	-2.29595800	4.44303500	-0.74748800
C	-0.50919700	3.71298500	1.64536300
H	-1.27994100	3.27133800	2.28087900
H	0.46687400	3.54817900	2.10710700
H	-0.68985600	4.78762700	1.54880800
Au	-0.17271900	0.62395000	0.06660700
O	2.76537100	0.13822100	-1.21732200
H	3.42531600	0.37118300	-1.88558700
C	2.48366200	-2.00976500	1.46471700
H	1.88222900	-2.46368000	2.24672600
C	-0.49797700	-1.66898600	0.62736400
C	0.62031600	-1.58653400	-0.18847000
H	0.49234000	-1.70497300	-1.26308500
H	-0.33383800	-1.62736900	1.70375500
C	-1.87223600	-2.02071700	0.22748200
C	-2.86519200	-2.04755900	1.22480300
C	-2.23377600	-2.35083900	-1.09357200
C	-4.18158300	-2.38401000	0.91342800
H	-2.59668800	-1.80543200	2.24963900
C	-3.54942300	-2.68354200	-1.40190100
H	-1.48769700	-2.35695500	-1.88178400
C	-4.52846400	-2.69990900	-0.40165600
H	-4.93326500	-2.40204300	1.69645400
H	-3.81258400	-2.93920300	-2.42371700
H	-5.55236700	-2.96430800	-0.64729800

Table S48: Optimized geometry for TS_{6d}

Free energy G = -1214.748199 Hartree/particle.

C	-1.03238000	2.37925200	-0.59644700
C	-0.31097300	2.67926000	0.75086000
C	-1.09386900	1.94207500	1.85304300
C	-1.64373100	0.67317600	1.21699300

C	-1.60458900	0.95749800	-0.41732500
H	-0.26370300	3.75905900	0.91569700
H	0.71495200	2.30475400	0.70702300
H	-0.46043600	1.68482600	2.70655300
H	-1.92286500	2.54848100	2.23037700
H	-2.05685200	-0.12269200	1.82374800
C	-0.12311300	2.46979000	-1.82591700
H	-0.70056800	2.29212400	-2.73711100
H	0.30953100	3.47408600	-1.88189000
H	0.69428800	1.74644100	-1.77325000
O	-2.15949900	3.24208700	-0.75719400
H	-1.82685500	4.14062400	-0.88732200
C	-2.95010700	0.85551300	0.07159500
H	-3.46492700	1.80633400	0.17331000
C	-3.82994200	-0.34547000	-0.02532800
C	-4.85726100	-0.51968600	0.91533300
C	-3.70097900	-1.26433000	-1.07721700
C	-5.72451700	-1.60636900	0.81900800
H	-4.97502800	0.19670400	1.72392500
C	-4.57531700	-2.34807900	-1.17494600
H	-2.93093000	-1.12283600	-1.82953600
C	-5.58388100	-2.52436000	-0.22580700
H	-6.51265900	-1.73364700	1.55467900
H	-4.46995800	-3.05010500	-1.99636900
H	-6.26248400	-3.36833600	-0.30328800
C	-0.74836900	-0.11473500	0.05433700
H	-1.24214500	-1.08351700	0.00553900
Au	1.32769200	-0.32125700	0.04372800
P	3.65070300	-0.70777900	0.01281900
C	4.66092100	0.65015100	0.72888000
H	5.72547400	0.40416100	0.67527500
H	4.47548000	1.57443900	0.17625900
H	4.37836200	0.80379300	1.77313700
C	4.16108800	-2.20763100	0.94431600
H	3.66689100	-3.08540000	0.52081500
H	5.24535500	-2.34205200	0.89178000
H	3.85997800	-2.10781500	1.99001400
C	4.33280000	-0.95242000	-1.67617800
H	5.41148100	-1.12866600	-1.63289000
H	3.84467300	-1.80958200	-2.14621000
H	4.13606100	-0.06374700	-2.28074500

Table S49: Optimized geometry for TS_{7d}

Free energy G = -1214.752285 Hartree/particle.

C	-1.26756400	2.50686500	-0.59582300
C	-1.30456200	3.28628100	0.74110100
C	-0.72773000	2.41897400	1.88487700
C	-1.18118000	1.00305000	1.71348500
C	-1.69621000	1.06327200	-0.25851700
H	-2.35671100	3.49744400	0.95309900
H	-0.77065400	4.24009200	0.67490200
H	0.36748600	2.43003800	1.84487300
H	-1.04232700	2.80810300	2.85510500
H	-1.88434300	0.60725900	2.44953600
C	0.09509700	2.59660000	-1.29766700
H	0.10259300	1.97463700	-2.19686300
H	0.28027100	3.63541600	-1.59198200
H	0.91939000	2.28297200	-0.65227700
O	-2.26346900	3.04099600	-1.47308200
H	-2.02073700	3.95631100	-1.66794000

C	-2.94106900	0.63710000	-0.62326700
H	-3.50030800	1.34933000	-1.22477100
C	-3.64084200	-0.62851000	-0.36655600
C	-5.02260800	-0.56764200	-0.09050600
C	-3.03234900	-1.89611500	-0.45256000
C	-5.75459100	-1.72618200	0.15386800
H	-5.51415800	0.40058500	-0.05377600
C	-3.77364200	-3.05649000	-0.22819700
H	-1.99245300	-1.98078700	-0.74922100
C	-5.13084500	-2.97620500	0.08993400
H	-6.81364000	-1.65645200	0.38288800
H	-3.29022600	-4.02512300	-0.31269300
H	-5.70354700	-3.88111200	0.26885500
C	-0.76653000	0.17300400	0.63419100
H	-1.21707600	-0.81050600	0.74063700
Au	1.28707700	-0.19950700	0.18851900
P	3.51336100	-0.82265500	-0.21692400
C	3.99919700	-2.37079100	0.64359000
H	5.04208200	-2.61713700	0.42374800
H	3.87810900	-2.24089100	1.72167300
H	3.35619900	-3.19048600	0.31445500
C	3.89210000	-1.12743200	-1.98858200
H	3.71648300	-0.21442700	-2.56251400
H	4.93525700	-1.43434900	-2.10785000
H	3.23769200	-1.91390300	-2.37188000
C	4.75327200	0.42006700	0.32414500
H	5.76586400	0.06386200	0.11339000
H	4.58441300	1.36124200	-0.20441000
H	4.64895300	0.59575400	1.39741000

Table S50: Optimized geometry for VIId

Free energy G = -1214.818377 Hartree/particle.

C	1.33594700	2.61032400	0.75397500
C	0.92121300	3.54482200	-0.40511300
C	-0.20337900	2.98104300	-1.28700500
C	0.05407900	1.53440200	-1.65239900
C	1.75110000	1.25258000	0.17190700
H	1.81383900	3.71319400	-1.01781700
H	0.62341800	4.51542400	0.00672700
H	-1.17840900	3.08678600	-0.79500500
H	-0.27794700	3.56225800	-2.21255800
H	-0.31915000	1.17595300	-2.61035100
C	0.21661600	2.45814900	1.80076900
H	0.57360000	1.84869600	2.63537400
H	-0.07653500	3.44249800	2.18444800
H	-0.67963300	1.98289500	1.38856300
O	2.49579800	3.15276400	1.39349200
H	2.21775100	3.94155800	1.87944700
C	2.77060700	0.55276700	0.72977600
H	3.20752100	0.98955500	1.62460700
C	3.36348000	-0.72623700	0.30806900
C	3.72382200	-1.65671600	1.30358400
C	3.64369100	-1.04602300	-1.03480400
C	4.28850100	-2.88446900	0.96542300
H	3.54619800	-1.41234700	2.34745100
C	4.22013100	-2.27156000	-1.37039600
H	3.45530500	-0.31500800	-1.81459000
C	4.53430200	-3.19890300	-0.37465000
H	4.54653800	-3.59244300	1.74741100
H	4.43758500	-2.49452100	-2.41077100

H	4.98462900	-4.15117800	-0.63802100
C	0.99994800	0.77402500	-0.98857200
H	1.28875300	-0.18353000	-1.41087700
Au	-1.23019400	-0.00469700	-0.41330700
P	-2.97729500	-1.26985800	0.46471800
C	-4.62285100	-0.56066900	0.07635200
H	-5.40941000	-1.19904900	0.48889900
H	-4.70169400	0.43893900	0.50972900
H	-4.74492400	-0.48772000	-1.00662200
C	-3.00978500	-2.98368500	-0.18486000
H	-2.08205700	-3.49374600	0.08367800
H	-3.85966300	-3.52637600	0.23882200
H	-3.09990900	-2.96095500	-1.27324600
C	-2.92759200	-1.43021500	2.29044400
H	-3.77065500	-2.03631300	2.63457400
H	-1.99180300	-1.90622600	2.59144000
H	-2.98395400	-0.43933500	2.74627100

Table S51: Optimized geometry for TS1d'

Free energy G = -1214.75568 Hartree/particle.

C	-1.01732200	2.69402900	0.18119700
C	0.01197000	2.95826800	1.29954800
H	0.12617100	4.03908700	1.41698900
H	-0.31684400	2.52615700	2.24776800
H	0.98313500	2.52602200	1.04027400
O	-0.59227800	3.28156400	-1.04982000
H	0.26194000	2.89330300	-1.28964400
C	-2.37041500	3.33948900	0.51727900
H	-2.68241200	2.99850800	1.50932700
H	-2.23361700	4.42354700	0.56469000
C	-3.41684300	2.94728600	-0.53781700
H	-4.40798800	3.28116700	-0.21159800
H	-3.19360100	3.45068300	-1.48021100
C	-3.40105300	1.45110100	-0.74745900
H	-3.17621100	1.09046600	-1.74721700
C	-3.82403000	0.57201800	0.20801200
C	-1.18523600	1.21864900	-0.01270800
C	-0.71573700	0.03927000	-0.12972200
H	-1.26250800	-0.87994000	-0.32844000
P	3.67514500	-0.66126500	0.02081400
C	4.46894000	-0.62120400	-1.63350500
H	3.98880200	-1.35247400	-2.28779200
H	4.35058600	0.37286900	-2.07075500
H	5.53404800	-0.85534600	-1.54795700
C	4.64989600	0.50689000	1.04783600
H	4.53741600	1.52060400	0.65641700
H	4.28271500	0.48320100	2.07653300
H	5.70779800	0.22900200	1.03369000
C	4.05396600	-2.32441400	0.69793900
H	3.67989800	-2.39393500	1.72203300
H	3.56046500	-3.08712900	0.09111500
H	5.13374500	-2.49878300	0.69342600
Au	1.37781900	-0.20781600	-0.03175200
H	-4.12425800	0.97844200	1.17372500
C	-3.90523500	-0.87620700	0.09861400
C	-4.27268200	-1.62026800	1.24026400
C	-3.61898700	-1.57603000	-1.09518800
C	-4.35056800	-3.00863000	1.19400900
H	-4.49654400	-1.09507500	2.16472600
C	-3.69560000	-2.96613600	-1.13690300

H	-3.35820200	-1.03287800	-1.99760500
C	-4.05998900	-3.68595200	0.00523300
H	-4.63690900	-3.56433400	2.08143000
H	-3.47848800	-3.48899200	-2.06317000
H	-4.12222400	-4.76917600	-0.03273600

Table S52: Optimized geometry for IId'

Free energy G = -1214.772564 Hartree/particle.

C	-0.85719100	2.45675900	0.42894200
C	-0.36174800	2.24337800	1.86134200
H	-0.02475200	3.19810900	2.27602600
H	-1.15175000	1.84534600	2.50551200
H	0.47673200	1.53965000	1.88055400
O	0.19640700	2.98510000	-0.38954500
H	0.88487300	2.30209000	-0.44875700
C	-2.02442000	3.45960800	0.33463800
H	-2.63505300	3.40981400	1.24175700
H	-1.63769600	4.47827600	0.26325200
C	-2.83848300	3.04756300	-0.90104600
H	-3.87406200	3.39649700	-0.87156900
H	-2.37651500	3.43661300	-1.81201900
C	-2.76369100	1.51695700	-0.95032500
H	-2.88519600	1.07274600	-1.93436300
C	-3.26963700	0.75455200	0.16108300
C	-1.37373200	1.17353800	-0.26581900
C	-0.66942300	0.03880100	-0.50577100
H	-1.23393400	-0.77682500	-0.96357800
P	3.64347800	-0.71510200	0.11462200
C	4.72621500	0.69853900	-0.33912600
H	4.58054000	0.94747400	-1.39311800
H	4.46147300	1.56914100	0.26594200
H	5.77672700	0.44622100	-0.16843800
C	4.10071900	-1.11356600	1.84928500
H	3.83244400	-0.27804900	2.50005900
H	3.55507600	-2.00190300	2.17635300
H	5.17577400	-1.30047700	1.92547100
C	4.27681300	-2.13096500	-0.87036200
H	3.72919700	-3.03673000	-0.59902900
H	4.12319000	-1.93456100	-1.93407700
H	5.34325800	-2.28278500	-0.68044700
Au	1.33144400	-0.29216200	-0.19651600
H	-3.33955400	1.26300200	1.11896100
C	-3.77701700	-0.59856900	0.13542300
C	-4.11165300	-1.21249900	1.36415500
C	-3.99450700	-1.30913200	-1.06688000
C	-4.63911300	-2.49722000	1.39075000
H	-3.95271200	-0.66934500	2.29124900
C	-4.52047300	-2.59567000	-1.03277200
H	-3.77217700	-0.85101400	-2.02432500
C	-4.84029300	-3.19169800	0.19235500
H	-4.89451300	-2.95970400	2.33841500
H	-4.68996800	-3.13380600	-1.95956200
H	-5.25307600	-4.19553600	0.21231400

Table S53: Optimized geometry for $TS_{6d'}$

Free energy G = -1214.733990 Hartree/particle.

C	1.40128500	1.32618300	0.35624900
C	2.66986200	2.17435700	0.48501400

H	2.42694700	3.05129600	1.09494300
H	3.48032800	1.64325600	0.98452200
H	3.01902300	2.52308000	-0.48890500
O	0.36821500	2.13328100	-0.21472400
H	0.27403300	2.92129500	0.33861600
C	0.97323000	0.70812600	1.73251600
H	1.38850200	1.28243100	2.56461800
H	-0.11615700	0.77289200	1.78723000
C	1.41050900	-0.76612800	1.77067400
H	2.40613200	-0.88014200	2.20739900
H	0.72613900	-1.37440800	2.36943200
C	1.40861200	-1.28706000	0.34027400
H	1.52672400	-2.34817800	0.15398900
C	2.65701500	-0.70685300	-0.76169000
C	1.49106300	0.10842400	-0.59397800
C	0.30288200	-0.73774100	-0.71775100
H	0.39183800	-1.43388600	-1.55330100
P	-3.97618800	0.09071300	0.10628600
C	-4.73414900	-1.04492900	1.33752100
H	-4.62149400	-2.07793900	0.99958600
H	-4.22505900	-0.93447200	2.29806800
H	-5.79722100	-0.81958300	1.46361900
C	-4.37417500	1.77391600	0.72980000
H	-3.85927000	1.94269300	1.67874200
H	-4.03226500	2.52059600	0.00899500
H	-5.45218500	1.88280200	0.87974900
C	-5.01468900	-0.10082300	-1.39816400
H	-4.68192300	0.60539100	-2.16273500
H	-4.90575300	-1.11521200	-1.78947600
H	-6.06708100	0.08632600	-1.16572300
Au	-1.68941600	-0.29298100	-0.27648800
H	2.55323200	-1.44714000	-1.55516700
C	4.08900900	-0.44328900	-0.41738400
C	4.83630300	0.41284000	-1.24190600
C	4.73680600	-1.14338600	0.60895200
C	6.19955200	0.59463100	-1.01460000
H	4.35022800	0.93595900	-2.06013700
C	6.10196700	-0.95339400	0.83664400
H	4.18632200	-1.84587400	1.22597900
C	6.83470300	-0.08224100	0.03003000
H	6.76508700	1.26397500	-1.65548800
H	6.59021500	-1.49480000	1.64102400
H	7.89593200	0.06242000	0.20727500

Table S54: Optimized geometry for $TS_{7d'}$

Free energy G = -1214.76003199 Hartree/particle.

C	-1.48873500	1.21865700	1.12469800
C	-2.81125200	1.83405300	1.61672400
H	-2.68783200	2.15163300	2.65768100
H	-3.07262800	2.71951600	1.02977100
H	-3.64423700	1.13618200	1.57045900
O	-1.11745900	0.09490700	1.93881800
H	-1.05407600	0.40123600	2.85570700
C	-0.39179400	2.31893600	1.17512600
H	-0.52179900	2.95436400	2.05507900
H	0.58372400	1.82936400	1.25846400
C	-0.46085600	3.13619600	-0.11178400
H	-1.29677900	3.84496900	-0.12071600
H	0.44136200	3.75200200	-0.25692600
C	-0.54442800	2.23385100	-1.29317500

H	-0.85073900	2.68232600	-2.24063200
C	-2.61217500	0.15496300	-0.98698900
C	-1.55760500	0.71474000	-0.32272800
C	-0.25985400	0.85379200	-1.20720600
H	-0.39625500	0.34884200	-2.16475100
P	3.57025400	-0.94233700	0.26520600
C	5.03622400	0.14491400	0.05994800
H	5.13874200	0.42730100	-0.99048000
H	4.90530100	1.05096400	0.65636700
H	5.94233800	-0.37469200	0.38510300
C	3.61024100	-1.45656800	2.02804300
H	3.46049700	-0.58308200	2.66694100
H	2.80700200	-2.17232100	2.21810100
H	4.57252000	-1.91912900	2.26666800
C	3.97274400	-2.46428400	-0.68044300
H	3.17744700	-3.20001700	-0.53980200
H	4.04461100	-2.22475900	-1.74403000
H	4.92219400	-2.88696100	-0.33925800
Au	1.55291200	0.04130300	-0.40633900
H	-2.51121600	0.11974200	-2.07082800
C	-3.81189300	-0.53260300	-0.50712900
C	-3.89575100	-1.17459100	0.74676600
C	-4.90745900	-0.63488400	-1.39016900
C	-5.05280000	-1.85927600	1.11073900
H	-3.04366700	-1.14489200	1.41762600
C	-6.06689200	-1.30797500	-1.01526400
H	-4.84518800	-0.17158500	-2.37115200
C	-6.14381600	-1.92191400	0.23815900
H	-5.10083800	-2.35487500	2.07580200
H	-6.90542600	-1.36345200	-1.70272800
H	-7.04265000	-2.45789400	0.52775600

Optimization studies for IIb (R= H)

Conditions	A	B	C	D	E	F	G
**Functional**<br>**Basis set**<br>**Solvent**	**B3YLP**<br>**6-31G(d, p)**<br>**IEFPCM**	**B3YLP**<br>**6-31G(d, p)**<br>**SMD**	**B3YLP**<br>**6-311++G(d, p)**<br>**IEFPCM**	**M06**<br>**6-31G(d, p)**<br>**IEFPCM**	**M06**<br>**6-31G(d, p)**<br>**SMD**	**M06**<br>**6-311++G(d, p)**<br>**IEFPCM**	**wB97XD**<br>**6-31G(d, p)**<br>**IEFPCM**
	ΔG (Kcal/mol)	ΔG (Kcal/mol)	ΔG (Kcal/mol)	ΔG (Kcal/mol)	ΔG (Kcal/mol)	ΔG (Kcal/mol)	ΔG (Kcal/mol)
**Ib**	0.0	0.0	0.0	0.0	0.0	0.0	0.0
**TS_{1b}**	3.6	6.9	8.3	5.6	7.9	7.3	8.7
**IIb**	-1.1	2.6	5.9	-4.9	-2.2	0.4	-4.1
**TS_{2b}**	7.4	10.7	11.8	10.0	11.2	10.6	12.2
**IIIb**	-3.1	11.8	3.5	-6.1	-5.4	-1.3	-4.9
**TS_{3b}**	2.1	5.5	8.3	1.7	2.4	4.6	3.4
**IVb**	-6.6	-3.8	2.2	-7.4	-5.4	-0.8	-9.2
**TS_{5b}**	-6.6	-4.0	1.8	-6.5	-5.0	-1.4	-8.0
**VIb**	-15.7	-12.3	-10.1	-14.8	-13.5	-12.3	-15.6
**TS_{4b}**	6.7	9.2	15.0	-1.4	0.1	4.1	-1.3
**Vb**	-37.5	-35.5	-30.7	-38.1	-37.3	-36.2	-39.4

Conditions B:

Coordinates and energies for the the cyclization of **Ib** calculated at the B3LYP/6-31G(d,p) (C, H, P, O, N), SDD(Au) level taking into account solvent effect of CH_2Cl_2 (SMD) and employing PMe_3 as the phosphine.

Cartesian Coordinates (in Å).

Table S55: Optimized geometry for Ib

Free energy G = -1062.386034 Hartree/particle.

C	1.84499600	1.86008900	0.29764300
C	1.35578500	3.30880500	0.12557700
H	1.76961000	3.91877500	0.93347500
H	1.67934400	3.72366000	-0.83303200
H	0.26226600	3.35574700	0.17026600
O	1.46944700	1.35322700	1.57697700
H	0.54655700	1.60751500	1.73717200
C	3.39134500	1.79946500	0.21439800
H	3.69057700	2.03607600	-0.81295500
H	3.75148400	2.61824800	0.84714300
C	4.05793000	0.49291600	0.68596500
H	5.13216200	0.71761100	0.76428200
H	3.71980700	0.26730300	1.69964200
C	3.88603500	-0.68963100	-0.23151900
H	4.10274400	-0.48454200	-1.28193600
C	3.55637400	-1.95254200	0.09222300
C	3.25336100	-2.43404200	1.48982600
H	3.23431000	-1.63286600	2.23130400
H	2.28362800	-2.94872500	1.52059900
H	4.00083500	-3.17131900	1.81308000
C	3.48448900	-3.02734900	-0.96654100
H	2.47848700	-3.46660500	-1.01731000
H	3.73991200	-2.64414100	-1.95916400
H	4.16857500	-3.85547300	-0.73624700
C	1.24633600	1.02576000	-0.79124600
C	0.84728600	0.42706600	-1.79014200
H	0.75241800	-0.06444800	-2.74025600
P	-2.99623400	-0.48825800	0.44120600
C	-4.31802300	0.75274800	0.19879700
H	-4.03989500	1.68848200	0.69126400
H	-4.45307100	0.94299100	-0.86934700
H	-5.25774200	0.38571900	0.62337900
C	-3.62382600	-2.03487200	-0.30949000
H	-3.76770000	-1.89033300	-1.38358300
H	-2.90055300	-2.83996400	-0.15503200
H	-4.57812700	-2.31071100	0.15025400
C	-2.92966100	-0.79977400	2.24340700
H	-2.19920000	-1.58539300	2.45360800
H	-2.62715400	0.11255400	2.76457400
H	-3.91481700	-1.11221000	2.60398600
Au	-0.94558300	0.13618100	-0.45572700

Table S56: Optimized geometry for TS_{1b}

Free energy G = -1062.375049 Hartree/particle.

C	2.21404000	1.56167300	0.12030700
C	0.99070000	2.48550500	-0.06042700
H	1.28569600	3.49618000	0.23671600
H	0.65525900	2.50229700	-1.10022700
H	0.15811700	2.15562500	0.56877400
O	2.68634200	1.61346500	1.46674900
H	1.96769500	1.32159000	2.04984500
C	3.37825600	2.02475200	-0.76593400
H	3.02442100	2.07781100	-1.80073100

H	3.67713300	3.03129400	-0.45836900
C	4.54364300	1.02883800	-0.64796300
H	5.28660400	1.24407700	-1.42459900
H	5.03658500	1.14685100	0.31819000
C	4.01834600	-0.36599200	-0.83188800
H	3.73618000	-0.63104100	-1.85021400
C	4.02346500	-1.37732600	0.09046600
C	4.49679800	-1.23953500	1.50954100
H	4.60898900	-0.20518600	1.83193700
H	3.80119100	-1.74266500	2.19086400
H	5.46233700	-1.75138500	1.61984200
C	3.57890600	-2.75351000	-0.30630400
H	2.74499800	-3.09172600	0.32289300
H	3.28491400	-2.81407300	-1.35683400
H	4.39505100	-3.46787200	-0.13601500
C	1.82452300	0.15979900	-0.23763900
C	0.93684300	-0.74454800	-0.40936900
H	1.10732500	-1.78394600	-0.67321100
P	-3.40989700	0.03632400	0.13500200
C	-4.19798300	0.86557300	-1.29778600
H	-3.73486000	1.84340500	-1.45568600
H	-4.05432800	0.26074200	-2.19726400
H	-5.26955500	0.99786500	-1.11753700
C	-4.36214700	-1.51181100	0.37429000
H	-4.22033600	-2.16745800	-0.48899100
H	-4.00277300	-2.02777600	1.26887300
H	-5.42720200	-1.28694400	0.48872000
C	-3.83595700	1.08325700	1.57884800
H	-3.47116300	0.61231200	2.49579000
H	-3.36231300	2.06324000	1.47420200
H	-4.92093000	1.21260500	1.64612300
Au	-1.11098600	-0.30061300	-0.13808800

Table S57: Optimized geometry for IIb

Free energy G = -1062.381872 Hartree/particle.

C	4.38403000	0.89607700	-0.81318200
C	3.56667800	-0.39622800	-0.87488200
C	2.16757800	1.42879100	0.16826600
C	3.66978800	1.82750200	0.18149200
H	5.42438000	0.70113100	-0.53575400
H	4.39035300	1.33139700	-1.81654000
H	3.80401100	2.87889800	-0.08723000
H	4.06407500	1.70333800	1.19187600
C	1.36118200	2.39362200	-0.71421100
H	0.31486200	2.08073200	-0.77531100
H	1.40605900	3.40191100	-0.28499400
H	1.77175700	2.44280000	-1.72797200
C	2.10505900	0.00457600	-0.42189000
C	0.98783100	-0.73799400	-0.63324100
H	1.16637900	-1.72321600	-1.06403800
P	-3.26897900	0.09694700	0.21020800
C	-3.79932700	1.82689900	-0.10163600
H	-4.86371600	1.95580000	0.11859400
H	-3.21535900	2.50541100	0.52685600
H	-3.61628800	2.08041500	-1.14982400
C	-4.41894700	-0.92742800	-0.78922000
H	-4.24892900	-0.73399200	-1.85218900
H	-4.22871100	-1.98645700	-0.59311100
H	-5.45968600	-0.69513400	-0.54204000

C	-3.74765900	-0.23053700	1.95190500
H	-3.53986700	-1.27619900	2.19610200
H	-3.15701900	0.40519900	2.61763500
H	-4.81197100	-0.02834100	2.10841700
Au	-0.98032700	-0.30122800	-0.23678000
O	1.60921800	1.39401700	1.49084300
H	1.58863900	2.30613200	1.82097300
C	3.49583200	-1.29563400	0.26087600
C	3.23891100	-2.74636400	0.01736600
H	4.22075700	-3.22676800	0.15715900
H	2.56752100	-3.18529400	0.75984600
H	2.90429800	-2.97369100	-0.99505400
C	3.90236800	-0.93306600	1.65872800
H	3.28232200	-0.14086800	2.08341800
H	3.83829900	-1.80598000	2.31074100
H	4.93947100	-0.57369300	1.64805600
H	3.58517800	-0.91412300	-1.83211300

Table S58: Optimized geometry for TS_{2b}

Free energy G = -1062.368997 Hartree/particle.

C	1.65843100	1.87737000	0.04555200
C	0.75468200	2.93777800	-0.58968600
H	1.14520000	3.93576800	-0.36725400
H	0.70266600	2.81558500	-1.67600200
H	-0.26335600	2.86022800	-0.19064900
O	1.70213000	2.05009900	1.46598000
H	0.78737900	2.10672500	1.78436400
C	3.10179700	2.02482000	-0.45979700
H	3.08981400	2.02319800	-1.55572500
H	3.44398000	3.01444500	-0.14026100
C	4.08902600	0.96481200	0.05728600
H	5.10368800	1.31301400	-0.17900200
H	4.02490500	0.90889800	1.14658100
C	3.92213700	-0.39122400	-0.57929100
H	3.92931600	-0.39128000	-1.67008700
C	4.01668600	-1.60824100	0.03408200
C	4.12054500	-1.80399000	1.51916700
H	3.98142000	-0.88484700	2.08925900
H	3.38535800	-2.54316900	1.86023500
H	5.10814200	-2.21419900	1.76883700
C	4.00326200	-2.87037600	-0.78105900
H	3.14185500	-3.49810700	-0.51201500
H	3.97155900	-2.67218600	-1.85555000
H	4.89565100	-3.47060200	-0.56340400
C	1.13758700	0.47194200	-0.24364000
C	1.63716900	-0.67495000	-0.45884100
H	1.54020500	-1.71807800	-0.69434500
P	-3.24559700	-0.42133400	0.11063000
C	-3.88893100	-0.05803700	1.78757900
H	-3.38308700	-0.69181900	2.52105100
H	-3.69602900	0.98933300	2.03624400
H	-4.96647000	-0.24577500	1.82832100
C	-4.27752000	0.58229100	-1.02366200
H	-4.09570900	1.64517200	-0.84238300
H	-4.01415900	0.35195100	-2.05968000
H	-5.33792100	0.36368300	-0.86223800
C	-3.72069000	-2.16025800	-0.22175500
H	-3.44942300	-2.42336300	-1.24786300
H	-3.18789300	-2.82434300	0.46428200
H	-4.79909000	-2.29114200	-0.08717700

Au	-0.95336900	-0.02900600	-0.11263900

Table S59: Optimized geometry for IIIb

Free energy G = -1062.367260 Hartree/particle.

P	3.21481700	0.29810200	0.06298100
C	4.02434100	0.69923800	-1.53805700
H	5.09912500	0.84459600	-1.39553900
H	3.86099700	-0.11669500	-2.24611400
H	3.58734900	1.61244100	-1.94915900
C	3.69514000	1.67596000	1.18067600
H	3.24395100	2.60623300	0.82724300
H	3.32960900	1.47145700	2.18988700
H	4.78297800	1.78835700	1.20526800
C	4.13271100	-1.16207400	0.69708200
H	3.77011700	-1.41438000	1.69657800
H	3.95937800	-2.01525100	0.03670200
H	5.20522500	-0.95273000	0.74359700
Au	0.87552700	-0.03709500	-0.09470500
C	-1.93351000	0.77369200	-0.63730800
C	-1.84338900	-1.62766000	0.15574900
C	-3.47976300	0.65303300	-0.93212900
H	-1.42594800	1.62906600	-1.06564700
C	-3.03811500	-1.85346200	-0.80074300
C	-4.07929600	-0.73725100	-0.71316200
H	-3.68711600	1.09517400	-1.90736200
H	-3.50569700	-2.81663800	-0.56471900
H	-2.65334900	-1.93327600	-1.82415200
H	-4.58037700	-0.78469200	0.25595900
H	-4.84953900	-0.89280600	-1.47415000
C	-1.17535600	-0.28269300	-0.18142400
C	-0.86019100	-2.79721500	0.04293000
H	-0.02845700	-2.66678500	0.74060600
H	-1.37113300	-3.73896300	0.27556300
H	-0.45564900	-2.87705900	-0.97043500
O	-2.28556500	-1.50732500	1.52041900
H	-2.61797000	-2.37285900	1.80119500
C	-3.38739700	1.61313000	0.14775300
C	-3.30606600	3.07705200	-0.18653400
H	-4.29104300	3.50941500	0.03363700
H	-2.57846900	3.59631800	0.44284600
H	-3.08316800	3.26097400	-1.23903200
C	-3.66773000	1.29574700	1.58541200
H	-3.10998500	1.96369600	2.24636100
H	-4.73682300	1.49578500	1.74638000
H	-3.45667400	0.26125500	1.84667000

Table S60: Optimized geometry for TS_{3b}

Free energy G = -1062.377282 Hartree/particle.

C	4.01673500	-1.17386900	0.20497600
C	3.43613400	-0.44313800	-0.92017400
H	3.27282800	-1.15395500	-1.73497900
C	5.16687000	-0.68746000	0.98887600
H	5.19360500	-1.11484100	1.99371800
H	6.04761500	-1.08558000	0.45131200
H	5.26218800	0.39498400	1.01874700
C	3.52054300	-2.53039400	0.51974900
H	4.37642600	-3.22052800	0.48984700
H	3.16864300	-2.55661400	1.55907900

H	2.73915000	-2.88038500	-0.15306800
O	2.83052100	1.01516100	1.56376500
C	2.25607800	1.34726000	0.27696700
C	4.09990800	0.87769600	-1.34335400
H	5.18295700	0.86610200	-1.19984200
H	3.92171800	1.01164600	-2.41345800
C	3.39659800	2.00161300	-0.53505200
H	2.98282100	2.75496900	-1.21005400
H	4.07354000	2.51508400	0.15335600
H	2.12174100	0.61961200	2.09726100
C	1.98432800	0.01573900	-0.42548300
C	0.86307400	-0.67781400	-0.64363700
H	1.01242300	-1.64772900	-1.12556800
Au	-1.11801200	-0.26724900	-0.22872300
C	-3.84972600	1.68824400	0.92170800
H	-4.92711700	1.75781800	1.10216500
H	-3.31677100	1.82682400	1.86675500
H	-3.54825100	2.48253000	0.23289400
C	-4.13419000	-1.15764000	1.38734100
H	-5.19873900	-0.95763000	1.54552500
H	-4.01283400	-2.17089300	0.99389200
H	-3.60915600	-1.09131700	2.34437900
C	-4.48079700	-0.08580500	-1.28489800
H	-4.35782100	-1.07608000	-1.73275000
H	-5.53404200	0.06557400	-1.02766200
H	-4.17811200	0.66563400	-2.01964700
P	-3.41834000	0.05253900	0.20631500
C	1.05759800	2.27083400	0.46636600
H	0.61232700	2.54263400	-0.49451900
H	0.28230800	1.79764400	1.07799500
H	1.38751000	3.18616900	0.96855700

Table S61: Optimized geometry for IVb

Free energy G = -1062.392071 Hartree/particle.

C	4.06585200	-0.68745700	0.40540900
C	3.35527300	-0.43518700	-0.94906100
H	3.47234000	-1.29564200	-1.60938400
C	5.54605900	-0.37345500	0.50072300
H	5.91705200	-0.57886300	1.50871300
H	6.08593500	-1.01995300	-0.19876700
H	5.76937900	0.66470300	0.25262000
C	3.72025200	-2.02727400	1.03184100
H	4.27248400	-2.80893200	0.50088200
H	4.03114500	-2.06123200	2.08152700
H	2.65355300	-2.25237900	0.95995600
O	3.35330100	0.43221300	1.26592400
C	2.23026500	1.09533700	0.30805400
C	3.77833200	0.92327800	-1.56117600
H	4.85898800	1.05407500	-1.64223200
H	3.35991900	1.00374700	-2.56740500
C	3.11181700	1.95639000	-0.60181900
H	2.46836900	2.66237500	-1.13424300
H	3.82910700	2.53680200	-0.01514200
H	2.90085200	0.01212700	2.02717600
C	1.92165100	-0.12449100	-0.51954000
C	0.76176200	-0.72810600	-0.81774600
H	0.88754500	-1.59519100	-1.47528700
Au	-1.17975000	-0.28783400	-0.29341900
C	-4.17773700	1.59710000	-0.55690400
H	-5.22386300	1.72397200	-0.26051300

H	-3.61415900	2.49205100	-0.27786500
H	-4.12366500	1.47712500	-1.64260800
C	-3.74816700	0.40072700	2.05248400
H	-4.81087700	0.58250700	2.24262900
H	-3.42618400	-0.47878500	2.61744800
H	-3.17022600	1.26457800	2.39318200
C	-4.58169900	-1.24941400	-0.18174500
H	-4.27423000	-2.16118600	0.33822800
H	-5.61180600	-1.00486100	0.09603300
H	-4.53371400	-1.43192700	-1.25909300
P	-3.44528500	0.12363200	0.26116600
C	1.24580200	1.77885800	1.21120700
H	0.47109600	2.23493500	0.58826600
H	0.75583800	1.07739100	1.89293200
H	1.73144100	2.57143500	1.78781800

Table S62: Optimized geometry for TS_{5b}

Free energy G = -1062.392414 Hartree/particle.

C	4.04556000	-0.72590500	0.38860900
C	3.35840400	-0.31525500	-0.95332700
H	3.46424400	-1.10827300	-1.69600200
C	5.56285000	-0.57572400	0.42041100
H	5.95844600	-0.93094200	1.37678800
H	6.01177100	-1.17565500	-0.37764100
H	5.87475200	0.46238100	0.29022400
C	3.62308400	-2.11646600	0.85103900
H	2.53752100	-2.24428500	0.82632300
H	4.07179100	-2.87225600	0.19950100
H	3.97857300	-2.30478300	1.87010900
O	3.49645700	0.29552500	1.34542400
C	2.12237100	1.20159400	0.25761100
C	3.83258900	1.07491800	-1.44373300
H	4.91682000	1.19189000	-1.45539500
H	3.47366700	1.23263500	-2.46421300
C	3.13342400	2.05931000	-0.46439100
H	2.57999900	2.85145300	-0.98447400
H	3.81055500	2.55302800	0.23710900
H	3.03643900	-0.16242900	2.07282200
C	1.92204200	-0.01236500	-0.53523700
C	0.77206000	-0.67071400	-0.80599600
H	0.92147300	-1.53189800	-1.46582300
Au	-1.17627700	-0.27637300	-0.28561000
C	-3.86233600	1.83854200	0.66736000
H	-4.92804100	1.94205900	0.89502500
H	-3.27776100	2.15284500	1.53673900
H	-3.61106700	2.48937300	-0.17506100
C	-4.06310500	-0.87170500	1.68131800
H	-5.12149500	-0.66342700	1.86765100
H	-3.93545200	-1.93963100	1.48294800
H	-3.48345600	-0.61275000	2.57199400
C	-4.61121600	-0.32666800	-1.11590200
H	-4.50427900	-1.38514800	-1.36964500
H	-5.64658300	-0.12887900	-0.82106000
H	-4.36889100	0.26808200	-2.00121600
P	-3.45429900	0.09760500	0.24560400
C	1.19346100	1.78298700	1.25794600
H	0.47188900	2.40307700	0.70993400
H	0.63619700	1.02116500	1.80641000
H	1.72646200	2.44229500	1.94849000

Table S63: Optimized geometry for VIb

Free energy G = -1062.405632 Hartree/particle.

C	4.11309000	1.31943600	-0.88110200
C	3.38925100	-0.04770100	-0.76467200
C	1.96562800	1.66909400	0.14634800
C	3.33000800	2.25522300	0.05602000
H	5.17261100	1.27748900	-0.62730300
H	4.03303600	1.68455400	-1.91013800
H	3.30317600	3.30949800	-0.24255700
H	3.74082400	2.23230500	1.07793700
C	0.85572600	2.45496000	0.72151400
H	-0.01780400	1.85577500	0.98193900
H	1.21464400	3.00950900	1.59626400
H	0.55294300	3.21599300	-0.01282100
C	1.95558100	0.36878100	-0.39747600
C	0.81651300	-0.38084800	-0.65937600
H	1.04953300	-1.28394600	-1.23311600
P	-3.49655700	-0.08834500	0.20832300
C	-4.19315300	1.60864500	0.26297700
H	-5.26659900	1.57702900	0.47477100
H	-3.68961600	2.18711700	1.04266200
H	-4.03156200	2.10301400	-0.69915100
C	-4.52560000	-0.97521700	-1.02479700
H	-4.37542400	-0.53320600	-2.01385100
H	-4.22640900	-2.02632300	-1.06316000
H	-5.58517300	-0.91094300	-0.75788900
C	-3.95083600	-0.83536800	1.82115000
H	-3.63597900	-1.88249000	1.83929500
H	-3.44014800	-0.30339300	2.62874100
H	-5.03239200	-0.78199600	1.98009300
Au	-1.16943300	-0.17722300	-0.24714000
O	4.05653800	-0.26021100	1.52116700
H	4.43460300	-0.83579300	2.20320300
C	4.02117100	-1.00777100	0.29110400
C	3.18025100	-2.27673300	0.49263200
H	3.07883300	-2.84780500	-0.43623800
H	3.66758700	-2.92716500	1.22830700
H	2.18083900	-2.04235200	0.87152300
C	5.44683400	-1.40739100	-0.11970600
H	6.10377300	-0.53838100	-0.20632000
H	5.87626300	-2.07704000	0.63453300
H	5.44990900	-1.94029900	-1.07643500
H	3.39701100	-0.58430400	-1.71811300

Table S64: Optimized geometry for TS_{4b}

Free energy G = -1062.384233 Hartree/particle.

C	4.39508200	0.94371800	-0.67357400
C	3.59436500	-0.35288800	-0.83420000
C	2.12361700	1.44401300	0.16298300
C	3.61035400	1.84104000	0.30001000
H	5.41582700	0.74595900	-0.33312400
H	4.46554200	1.41155400	-1.65966700
H	3.75984700	2.90055800	0.07806800
H	3.92755200	1.68523800	1.33269400
C	1.38264000	2.40964900	-0.77662900
H	0.34126600	2.09944600	-0.91294900
H	1.39843300	3.41334000	-0.33985500
H	1.85830500	2.45230200	-1.76214100

C	2.10960400	0.02368900	-0.45430800
C	1.00596900	-0.73340500	-0.67222400
H	1.18068000	-1.71874000	-1.10316800
P	-3.24976900	0.08857800	0.22689300
C	-3.58752200	1.65676300	1.11919600
H	-4.66012100	1.77250100	1.30478900
H	-3.05455100	1.65812500	2.07425700
H	-3.23246400	2.50145200	0.52189200
C	-4.30595200	0.16897000	-1.27201900
H	-3.95917200	0.97568200	-1.92390700
H	-4.23414400	-0.77546900	-1.81894600
H	-5.35019200	0.35029900	-0.99841600
C	-4.01978500	-1.21518400	1.26521600
H	-3.94589400	-2.17901700	0.75371900
H	-3.48880100	-1.28549800	2.21881700
H	-5.07326600	-0.98601200	1.45436900
Au	-0.96123100	-0.29625600	-0.25026200
O	1.52962600	1.47746000	1.46490300
H	0.61628300	1.15626300	1.36848300
C	3.49030700	-1.30189000	0.26084800
C	3.26602900	-2.74222600	-0.05125700
H	4.25334600	-3.21007700	0.09730000
H	2.58418400	-3.22465800	0.65354900
H	2.96637000	-2.93188500	-1.08216400
C	3.81381900	-0.98110600	1.68872500
H	4.83801600	-0.59042600	1.74441100
H	3.14975700	-0.22084600	2.10554600
H	3.74185200	-1.87809100	2.30606000
H	3.67697700	-0.83300200	-1.80817600

Table S65: Optimized geometry for Vb

Free energy G = -1062.442582 Hartree/particle.

C	-4.05974500	-0.81112000	1.54244500
C	-2.70902900	-1.25247600	-0.46743800
C	-3.45880100	-1.95349300	0.69534200
H	-4.06492300	-1.02752800	2.61713800
H	-5.10362900	-0.60347200	1.26585800
H	-4.20602500	-2.67028100	0.34241700
H	-2.71549800	-2.50018100	1.28484300
C	-3.56597800	-1.15804000	-1.73851700
H	-3.06449600	-0.57331700	-2.51621200
H	-3.76000000	-2.16085300	-2.13848400
H	-4.53285400	-0.69096900	-1.52543700
C	-2.41019000	0.13341100	0.11795800
P	2.70259900	-0.81003200	0.21758600
C	2.78600300	-2.40543400	-0.67821600
H	3.73062300	-2.91188700	-0.45627200
H	1.95180100	-3.04275700	-0.37291400
H	2.71569600	-2.22586600	-1.75445400
C	4.17906200	0.13083600	-0.31983200
H	4.10179200	0.35985000	-1.38604200
H	4.23876700	1.06862200	0.23917900
H	5.08506300	-0.45650200	-0.13948100
C	3.00190100	-1.22099900	1.97717100
H	3.05546700	-0.29998600	2.56388300
H	2.17973300	-1.83335600	2.35699100
H	3.94245900	-1.77182200	2.07860700
Au	0.69650100	0.31534400	-0.12909000
O	-1.46082600	-1.89417700	-0.77766100
H	-1.65603500	-2.69079700	-1.29466800

C	-3.18569400	0.36510300	1.19547200
H	-3.24452500	1.30335800	1.73539600
C	-0.85491400	2.18633800	-0.11776100
C	-0.92883200	2.67512200	1.30689100
H	-1.77698300	3.36611900	1.40939600
H	-1.06738900	1.86753400	2.02784700
H	-0.02638700	3.23631500	1.56700000
C	-0.27066800	3.17196000	-1.10280600
H	-0.18881700	2.75585700	-2.11006300
H	-0.92782900	4.05162500	-1.14955400
H	0.71196500	3.53025300	-0.78040600
C	-1.48951900	1.04172900	-0.58474500
H	-1.45548700	0.89416700	-1.66476900

Conditions C:

Coordinates and energies for the the cyclization of **Ib** calculated at the B3LYP/6-311++G(d,p) (C, H, P, O, N), SDD(Au) level taking into account solvent effect of CH_2Cl_2 (IEF-PCM) and employing PMe_3 as the phosphine. Cartesian Coordinates (in Å).

Table S66: Optimized geometry for Ib

Free energy G = -1062.537471 Hartree/particle.

C	1.68987600	1.80928200	0.07527100
C	0.85366400	3.00390300	-0.41266500
H	1.19075000	3.90427100	0.10375100
H	0.97003700	3.14648200	-1.48869100
H	-0.20732200	2.85044600	-0.19985700
O	1.56873200	1.64671500	1.49177700
H	0.63481200	1.67925600	1.73341700
C	3.19072800	2.06358800	-0.21885400
H	3.32322700	2.12167000	-1.30330500
H	3.38883800	3.06287500	0.17901500
C	4.20377000	1.08103000	0.39766300
H	5.17641100	1.58872600	0.35539000
H	3.97795500	0.95070000	1.45621800
C	4.33285200	-0.23784800	-0.31610200
H	4.49496100	-0.15317300	-1.39104000
C	4.31406200	-1.47258900	0.20421600
C	4.11146800	-1.79067100	1.66405500
H	3.91613500	-0.91083100	2.27615100
H	3.27290900	-2.48544600	1.78997400
H	4.99510700	-2.29606800	2.07109300
C	4.50422300	-2.68353500	-0.67643000
H	3.63519200	-3.35018900	-0.61729400
H	4.65548900	-2.40777200	-1.72216400
H	5.36897800	-3.27329600	-0.35023900
C	1.24287000	0.57022200	-0.61720900
C	0.98762500	-0.43519800	-1.26715400
H	1.05239900	-1.30379600	-1.88742200
P	-3.26471900	-0.32124600	0.35281000
C	-3.53912500	0.35738900	2.02879000
H	-2.91834900	-0.17946300	2.74669600
H	-3.26844800	1.41372700	2.04506300
H	-4.59030000	0.24904600	2.30459000
C	-4.43059500	0.56386400	-0.74353300
H	-4.17187600	1.62284400	-0.77662500
H	-4.36671500	0.15276900	-1.75166600
H	-5.45059900	0.45060800	-0.36950800
C	-3.85164200	-2.05233400	0.40966500
H	-3.77859700	-2.49425900	-0.58455200
H	-3.23210700	-2.62661600	1.09911600

H	-4.89106500	-2.08068300	0.74429700
Au	-1.04323500	-0.16012000	-0.33993700

Table S67: Optimized geometry for TS_{1b}

Free energy G = -1062.524274 Hartree/particle.

C	2.24624900	1.56528000	0.12380900
C	1.02778100	2.49163000	-0.04976400
H	1.33984600	3.50740300	0.20140600
H	0.66055400	2.47278200	-1.07642200
H	0.21552300	2.19537700	0.61652200
O	2.72790100	1.62258400	1.47486700
H	2.02965000	1.31736100	2.06745500
C	3.40676000	2.02231100	-0.76495600
H	3.04359500	2.10264000	-1.79300200
H	3.73683500	3.01289600	-0.44567600
C	4.53787200	0.98774300	-0.67580100
H	5.26550600	1.16232200	-1.47347600
H	5.06196700	1.09160100	0.27286000
C	3.94651300	-0.38430400	-0.83246600
H	3.65373100	-0.65455300	-1.84401800
C	3.97224500	-1.39702900	0.09151400
C	4.50259000	-1.26558800	1.48677000
H	4.67678600	-0.23686000	1.78958300
H	3.81539700	-1.73097800	2.19892800
H	5.44557500	-1.82084100	1.55799500
C	3.50297000	-2.76533000	-0.29166200
H	2.72398400	-3.11964600	0.39210700
H	3.13931900	-2.81446300	-1.31803600
H	4.33684400	-3.46993600	-0.19307500
C	1.87176400	0.15592800	-0.22987900
C	0.94371300	-0.71022800	-0.39549100
H	1.10688400	-1.74828700	-0.66148300
P	-3.41667800	0.03945600	0.13580000
C	-3.83361900	1.38411800	1.30934400
H	-3.41280800	1.15685600	2.28965900
H	-3.40830100	2.32366700	0.95407600
H	-4.91761700	1.48770900	1.39596400
C	-4.27987000	0.48315100	-1.41893500
H	-3.85981900	1.40776900	-1.81706000
H	-4.13959400	-0.31038700	-2.15418400
H	-5.34743300	0.62017200	-1.23246400
C	-4.30040500	-1.43565900	0.76773500
H	-4.16229900	-2.26712400	0.07549900
H	-3.89234000	-1.71715300	1.73942800
H	-5.36674900	-1.22188700	0.86998900
Au	-1.10867600	-0.29254500	-0.13885800

Table S68: Optimized geometry for IIb

Free energy G = -1062.528093 Hartree/particle.

C	4.37028500	0.90259100	-0.85346600
C	3.56068600	-0.39436700	-0.88497800
C	2.17941100	1.43605600	0.17327200
C	3.68006000	1.83272800	0.15649500
H	5.41858100	0.71765000	-0.60852200
H	4.34462300	1.33183500	-1.85713300
H	3.80911800	2.88250700	-0.11175000
H	4.09538900	1.70752300	1.15588400

C	1.36034300	2.38794200	-0.70987100
H	0.31660500	2.07272900	-0.75354300
H	1.40967500	3.39969700	-0.29528200
H	1.75942100	2.42039700	-1.72675400
C	2.10308000	0.00605300	-0.39854000
C	0.98923600	-0.73645500	-0.59437500
H	1.16372200	-1.72618900	-1.01127700
P	-3.28558800	0.09801800	0.19599200
C	-3.77597100	1.85439900	-0.00791100
H	-4.84269100	1.98094900	0.19060200
H	-3.20348000	2.47437100	0.68375000
H	-3.55804400	2.17688600	-1.02716200
C	-4.42452700	-0.83026000	-0.90261000
H	-4.22561000	-0.56526600	-1.94211800
H	-4.25763700	-1.90113500	-0.77817700
H	-5.46375700	-0.59533400	-0.66127700
C	-3.82306200	-0.33921800	1.89435600
H	-3.64666800	-1.40153300	2.06956300
H	-3.24296400	0.23459700	2.61837200
H	-4.88536400	-0.12191000	2.02673300
Au	-0.98716300	-0.30804000	-0.21968200
O	1.64399500	1.42018400	1.50877800
H	1.57900100	2.33178900	1.81888400
C	3.52853500	-1.29394900	0.24732200
C	3.26971200	-2.74312600	0.00679400
H	4.25584200	-3.22195200	0.10372000
H	2.62930000	-3.18697400	0.77011600
H	2.89691700	-2.96636400	-0.99056500
C	3.96858200	-0.93387600	1.63347700
H	3.39372800	-0.11104100	2.05583100
H	3.87873600	-1.79313200	2.29675000
H	5.01948200	-0.62259100	1.60181000
H	3.55555800	-0.90524800	-1.84343400

Table S69: Optimized geometry for TS_{2b}

Free energy G = -1062.518591 Hartree/particle.

C	1.66275800	1.85952000	0.04634900
C	0.70998500	2.90495900	-0.53869700
H	1.11998300	3.90467600	-0.37831300
H	0.57142900	2.74868800	-1.61116600
H	-0.27049600	2.84461900	-0.05800200
O	1.81389400	2.05965600	1.46168600
H	0.94263700	2.01663200	1.87487100
C	3.06170100	2.01371900	-0.56390700
H	2.96909800	1.98999000	-1.65383700
H	3.41499600	3.01184300	-0.29251200
C	4.09460800	0.97759700	-0.09895600
H	5.07890300	1.30791600	-0.45173500
H	4.14178000	0.97452500	0.99009500
C	3.86970600	-0.40689100	-0.64777300
H	3.80510500	-0.46712100	-1.73297000
C	4.03989100	-1.58593400	0.01994400
C	4.27832700	-1.69353300	1.49662500
H	4.08609200	-0.76785700	2.03539500
H	3.66120000	-2.48758200	1.92741200
H	5.32196100	-1.98000900	1.67426800
C	3.99155000	-2.88902000	-0.72220500
H	3.18092500	-3.52422100	-0.34294100
H	3.86392300	-2.75313100	-1.79690800
H	4.91713900	-3.44888100	-0.55040800

C	1.13668500	0.44627800	-0.18227100
C	1.65593000	-0.68945200	-0.39678100
H	1.54128800	-1.73355100	-0.60399800
P	-3.26983800	-0.41244300	0.08438700
C	-4.12799600	0.84311000	1.10554100
H	-3.71520700	0.83715500	2.11525000
H	-3.97721200	1.83173500	0.66984200
H	-5.19788900	0.62729400	1.15055500
C	-4.12815000	-0.38580600	-1.53358100
H	-3.97657000	0.58390700	-2.00926600
H	-3.71513800	-1.16192200	-2.17915300
H	-5.19783300	-0.55954900	-1.39660700
C	-3.70033900	-2.02648700	0.83576400
H	-3.27449000	-2.83209300	0.23633300
H	-3.28537600	-2.08109100	1.84301000
H	-4.78525500	-2.14400200	0.88307900
Au	-0.96142400	-0.04097400	-0.07875800

Table S70: Optimized geometry for IIIb

Free energy G = -1062.531826 Hartree/particle.

P	3.23712000	0.31455900	0.06390800
C	4.04705900	0.71708100	-1.53276900
H	5.12099800	0.85733200	-1.38980200
H	3.87995100	-0.09456900	-2.24228800
H	3.61405500	1.63180200	-1.94021000
C	3.71997800	1.68579100	1.18323500
H	3.27069800	2.61631300	0.83325000
H	3.35454400	1.47926600	2.19022600
H	4.80661800	1.79574700	1.20740800
C	4.15151900	-1.14598200	0.69307800
H	3.78815300	-1.40056400	1.68987000
H	3.97752600	-1.99559900	0.03112600
H	5.22276800	-0.93793800	0.74060000
Au	0.89657000	-0.02295000	-0.08888000
C	-1.91600700	0.75686800	-0.61779600
C	-1.82159300	-1.63614300	0.14988800
C	-3.46298200	0.63420300	-0.93026300
H	-1.44151300	1.66121500	-0.97117500
C	-2.98226200	-1.87114900	-0.84165000
C	-4.04221900	-0.77385900	-0.76661800
H	-3.63839500	1.07632400	-1.91057200
H	-3.44224000	-2.84170300	-0.63045800
H	-2.56558100	-1.93108900	-1.85215000
H	-4.57693600	-0.85416000	0.17984700
H	-4.78132500	-0.92200500	-1.55723000
C	-1.16296900	-0.28545200	-0.16865200
C	-0.82797600	-2.79643400	0.07515800
H	-0.01829000	-2.65295100	0.79336700
H	-1.33664200	-3.74017100	0.29901900
H	-0.39467200	-2.87694800	-0.92424500
O	-2.32089400	-1.52633100	1.50366300
H	-2.54477800	-2.41218500	1.81538300
C	-3.53142800	1.59424300	0.14684600
C	-3.46057200	3.05063900	-0.17537300
H	-4.47172500	3.45052200	-0.02090000
H	-2.80182700	3.58807200	0.50952400
H	-3.17429900	3.24726200	-1.20784800
C	-3.84207700	1.24289000	1.55983800
H	-3.36610800	1.93778500	2.25306200
H	-4.92915900	1.36760200	1.67312600

H	-3.58134600	0.21981000	1.81510500

Table S71: Optimized geometry for TS_{3b}

Free energy G = -1062.524298 Hartree/particle.

C	4.08810500	-1.11103600	0.19804500
C	3.41904400	-0.44759900	-0.91797400
H	3.24847600	-1.19414300	-1.69698400
C	5.29898300	-0.58254600	0.85038600
H	5.43337900	-0.97363500	1.85837900
H	6.13364700	-0.97192200	0.24052700
H	5.36896500	0.50050300	0.83839900
C	3.63527100	-2.45321500	0.61640000
H	4.46648900	-3.15054200	0.43911800
H	3.47098700	-2.47233100	1.69849800
H	2.75038300	-2.80149600	0.09108700
O	2.90694000	0.98568300	1.54628600
C	2.24838900	1.31957800	0.29055000
C	4.00713200	0.87877300	-1.42308100
H	5.09587200	0.90974100	-1.37310700
H	3.73690700	0.98289300	-2.47496400
C	3.33100800	1.99409300	-0.58101200
H	2.86714100	2.73601600	-1.23208700
H	4.03513700	2.52277700	0.06424000
H	2.24897200	0.57475800	2.12297500
C	1.97034400	-0.01464500	-0.39690200
C	0.85477300	-0.70666700	-0.61213100
H	0.99516300	-1.67167900	-1.10236000
Au	-1.13122800	-0.28977700	-0.21159000
C	-3.84683700	1.73736200	0.83596500
H	-4.92305400	1.83256100	0.99718600
H	-3.32472800	1.90037100	1.78002200
H	-3.52053200	2.49530200	0.12221800
C	-4.17736800	-1.08588600	1.40957900
H	-5.24081500	-0.87372500	1.54273400
H	-4.05462400	-2.11215700	1.06032100
H	-3.66529300	-0.98054400	2.36718200
C	-4.48589800	-0.11188800	-1.30605000
H	-4.37290900	-1.11932800	-1.70941500
H	-5.53621100	0.06528200	-1.06332500
H	-4.16415400	0.60410400	-2.06381500
P	-3.43740900	0.06873300	0.18949100
C	1.05039200	2.22024500	0.55672400
H	0.53193000	2.45929200	-0.37313000
H	0.33494700	1.74430700	1.23127000
H	1.39651800	3.15013200	1.01502700

Table S72: Optimized geometry for IVb

Free energy G = -1062.534013 Hartree/particle.

C	4.08150500	-0.68759000	0.39238700
C	3.35356700	-0.42404500	-0.95005500
H	3.46323500	-1.27592600	-1.61938200
C	5.56482500	-0.38302700	0.46247300
H	5.95468500	-0.60034900	1.45842400
H	6.08722900	-1.02208300	-0.25435500
H	5.78542500	0.65521100	0.22146000
C	3.73934300	-2.03478900	1.00431700
H	4.26937400	-2.80995400	0.44549700
H	4.07734000	-2.09324100	2.04268600

H	2.67079400	-2.24680000	0.95389100
O	3.38634200	0.42612700	1.26853200
C	2.22630600	1.09747300	0.31587100
C	3.76325800	0.93858800	-1.56031000
H	4.83942800	1.07852300	-1.65451300
H	3.33458900	1.01821500	-2.56004400
C	3.09928800	1.96421900	-0.59229300
H	2.45204800	2.66897000	-1.11800600
H	3.81620200	2.54566600	-0.00988700
H	2.96630700	0.01985100	2.04568100
C	1.92431100	-0.12129800	-0.50462700
C	0.76645100	-0.71859800	-0.80412100
H	0.88534200	-1.57781300	-1.46934400
Au	-1.17994900	-0.28525600	-0.28007400
C	-3.75447100	0.73397900	1.95341400
H	-4.82322900	0.87733500	2.12845900
H	-3.36474400	0.01268200	2.67335700
H	-3.23559300	1.68316800	2.09536200
C	-4.52372200	-1.36976700	0.11554000
H	-5.55872400	-1.12633900	0.36684900
H	-4.48081700	-1.75820400	-0.90311700
H	-4.15827200	-2.14008400	0.79655800
C	-4.27127600	1.36265800	-0.82953700
H	-4.21903500	1.03272000	-1.86825700
H	-5.31753700	1.49498800	-0.54425800
H	-3.74918600	2.31674800	-0.74137900
P	-3.45754600	0.11913800	0.24884200
C	1.25085700	1.76684700	1.23383900
H	0.47529000	2.22886200	0.61899600
H	0.75919700	1.05771300	1.90239400
H	1.73758500	2.55135700	1.81650800

Table S73: Optimized geometry for TS_{5b}

Free energy G = -1062.534632 Hartree/particle.

C	4.04496900	-0.73794900	0.39132600
C	3.36125300	-0.34178600	-0.95194700
H	3.46464600	-1.13972200	-1.68582300
C	5.55624700	-0.56987600	0.44598200
H	5.94394800	-0.90964400	1.40844600
H	6.01598300	-1.17570700	-0.33926600
H	5.85848400	0.46684700	0.30317800
C	3.61740100	-2.11475900	0.88225500
H	2.53559200	-2.24595200	0.83349700
H	4.08538500	-2.87894600	0.25767800
H	3.95309800	-2.27892200	1.91064100
O	3.46018300	0.31450500	1.32841100
C	2.16795000	1.16269500	0.29617900
C	3.83654200	1.04436600	-1.45084900
H	4.91885700	1.16050400	-1.46765600
H	3.47528600	1.19556100	-2.46901000
C	3.14077600	2.03283500	-0.47127100
H	2.55707600	2.79673400	-0.99430200
H	3.82581500	2.55459600	0.19829800
H	3.02532800	-0.12153700	2.07767600
C	1.92806000	-0.03197600	-0.53125000
C	0.77161100	-0.64196700	-0.84144400
H	0.90329900	-1.48244900	-1.52789200
Au	-1.17824600	-0.25359200	-0.30579700
C	-4.21880600	1.58077700	-0.48005900
H	-5.26681400	1.66548600	-0.18342500

H	-3.67835000	2.46868600	-0.14876200
H	-4.15496300	1.52012900	-1.56751800
C	-3.76775400	0.26256300	2.06345000
H	-4.83456300	0.39659200	2.25722300
H	-3.41610400	-0.62990600	2.58330800
H	-3.22047000	1.12628800	2.44447700
C	-4.55641600	-1.29161100	-0.25415500
H	-4.22214300	-2.21913000	0.21301800
H	-5.58879800	-1.09002100	0.04038000
H	-4.50633600	-1.41008200	-1.33751300
P	-3.45748500	0.08444800	0.26274400
C	1.22847900	1.76104300	1.28000400
H	0.48646100	2.33298400	0.71310400
H	0.69426000	1.00631800	1.85682000
H	1.74543200	2.45616400	1.94364800

Table S74: Optimized geometry for VIb

Free energy G = -1062.55363 Hartree/particle.

C	4.10393200	1.31871300	-0.89723900
C	3.38489800	-0.04804200	-0.75857900
C	1.96531400	1.67261900	0.14630900
C	3.32255900	2.26772300	0.02731000
H	5.16078500	1.28184200	-0.64208300
H	4.02410600	1.66664500	-1.92992400
H	3.28537700	3.31123300	-0.29896100
H	3.74916600	2.27658800	1.04110300
C	0.85452800	2.44902500	0.72767900
H	-0.04287400	1.85887500	0.90358100
H	1.19065100	2.91659400	1.65942600
H	0.61134600	3.27766300	0.05036800
C	1.95750100	0.36638600	-0.36940400
C	0.81869400	-0.38665900	-0.61078200
H	1.04714300	-1.30143800	-1.16393200
P	-3.51561300	-0.09021800	0.18038200
C	-4.22582400	1.59968900	0.14192400
H	-5.29996900	1.56312700	0.33705000
H	-3.74150100	2.21669600	0.90008900
H	-4.05134700	2.04672600	-0.83769600
C	-4.50138800	-1.04030600	-1.03907100
H	-4.32371100	-0.64158700	-2.03886800
H	-4.19419300	-2.08692700	-1.02067600
H	-5.56618000	-0.97047600	-0.80577000
C	-4.00561300	-0.76494400	1.81377300
H	-3.69826700	-1.80914200	1.88464300
H	-3.51094000	-0.19973900	2.60487200
H	-5.08816300	-0.69599300	1.94221300
Au	-1.17322800	-0.17907000	-0.21941300
O	4.17212700	-0.23603900	1.49375900
H	4.58290300	-0.78965600	2.16799400
C	4.05088800	-1.00617900	0.27539000
C	3.19724600	-2.25158100	0.54736800
H	3.02925900	-2.82802000	-0.36606800
H	3.71315000	-2.90345800	1.25859300
H	2.23045500	-1.98768700	0.98103700
C	5.44140000	-1.43726600	-0.21257700
H	6.09882800	-0.58099000	-0.36759400
H	5.91006100	-2.09214500	0.52771300
H	5.36824800	-1.99369700	-1.15069600
H	3.37308900	-0.58196800	-1.71121300

Table S75: Optimized geometry for TS_{4b}

Free energy G = -1062.513549 Hartree/particle.

C	4.55522200	0.22518600	0.22190300
C	3.52943700	-0.57504300	-0.55377500
C	2.43152800	1.48883500	0.08147500
C	3.76275900	1.38925600	0.86151700
H	5.07868500	-0.38668000	0.95998200
H	5.31201900	0.58669600	-0.47965900
H	4.31088000	2.33073000	0.82507500
H	3.53467200	1.18326100	1.90713100
C	2.50825800	2.49430300	-1.09055100
H	1.58327600	2.48943300	-1.67256800
H	2.65134700	3.48944900	-0.66443800
H	3.34392700	2.27793100	-1.76004900
C	2.29094200	0.13757000	-0.62425300
C	1.13474600	-0.69421200	-0.78736000
H	1.17275500	-1.31846800	-1.67797300
P	-3.11148600	0.21199400	0.21427400
C	-3.88655100	1.44443500	-0.90149500
H	-4.93482300	1.59633400	-0.63426400
H	-3.35255800	2.39252100	-0.82315100
H	-3.82406100	1.09255300	-1.93223700
C	-4.18616700	-1.26994200	0.09115200
H	-4.13402500	-1.67522000	-0.92041100
H	-3.83778800	-2.03125900	0.79075900
H	-5.22155800	-1.01126300	0.32461200
C	-3.41064200	0.85996100	1.90392800
H	-3.03465900	0.14733400	2.63941000
H	-2.88299000	1.80603900	2.03260900
H	-4.47929800	1.01735300	2.06644400
Au	-0.84126800	-0.22985100	-0.27731400
O	1.39232500	1.83941100	0.97532600
H	0.53602400	1.72545500	0.53177600
C	2.24986600	-1.42445400	0.20997700
C	2.41404300	-2.88942300	-0.22941900
H	3.26833400	-3.32870200	0.29152900
H	1.51095800	-3.43413000	0.05479500
H	2.56143300	-2.99652700	-1.30231900
C	2.08331000	-1.28865300	1.71455100
H	1.75593300	-0.29824600	2.02048400
H	1.32883800	-2.00765900	2.04249700
H	3.01820500	-1.53414500	2.22588100
H	3.86786800	-1.23663000	-1.34071900

Table S76: Optimized geometry for Vb

Free energy G = -1062.586406 Hartree/particle.

C	-4.02517200	-0.82125600	1.58536100
C	-2.76215200	-1.23831700	-0.48126800
C	-3.48965500	-1.95354600	0.68427100
H	-3.99544000	-1.06927500	2.65044200
H	-5.07005900	-0.57539400	1.35611500
H	-4.26916600	-2.63417300	0.33609000
H	-2.74642100	-2.53899700	1.23070700
C	-3.66203000	-1.07242200	-1.71418000
H	-3.17111800	-0.48031200	-2.49002100
H	-3.90543500	-2.05275000	-2.13638400
H	-4.60080600	-0.58181900	-1.44562000
C	-2.39949700	0.11756600	0.13575600

P	2.71651300	-0.82133900	0.20632800
C	2.71509500	-2.50345300	-0.51489900
H	3.66977600	-2.99354500	-0.31008700
H	1.90406700	-3.08931200	-0.08138000
H	2.56518200	-2.43706000	-1.59318000
C	4.16298400	0.01889200	-0.53562200
H	4.01118600	0.12344900	-1.61063600
H	4.27802200	1.01040600	-0.09638800
H	5.06580700	-0.56778300	-0.35146100
C	3.12873200	-1.04323100	1.97531100
H	3.21128500	-0.06731500	2.45502700
H	2.33721600	-1.61115200	2.46577900
H	4.07593200	-1.57840300	2.07316900
Au	0.70940900	0.32149300	-0.12102400
O	-1.54417900	-1.90872700	-0.86008800
H	-1.77145200	-2.67804100	-1.39594300
C	-3.13127100	0.33850100	1.24127400
H	-3.14608100	1.25796700	1.81017000
C	-0.84778800	2.16695200	-0.11810200
C	-0.92271600	2.66776000	1.30122000
H	-1.79364100	3.32729100	1.40344300
H	-1.02243000	1.86524300	2.03096400
H	-0.04005400	3.26220200	1.54410400
C	-0.29553900	3.15855900	-1.11517200
H	-0.21145700	2.73860600	-2.11799600
H	-0.97778500	4.01649200	-1.16058000
H	0.67788000	3.54379000	-0.80403600
C	-1.48075200	1.01973200	-0.57172900
H	-1.45401100	0.86742400	-1.64891400

Conditions D:

Coordinates and energies for the the cyclization of **Ib** calculated at the M06/6-31G(d,p) (C, H, P, O, N), SDD(Au) level taking into account solvent effect of CH_2Cl_2 (IEF-PCM) and employing PMe_3 as the phosphine.
Cartesian Coordinates (in Å).

Table S66: Optimized geometry for Ib

Free energy G = -1061.853359 Hartree/particle.

C	2.56269000	1.13140100	0.48838100
C	3.23677900	2.49992800	0.45145800
H	3.77714200	2.65135400	1.39164900
H	3.94674000	2.56481800	-0.37939700
H	2.49547800	3.29896000	0.33215200
O	1.69713700	1.02380400	1.60390500
H	1.19217600	1.84388800	1.68506600
C	3.61063300	0.02243900	0.62126300
H	4.21276700	0.00999400	-0.29855900
H	4.27975900	0.33214800	1.43482800
C	3.06458100	-1.37113300	0.92370900
H	3.93470700	-2.01764700	1.11295900
H	2.50149900	-1.34033200	1.86310300
C	2.24987200	-1.95307200	-0.18610400
H	2.70600800	-1.87605500	-1.17869300
C	1.05711200	-2.56108100	-0.11036500
C	0.29630200	-2.79581600	1.15921100
H	0.67408600	-2.22386200	2.01285300
H	-0.76422900	-2.53856900	1.02046200
H	0.31430900	-3.85973800	1.43286400
C	0.39505400	-3.10417100	-1.34085500
H	-0.59172000	-2.63910000	-1.49253600

H	0.99400100	-2.92917300	-2.24133000
H	0.21417200	-4.18445600	-1.25237600
C	1.79509800	0.98692600	-0.78592000
C	1.29649000	0.97444600	-1.90943100
H	1.01669000	0.99248700	-2.94502900
P	-2.54513800	0.14371300	0.47637500
C	-3.47418600	-1.32571000	-0.05828600
H	-3.63927200	-1.28468900	-1.13854000
H	-2.91352100	-2.23476600	0.17900200
H	-4.44080100	-1.35374500	0.45762500
C	-2.38706900	-0.00637000	2.28297500
H	-1.75179900	-0.86205700	2.53227400
H	-1.92301200	0.90066600	2.68254000
H	-3.37663100	-0.13966200	2.73529100
C	-3.68010500	1.54006400	0.21160600
H	-3.22193200	2.46215700	0.58055900
H	-3.88629300	1.65036500	-0.85670000
H	-4.61879600	1.35968600	0.74730700
Au	-0.46419100	0.46638100	-0.51746900

Table S67: Optimized geometry for TS_{1b}

Free energy G = -1061.844424 Hartree/particle.

C	2.16333200	1.52018500	0.11315100
C	0.92372100	2.39798400	-0.08008500
H	1.19846500	3.42756200	0.17284200
H	0.56453100	2.36481700	-1.11368900
H	0.10953400	2.08005000	0.58385000
O	2.61930500	1.59218800	1.44887000
H	1.92515300	1.25396700	2.02934600
C	3.30417300	2.01869700	-0.76409600
H	2.95192500	2.04672700	-1.80382200
H	3.55446400	3.04344900	-0.46578800
C	4.49797600	1.07755800	-0.62923700
H	5.24849300	1.31741600	-1.39152700
H	4.97177800	1.21445700	0.34721100
C	4.01988500	-0.32369700	-0.81372600
H	3.78102500	-0.61256700	-1.84054200
C	3.96744800	-1.30381100	0.13186700
C	4.32840400	-1.12926500	1.56938700
H	4.53689200	-0.09547300	1.84863900
H	3.50783800	-1.49258700	2.20209900
H	5.20025900	-1.75274000	1.80689200
C	3.55670200	-2.68389700	-0.25028600
H	2.72662700	-3.03535800	0.37845400
H	3.27401200	-2.76536300	-1.30447200
H	4.38693000	-3.37870900	-0.06757700
C	1.82207000	0.11408100	-0.25324800
C	0.96599500	-0.81151500	-0.45318200
H	1.15408400	-1.84705700	-0.72539900
P	-3.36393100	0.08068100	0.15698700
C	-3.67502200	1.44777600	1.32021700
H	-3.25699700	1.20130400	2.30053100
H	-3.19469200	2.35961100	0.95206100
H	-4.75335000	1.61775900	1.41690600
C	-4.25786500	0.55991100	-1.35619200
H	-3.80540900	1.46134900	-1.77949000
H	-4.19645400	-0.24492600	-2.09426000

H	-5.30952500	0.75887600	-1.11934400
C	-4.30909900	-1.32237300	0.82924000
H	-4.25546700	-2.16786600	0.13770000
H	-3.88450600	-1.62599700	1.79044600
H	-5.35668500	-1.03270200	0.96942500
Au	-1.08127500	-0.34700700	-0.16910300

Table S68: Optimized geometry for IIb

Free energy G = -1061.861099 Hartree/particle.

C	4.36617300	-0.94176000	0.74930400
C	3.61320500	0.36688600	0.86055000
C	2.13078400	-1.36576800	-0.16551900
C	3.60389800	-1.80839300	-0.25244400
H	5.40879200	-0.77269900	0.45571100
H	4.38400100	-1.41133000	1.73911600
H	3.70895300	-2.87474900	-0.02523600
H	3.97379700	-1.66742700	-1.27083900
C	1.39656700	-2.25741600	0.82934600
H	0.35849700	-1.93158900	0.96375800
H	1.39406500	-3.29042700	0.45914500
H	1.89295600	-2.25379300	1.80731500
C	2.14414800	0.07294200	0.37163200
C	1.01968900	0.79787900	0.66257900
H	1.20976500	1.77382800	1.11339700
P	-3.22161100	-0.14191400	-0.21555200
C	-3.66436600	-1.89817600	-0.00243100
H	-4.72415800	-2.05482200	-0.23373900
H	-3.05269200	-2.51420900	-0.66852200
H	-3.47075100	-2.20162700	1.03076500
C	-4.41475500	0.75491500	0.83125100
H	-4.23634100	0.51288800	1.88300700
H	-4.28669900	1.83250900	0.69325300
H	-5.43916300	0.47319200	0.56227300
C	-3.73504700	0.25086000	-1.91977300
H	-3.58338400	1.31696500	-2.11233400
H	-3.12904300	-0.32257600	-2.62699700
H	-4.79303700	0.00304200	-2.06194800
Au	-0.94847200	0.33429900	0.24928300
O	1.47142500	-1.38120800	-1.42159200
H	1.35695000	-2.30170500	-1.68861300
C	3.33366900	1.18862800	-0.29247900
C	3.14392900	2.66166900	-0.06220800
H	4.10548700	3.12774400	-0.31559800
H	2.38820400	3.09409600	-0.72503500
H	2.92311900	2.92292300	0.97562600
C	3.71800500	0.85421700	-1.71207700
H	3.11652100	0.05343400	-2.14578600
H	3.59223500	1.73984500	-2.34057700
H	4.77345600	0.55614400	-1.75081200
H	3.67514500	0.90018700	1.80895200

Table S69: Optimized geometry for TS_{2b}

Free energy G = -1061.837437 Hartree/particle.

C	1.73393300	1.78109700	0.08259600
C	0.76448700	2.87622400	-0.32656500
H	1.20286400	3.85648500	-0.10926800
H	0.53728300	2.82044400	-1.39747700

H	-0.18168100	2.78342600	0.22361600
O	2.01918600	1.85513800	1.46725700
H	1.18195600	1.90276200	1.94643500
C	3.05878200	1.94579600	-0.65460100
H	2.86390100	1.89319600	-1.73499000
H	3.41525500	2.96105400	-0.43936100
C	4.14350000	0.94871300	-0.25527700
H	5.08797200	1.28486600	-0.70556600
H	4.28933000	0.99554800	0.82880800
C	3.91027500	-0.45517600	-0.71670700
H	3.88605200	-0.59562800	-1.80137200
C	3.92000300	-1.57612500	0.05241900
C	3.98782500	-1.57148200	1.54449700
H	3.79570900	-0.58934600	1.98427000
H	3.25684600	-2.28031000	1.95467900
H	4.97523700	-1.91596800	1.87969800
C	3.85331100	-2.92758200	-0.57866200
H	3.00677700	-3.50678700	-0.18143300
H	3.76931700	-2.87673500	-1.66898700
H	4.75240100	-3.50562200	-0.32876200
C	1.15908500	0.40608900	-0.20840000
C	1.61420500	-0.75083500	-0.45117400
H	1.51026600	-1.79273800	-0.70165000
P	-3.25949100	-0.35729400	0.07664600
C	-4.09912600	0.89993000	1.09140100
H	-3.69047100	0.88853400	2.10595600
H	-3.93912400	1.89114800	0.65707100
H	-5.17407100	0.69049300	1.13123000
C	-4.12491100	-0.31512100	-1.52495700
H	-3.96844300	0.65756100	-2.00004200
H	-3.72787100	-1.09547900	-2.18037200
H	-5.19828500	-0.47752200	-1.37480100
C	-3.73049600	-1.94946100	0.82374600
H	-3.33649200	-2.77115500	0.21891200
H	-3.31010900	-2.02051100	1.83101400
H	-4.82219800	-2.02859000	0.87855400
Au	-0.94416200	-0.03194000	-0.09080800

Table S70: Optimized geometry for IIIb

Free energy G = -1061.863090 Hartree/particle.

P	-3.20961900	-0.27922400	0.05103200
C	-4.01715400	-0.68716200	-1.53220800
H	-5.09646500	-0.80354900	-1.38116600
H	-3.83693900	0.11117800	-2.25788500
H	-3.60374700	-1.62023200	-1.92606500
C	-3.72514300	-1.62166400	1.17191400
H	-3.29610900	-2.56886400	0.83217300
H	-3.36449800	-1.41579700	2.18387700
H	-4.81807400	-1.70214500	1.18464500
C	-4.11211600	1.18546400	0.65342700
H	-3.75750200	1.45002600	1.65406500
H	-3.93040400	2.02995300	-0.01776600
H	-5.18728300	0.97728400	0.69240200
Au	-0.86123300	0.02601800	-0.08599800
C	1.96149600	-0.82871500	-0.55206400
C	1.84714300	1.60704100	0.15810500
C	3.49103100	-0.64065500	-0.94667100
H	1.42535400	-1.64418000	-1.03389000
C	2.99901200	1.83104100	-0.83055200
C	4.05524400	0.74580300	-0.73229900

H	3.65477700	-1.08748800	-1.92940200
H	3.44083200	2.81797800	-0.63639900
H	2.58271900	1.86776300	-1.84775200
H	4.54849400	0.80592100	0.24405900
H	4.83034400	0.91440300	-1.48738200
C	1.20114800	0.25529800	-0.14106600
C	0.84504400	2.74367200	0.04303700
H	0.03260000	2.61485900	0.76771100
H	1.34372000	3.70209100	0.24012600
H	0.41288100	2.79175300	-0.96328300
O	2.31542300	1.52090900	1.49885200
H	2.60801900	2.40166200	1.76777300
C	3.31043300	-1.57160800	0.13675200
C	3.27271400	-3.04030600	-0.17970900
H	4.25600900	-3.46369000	0.05972700
H	2.53166900	-3.56008200	0.43655700
H	3.06456800	-3.23762500	-1.23561500
C	3.63795700	-1.27008300	1.56707700
H	3.03655700	-1.89914000	2.23129700
H	4.69098100	-1.53947500	1.72578200
H	3.48896200	-0.22572300	1.83949700

Table S71: Optimized geometry for TS_{3b}

Free energy G = -1061.850603 Hartree/particle.

C	3.98488900	-1.08358300	0.24248000
C	3.45123700	-0.40690500	-0.91979500
H	3.32813100	-1.12457000	-1.73787000
C	5.02020800	-0.50954900	1.11041200
H	4.98652300	-0.91576100	2.12451100
H	5.96690800	-0.85898900	0.65917800
H	5.04533700	0.57846700	1.12921700
C	3.57079500	-2.46340700	0.54080600
H	4.47530300	-3.08734100	0.58358700
H	3.14371900	-2.50974700	1.55111300
H	2.87141600	-2.88364000	-0.18242900
O	2.64634300	1.08359900	1.55184600
C	2.15315500	1.33471800	0.23049600
C	4.06300700	0.93349700	-1.31375400
H	5.14270600	0.96591300	-1.13472100
H	3.92371600	1.06240100	-2.39128100
C	3.29073900	2.01879500	-0.53794800
H	2.87379300	2.75752500	-1.23044900
H	3.91843600	2.56800800	0.17313100
H	1.94482600	0.62306800	2.03239400
C	1.98007100	-0.02041000	-0.43616500
C	0.90508100	-0.78521200	-0.62952100
H	1.09896800	-1.77045300	-1.06556400
Au	-1.08666500	-0.35977300	-0.24205700
C	-4.36870400	-1.30116700	0.77123300
H	-5.39540700	-0.97555700	0.97446800
H	-4.38062900	-2.07324300	-0.00375900
H	-3.93635800	-1.72607700	1.68191900
C	-4.30301300	0.80359700	-1.18473900
H	-5.33426300	1.01169000	-0.87712800
H	-3.83163400	1.73092800	-1.52350400
H	-4.30840000	0.09078200	-2.01425300
C	-3.57170000	1.35278600	1.53925700
H	-3.04709100	2.27370900	1.26540700
H	-4.63478500	1.57134600	1.69315800
H	-3.14229600	0.97048400	2.47044400

P	-3.35969800	0.11353200	0.21557200
C	0.91492700	2.20545300	0.30494700
H	0.49174700	2.38432300	-0.68981200
H	0.13712600	1.74637400	0.93029700
H	1.18931400	3.16898200	0.74981800

Table S72: Optimized geometry for IVb

Free energy G = -1061.865201 Hartree/particle.

C	4.00438400	-0.57723200	0.52345300
C	3.36532400	-0.49109200	-0.87254000
H	3.55046700	-1.41082300	-1.43631700
C	5.45418900	-0.19279500	0.65366100
H	5.79448000	-0.31354000	1.68688100
H	6.05099800	-0.85647900	0.01777100
H	5.64086200	0.83883300	0.34635400
C	3.68627500	-1.86513300	1.23984200
H	4.30692200	-2.66022200	0.81278600
H	3.93506600	-1.79711200	2.30541600
H	2.63599900	-2.15134300	1.12411300
O	3.21134100	0.56366200	1.23785900
C	2.14086400	1.08276800	0.18384900
C	3.76475300	0.80939100	-1.58425200
H	4.84467600	0.97343600	-1.64134400
H	3.38662100	0.78164400	-2.61057100
C	3.02434800	1.88836900	-0.75638800
H	2.37841400	2.51873100	-1.37749200
H	3.68878200	2.55390300	-0.19319700
H	2.74250700	0.19859800	2.01193900
C	1.91152600	-0.20625100	-0.54174500
C	0.78605000	-0.87109400	-0.82046100
H	0.95347100	-1.79229200	-1.39064200
Au	-1.15833000	-0.37303900	-0.32646800
C	-4.25899900	1.31367600	-0.84146200
H	-5.25578700	1.54156800	-0.44630700
H	-3.69438900	2.24356400	-0.95803200
H	-4.35857000	0.84055800	-1.82267300
C	-3.44916600	1.06076700	1.90201300
H	-4.48306600	1.33136200	2.14527100
H	-3.05086200	0.41014200	2.68665900
H	-2.83744100	1.96770200	1.85714100
C	-4.52066200	-1.21091100	0.50460700
H	-4.11939000	-1.90434800	1.24970900
H	-5.50381900	-0.85398800	0.83268800
H	-4.62674900	-1.74264300	-0.44563300
P	-3.37441500	0.19422800	0.29649100
C	1.09276700	1.81142400	0.95635000
H	0.35707400	2.20934600	0.24830100
H	0.55690000	1.15594200	1.65361700
H	1.53046200	2.65349400	1.50270300

Table S73: Optimized geometry for TS_{5b}

Free energy G = -1061.863778 Hartree/particle.

C	4.00224600	-0.68188700	0.44704100
C	3.36854200	-0.34195500	-0.92431300
H	3.51607400	-1.16683700	-1.62940800
C	5.49626200	-0.46600800	0.53740700
H	5.87393000	-0.80297100	1.50841500
H	5.99910100	-1.04428100	-0.24654300

H	5.76282000	0.58805900	0.41672700
C	3.61351500	-2.06721400	0.91321000
H	2.53495300	-2.23766600	0.82298400
H	4.13126500	-2.81389500	0.30185500
H	3.91632200	-2.22641600	1.95530000
O	3.37264400	0.33066700	1.35006800
C	2.10269100	1.17018800	0.21343200
C	3.83324500	1.02594600	-1.44240200
H	4.91885600	1.15208800	-1.45417500
H	3.47976500	1.15525800	-2.47023400
C	3.11860900	2.01696500	-0.49842800
H	2.57657100	2.80522900	-1.03776300
H	3.77964600	2.52133200	0.21502400
H	2.88464900	-0.12591400	2.05502500
C	1.92494800	-0.05266400	-0.56693600
C	0.79057200	-0.72169200	-0.84291400
H	0.95779500	-1.60161900	-1.47595200
Au	-1.16199200	-0.29998800	-0.31508700
C	-4.18981200	1.53072000	-0.59413200
H	-5.22634100	1.65643800	-0.26087500
H	-3.62856000	2.44591600	-0.38375800
H	-4.17501900	1.35040800	-1.67295200
C	-3.67362500	0.50877800	2.04049900
H	-4.73365000	0.70732500	2.23673000
H	-3.34428100	-0.33625200	2.65251800
H	-3.08391700	1.38942600	2.31302500
C	-4.56451000	-1.26304900	-0.04015700
H	-4.24495300	-2.14146900	0.52825700
H	-5.58255800	-0.98770000	0.25847700
H	-4.55454100	-1.51320000	-1.10517700
P	-3.42021600	0.12245400	0.27467900
C	1.13413500	1.77406500	1.14950000
H	0.42776800	2.36674800	0.55260300
H	0.55777200	1.02403200	1.70042100
H	1.63510400	2.45969300	1.83980000

Table S74: Optimized geometry for VIb

Free energy G = -1061.876886 Hartree/particle.

C	4.11915000	1.32301000	-0.82565700
C	3.40072500	-0.03642700	-0.75364200
C	1.95245600	1.65068300	0.12833700
C	3.30815900	2.23667100	0.09304100
H	5.17165600	1.27851600	-0.53555200
H	4.07727300	1.70560300	-1.85178500
H	3.29435900	3.30024300	-0.17591500
H	3.67876400	2.18476800	1.13169800
C	0.82587600	2.42489100	0.66178400
H	-0.05781200	1.82221000	0.89024300
H	1.14811900	2.98628700	1.54681700
H	0.54498300	3.18189300	-0.08512300
C	1.96688700	0.36074700	-0.42609700
C	0.83899200	-0.38737900	-0.70597800
H	1.08048700	-1.28358300	-1.29285300
P	-3.47773800	-0.08102400	0.22716400
C	-4.17679900	1.60262100	0.23491700
H	-5.24552400	1.56489700	0.47486900
H	-3.65891900	2.21278900	0.98080400
H	-4.04274000	2.06121500	-0.74912700
C	-4.53207600	-0.99936000	-0.94222600
H	-4.40687500	-0.59065500	-1.94917300

H	-4.24121700	-2.05381800	-0.95203400
H	-5.58333900	-0.91492400	-0.64426900
C	-3.92041700	-0.75953000	1.86027700
H	-3.62577500	-1.81159100	1.91326000
H	-3.39502200	-0.20718900	2.64475600
H	-5.00162800	-0.67673100	2.01912000
Au	-1.15731900	-0.18349400	-0.26308700
O	3.99058200	-0.25891300	1.52988800
H	4.35839600	-0.82652900	2.21760800
C	3.99115500	-0.99255800	0.30670800
C	3.14448900	-2.24917500	0.46597600
H	3.06719700	-2.80538900	-0.47653000
H	3.60762900	-2.91462600	1.20546400
H	2.13480600	-2.01341900	0.82362400
C	5.41283300	-1.39498800	-0.06696300
H	6.07891900	-0.52917500	-0.12937500
H	5.81894700	-2.07573000	0.69168800
H	5.43355900	-1.92060600	-1.02899000
H	3.44479400	-0.56698200	-1.71478800

Table S75: Optimized geometry for TS_{4b}

Free energy G = -1061.855668 Hartree/particle.

C	4.55438200	0.38850200	0.15076000
C	3.58447200	-0.45593900	-0.61104200
C	2.35804800	1.46045100	0.09283700
C	3.68758900	1.43944000	0.85572000
H	5.16808100	-0.20998200	0.83444000
H	5.25163100	0.83692400	-0.57063800
H	4.15259600	2.42962200	0.87412100
H	3.48797800	1.15229100	1.89334000
C	2.37365400	2.45484600	-1.06792800
H	1.45593200	2.37708200	-1.66537600
H	2.43973700	3.46731000	-0.65592900
H	3.22942300	2.29214700	-1.73322300
C	2.30121900	0.09008000	-0.59645200
C	1.15181000	-0.78608000	-0.77878300
H	1.21367100	-1.38893600	-1.68948000
P	-3.08380800	0.22830200	0.20417200
C	-3.58034900	1.93872300	-0.18552400
H	-4.63763600	2.09197700	0.05874800
H	-2.97000200	2.63934200	0.39185000
H	-3.42328700	2.13254900	-1.25078400
C	-4.28847500	-0.80071200	-0.69896300
H	-4.15423100	-0.66261800	-1.77590900
H	-4.12528900	-1.85531100	-0.45839300
H	-5.31046900	-0.51871800	-0.42108000
C	-3.53853600	0.01701000	1.95765600
H	-3.35870800	-1.01882500	2.26034000
H	-2.92372700	0.67477800	2.57939800
H	-4.59664000	0.26022600	2.10803200
Au	-0.82704400	-0.27227600	-0.28516900
O	1.31924400	1.75874400	0.98533000
H	0.48084700	1.77592800	0.49707500
C	2.15240300	-1.39649500	0.23807500
C	2.57901900	-2.81615900	-0.11136300
H	3.48699100	-3.10511800	0.42899100
H	1.76147200	-3.46916500	0.21801600
H	2.72553600	-2.97826500	-1.18097900
C	1.98939300	-1.21375000	1.72518900
H	1.58554800	-0.23706700	1.99532300

H	1.28866200	-1.98252800	2.07466900
H	2.94157300	-1.38027800	2.24430600
H	3.92218600	-1.22754800	-1.29481500

Table S76: Optimized geometry for Vb

Free energy G = -1061.914039 Hartree/particle.

C	3.94956200	-0.99075900	-1.45395000
C	2.48586700	-1.31882500	0.46449800
C	3.16222600	-2.06486100	-0.69469100
H	4.01002700	-1.17748500	-2.53260000
H	4.98908100	-0.91926500	-1.09788900
H	3.77768000	-2.90588800	-0.35519400
H	2.36605400	-2.46093700	-1.33723200
C	3.32820500	-1.34051800	1.73295300
H	2.89094400	-0.70913700	2.51522800
H	3.40457400	-2.36406700	2.12253000
H	4.34505700	-0.98511600	1.52903400
C	2.36582700	0.09299900	-0.08622600
P	-2.64152300	-0.78684600	-0.21838700
C	-2.56747600	-2.48676600	0.42867200
H	-3.52075900	-2.99595800	0.24589100
H	-1.75978900	-3.03069100	-0.07033300
H	-2.36563100	-2.46504000	1.50364400
C	-4.10464700	-0.04267600	0.56619800
H	-3.94675800	0.03174900	1.64574900
H	-4.27044200	0.95992700	0.16207900
H	-4.98494200	-0.66462300	0.36826800
C	-3.08725100	-0.97329900	-1.97233800
H	-3.22992400	0.01267700	-2.42323500
H	-2.28147800	-1.49042900	-2.50099500
H	-4.01299200	-1.55330600	-2.06054000
Au	-0.64318000	0.36346000	0.12814500
O	1.17615900	-1.80911200	0.74422400
H	1.25355100	-2.62001300	1.26148000
C	3.20423700	0.26521400	-1.12073400
H	3.39264000	1.20931300	-1.62475500
C	0.93972200	2.21699000	0.09504600
C	1.04128500	2.64177700	-1.33635000
H	1.90236900	3.31476200	-1.45531300
H	1.17605900	1.80117500	-2.02303100
H	0.15110700	3.20777400	-1.63185500
C	0.36414300	3.24136800	1.02549500
H	0.26321200	2.86669700	2.04854800
H	1.02836100	4.11666400	1.04291500
H	-0.61215700	3.59541800	0.67494200
C	1.51645500	1.06621800	0.60170400
H	1.47002200	0.94686700	1.68957200

<u>Conditions E:</u>
Coordinates and energies for the the cyclization of **Ib** calculated at the M06/6-31G(d,p) (C, H, P, O, N), SDD(Au) level taking into account solvent effect of CH_2Cl_2 (SMD) and employing PMe_3 as the phosphine.
Cartesian Coordinates (in Å).

Table S77: Optimized geometry for Ib

Free energy G = -061,875435 Hartree/particle.

C	-2.62703000	-1.12944800	0.44285200
C	-3.32966800	-2.47698700	0.30730600
H	-3.91968400	-2.66090300	1.21199800

H	-3.99901900	-2.48703500	-0.55962000
H	-2.60022800	-3.28753300	0.18770800
O	-1.80313600	-1.10872500	1.59395900
H	-1.31876100	-1.94686500	1.63523400
C	-3.65954800	-0.00915400	0.59745900
H	-4.25175000	0.03365000	-0.32798700
H	-4.33923600	-0.33627900	1.39571700
C	-3.10965000	1.37381000	0.94095600
H	-3.97938000	2.01052300	1.16470000
H	-2.53132900	1.31602600	1.87047400
C	-2.31638100	1.99400500	-0.16389300
H	-2.80564600	1.97251700	-1.14390900
C	-1.10194200	2.55906000	-0.10033500
C	-0.28493300	2.69395600	1.14811600
H	-0.69240800	2.14308200	2.00283400
H	0.74121200	2.33614800	0.97286700
H	-0.19328000	3.74928400	1.44252000
C	-0.45115100	3.11939500	-1.32834500
H	0.50273700	2.60699400	-1.53428600
H	-1.08604800	3.01987500	-2.21621200
H	-0.20376600	4.18315600	-1.20176800
C	-1.80649300	-0.93914100	-0.79085200
C	-1.26351300	-0.88995300	-1.89261900
H	-0.95023800	-0.86869200	-2.92058300
P	2.59373600	-0.12873200	0.44396600
C	3.45177600	1.33552500	-0.20335600
H	3.52419300	1.27374300	-1.29388800
H	2.89693800	2.24087500	0.06504300
H	4.45972100	1.39002600	0.22566800
C	2.55405400	0.09362600	2.24700200
H	1.93051400	0.95672600	2.50308200
H	2.13090100	-0.79928500	2.71866600
H	3.57248500	0.25467600	2.62094700
C	3.73724600	-1.51250000	0.16400300
H	3.31692300	-2.43068100	0.58648800
H	3.89167700	-1.65675700	-0.91004700
H	4.69985400	-1.29872800	0.64427700
Au	0.48058300	-0.48337900	-0.46532700

Table S78: Optimized geometry for TS_{1b}

Free energy G = -1061.862853 Hartree/particle.

C	2.16390300	1.51933900	0.11723600
C	0.91488600	2.38609400	-0.04956400
H	1.18114700	3.41644900	0.21243000
H	0.54388000	2.36307600	-1.07980000
H	0.11176900	2.04774800	0.61909700
O	2.64159200	1.59096500	1.44646200
H	1.94932500	1.25487200	2.03397400
C	3.28588100	2.02422700	-0.77891800
H	2.91248700	2.05354100	-1.81129000
H	3.54134700	3.04857800	-0.48258600
C	4.48136700	1.08202000	-0.66955700
H	5.21191900	1.31533700	-1.45337900
H	4.98122900	1.22212200	0.29375600
C	3.99553600	-0.31970800	-0.83345700
H	3.73660100	-0.61798700	-1.85270500
C	3.97487900	-1.29640400	0.11897000
C	4.37130300	-1.11131200	1.54473400
H	4.57564800	-0.07407700	1.81633200

H	3.57477900	-1.48534600	2.20213900
H	5.25787700	-1.72313700	1.76029200
C	3.55879100	-2.67909400	-0.24251000
H	2.73365600	-3.02121700	0.39858400
H	3.26561800	-2.77319200	-1.29312800
H	4.39033100	-3.37259900	-0.05678700
C	1.82964700	0.10880600	-0.24453700
C	0.96752900	-0.81532300	-0.43357300
H	1.15560500	-1.85168200	-0.70675000
P	-3.36616000	0.08229400	0.14323900
C	-3.71187300	1.13265800	1.58856500
H	-3.37230700	0.63182100	2.50098700
H	-3.17582700	2.08260100	1.48865300
H	-4.78820500	1.33068400	1.66258400
C	-4.12852200	0.96820800	-1.25140100
H	-3.61572500	1.92343500	-1.40438100
H	-4.04023900	0.37155900	-2.16495500
H	-5.18858700	1.15590900	-1.04123300
C	-4.40951300	-1.38744200	0.38304500
H	-4.32796300	-2.04778000	-0.48599400
H	-4.07849300	-1.93366300	1.27215900
H	-5.45546300	-1.08403000	0.51176000
Au	-1.08207100	-0.35275400	-0.15385100

Table S79: Optimized geometry for IIb

Free energy G = -1061,878888 Hartree/particle.

C	4.28984600	1.01658500	-0.86351600
C	3.58935100	-0.32400300	-0.91367700
C	2.09323000	1.36840700	0.16621300
C	3.55099900	1.86534500	0.16925400
H	5.35336500	0.89338900	-0.62593900
H	4.22906600	1.47171200	-1.85853700
H	3.60293200	2.93322600	-0.07081600
H	3.98035300	1.74385500	1.16761200
C	1.27300300	2.21457300	-0.80157900
H	0.24712500	1.83116200	-0.89978700
H	1.22843000	3.24876600	-0.43549500
H	1.73405300	2.23198700	-1.79737200
C	2.13247700	-0.07960700	-0.34789600
C	1.02338700	-0.84207900	-0.59165200
H	1.22758800	-1.82205800	-1.02812400
P	-3.22514500	0.15829700	0.17482400
C	-3.65850000	1.79728500	-0.49281500
H	-4.71095700	2.02776700	-0.28699200
H	-3.02325600	2.56049900	-0.03027800
H	-3.48882500	1.81242200	-1.57452800
C	-4.43347200	-0.96885500	-0.59033900
H	-4.25970300	-1.01481800	-1.67042000
H	-4.31838200	-1.97531300	-0.17494900
H	-5.45377500	-0.61343700	-0.40237000
C	-3.73399900	0.24784400	1.92091300
H	-3.60449100	-0.72909800	2.39781800
H	-3.11345200	0.97973800	2.44815200
H	-4.78661600	0.54761100	1.98847400
Au	-0.95020300	-0.38676600	-0.19200100
O	1.50654600	1.38752200	1.45957800
H	1.40178400	2.31312900	1.72068600
C	3.40728500	-1.13885900	0.26070500
C	3.25103000	-2.61845000	0.05747500
H	4.23888300	-3.05337200	0.26346900

H	2.54472500	-3.06480800	0.76492200
H	2.98314200	-2.89694600	-0.96507100
C	3.85344000	-0.77175100	1.65251700
H	3.23313800	-0.00389500	2.11939300
H	3.81549300	-1.65917700	2.29117100
H	4.89013200	-0.41175800	1.63142700
H	3.61166300	-0.86931000	-1.85697900

Table S80: Optimized geometry for TS_{2b}

Free energy G = -1061.857603 Hartree/particle.

C	1.71976200	1.77973200	0.08558600
C	0.73803100	2.86720500	-0.31191000
H	1.16991300	3.85207700	-0.09887000
H	0.49860900	2.81223700	-1.38077000
H	-0.19995900	2.76422600	0.25170000
O	2.00391600	1.85090900	1.47197400
H	1.16109600	1.90674600	1.94473600
C	3.04082300	1.95954300	-0.65384700
H	2.84122700	1.91158500	-1.73375400
H	3.39236600	2.97581600	-0.43356700
C	4.13286200	0.96619800	-0.26634300
H	5.06962200	1.30334700	-0.73268700
H	4.29587800	1.01680900	0.81549400
C	3.90061000	-0.44165400	-0.71852500
H	3.87568700	-0.59086100	-1.80210100
C	3.93147900	-1.55875200	0.05835400
C	3.99928500	-1.54574300	1.54899800
H	3.80931800	-0.56233700	1.98770100
H	3.26809100	-2.25415600	1.96104800
H	4.98619300	-1.89073100	1.88699300
C	3.87194400	-2.91537200	-0.55847100
H	3.00712500	-3.47863400	-0.17645000
H	3.81721300	-2.87794700	-1.65135700
H	4.75844300	-3.49903600	-0.27568400
C	1.15800600	0.40125100	-0.21169500
C	1.63557600	-0.74725200	-0.46092300
H	1.52936000	-1.78848000	-0.72034600
P	-3.25561000	-0.34785400	0.08158000
C	-4.06053500	0.86033100	1.17699100
H	-3.64428700	0.78090600	2.18649800
H	-3.88836100	1.87496400	0.80349900
H	-5.13930300	0.66690200	1.21459800
C	-4.15413500	-0.21107200	-1.49397400
H	-4.00959100	0.78762200	-1.91855100
H	-3.77304600	-0.95190200	-2.20408800
H	-5.22441400	-0.38330900	-1.32752400
C	-3.74461900	-1.96781000	0.75017800
H	-3.38089400	-2.76415200	0.09268100
H	-3.30674400	-2.10629000	1.74397400
H	-4.83730500	-2.02735700	0.82328200
Au	-0.94035400	-0.04743200	-0.10028400

Table S81: Optimized geometry for IIIb

Free energy G = -1061.884046 Hartree/particle.

P	3.20504800	0.27728300	0.05380100
C	4.00473700	0.68842300	-1.53073300
H	5.08169600	0.83216500	-1.38111700
H	3.84374000	-0.12148800	-2.24958000

H	3.57072200	1.60795800	-1.93691300
C	3.71018200	1.62655800	1.16848900
H	3.27446400	2.57069600	0.82550500
H	3.35024900	1.42361400	2.18231900
H	4.80311000	1.71556400	1.18213000
C	4.12777100	-1.17249600	0.65555700
H	3.78872600	-1.43737500	1.66248000
H	3.94801500	-2.02490000	-0.00777700
H	5.20207500	-0.95354300	0.68093200
Au	0.85947900	-0.03187900	-0.08784200
C	-1.96362400	0.82038000	-0.57360400
C	-1.85146100	-1.60855500	0.15530500
C	-3.49977800	0.64382900	-0.93637500
H	-1.43051200	1.63364900	-1.06235800
C	-3.03002100	-1.83137400	-0.80029500
C	-4.07305300	-0.73487100	-0.69580900
H	-3.68047600	1.08675600	-1.91801100
H	-3.47654100	-2.81095700	-0.57938800
H	-2.63713400	-1.88681600	-1.82590900
H	-4.55139400	-0.77944600	0.28914700
H	-4.86239600	-0.90400700	-1.43639600
C	-1.20367200	-0.26027400	-0.15454900
C	-0.85905800	-2.74948400	0.01099000
H	-0.01994200	-2.62212400	0.70564800
H	-1.35581000	-3.70490700	0.22968500
H	-0.46208900	-2.80429500	-1.00988100
O	-2.28305400	-1.52356400	1.50993800
H	-2.57831600	-2.40701800	1.77558900
C	-3.29671100	1.58392700	0.13537500
C	-3.25206000	3.04785600	-0.19398600
H	-4.23234700	3.47840100	0.04855000
H	-2.50447200	3.57055000	0.41258900
H	-3.05000600	3.23334100	-1.25367900
C	-3.59254300	1.29603400	1.57423700
H	-2.96450300	1.91909300	2.22041000
H	-4.63706600	1.58234000	1.76071100
H	-3.45420600	0.25069400	1.85070200

Table S82: Optimized geometry for TS_{3b}

Free energy G = -1061.871581 Hartree/particle.

C	3.96615000	-1.12598500	0.23640100
C	3.45848600	-0.41146200	-0.91775000
H	3.33260400	-1.11475700	-1.74804800
C	4.98950400	-0.58967800	1.13769500
H	4.90770200	-1.00136000	2.14798500
H	5.93639200	-0.97926900	0.71849600
H	5.05934900	0.49696200	1.16007600
C	3.52399900	-2.50329700	0.49077200
H	4.41981200	-3.13956000	0.54661100
H	3.07020200	-2.56528200	1.48929100
H	2.84033200	-2.89673800	-0.26277600
O	2.64304400	1.07303400	1.56431900
C	2.18104700	1.34508000	0.23492000
C	4.10682300	0.92019600	-1.28111300
H	5.18353200	0.92460200	-1.08132500
H	3.98808900	1.06751500	-2.35916700
C	3.34723100	2.01235100	-0.50410900
H	2.96021100	2.77116200	-1.19279900
H	3.97636800	2.53351000	0.22675400
H	1.91336100	0.63757300	2.02986300

C	1.99318900	-0.00221500	-0.44648700
C	0.90498200	-0.74812200	-0.64550300
H	1.08991500	-1.73168100	-1.09082000
Au	-1.08838000	-0.32833500	-0.24629500
C	-3.62460300	1.04326200	1.75225600
H	-4.69218700	1.23435000	1.91624900
H	-3.22274500	0.48472600	2.60422300
H	-3.09343500	1.99888000	1.68166500
C	-4.41178800	-1.38675500	0.43847600
H	-5.44129100	-1.09492400	0.67896800
H	-4.41049500	-1.98012200	-0.48177500
H	-4.01057900	-2.00281500	1.24977200
C	-4.24666300	1.06349800	-1.04965800
H	-4.23401200	0.52302300	-2.00184300
H	-5.28618600	1.23653300	-0.74541000
H	-3.74562100	2.02710000	-1.18881700
P	-3.36971100	0.09128100	0.21850500
C	0.95891300	2.23884500	0.29237200
H	0.56722900	2.44736500	-0.70989700
H	0.15307500	1.78422800	0.88554800
H	1.23809400	3.18966900	0.76270800

Table S83: Optimized geometry for IVb

Free energy G = -1061.884003 Hartree/particle.

C	4.04454700	-0.64628100	0.42665500
C	3.36019000	-0.44146200	-0.93380700
H	3.50560600	-1.32010700	-1.56987100
C	5.50176700	-0.29176600	0.53645500
H	5.87131800	-0.49137300	1.54777600
H	6.06667800	-0.91930200	-0.16294700
H	5.69720000	0.75588800	0.29478600
C	3.73183200	-1.98020100	1.05267100
H	4.31869700	-2.74870400	0.53698600
H	4.02498700	-1.99893000	2.10947100
H	2.67196200	-2.24102700	0.96135800
O	3.29040500	0.44668900	1.25864500
C	2.20989300	1.07093200	0.28483900
C	3.76334500	0.90354600	-1.55729100
H	4.84507500	1.04693300	-1.63870100
H	3.34705400	0.96726100	-2.56753700
C	3.08214300	1.92854500	-0.61820200
H	2.43265600	2.62539600	-1.15955500
H	3.78625000	2.52437000	-0.02560300
H	2.82112900	0.01615900	2.00448900
C	1.92526700	-0.15311200	-0.53203700
C	0.77837000	-0.77690900	-0.81807600
H	0.91821400	-1.65787700	-1.45733900
Au	-1.16432700	-0.30137500	-0.28747600
C	-4.24383600	1.40381000	-0.77262500
H	-5.28901400	1.52932600	-0.46457100
H	-3.72045600	2.36037800	-0.67252200
H	-4.21179300	1.09740500	-1.82332600
C	-3.71547200	0.72408800	1.96384700
H	-4.78652500	0.88289400	2.13895000
H	-3.33747700	-0.01652000	2.67647700
H	-3.18092500	1.66662800	2.12418100
C	-4.51554400	-1.31324900	0.10334900
H	-4.16580300	-2.10881400	0.76964600
H	-5.54917400	-1.05200100	0.36154000
H	-4.48373100	-1.68456200	-0.92641900

P	-3.42831500	0.14170700	0.26005100
C	1.19698100	1.75127700	1.14047700
H	0.43316400	2.18859100	0.48730600
H	0.69392700	1.05221100	1.81994400
H	1.65737800	2.55995200	1.71866800

Table S84: Optimized geometry for TS_{5b}

Free energy G = -1061.883356 Hartree/particle.

C	3.99606800	-0.62926100	0.48177200
C	3.36492900	-0.35694000	-0.90571000
H	3.53672700	-1.20580500	-1.57599600
C	5.48163000	-0.36666800	0.57373400
H	5.85813800	-0.65046600	1.56280800
H	6.00900000	-0.96556400	-0.17837700
H	5.71966700	0.68823100	0.40645600
C	3.64849000	-2.00896900	0.99316200
H	2.57565700	-2.21793300	0.90619300
H	4.19232800	-2.76164900	0.41137400
H	3.94978400	-2.12063300	2.04247600
O	3.33233700	0.39124900	1.34748500
C	2.04959400	1.17296100	0.15659800
C	3.80246200	1.00142700	-1.47237100
H	4.88536500	1.15184200	-1.47809200
H	3.45518500	1.08562000	-2.50729300
C	3.05924800	2.01033000	-0.57132000
H	2.50770400	2.77059000	-1.14121400
H	3.70189800	2.54962100	0.13367600
H	2.83023900	-0.06300900	2.04857700
C	1.91116600	-0.08748500	-0.57255700
C	0.79542300	-0.79715000	-0.82470200
H	0.98732100	-1.69994900	-1.41796800
Au	-1.15995000	-0.35694400	-0.31740600
C	-3.51324600	1.68032600	1.28718000
H	-4.55929700	1.89981900	1.53280800
H	-2.94306700	1.55062000	2.21377200
H	-3.08744100	2.52378100	0.73264500
C	-4.24396500	-1.09873500	1.28236100
H	-5.25744500	-0.76538300	1.53678800
H	-4.30350500	-2.03562500	0.71856100
H	-3.68152800	-1.28208400	2.20372100
C	-4.52834600	0.46342400	-1.11067200
H	-4.58099300	-0.42876500	-1.74320700
H	-5.53161600	0.70331500	-0.73804000
H	-4.15836500	1.29776200	-1.71545800
P	-3.39181700	0.16407500	0.28219100
C	1.06638900	1.79279600	1.06437800
H	0.36873800	2.37606300	0.44646400
H	0.48379100	1.05442000	1.62546300
H	1.55728700	2.49630000	1.74505300

Table S85: Optimized geometry for VIb

Free energy G = -1061.896906 Hartree/particle.

C	4.08653700	1.34739100	-0.84383000
C	3.39779500	-0.02633700	-0.75227600
C	1.91852900	1.63855500	0.12317600
C	3.25610600	2.25931700	0.05856500
H	5.14049100	1.32877300	-0.55413100
H	4.03620900	1.71283200	-1.87616500

H	3.20186600	3.31375800	-0.24232000
H	3.63433600	2.25957800	1.09566100
C	0.78059500	2.38776900	0.66705300
H	-0.04347000	1.75214400	1.00752700
H	1.11833400	3.04936900	1.47327600
H	0.39526300	3.04711000	-0.12611900
C	1.95725700	0.34329400	-0.42149800
C	0.84253900	-0.42474200	-0.69568500
H	1.09573600	-1.32084400	-1.27822800
P	-3.47342400	-0.06171000	0.22651500
C	-4.09673800	1.64766900	0.31425000
H	-5.16889700	1.64773400	0.54487800
H	-3.55905500	2.19546500	1.09565700
H	-3.93428400	2.15205500	-0.64395500
C	-4.56586500	-0.87792200	-0.98035900
H	-4.42510400	-0.43008200	-1.96961900
H	-4.32002000	-1.94312600	-1.03992300
H	-5.61383400	-0.76561400	-0.67718800
C	-3.94688300	-0.78923500	1.82694300
H	-3.70759500	-1.85758800	1.83286500
H	-3.39078500	-0.30111900	2.63394800
H	-5.02221400	-0.65791400	1.99818500
Au	-1.15620600	-0.21544500	-0.26402200
O	4.00742500	-0.21691500	1.53256000
H	4.38438400	-0.78098600	2.22108600
C	4.01440400	-0.95937400	0.31391000
C	3.19682300	-2.23199600	0.48989800
H	3.12738000	-2.80123100	-0.44567200
H	3.67805700	-2.87831900	1.23571600
H	2.18231300	-2.01578200	0.84738500
C	5.44194500	-1.33564500	-0.06237700
H	6.08993300	-0.45675000	-0.14297600
H	5.86725400	-1.99498800	0.70566100
H	5.47060100	-1.87699400	-1.01617900
H	3.44710600	-0.56883500	-1.70645500

Table S86: Optimized geometry for TS_{4b}

Free energy G = -1061.875313 Hartree/particle.

C	4.53186900	0.44183700	0.16973200
C	3.58645000	-0.42621800	-0.59455800
C	2.31758000	1.47341600	0.07998400
C	3.64021600	1.48367500	0.85475000
H	5.14607000	-0.14217600	0.86602900
H	5.22897300	0.89429300	-0.54955200
H	4.08666100	2.48292300	0.86561800
H	3.43620400	1.20227300	1.89343900
C	2.32360100	2.45697000	-1.08858200
H	1.40750200	2.35934900	-1.68675800
H	2.37387700	3.47624700	-0.68966900
H	3.18412300	2.29879000	-1.74987600
C	2.29243200	0.09284600	-0.59610500
C	1.16204400	-0.81065100	-0.78145800
H	1.25024800	-1.42411700	-1.68306300
P	-3.06497200	0.23903400	0.21219500
C	-4.26729000	-0.11027000	-1.11126600
H	-5.27490500	0.18788100	-0.79671400
H	-3.99167500	0.44198300	-2.01577000
H	-4.26232200	-1.18085700	-1.34103100
C	-3.74934800	-0.61218400	1.67055000
H	-3.73689000	-1.69469600	1.50724200

H	-3.14000600	-0.38486600	2.55148000
H	-4.78075700	-0.28473800	1.84922700
C	-3.30356400	2.00807600	0.57906400
H	-2.67722700	2.29584000	1.43047700
H	-3.00961300	2.60677700	-0.28975500
H	-4.35460100	2.21116800	0.81818900
Au	-0.82496200	-0.30891500	-0.29902500
O	1.26593400	1.76623800	0.96100500
H	0.43170000	1.75864900	0.46090200
C	2.16314300	-1.38377400	0.25370100
C	2.62989700	-2.79557000	-0.06942000
H	3.54173400	-3.05282500	0.48121900
H	1.82853900	-3.46618800	0.26628000
H	2.78906200	-2.96943300	-1.13593500
C	1.97786100	-1.18730600	1.73595900
H	1.55018900	-0.21684000	1.99235800
H	1.29162600	-1.96891400	2.08772800
H	2.92705400	-1.32475700	2.26939600
H	3.94422800	-1.20198600	-1.26355600

Table S87: Optimized geometry for Vb

Free energy G = -1061.934948 Hartree/particle.

C	-4.00832400	-0.92468300	1.44041100
C	-2.52266900	-1.31026500	-0.45221900
C	-3.23577500	-2.02543000	0.70528400
H	-4.08434700	-1.09559600	2.52119600
H	-5.04231900	-0.83645000	1.07138100
H	-3.86751900	-2.85410100	0.36402900
H	-2.46079900	-2.43496300	1.36579400
C	-3.34147400	-1.33843400	-1.73472100
H	-2.88321100	-0.72094800	-2.51689900
H	-3.41979200	-2.36685800	-2.11282900
H	-4.35924400	-0.97062800	-1.55597500
C	-2.38346700	0.10799300	0.07904000
P	2.65812900	-0.78391500	0.22108900
C	2.47985500	-2.56930000	-0.06992100
H	3.44475500	-3.07069900	0.07384100
H	1.74683300	-2.98404400	0.62969500
H	2.12939300	-2.74405100	-1.09249300
C	4.01970700	-0.26364200	-0.86478300
H	3.73366200	-0.40318800	-1.91224200
H	4.24286400	0.79487700	-0.69786000
H	4.91291300	-0.86258800	-0.64905800
C	3.32060900	-0.64582700	1.90767800
H	3.52569500	0.40400400	2.14004100
H	2.58769400	-1.03015700	2.62433700
H	4.24886300	-1.22439200	1.98979500
Au	0.65099500	0.34978000	-0.12472700
O	-1.22025000	-1.83506200	-0.70191200
H	-1.31740100	-2.66915300	-1.18172800
C	-3.23341200	0.31060300	1.09899300
H	-3.40995200	1.26596400	1.58682300
C	-0.92554700	2.20923800	-0.10960700
C	-1.02396000	2.64972800	1.31665300
H	-1.86354300	3.35158100	1.42576800
H	-1.18831800	1.82144100	2.01242200
H	-0.11779900	3.19193800	1.61133800
C	-0.32129300	3.21241600	-1.04375800
H	-0.22021300	2.82662900	-2.06337100
H	-0.96531300	4.10332200	-1.07508100

H	0.65976200	3.54989000	-0.68797600
C	-1.51043500	1.06029300	-0.61010700
H	-1.45408000	0.92861400	-1.69622000

Conditions F:
Coordinates and energies for the the cyclization of **Ib** calculated at the M06/6-311++G(d,p) (C, H, P, O, N), SDD(Au) level taking into account solvent effect of CH_2Cl_2 (IEF-PCM) and employing PMe_3 as the phosphine. Cartesian Coordinates (in Å).

Table S88: Optimized geometry for Ib

Free energy G = -1062.016988 Hartree/particle.

C	-2.57460000	-1.12911100	0.49728700
C	-3.24299700	-2.49842400	0.45785200
H	-3.81661500	-2.63880900	1.37835600
H	-3.92081000	-2.57412900	-0.39708100
H	-2.49700900	-3.29566200	0.37447100
O	-1.74195900	-1.00256100	1.63657500
H	-1.23775100	-1.81571300	1.75449100
C	-3.63080400	-0.02579800	0.58944300
H	-4.20672400	-0.02602300	-0.34567800
H	-4.31965300	-0.33003300	1.38679300
C	-3.10189100	1.37227300	0.89113600
H	-3.97958100	2.01205000	1.06073700
H	-2.55466200	1.35293200	1.83828900
C	-2.27729900	1.95602000	-0.20778200
H	-2.72331700	1.88178200	-1.20323800
C	-1.08754200	2.55663900	-0.11961600
C	-0.33536200	2.77998300	1.15450100
H	-0.72133000	2.20782100	2.00173600
H	0.72280500	2.51914500	1.02037800
H	-0.35137300	3.84251000	1.42977800
C	-0.40779800	3.09615500	-1.33906800
H	0.57321700	2.61991500	-1.48094000
H	-0.99956500	2.93408000	-2.24458400
H	-0.21528400	4.17247800	-1.24102600
C	-1.78258500	-0.99694700	-0.75859900
C	-1.30576500	-0.99672600	-1.88273700
H	-1.02844300	-1.02352700	-2.91554700
P	2.56124000	-0.12476300	0.47909200
C	3.46610100	1.35563700	-0.05291700
H	3.62647500	1.32277600	-1.13279700
H	2.89686700	2.25525400	0.19317500
H	4.43404300	1.39134600	0.45765500
C	2.39411700	0.02961600	2.28027500
H	1.74345300	0.87368900	2.52233500
H	1.94686000	-0.88285300	2.68247500
H	3.37935000	0.18313300	2.73314900
C	3.71875100	-1.49893900	0.22544200
H	3.27275100	-2.42832100	0.58659400
H	3.94177400	-1.60317700	-0.83857100
H	4.64512400	-1.30181900	0.77439100
Au	0.47990500	-0.47796400	-0.52007100

Table S89: Optimized geometry for TS_{1b}

Free energy G = -1062.005315 Hartree/particle.

C	2.16306600	1.52054800	0.10846300
C	0.92301300	2.38534600	-0.10717700

H	1.18612100	3.41964900	0.13381800
H	0.57864500	2.33477100	-1.14344400
H	0.10419600	2.06903000	0.54877300
O	2.59343500	1.59993900	1.45887200
H	1.89443600	1.25973600	2.02900100
C	3.31387600	2.02824000	-0.74413600
H	2.97344500	2.07358900	-1.78592100
H	3.57167800	3.04444800	-0.42951200
C	4.48838700	1.06788300	-0.61105300
H	5.24378300	1.28968600	-1.37188300
H	4.96382000	1.19060600	0.36461500
C	3.97658900	-0.31977000	-0.79911100
H	3.74896200	-0.60623200	-1.82713300
C	3.93539300	-1.30783600	0.13821600
C	4.30707200	-1.14461300	1.57035700
H	4.52825100	-0.11705700	1.85602700
H	3.49131400	-1.50951600	2.20606600
H	5.17519800	-1.77779100	1.79119000
C	3.52804000	-2.68210900	-0.25245600
H	2.71703000	-3.04986900	0.38869800
H	3.22909200	-2.75370800	-1.30070000
H	4.37142300	-3.36532100	-0.09305700
C	1.85707500	0.10638300	-0.25885800
C	0.98055200	-0.79596100	-0.46025300
H	1.15934300	-1.83061700	-0.73495000
P	-3.36069700	0.07874200	0.16407500
C	-3.66827700	1.43934000	1.32989400
H	-3.24604600	1.19143900	2.30653600
H	-3.19284900	2.35257400	0.96316000
H	-4.74566400	1.60557300	1.43143700
C	-4.26911800	0.55839400	-1.33553100
H	-3.82555300	1.46181300	-1.76054600
H	-4.21309400	-0.24287000	-2.07592600
H	-5.31778900	0.75163500	-1.08599600
C	-4.30013000	-1.32169900	0.83972000
H	-4.25172200	-2.16594100	0.14842400
H	-3.87101900	-1.62573600	1.79721300
H	-5.34545800	-1.03048500	0.98557400
Au	-1.07187100	-0.34373300	-0.17698800

Table S90: Optimized geometry for IIb

Free energy G = -1062.016335 Hartree/particle.

C	4.35743000	0.93641100	-0.78932900
C	3.60486100	-0.37178800	-0.87239000
C	2.14750100	1.37017800	0.17176100
C	3.62121600	1.80793200	0.22351700
H	5.40564900	0.77069300	-0.51986300
H	4.35019900	1.39827000	-1.78140900
H	3.72204700	2.87170500	-0.00994600
H	4.01352400	1.66968500	1.23202900
C	1.39430600	2.25546300	-0.81086300
H	0.35348400	1.93396600	-0.91506900
H	1.40928000	3.29131100	-0.45262500
H	1.86643400	2.23604600	-1.79905900
C	2.14294200	-0.07084300	-0.35577500
C	1.02198600	-0.79460900	-0.63216100
H	1.20961500	-1.77401700	-1.07250600
P	-3.23827300	0.14046200	0.20750100
C	-3.67828200	1.89525900	0.01142700

H	-4.73950000	2.04610700	0.23495400
H	-3.07531700	2.50265000	0.69092300
H	-3.47611600	2.21274300	-1.01439800
C	-4.42649900	-0.73985900	-0.85184500
H	-4.24359300	-0.48841000	-1.89920200
H	-4.30407800	-1.81802000	-0.72450100
H	-5.44945000	-0.45617200	-0.58362700
C	-3.76429600	-0.26623200	1.90040000
H	-3.62312600	-1.33429200	2.08217100
H	-3.16016100	0.29405900	2.61765400
H	-4.82038000	-0.01157500	2.03626700
Au	-0.95566500	-0.33555900	-0.24191800
O	1.51808900	1.39965300	1.44500900
H	1.34865600	2.31700900	1.68310000
C	3.36258700	-1.19082600	0.28517200
C	3.16238000	-2.65996500	0.06464300
H	4.12896200	-3.12716900	0.29357700
H	2.42589800	-3.08685600	0.74982600
H	2.91255100	-2.92207500	-0.96436200
C	3.77779800	-0.85122400	1.69083000
H	3.19241000	-0.04676400	2.13443500
H	3.66405800	-1.73229000	2.32545400
H	4.83361900	-0.55590500	1.70251900
H	3.63867900	-0.90503500	-1.81974300

Table S91: Optimized geometry for TS_{2b}

Free energy G = -1062.000076 Hartree/particle.

C	1.73306300	1.77701600	0.08553200
C	0.75761500	2.86219800	-0.32809300
H	1.19676600	3.84632900	-0.13725900
H	0.51684700	2.78289100	-1.39326100
H	-0.17865800	2.77646400	0.23615200
O	2.02299400	1.86903000	1.47404400
H	1.19397800	1.85027500	1.96533200
C	3.05245400	1.94843700	-0.65302400
H	2.85125600	1.90883000	-1.73150300
H	3.41632600	2.95799600	-0.43017800
C	4.12779600	0.93962900	-0.27308800
H	5.06647900	1.25423800	-0.74742500
H	4.30073200	0.98689900	0.80517100
C	3.85979600	-0.46023700	-0.72216000
H	3.82115500	-0.60726000	-1.80381400
C	3.90565700	-1.57772700	0.05014300
C	4.02081000	-1.56732000	1.53507400
H	3.86428200	-0.58243700	1.97691600
H	3.29435100	-2.26344700	1.97048500
H	5.01290500	-1.93017100	1.83199700
C	3.82305200	-2.92819800	-0.57134100
H	2.99354500	-3.50606700	-0.14194100
H	3.70469300	-2.88305700	-1.65682400
H	4.73239500	-3.49752800	-0.34505200
C	1.16742200	0.39913000	-0.19170300
C	1.65616700	-0.74012700	-0.44361900
H	1.52244200	-1.77605300	-0.69804600
P	-3.26092400	-0.35969400	0.07819600
C	-4.09258300	0.89269500	1.09879000
H	-3.67712000	0.88029000	2.10914900
H	-3.93675200	1.88357500	0.66570700
H	-5.16585800	0.68180000	1.14569500
C	-4.14003200	-0.31309300	-1.51152300

H	-3.98251000	0.65677200	-1.98886800
H	-3.75660500	-1.09608100	-2.16963400
H	-5.21164400	-0.46770900	-1.34886600
C	-3.73258900	-1.94775000	0.82460400
H	-3.34475100	-2.77021900	0.21935300
H	-3.30945000	-2.02005700	1.82920100
H	-4.82339800	-2.02191700	0.88343500
Au	-0.93928200	-0.02922900	-0.09378200

Table S92: Optimized geometry for IIIb

Free energy G = -1062.019139 Hartree/particle.

P	3.21364400	0.27852300	0.05330200
C	4.03418700	0.68576200	-1.51871500
H	5.11102300	0.79853700	-1.35523500
H	3.85960800	-0.11027400	-2.24623700
H	3.62829700	1.61937000	-1.91521300
C	3.72531000	1.61525700	1.17618800
H	3.29903400	2.56292700	0.83885400
H	3.36346200	1.40743700	2.18584800
H	4.81735500	1.69315400	1.19011800
C	4.11161700	-1.18277200	0.65875400
H	3.75068500	-1.44997100	1.65494700
H	3.93839500	-2.02556900	-0.01452500
H	5.18460500	-0.97063300	0.70616900
Au	0.86047300	-0.02719200	-0.09150300
C	-1.96009700	0.82425200	-0.55365100
C	-1.85723400	-1.60583200	0.15692400
C	-3.49195700	0.64393000	-0.94126100
H	-1.42741300	1.64182100	-1.03006200
C	-3.01147700	-1.82678100	-0.82481500
C	-4.06082800	-0.73795100	-0.72574900
H	-3.65474600	1.08999200	-1.92238400
H	-3.45638400	-2.80942200	-0.62581300
H	-2.59774500	-1.86486200	-1.84196900
H	-4.55346400	-0.79568800	0.24941200
H	-4.83662900	-0.90338000	-1.47920400
C	-1.20718600	-0.25728200	-0.14189500
C	-0.85979400	-2.74192600	0.03642500
H	-0.04595700	-2.61716800	0.75807300
H	-1.35990700	-3.70001600	0.22551900
H	-0.42975100	-2.78108000	-0.96917100
O	-2.32769400	-1.52229200	1.50085000
H	-2.42641900	-2.41620800	1.84624400
C	-3.31083500	1.57442300	0.13837100
C	-3.26536600	3.03974000	-0.17832800
H	-4.24415100	3.46762500	0.06705400
H	-2.51938200	3.55409600	0.43433800
H	-3.06171100	3.23458300	-1.23387600
C	-3.63075900	1.27525000	1.56764200
H	-3.01327800	1.89050000	2.22805200
H	-4.67598000	1.56660800	1.73413400
H	-3.50346700	0.22986500	1.83798900

Table S93: Optimized geometry for TS_{3b}

Free energy G = -1062.00972 Hartree/particle.

C	4.01851900	-1.05858200	0.24735900
C	3.44838000	-0.41258200	-0.91290200
H	3.32738900	-1.14666600	-1.71449000

C	5.07164000	-0.45904300	1.06679600
H	5.05165100	-0.81645500	2.09826200
H	6.00423300	-0.84911700	0.61799000
H	5.12082300	0.62599800	1.02491200
C	3.60878900	-2.42297800	0.59922300
H	4.51028600	-3.04073700	0.70555700
H	3.15584900	-2.40881500	1.59934700
H	2.92356100	-2.87959800	-0.11288300
O	2.62578000	1.05776200	1.56322000
C	2.14504300	1.31916500	0.23194100
C	4.02810400	0.92874300	-1.34046000
H	5.11448900	0.97301200	-1.22596400
H	3.82071000	1.05616100	-2.40619400
C	3.29249300	2.00328100	-0.52023900
H	2.89204400	2.77963100	-1.17780900
H	3.94149800	2.50702400	0.20310500
H	1.92081500	0.60377500	2.04102900
C	1.97651500	-0.03266000	-0.43635100
C	0.90664100	-0.79114700	-0.64075400
H	1.09961500	-1.77138000	-1.08380800
Au	-1.09101900	-0.36901400	-0.24922200
C	-3.55479500	1.36486200	1.53834200
H	-4.61432200	1.59234100	1.69709300
H	-3.12277200	0.98568400	2.46801500
H	-3.02541000	2.27867800	1.25545800
C	-4.38136800	-1.27857200	0.79106000
H	-5.40032100	-0.93858500	1.00358100
H	-4.41153900	-2.05115400	0.01902200
H	-3.94587500	-1.70664200	1.69712700
C	-4.31096600	0.81183700	-1.16834600
H	-4.33195400	0.09710200	-1.99415700
H	-5.33577100	1.03018200	-0.85043300
H	-3.83490000	1.73235600	-1.51457300
P	-3.36349400	0.11941800	0.22282600
C	0.91157900	2.19203800	0.29935500
H	0.50448700	2.37565500	-0.69961900
H	0.12503700	1.73184800	0.90955700
H	1.18360500	3.15224900	0.75014900

Table S94: Optimized geometry for IVb

Free energy G = -1062.018204 Hartree/particle.

C	4.01285300	-0.51779500	0.54139400
C	3.36260900	-0.50900800	-0.84990200
H	3.56158300	-1.44847400	-1.37101300
C	5.45706100	-0.10970000	0.63599700
H	5.81460100	-0.19084500	1.66607100
H	6.05122600	-0.78785400	0.01447200
H	5.62701400	0.91108200	0.28987900
C	3.72309000	-1.77499900	1.31730500
H	4.35399500	-2.57602000	0.92021300
H	3.98003900	-1.65363300	2.37498100
H	2.67797400	-2.08212400	1.22577800
O	3.20593100	0.64189200	1.20406000
C	2.11071700	1.08537200	0.13827900
C	3.72731400	0.76407700	-1.62408700
H	4.80079000	0.95119900	-1.69281400
H	3.34703800	0.67645400	-2.64491400
C	2.96354200	1.86349300	-0.84840000
H	2.29522100	2.43987700	-1.49528900
H	3.61246300	2.57596300	-0.32904900

H	2.76576900	0.32516100	2.01226800
C	1.90669600	-0.24070100	-0.52320100
C	0.79771300	-0.93557200	-0.76829700
H	0.97798500	-1.87672800	-1.29809600
Au	-1.15734400	-0.43195300	-0.29941800
C	-3.37705500	1.88946100	1.04386200
H	-4.39817200	2.18113200	1.31063900
H	-2.75507000	1.88186400	1.94312900
H	-2.96278700	2.61825900	0.34195100
C	-4.22020300	-0.81109600	1.50156300
H	-5.19948900	-0.38313700	1.74034800
H	-4.35418000	-1.81661900	1.09575200
H	-3.62209000	-0.87921700	2.41350400
C	-4.55943800	0.37394000	-1.08338600
H	-4.70115900	-0.60392800	-1.54983800
H	-5.52037200	0.73721800	-0.70388700
H	-4.18131100	1.06898400	-1.83683800
P	-3.36246000	0.23430600	0.28179000
C	1.05148100	1.81797800	0.88514700
H	0.30122200	2.16517600	0.16743900
H	0.54032400	1.17525400	1.60971300
H	1.46871600	2.69282500	1.39265500

Table S95: Optimized geometry for TS_{5b}

Free energy G = -1062.019161 Hartree/particle.

C	4.04812600	-0.60951200	0.45063000
C	3.35755900	-0.37669300	-0.91025700
H	3.51283800	-1.23463100	-1.56934400
C	5.52988000	-0.32687200	0.48005900
H	5.95074200	-0.59124700	1.45419500
H	6.03015800	-0.92922700	-0.28552900
H	5.74410900	0.72629100	0.28781500
C	3.73280000	-1.97734300	1.00868200
H	2.66161300	-2.19655100	0.96758000
H	4.26077100	-2.73356500	0.41988900
H	4.07908300	-2.06497300	2.04458800
O	3.39665000	0.43175500	1.31189700
C	2.08118500	1.14568100	0.19408600
C	3.74578800	0.97583300	-1.52389400
H	4.82232900	1.14622000	-1.58297200
H	3.35152300	1.02779900	-2.54223500
C	3.02369100	1.99013700	-0.61211400
H	2.41779300	2.71148600	-1.17400000
H	3.68853300	2.57019000	0.03493900
H	2.96670700	0.00845600	2.07070100
C	1.91641200	-0.11783500	-0.52460600
C	0.79560000	-0.80699700	-0.77365400
H	0.96778900	-1.70494300	-1.37681000
Au	-1.16459900	-0.35580300	-0.28263400
C	-3.59033400	1.39848800	1.58079200
H	-4.64745500	1.62279300	1.75923300
H	-3.14269100	1.01111000	2.49957000
H	-3.06780900	2.31629500	1.29833300
C	-4.43241200	-1.23618700	0.82074700
H	-5.44768600	-0.89755500	1.05186600
H	-4.47486100	-2.00069500	0.04115000
H	-3.98398800	-1.67457700	1.71551700
C	-4.38944900	0.87107900	-1.11909500
H	-4.42949700	0.15992700	-1.94741300
H	-5.40718500	1.09319500	-0.78184600

H	-3.91554200	1.79063700	-1.47070800
P	-3.42017700	0.16542700	0.25055200
C	1.11839500	1.76721900	1.12203200
H	0.36152300	2.27831500	0.51335100
H	0.60258600	1.03246300	1.74522500
H	1.60793900	2.52375900	1.74105200

Table S96: Optimized geometry for VIb

Free energy G = -1062.036650 Hartree/particle.

C	4.10654300	1.32447000	-0.84421300
C	3.39859200	-0.03696800	-0.74746400
C	1.95023600	1.64596700	0.13188100
C	3.29493000	2.24839500	0.06057000
H	5.15926200	1.29113500	-0.56018400
H	4.05647600	1.68760400	-1.87575200
H	3.25683400	3.30044300	-0.24399100
H	3.68189500	2.24599600	1.09320200
C	0.82895100	2.40972400	0.68243700
H	-0.05419800	1.80620600	0.89923200
H	1.15910900	2.94660200	1.57884900
H	0.55297900	3.18781000	-0.04295200
C	1.96825300	0.35342800	-0.40433100
C	0.84324400	-0.39511300	-0.67892300
H	1.08165000	-1.29292700	-1.26223500
P	-3.49074500	-0.07793900	0.21462600
C	-4.18516900	1.60326100	0.20927500
H	-5.25507400	1.56505800	0.43863900
H	-3.67559100	2.21462700	0.95787800
H	-4.04188900	2.05881600	-0.77328700
C	-4.53890100	-0.99606500	-0.95415500
H	-4.40419700	-0.59626400	-1.96196100
H	-4.25568200	-2.05119900	-0.95438100
H	-5.59043900	-0.90179900	-0.66399000
C	-3.95171000	-0.74210300	1.84439000
H	-3.66056200	-1.79290800	1.91047100
H	-3.43573100	-0.18436000	2.62938900
H	-5.03365900	-0.65544000	1.98821900
Au	-1.16034700	-0.18953500	-0.25366700
O	4.05747000	-0.24027700	1.52302000
H	4.43461700	-0.79797600	2.21092200
C	4.01555900	-0.98534800	0.30170300
C	3.17006600	-2.23393100	0.49955800
H	3.06560500	-2.79659400	-0.43474100
H	3.65093500	-2.89304100	1.23137500
H	2.17264100	-1.98927800	0.87954600
C	5.42150000	-1.39393000	-0.11193700
H	6.08261000	-0.53030400	-0.21775000
H	5.85456800	-2.05873600	0.64386100
H	5.40437700	-1.93746900	-1.06258200
H	3.42976000	-0.57219200	-1.70441100

Table S97: Optimized geometry for TS_{4b}

Free energy G = -1062.010532 Hartree/particle.

C	4.55841800	0.29954800	0.17739400
C	3.56734800	-0.50820400	-0.59560700
C	2.40683900	1.45664800	0.09042600
C	3.73086100	1.39636300	0.85690100
H	5.12714400	-0.31809200	0.88071600

H	5.28965000	0.70835800	-0.53115000
H	4.23336100	2.36655600	0.85286400
H	3.52049700	1.14246200	1.89916000
C	2.45920700	2.44327900	-1.07461500
H	1.54364300	2.38791000	-1.67495300
H	2.55334400	3.45351200	-0.66604200
H	3.31565800	2.24969300	-1.72872800
C	2.30606800	0.09686600	-0.60327700
C	1.14757300	-0.74895100	-0.78381800
H	1.20129200	-1.36080800	-1.68673600
P	-3.10305100	0.21947300	0.20524600
C	-3.87300900	1.44339300	-0.89896900
H	-4.92416000	1.58462900	-0.62696100
H	-3.34624700	2.39689400	-0.81488500
H	-3.81022700	1.09784200	-1.93351400
C	-4.19236000	-1.23345500	0.08333900
H	-4.14480700	-1.64575200	-0.92738300
H	-3.86402100	-1.99963500	0.78980300
H	-5.22492100	-0.94880200	0.31133600
C	-3.42421600	0.87016500	1.87360200
H	-3.06534300	0.16057900	2.62272900
H	-2.89865800	1.81852300	2.00821000
H	-4.49886600	1.02919600	2.01047900
Au	-0.83275900	-0.24783800	-0.27974800
O	1.37405200	1.79002700	0.97762800
H	0.52762300	1.77120300	0.50658000
C	2.17157100	-1.38692300	0.22767400
C	2.51217500	-2.82487200	-0.14090300
H	3.40741200	-3.15985400	0.39199400
H	1.66577100	-3.43994900	0.18357000
H	2.64996000	-2.97701500	-1.21135300
C	2.01287600	-1.21662400	1.71526600
H	1.62999400	-0.23685400	1.99793500
H	1.29989900	-1.97354000	2.06053900
H	2.96316700	-1.40436400	2.22792400
H	3.88999700	-1.25918600	-1.30621800

Table S98: Optimized geometry for Vb

Free energy G = -1062.074679 Hartree/particle.

C	-3.98097600	-0.96678900	1.43591100
C	-2.50841900	-1.33563300	-0.46131300
C	-3.20393900	-2.05895200	0.69761500
H	-4.05465500	-1.13993500	2.51452600
H	-5.01434500	-0.88185500	1.06867900
H	-3.82661800	-2.89329800	0.35953200
H	-2.42108800	-2.45823900	1.35244500
C	-3.34560700	-1.35062700	-1.73105700
H	-2.89573900	-0.73170800	-2.51410100
H	-3.43619800	-2.37452800	-2.11302800
H	-4.35574700	-0.97821100	-1.53089100
C	-2.36749500	0.07755000	0.07921500
P	2.64396200	-0.81042400	0.20951200
C	2.37543200	-2.58189600	-0.08575000
H	3.29932200	-3.13726400	0.10507200
H	1.58301300	-2.94491300	0.57299700
H	2.06455700	-2.73497800	-1.12212700
C	4.00613600	-0.34699400	-0.89757700
H	3.69328500	-0.46285000	-1.93780000
H	4.28355700	0.69520600	-0.72494600
H	4.87055700	-0.99029100	-0.70323300

C	3.34479600	-0.70882400	1.88050400
H	3.60165400	0.32749700	2.11051700
H	2.61315900	-1.06305600	2.60995500
H	4.24627000	-1.32801100	1.93726500
Au	0.66621600	0.39590500	-0.10685300
O	-1.20755200	-1.85722000	-0.73012100
H	-1.29626000	-2.67694200	-1.22731000
C	-3.21372700	0.27148100	1.09743800
H	-3.39493900	1.22119700	1.58853000
C	-0.94469500	2.19577600	-0.11269400
C	-1.06069800	2.64114100	1.30938100
H	-1.92459700	3.31196000	1.41028600
H	-1.19615800	1.81193000	2.00687700
H	-0.17603900	3.21485700	1.60134500
C	-0.37707200	3.21511400	-1.05081900
H	-0.25827500	2.82838300	-2.06568700
H	-1.05779200	4.07592300	-1.08754200
H	0.58700900	3.59244100	-0.69520800
C	-1.50970100	1.04013400	-0.60835300
H	-1.44825100	0.90542200	-1.69149800

<u>Conditions G:</u>

Coordinates and energies for the the cyclization of **Ib** calculated at the wB97XD/6-31G(d,p) (C, H, P, O, N), SDD(Au) level taking into account solvent effect of CH_2Cl_2 (IEF-PCM) and employing PMe_3 as the phosphine. Cartesian Coordinates (in Å).

Table S99: Optimized geometry for Ib

Free energy G = -1062.194048 Hartree/particle.

C	-2.71245700	-0.97372600	0.47143400
C	-3.58805200	-2.23040100	0.38466200
H	-4.22782500	-2.27086300	1.26940200
H	-4.21711500	-2.20789800	-0.50820200
H	-2.97031600	-3.13240200	0.34947500
O	-1.93813000	-0.97786700	1.65505000
H	-1.56016800	-1.85658700	1.77326900
C	-3.58662000	0.29118000	0.49888000
H	-4.11709500	0.36190700	-0.45695600
H	-4.34157600	0.11803200	1.27257100
C	-2.85830300	1.60442400	0.80482800
H	-3.63501600	2.36100100	0.97750900
H	-2.31858900	1.50110000	1.74894300
C	-1.95659700	2.08351000	-0.29780600
H	-2.36583600	1.97806100	-1.30359800
C	-0.74835900	2.64908600	-0.18004900
C	-0.05547900	2.90831500	1.13262200
H	-0.44728400	2.30349200	1.95299700
H	1.01823700	2.71039700	1.04363200
H	-0.15281800	3.96308600	1.41540500
C	0.00857300	3.11545000	-1.39655200
H	0.95838500	2.57338400	-1.49252900
H	-0.56191800	2.96399100	-2.31640100
H	0.25955900	4.17973900	-1.31699900
C	-1.82137300	-0.97493400	-0.73782900
C	-1.28483200	-1.08080400	-1.83798500
H	-1.00911300	-1.21399300	-2.86550900
P	2.51867200	-0.23311400	0.44894800
C	3.44951900	1.15772000	-0.27127000
H	3.56319700	1.00091900	-1.34571700
H	2.91198600	2.09348900	-0.10699000
H	4.43637600	1.22237400	0.19428200

C	2.47847500	0.07181900	2.24548700
H	1.89081200	0.96702600	2.45673800
H	2.01295900	-0.78019000	2.74494600
H	3.49580700	0.20471000	2.62240700
C	3.59478900	-1.68735400	0.23216900
H	3.12550800	-2.55886600	0.69279800
H	3.73401800	-1.88150500	-0.83312800
H	4.56573700	-1.50554500	0.69989300
Au	0.40710600	-0.54912500	-0.44220800

Table S100: Optimized geometry for TS_{1b}

Free energy G = -1062.180207 Hartree/particle.

C	2.15663900	1.53371100	0.11632600
C	0.93236900	2.45276400	-0.06045600
H	1.24313900	3.47201700	0.17927600
H	0.55352900	2.42098800	-1.08401300
H	0.12843700	2.15986800	0.62117500
O	2.63486000	1.58813800	1.44345000
H	1.93541400	1.29661800	2.03841100
C	3.30313100	1.99732900	-0.78676100
H	2.94623600	2.00284200	-1.82163900
H	3.55976900	3.02450600	-0.51379600
C	4.50962700	1.06370900	-0.63941000
H	5.25270500	1.31514500	-1.40215600
H	4.97639100	1.21704100	0.33447200
C	4.06087600	-0.35400000	-0.82002600
H	3.82333700	-0.64256600	-1.84382000
C	3.98929600	-1.31783900	0.13102700
C	4.35049700	-1.12967700	1.57672600
H	4.49680800	-0.08668100	1.85042200
H	3.56057400	-1.54124000	2.21343200
H	5.26364500	-1.69356000	1.79895600
C	3.57565300	-2.71103500	-0.24005600
H	2.71528400	-3.03192400	0.36015600
H	3.33060100	-2.80665300	-1.30045300
H	4.38653300	-3.41178900	-0.01232700
C	1.75419900	0.14108700	-0.24497800
C	0.93786400	-0.80171300	-0.46979500
H	1.13560100	-1.82722600	-0.75957300
P	-3.35582300	0.07289100	0.15995600
C	-3.80530400	1.82168700	-0.09911000
H	-3.22654500	2.45023800	0.58106500
H	-3.57058300	2.10635600	-1.12688600
H	-4.87243300	1.97003800	0.08492600
C	-4.45111500	-0.87193300	-0.94998600
H	-4.21939000	-0.62567600	-1.98821200
H	-4.28703100	-1.94030900	-0.79691700
H	-5.49704800	-0.63082500	-0.74266500
C	-3.93039500	-0.32449000	1.84458200
H	-3.76085900	-1.38468600	2.04388900
H	-3.36570500	0.26536700	2.56925200
H	-4.99577000	-0.10127600	1.94384700
Au	-1.09562300	-0.33889500	-0.16863600

Table S101: Optimized geometry for IIb

Free energy G = -1062.200525 Hartree/particle.

C	4.40045700	0.90413200	-0.60170300

C	3.59645200	-0.37161300	-0.80777600
C	2.10056300	1.39668400	0.14147700
C	3.58093300	1.79662700	0.34091900
H	5.39637400	0.68403800	-0.20841400
H	4.53081200	1.38238000	-1.57554900
H	3.73722800	2.85617200	0.12636800
H	3.86010900	1.63752400	1.38302200
C	1.43775100	2.32496000	-0.88205800
H	0.41281300	2.00492000	-1.08994300
H	1.41586700	3.34617600	-0.48799700
H	1.99880800	2.33593200	-1.82096200
C	2.11978900	-0.02695100	-0.43964500
C	1.00845700	-0.76688000	-0.72759700
H	1.20642400	-1.72362600	-1.20797100
P	-3.17653300	0.13560500	0.23960400
C	-3.67214700	1.85612400	-0.11893800
H	-4.72616800	2.00972300	0.12672000
H	-3.05705600	2.53981800	0.46974900
H	-3.51207200	2.06469800	-1.17896900
C	-4.37995600	-0.88913100	-0.67361900
H	-4.24914200	-0.72961000	-1.74606600
H	-4.20163200	-1.94356400	-0.45273600
H	-5.40158500	-0.62484400	-0.38829900
C	-3.59600100	-0.11105200	1.99968600
H	-3.40880000	-1.15164700	2.27308600
H	-2.96550700	0.53232700	2.61736700
H	-4.64769500	0.12795900	2.17811800
Au	-0.93061900	-0.32451000	-0.27331400
O	1.36529400	1.38937200	1.35604400
H	1.25794900	2.30105800	1.64645400
C	3.26387300	-1.23821900	0.30700000
C	3.06551000	-2.70500000	0.02876800
H	4.01068100	-3.17976800	0.31918300
H	2.27480100	-3.14029600	0.64237700
H	2.89571900	-2.93309300	-1.02340500
C	3.54399100	-0.91799300	1.75634500
H	2.88356100	-0.14553300	2.15002700
H	3.40929700	-1.81700400	2.35892600
H	4.57975800	-0.57888500	1.86200000
H	3.72591700	-0.87776400	-1.76086600

Table S102: Optimized geometry for TS_{2b}

Free energy G = Hartree/particle.

C	1.79317800	1.72696400	0.11378300
C	0.79457800	2.86634800	-0.07757800
H	1.29458900	3.82331800	0.09122300
H	0.38092200	2.85581100	-1.08969000
H	-0.03570600	2.77565500	0.63145700
O	2.32380200	1.74270000	1.42608300
H	1.59228600	1.77375600	2.05181200
C	2.97568200	1.87655800	-0.85092500
H	2.59843600	1.78846300	-1.87503900
H	3.36217800	2.89339300	-0.73073000
C	4.13505700	0.89901200	-0.61103400
H	4.94547600	1.18606300	-1.29061100
H	4.51318600	1.04481200	0.40228800
C	3.84072400	-0.55433900	-0.86583300
H	3.73295200	-0.83947300	-1.91236100
C	3.87092100	-1.54625500	0.05853100
C	4.06581200	-1.30541200	1.53085700

H	3.74890900	-0.30579800	1.83195800
H	3.49759700	-2.03797900	2.11165000
H	5.12217600	-1.43720500	1.79266900
C	3.75611000	-2.98662900	-0.35370900
H	2.92583900	-3.47835800	0.16896500
H	3.61615200	-3.10260800	-1.43119100
H	4.66426900	-3.52685400	-0.06440400
C	1.13577000	0.37144300	-0.13589400
C	1.52274600	-0.79721300	-0.40934600
H	1.41300700	-1.83508900	-0.66010300
P	-3.25923900	-0.31700000	0.04566100
C	-3.81098600	-1.47250000	1.34404000
H	-3.36698200	-2.45454900	1.16909100
H	-3.48275200	-1.10544400	2.31875300
H	-4.90079000	-1.55793800	1.33411900
C	-4.14607600	1.23961100	0.38577100
H	-3.81005300	1.64952300	1.34042900
H	-3.92922600	1.96267900	-0.40317500
H	-5.22282700	1.05674500	0.42684300
C	-3.98904200	-0.95149200	-1.50075200
H	-3.76857100	-0.25949100	-2.31603700
H	-3.55305200	-1.92444600	-1.73579200
H	-5.07174800	-1.05355700	-1.39032400
Au	-0.95740400	-0.04145100	-0.05864400

Table S103: Optimized geometry for IIIb

Free energy G = Hartree/particle.

P	3.18514600	0.27754600	0.05074500
C	4.05654400	0.05927000	-1.53868000
H	5.13038200	0.21288400	-1.40435800
H	3.87785000	-0.94924500	-1.91733600
H	3.67477200	0.77998800	-2.26489100
C	3.68223800	1.93664800	0.62801600
H	3.30963900	2.68958700	-0.06981600
H	3.24636000	2.12343800	1.61165100
H	4.77111000	2.00807200	0.69311500
C	4.00857400	-0.87962900	1.19736700
H	3.59185600	-0.75293600	2.19886500
H	3.82767000	-1.90564300	0.86988700
H	5.08475900	-0.68996600	1.22391900
Au	0.85775600	-0.03257900	-0.08557600
C	-1.93376100	0.80811300	-0.60982500
C	-1.85047500	-1.61101500	0.15810300
C	-3.47635100	0.66324400	-0.94061300
H	-1.40035200	1.61729900	-1.09449700
C	-3.02741100	-1.82484000	-0.81213900
C	-4.06968900	-0.71754400	-0.71440400
H	-3.67562700	1.11380300	-1.91108300
H	-3.48952600	-2.79465100	-0.59809200
H	-2.62945600	-1.88117400	-1.83170900
H	-4.56008900	-0.76447900	0.26040500
H	-4.84408800	-0.87757000	-1.46837200
C	-1.18000900	-0.26417800	-0.16293400
C	-0.86185200	-2.76959000	0.03173500
H	-0.05046100	-2.65879200	0.75638500
H	-1.37560900	-3.71822600	0.22213700
H	-0.43292600	-2.81541900	-0.97320800
O	-2.29266400	-1.50896900	1.50799300
H	-2.63526200	-2.36937100	1.77449000
C	-3.26671700	1.59356400	0.14270400

C	-3.20077800	3.06730800	-0.17412200
H	-4.16827100	3.50604500	0.09148900
H	-2.43308200	3.56400300	0.42393300
H	-3.01726000	3.26056400	-1.23285600
C	-3.55668300	1.28583400	1.58478600
H	-2.93889800	1.91046600	2.23371900
H	-4.60556100	1.54978500	1.76369300
H	-3.39829900	0.24072000	1.83792600

Table S104: Optimized geometry for TS_{3b}

Free energy G = -1062.188575 Hartree/particle.

C	3.94055700	-1.13230600	0.23461900
C	3.42795900	-0.42307200	-0.92811600
H	3.30817900	-1.12944300	-1.75291800
C	5.00701500	-0.59876600	1.09586300
H	4.98975500	-1.03055800	2.09648300
H	5.93038100	-0.94862000	0.60230600
H	5.04186100	0.48643300	1.13403100
C	3.46257500	-2.49531400	0.53602000
H	4.33711700	-3.15221900	0.62074700
H	3.00061200	-2.49167700	1.52985700
H	2.76465300	-2.89027300	-0.19878800
O	2.64549000	1.03132500	1.57220300
C	2.17926800	1.33664800	0.25022700
C	4.08663700	0.90576000	-1.30368000
H	5.16709200	0.89883600	-1.14384900
H	3.92504700	1.06405100	-2.37169300
C	3.36214400	1.99947400	-0.48323400
H	2.99488100	2.78661800	-1.14486800
H	4.01262400	2.47481300	0.25538500
H	1.92705700	0.57695000	2.02754500
C	1.97237900	0.00072300	-0.45681100
C	0.87598500	-0.72917100	-0.67296500
H	1.06043700	-1.69467500	-1.15136900
Au	-1.08881900	-0.32170300	-0.24680900
C	-3.64769000	1.72595800	0.99665400
H	-4.71525600	1.87821300	1.17593800
H	-3.10873300	1.78394900	1.94500600
H	-3.27580500	2.51087500	0.33435400
C	-4.11818200	-1.10045300	1.37234500
H	-5.16864100	-0.84984600	1.54252300
H	-4.04929200	-2.10319300	0.94511300
H	-3.58272700	-1.08785900	2.32410000
C	-4.43640700	0.08044400	-1.24206700
H	-4.37440400	-0.89641700	-1.72649800
H	-5.47262100	0.27943100	-0.95591400
H	-4.10194700	0.84254100	-1.94923600
P	-3.34893600	0.09618600	0.22595000
C	0.96688900	2.25416200	0.32533000
H	0.56610000	2.45803800	-0.67070600
H	0.16932000	1.81187200	0.93074400
H	1.26635300	3.19874400	0.78759600

Table S105: Optimized geometry for IVb

Free energy G = -1062.208782 Hartree/particle.

C	4.02599200	-0.63455100	0.44620400
C	3.33940400	-0.46237500	-0.92618400
H	3.48488800	-1.34915400	-1.54355600

C	5.49254700	-0.27536400	0.54635200
H	5.86523700	-0.45842600	1.55648700
H	6.05446400	-0.90621300	-0.14762100
H	5.67630500	0.76925400	0.29314300
C	3.70256700	-1.96209500	1.10236900
H	4.27564300	-2.74229400	0.59508300
H	4.00036100	-1.96707200	2.15519300
H	2.64080300	-2.20355400	1.01576700
O	3.27782700	0.47558000	1.24266700
C	2.19279100	1.07959100	0.26903200
C	3.73904300	0.87086400	-1.58746800
H	4.81590100	1.01340200	-1.68698400
H	3.30735900	0.90788800	-2.58944400
C	3.06549500	1.92171800	-0.66161600
H	2.41939400	2.60562200	-1.21631500
H	3.77553300	2.52165600	-0.08738400
H	2.83374400	0.08596100	2.01427600
C	1.90336900	-0.16407300	-0.52552200
C	0.75850700	-0.79042900	-0.80898900
H	0.90482600	-1.67719100	-1.43396900
Au	-1.16630700	-0.32317300	-0.28878100
C	-4.22553700	1.31455700	-0.88234500
H	-5.25887400	1.49064800	-0.57153400
H	-3.68381200	2.26277000	-0.89629700
H	-4.21686100	0.89194400	-1.88931700
C	-3.62323900	0.91860200	1.91154500
H	-4.68243300	1.10735100	2.10557700
H	-3.22861100	0.24750200	2.67762600
H	-3.07366800	1.86167100	1.95416700
C	-4.49348000	-1.30583300	0.29138200
H	-4.12918400	-2.01934800	1.03374200
H	-5.51829000	-1.01555500	0.53816700
H	-4.47911800	-1.78552500	-0.68967300
P	-3.39885000	0.15719700	0.26496300
C	1.18267100	1.78894700	1.11971200
H	0.44313300	2.24347800	0.45612000
H	0.65295300	1.10348000	1.78679400
H	1.65792800	2.58243900	1.70105900

Table S106: Optimized geometry for TS_{5b}

Free energy G = -1062.206793 Hartree/particle.

C	3.98175600	-0.74118700	0.41963000
C	3.35165400	-0.28731200	-0.93077400
H	3.46837600	-1.06491400	-1.68746900
C	5.49905300	-0.62977500	0.47096700
H	5.88148300	-1.07148900	1.39454200
H	5.94090100	-1.16253200	-0.37595400
H	5.82194200	0.41211700	0.43567900
C	3.52200000	-2.14367000	0.79742200
H	2.43488000	-2.24201200	0.73468600
H	3.97306800	-2.87229500	0.11921100
H	3.84267300	-2.39044700	1.81459400
O	3.44435100	0.22990400	1.38744600
C	2.07776300	1.23412300	0.22601400
C	3.86145800	1.09568200	-1.37991700
H	4.94678100	1.19640400	-1.36520300
H	3.52803400	1.27904100	-2.40386100
C	3.15767500	2.06686000	-0.40266600
H	2.67125700	2.90847200	-0.90993500
H	3.81574000	2.49113500	0.35855500

H	2.97055300	-0.24151100	2.08457000
C	1.91425600	0.02593500	-0.55898700
C	0.77385100	-0.64056300	-0.84054300
H	0.94706500	-1.50324500	-1.49233500
Au	-1.16488400	-0.26691000	-0.31236100
C	-4.18694300	1.52288900	-0.55411800
H	-5.23116200	1.62446900	-0.24712000
H	-3.64132000	2.43133200	-0.28964000
H	-4.13773600	1.38755300	-1.63663000
C	-3.69173000	0.38268900	2.05116700
H	-4.75501400	0.54041400	2.25042700
H	-3.33846000	-0.47858000	2.62225800
H	-3.12969000	1.26556500	2.36373300
C	-4.51358900	-1.31543200	-0.13666300
H	-4.16996600	-2.20989200	0.38728100
H	-5.54301000	-1.09443200	0.15727600
H	-4.47551600	-1.50387800	-1.21174200
P	-3.41556500	0.08647100	0.26968000
C	1.12860900	1.81268200	1.20031100
H	0.42901900	2.43507500	0.62802300
H	0.55199400	1.05050900	1.72517800
H	1.64774200	2.46518700	1.90456800

Table S107: Optimized geometry for VIb

Free energy G = -1062.218928 Hartree/particle.

C	4.05039400	1.35132000	-0.89927000
C	3.37342400	-0.03106400	-0.76932300
C	1.90279900	1.63694800	0.12522800
C	3.24398100	2.26358200	0.03299500
H	5.11045600	1.34543800	-0.64724800
H	3.95517600	1.70655500	-1.92919300
H	3.18578200	3.31524100	-0.26410900
H	3.65178200	2.24184700	1.05474200
C	0.76762800	2.38740700	0.69433000
H	-0.03329800	1.74543600	1.06165100
H	1.11965100	3.05693200	1.48323900
H	0.35771500	3.02575600	-0.09969000
C	1.93516400	0.34010100	-0.40975600
C	0.82054800	-0.44148600	-0.65314200
H	1.07984600	-1.35113800	-1.20590100
P	-3.46218300	-0.04741100	0.20471000
C	-4.12356500	1.62238400	-0.12531900
H	-5.19213000	1.66080100	0.10140900
H	-3.59416000	2.35086100	0.49241200
H	-3.96652600	1.87293400	-1.17674700
C	-4.51808200	-1.16709900	-0.77614100
H	-4.35321200	-0.98304900	-1.83967300
H	-4.25475000	-2.20354200	-0.55496400
H	-5.57193300	-1.00073700	-0.53800400
C	-3.90474400	-0.38996800	1.94226300
H	-3.63319300	-1.41871300	2.18852200
H	-3.35260300	0.28594000	2.59858400
H	-4.97802900	-0.25045900	2.09482500
Au	-1.15626900	-0.23426300	-0.24027200
O	4.02772900	-0.17716000	1.50786700
H	4.42700600	-0.71088200	2.20193200
C	4.01731200	-0.94680900	0.30571300
C	3.19880400	-2.22404800	0.52100300
H	3.08820400	-2.78859300	-0.40989400
H	3.70553100	-2.87092100	1.24447000

H	2.20564800	-1.99741100	0.91933400
C	5.44732100	-1.32381100	-0.09538500
H	6.08668200	-0.44290400	-0.18136200
H	5.88464000	-1.98349600	0.66099200
H	5.45775100	-1.85741900	-1.05024500
H	3.40425300	-0.57800700	-1.71632100

Table S108: Optimized geometry for TS_{4b}

Free energy G = -1062.196188 Hartree/particle.

C	4.52028100	0.31421300	0.21930900
C	3.53231000	-0.50619500	-0.56622000
C	2.35730100	1.48546500	0.07597700
C	3.67923500	1.43104600	0.86604300
H	5.06080400	-0.29574300	0.94814800
H	5.26559600	0.70946700	-0.47849100
H	4.18797100	2.39624300	0.85183600
H	3.44755700	1.19413100	1.90621500
C	2.40675700	2.48104900	-1.09510700
H	1.48780500	2.42897400	-1.68827700
H	2.50331300	3.48744600	-0.68088700
H	3.25710000	2.29052800	-1.75571300
C	2.27775900	0.12040200	-0.61948800
C	1.13392200	-0.76163000	-0.78960600
H	1.19766400	-1.36110400	-1.69989600
P	-3.05441600	0.23097100	0.21905000
C	-3.28938100	1.87370500	0.98237900
H	-4.34845100	2.05260900	1.18566100
H	-2.72678200	1.92447200	1.91724900
H	-2.91602800	2.64460900	0.30474400
C	-4.16861300	0.23071200	-1.22768600
H	-3.83082000	0.97977200	-1.94701600
H	-4.14032800	-0.75096600	-1.70536400
H	-5.19353200	0.45610700	-0.92130700
C	-3.82602000	-0.93800200	1.39062700
H	-3.79611600	-1.94447900	0.96784300
H	-3.26810800	-0.93444100	2.32940200
H	-4.86471000	-0.65631700	1.58291800
Au	-0.82715600	-0.27032000	-0.29411800
O	1.31179000	1.80194000	0.95618900
H	0.47875700	1.81238000	0.46489400
C	2.15718800	-1.40723700	0.22798300
C	2.50078300	-2.85617300	-0.13099700
H	3.38816100	-3.18758600	0.41354100
H	1.64800800	-3.46628600	0.18123900
H	2.65441700	-3.00766800	-1.19898900
C	1.97493700	-1.21444800	1.72057800
H	1.57768400	-0.23170700	1.96974300
H	1.26220500	-1.96827600	2.06762600
H	2.91918300	-1.37720900	2.24819400
H	3.87716500	-1.23723900	-1.28601100

Table S109: Optimized geometry for Vb

Free energy G = -1062.256768 Hartree/particle.

C	-4.05508500	-0.82463500	1.42600900
C	-2.53529900	-1.29236900	-0.43695900
C	-3.30083400	-1.96702700	0.72175600
H	-4.14261900	-0.96899800	2.50677000
H	-5.07654600	-0.71551100	1.03870700

H	-3.95611300	-2.77254300	0.38045300
H	-2.55751700	-2.39237500	1.40256600
C	-3.31356900	-1.33784200	-1.75419000
H	-2.79874600	-0.77606000	-2.53887500
H	-3.42606300	-2.37341700	-2.09305700
H	-4.31393800	-0.91600100	-1.62259400
C	-2.38205900	0.13851200	0.06697000
P	2.64463800	-0.77810100	0.23178600
C	2.65845000	-2.46529400	-0.45850600
H	3.61837400	-2.94528100	-0.25109400
H	1.85166900	-3.04811500	-0.00987100
H	2.50072600	-2.41625700	-1.53782800
C	4.09252800	0.04830800	-0.50423900
H	3.94717700	0.14308100	-1.58220300
H	4.20190900	1.04541200	-0.07308300
H	4.99527700	-0.53548700	-0.30649100
C	3.04459900	-0.97879100	1.99831500
H	3.12247400	0.00398600	2.46742100
H	2.24724100	-1.54138000	2.48815500
H	3.99226700	-1.51210000	2.10950300
Au	0.64240200	0.31626200	-0.13811300
O	-1.23146000	-1.84094800	-0.62323100
H	-1.31196100	-2.68560600	-1.07736700
C	-3.23884500	0.38644100	1.06582900
H	-3.39189100	1.35297400	1.53236900
C	-0.84556600	2.18809400	-0.10954200
C	-0.95331500	2.62208100	1.32851100
H	-1.77649100	3.34093300	1.41889800
H	-1.15314100	1.79118300	2.00692400
H	-0.03937700	3.13275500	1.64112700
C	-0.25188600	3.21236000	-1.04527000
H	-0.13728900	2.82825800	-2.06091400
H	-0.92381900	4.07816300	-1.07686300
H	0.71638900	3.56743100	-0.68298600
C	-1.47127700	1.06598900	-0.62374700
H	-1.44268100	0.95439100	-1.70932200

Ligand effect

Evaluation on the ligand effect for the computed trends of gold(I) complex **Ib**.

Ligand	PMe_3	PPh_3	NHC
**Ib**	0.0	0.0	0.0
**TS_{1b}**	3.6	5.8	9.5
**IIb**	-1.1	-1.5	1.6
**TS_{2b}**	7.4	9.3	11.3
**IIIb**	-3.1	-2.5	0.1
**TS_{3b}**	2.1	4.4	7.9
**IVb**	-6.6	-4.3	-2.3
**TS_{5b}**	-6.6	-5.1	-0.7
**VIb**	-15.7	-13.9	-13.3
**TS_{4b}**	6.7	7.0	11.0
**Vb**	-37.5	-35.4	-33.1

ΔG energies are given in kcal·mol-1.

Coordinates and energies for the the cyclization of **Ib** calculated at the B3LYP/6-31G(d,p) (C, H, P, O), SDD(Au) level taking into account solvent effect of CH_2Cl_2 (IEF-PCM) and employing PPh_3 as the phosphine. ΔG energies are given in kcal·mol-1

Table S110: Optimized geometry for Ib

Free energy G = -1637.415168 Hartree/particle.

C	-3.37344100	-1.93342900	0.12781500
C	-3.27046000	-3.44815800	-0.12992700
H	-3.72537700	-3.98031100	0.70974400
H	-3.79153800	-3.72662900	-1.04929600
H	-2.22417700	-3.75749200	-0.22210400
O	-2.74171400	-1.58556000	1.35792900
H	-1.95762700	-2.14228100	1.46829200
C	-4.85832800	-1.50697800	0.23695400
H	-5.33505400	-1.65882600	-0.73823600
H	-5.31448500	-2.22466700	0.92745000
C	-5.13669000	-0.08413800	0.75769800
H	-6.20664900	-0.06300400	1.01263300
H	-4.59998100	0.06253100	1.69770700
C	-4.85925200	1.02587400	-0.22299700
H	-5.24104800	0.84664200	-1.22990900
C	-4.26566800	2.20962200	0.00564400
C	-3.70782300	2.65342100	1.33625800
H	-3.74895700	1.87881700	2.10409300
H	-2.66315100	2.97315800	1.22975400
H	-4.25926900	3.52587300	1.71105600
C	-4.13413600	3.23263100	-1.09846300
H	-3.08114500	3.49218000	-1.27270100
H	-4.55974700	2.87613700	-2.04069800
H	-4.64014200	4.16917700	-0.82893900
C	-2.71486100	-1.22475100	-1.01820700
C	-2.31026600	-0.74996900	-2.08029900
H	-2.19547500	-0.35909400	-3.07187200
P	1.60445200	0.08925000	0.07349600
Au	-0.50420600	-0.45972500	-0.76439200
C	1.58576200	1.61766100	1.07841700
C	0.45585500	1.91310600	1.85915500
C	2.69203300	2.48117900	1.10280900
C	0.44009100	3.05316000	2.66243800
H	-0.41053600	1.25830800	1.83682700
C	2.66702800	3.62305900	1.90552700
H	3.56713600	2.26884900	0.49747100
C	1.54441800	3.90904800	2.68517800
H	-0.43626700	3.27543200	3.26317000
H	3.52469900	4.28832700	1.91832500
H	1.52767700	4.79956500	3.30596500
C	2.27367100	-1.25115300	1.12496800
C	2.97822000	-0.97543100	2.30609100
C	2.08670600	-2.58470800	0.72199600
C	3.49091600	-2.02467800	3.07203900
H	3.12664200	0.04899300	2.63026500
C	2.60684400	-3.62657900	1.48835400
H	1.53821800	-2.80728400	-0.18903500
C	3.30721100	-3.34736200	2.66540900
H	4.03317700	-1.80505100	3.98640400
H	2.46053300	-4.65405900	1.17002800
H	3.70581100	-4.15991500	3.26498300
C	2.80482900	0.36315200	-1.28158400
C	2.41703000	1.14669500	-2.38231000
C	4.10001400	-0.17222200	-1.23214800
C	3.31879400	1.39345800	-3.41614700
H	1.41570200	1.56589300	-2.43199800
C	4.99654000	0.07582800	-2.27429200
H	4.41098400	-0.77916100	-0.38861800
C	4.60854000	0.85656200	-3.36410900
H	3.01312600	1.99963300	-4.26323900

H	5.99724200	-0.34258100	-2.23086800
H	5.30742900	1.04538800	-4.17314900

Table S111: Optimized geometry for TS_{1b}

Free energy G = -1637.406000 Hartree/particle.

C	3.75370000	1.49366200	0.08871200
C	2.55921500	2.45870600	-0.09379200
H	2.89289600	3.45918300	0.19341100
H	2.21644900	2.47695000	-1.13058700
H	1.72189600	2.16697100	0.54610100
O	4.21928200	1.52038700	1.43618000
H	3.49688700	1.24203100	2.01765300
C	4.93726100	1.93607700	-0.78658300
H	4.59886800	1.99008700	-1.82642500
H	5.23866700	2.94161300	-0.47867800
C	6.09827900	0.93688900	-0.64943800
H	6.86081500	1.16537900	-1.40285000
H	6.56519400	1.04686200	0.33016600
C	5.58453100	-0.45732200	-0.86181000
H	5.31189500	-0.70425300	-1.88747500
C	5.55169500	-1.47258900	0.05012100
C	5.99691700	-1.35219100	1.48209100
H	6.96898800	-1.84760000	1.60383100
H	6.08232200	-0.32094700	1.82090600
H	5.29703200	-1.87612400	2.14200000
C	5.09934000	-2.84206300	-0.36808200
H	4.24867500	-3.17519000	0.24080400
H	4.82482200	-2.88965600	-1.42439700
H	5.90115200	-3.56966700	-0.19014700
C	3.31999700	0.11457500	-0.28729000
C	2.45041200	-0.79381300	-0.49499000
H	2.62038500	-1.82739600	-0.77622300
P	-1.91210800	-0.03498500	0.02209700
Au	0.39884800	-0.35945000	-0.24261100
C	-2.49344500	1.50501100	-0.78270900
C	-1.93751800	1.86822100	-2.02126000
C	-3.48494800	2.31324100	-0.20636600
C	-2.37641200	3.01800700	-2.67679300
H	-1.16149500	1.25476800	-2.47091100
C	-3.91600600	3.46668900	-0.86543100
H	-3.91875400	2.04917700	0.75243000
C	-3.36486800	3.81931200	-2.09866400
H	-1.94181600	3.29126500	-3.63340700
H	-4.68199600	4.08832600	-0.41217100
H	-3.70087800	4.71815700	-2.60655900
C	-2.39944400	0.07921500	1.78530100
C	-1.55929500	0.77424600	2.67184400
C	-3.58646900	-0.49422600	2.26491700
C	-1.90856700	0.90124000	4.01566000
H	-0.63310800	1.21366900	2.31191600
C	-3.92780100	-0.36883400	3.61344500
H	-4.24292400	-1.03785000	1.59367000
C	-3.09230400	0.32791000	4.48829000
H	-1.25389900	1.44019000	4.69361200
H	-4.84679600	-0.81771500	3.97749600
H	-3.35969200	0.42087800	5.53645100
C	-2.88573200	-1.40379200	-0.70840600
C	-4.12875100	-1.17901200	-1.31964400
C	-2.37605700	-2.71100200	-0.63591300

C	-4.85217500	-2.25110800	-1.84595800
H	-4.53141700	-0.17387700	-1.39000300
C	-3.10545500	-3.77811100	-1.15960900
H	-1.40968300	-2.89329200	-0.17395100
C	-4.34327000	-3.54890300	-1.76620100
H	-5.81201100	-2.06937100	-2.31954500
H	-2.70396600	-4.78505600	-1.10068900
H	-4.90716500	-4.37945900	-2.17970900

Table S112: Optimized geometry for IIb

Free energy G = -1637.417530 Hartree/particle.

C	4.88394500	-1.37792300	0.14671200
C	5.09016800	-0.36437200	-0.87584600
H	5.23097500	-0.77930500	-1.87274300
C	5.11219800	-1.16702400	1.61591300
H	4.91809500	-2.08806600	2.16731500
H	6.15942500	-0.87809900	1.77358900
H	4.48375500	-0.37695900	2.02819900
C	4.68846600	-2.79713800	-0.28156800
H	5.66293100	-3.27663400	-0.09829400
H	3.95621600	-3.32710600	0.33131800
H	4.46448800	-2.91344500	-1.34170700
O	2.90087800	1.35382400	1.40673200
C	3.58317700	1.37711300	0.15182400
C	5.89474900	0.90544100	-0.57791300
H	6.89230400	0.66979200	-0.19601300
H	6.02944000	1.44006400	-1.52231700
C	5.06206300	1.74286300	0.41022700
H	5.23525200	2.81311600	0.27679800
H	5.31406900	1.50907700	1.44585500
H	1.99030200	1.06039200	1.23607600
C	3.59451700	-0.00626800	-0.54503700
C	2.49494000	-0.74328800	-0.84982800
H	2.67556700	-1.69758700	-1.34192100
Au	0.52375800	-0.33944900	-0.44231400
P	-1.78899300	-0.01274600	0.02940700
C	2.92909000	2.39695100	-0.79805500
H	3.49091600	2.48495800	-1.73343300
H	1.90397700	2.10448000	-1.04557900
H	2.91088700	3.37470400	-0.30778700
C	-2.17109200	1.61601700	0.78309500
C	-3.11969700	1.75846600	1.80675500
C	-1.49901300	2.75241200	0.30172200
C	-3.39203000	3.02124400	2.33790200
H	-3.64402200	0.89012700	2.19187300
C	-1.77939100	4.01153100	0.83156500
H	-0.75816600	2.65394300	-0.48679400
C	-2.72479300	4.14702000	1.85187600
H	-4.12578900	3.12210100	3.13176000
H	-1.25568900	4.88384900	0.45267400
H	-2.93739800	5.12685100	2.26855200
C	-2.42814300	-1.27920000	1.19327400
C	-3.70917400	-1.83498400	1.05878200
C	-1.60557000	-1.69034100	2.25604600
C	-4.16039500	-2.78488500	1.97773500
H	-4.35361500	-1.53239700	0.24003200
C	-2.06353400	-2.63483600	3.17429200
H	-0.60802400	-1.27346500	2.36425800
C	-3.34081100	-3.18441700	3.03509300

H	-5.15239100	-3.21144100	1.86488100
H	-1.42125500	-2.94576800	3.99257600
H	-3.69402500	-3.92457600	3.74665900
C	-2.82653200	-0.12906000	-1.47900800
C	-2.44899700	-1.03147300	-2.48757600
C	-3.98627600	0.64365000	-1.64395400
C	-3.22628000	-1.16486200	-3.63802500
H	-1.54780800	-1.62750000	-2.37385300
C	-4.75799100	0.50947000	-2.79994400
H	-4.28687100	1.35009000	-0.87703400
C	-4.38058300	-0.39358900	-3.79594700
H	-2.92640600	-1.86436300	-4.41233200
H	-5.65270100	1.11251000	-2.92069000
H	-4.98150700	-0.49336700	-4.69475200

Table S113: Optimized geometry for TS_{2b}

Free energy G = -1637.400380 Hartree/particle.

P	-1.80406400	-0.11901700	0.01235900
Au	0.51336400	0.17759500	-0.18525200
C	3.04392100	-0.61094200	-0.51960500
C	3.23128000	1.92681600	-0.00090600
C	5.36495800	-0.48471100	-0.58558600
H	2.88946900	-1.64065400	-0.77496300
C	4.70310700	1.97380500	-0.46177100
C	5.58936200	0.84525800	0.08845700
H	5.38610700	-0.45609200	-1.67585400
H	5.10648500	2.93779000	-0.13090000
H	4.73628500	1.98060600	-1.55777100
H	5.44951900	0.77937300	1.17028700
H	6.63788400	1.12681100	-0.07624200
C	2.63062400	0.56443300	-0.29053100
C	2.42456800	3.03855800	-0.68392600
H	1.39713300	3.04740600	-0.31033000
H	2.88412800	4.01145000	-0.47745700
H	2.40301100	2.90060800	-1.76866500
O	3.14679000	2.04892000	1.42732100
H	3.34392700	2.96982800	1.65383500
C	5.38764500	-1.71956000	-0.00472800
C	5.31865900	-2.95878800	-0.85411600
H	6.19595400	-3.59070200	-0.66984600
H	4.44565900	-3.56969800	-0.58601900
H	5.27567600	-2.73113000	-1.92181900
C	5.47593300	-1.96105300	1.47557400
H	4.70195300	-2.66878500	1.79476400
H	6.44007200	-2.42884600	1.71302100
H	5.38538300	-1.05250700	2.07064500
C	-2.66016000	1.42086400	0.51431400
C	-3.72058500	1.41100800	1.43260900
C	-2.24836500	2.63749800	-0.05538900
C	-4.36119400	2.60467800	1.77240300
H	-4.04601000	0.47966900	1.88398500
C	-2.89543800	3.82498100	0.28408000
H	-1.42346400	2.65645800	-0.76203400
C	-3.95117600	3.80999000	1.19983500
H	-5.17950000	2.58953000	2.48567600
H	-2.57108300	4.76079000	-0.16059400
H	-4.44980700	4.73626900	1.46853100
C	-2.57865600	-0.65943000	-1.55676100
C	-3.88025000	-0.26898300	-1.90775000
C	-1.85339200	-1.50230100	-2.41521400

C	-4.44759900	-0.72252700	-3.10014000
H	-4.44937300	0.38810100	-1.25832000
C	-2.42738200	-1.95551600	-3.60270800
H	-0.84064300	-1.79927400	-2.15740000
C	-3.72418100	-1.56515100	-3.94663700
H	-5.45401200	-0.41444200	-3.36625600
H	-1.85980400	-2.60552700	-4.26144600
H	-4.16760900	-1.91277200	-4.87472500
C	-2.21611800	-1.38307700	1.27365100
C	-1.45263100	-1.42961300	2.45264900
C	-3.27314500	-2.28879600	1.09817600
C	-1.75137600	-2.36255100	3.44490600
H	-0.62513700	-0.73972200	2.59369100
C	-3.56320600	-3.22474900	2.09338700
H	-3.86720500	-2.26993400	0.19054700
C	-2.80575100	-3.26222000	3.26550800
H	-1.15682000	-2.39133100	4.35281100
H	-4.38109300	-3.92399700	1.94929700
H	-3.03294300	-3.99286200	4.03580800

Table S114: Optimized geometry for IIIb

Free energy G = -1637.419102 Hartree/particle.

P	1.76817600	-0.06928000	-0.00255000
Au	-0.58853600	0.20016600	0.16645300
C	-3.35809100	-0.73097500	0.69532700
C	-3.37321300	1.66512600	-0.11403800
C	-4.91307000	-0.69242000	0.95306900
H	-2.81827700	-1.56319600	1.12959400
C	-4.59903700	1.83333800	0.81500500
C	-5.57918000	0.66446400	0.71376500
H	-5.11954800	-1.13965600	1.92630300
H	-5.11149600	2.76937100	0.56344000
H	-4.24090100	1.93839400	1.84570500
H	-6.06304600	0.68053000	-0.26504800
H	-6.37133200	0.78421100	1.45842300
C	-2.64654400	0.35657800	0.24211400
C	-2.45195900	2.88194100	0.01678600
H	-1.59925700	2.79130800	-0.66159800
H	-3.00306600	3.79724200	-0.22929000
H	-2.07468800	2.98274000	1.03870600
O	-3.77853300	1.51753300	-1.48783100
H	-4.13934200	2.36658700	-1.78350400
C	-4.76423400	-1.65366200	-0.12036300
C	-4.61237700	-3.10900400	0.22100000
H	-5.57055600	-3.59139400	-0.01421900
H	-3.84989500	-3.59249600	-0.39545600
H	-4.39883400	-3.27915400	1.27761800
C	-5.03023800	-1.35299800	-1.56315900
H	-4.42906900	-1.99473300	-2.21185000
H	-6.08543500	-1.60498100	-1.74326300
H	-4.86552900	-0.30972300	-1.82304800
C	2.44958100	-1.16142900	1.30489500
C	3.70417000	-0.93069000	1.88918500
C	1.68955000	-2.26902100	1.71746200
C	4.19108600	-1.79946400	2.86825700
H	4.30005900	-0.07584000	1.58639800
C	2.18323100	-3.13662500	2.69139100
H	0.71277300	-2.45123500	1.27775300
C	3.43375100	-2.90178300	3.26915300
H	5.16193200	-1.61208400	3.31675100

H	1.58897100	-3.98990700	3.00384100
H	3.81425300	-3.57368400	4.03249200
C	2.25590800	-0.81896800	-1.60398700
C	3.32429300	-1.72206600	-1.71030600
C	1.53598300	-0.46234800	-2.75699400
C	3.66919200	-2.25517800	-2.95414600
H	3.88415400	-2.01361200	-0.82768700
C	1.88842600	-0.99349000	-3.99756600
H	0.69912200	0.22675400	-2.68343900
C	2.95425000	-1.89163700	-4.09714900
H	4.49586400	-2.95524600	-3.02721700
H	1.32633000	-0.71227500	-4.88288300
H	3.22347200	-2.31022800	-5.06217700
C	2.68394500	1.51500100	0.12415400
C	2.22922800	2.47852300	1.04052100
C	3.82024100	1.78524200	-0.65312700
C	2.90721300	3.68900300	1.18176400
H	1.34543700	2.28257700	1.64149500
C	4.49162200	3.00177100	-0.51197600
H	4.18040200	1.05350600	-1.36880700
C	4.03789300	3.95278400	0.40399600
H	2.54856400	4.42741200	1.89235100
H	5.36828300	3.20408400	-1.11969600
H	4.56089200	4.89847600	0.50918500

Table S115: Optimized geometry for TS_{3b}

Free energy G = -1637.408203 Hartree/particle.

C	-5.55984700	-1.21346400	-0.13884800
C	-4.96969200	-0.49099500	0.98363000
H	-4.80671200	-1.19394600	1.80465000
C	-6.69489700	-0.70337000	-0.93704900
H	-6.69746900	-1.09793200	-1.95476300
H	-7.59042500	-1.11435500	-0.43691800
H	-6.78231600	0.37916100	-0.94211000
C	-5.11077600	-2.59634500	-0.42330100
H	-5.96080600	-3.26145600	-0.20746400
H	-4.90054900	-2.72512900	-1.49036700
H	-4.25748000	-2.91090700	0.17487600
O	-4.36906100	0.93703400	-1.51360500
C	-3.77088400	1.27209300	-0.24048200
C	-5.60578600	0.84508900	1.40297900
H	-6.68991700	0.85311200	1.27025300
H	-5.41889000	0.98166700	2.47083200
C	-4.88841100	1.95191400	0.58249500
H	-4.45130700	2.69751400	1.25087600
H	-5.56150700	2.47974100	-0.09842100
H	-3.68516900	0.50671400	-2.04923800
C	-3.51064800	-0.05638700	0.46963900
C	-2.40092800	-0.76327200	0.69518700
H	-2.55374000	-1.73298800	1.17510100
Au	-0.41407300	-0.35481400	0.33362300
P	1.90953700	-0.02524600	-0.02126300
C	-2.56058300	2.17584600	-0.45408300
H	-2.08688100	2.43116000	0.49696400
H	-1.81058400	1.69311600	-1.08768900
H	-2.88732100	3.09846400	-0.94324500
C	2.36761800	0.00017900	-1.79955700
C	3.53814000	-0.60389000	-2.28185900
C	1.51054900	0.65646800	-2.69945000
C	3.84677000	-0.54711400	-3.64305400

H	4.20749600	-1.11984100	-1.60147400
C	1.82664600	0.71657700	-4.05634600
H	0.59625700	1.11884500	-2.33818700
C	2.99457800	0.11286000	-4.53023900
H	4.75375600	-1.01995300	-4.00732400
H	1.15855800	1.22752300	-4.74304700
H	3.23669100	0.15357500	-5.58793300
C	2.92541200	-1.34755800	0.74604100
C	4.18550300	-1.09126100	1.30797800
C	2.42021400	-2.65863800	0.75693100
C	4.92859000	-2.13342800	1.86657500
H	4.58692700	-0.08310500	1.31534900
C	3.16877600	-3.69693700	1.31128000
H	1.43990800	-2.86504700	0.33615500
C	4.42327800	-3.43516900	1.86805100
H	5.90153700	-1.92546600	2.30137000
H	2.76909200	-4.70645900	1.31537400
H	5.00264400	-4.24239800	2.30589000
C	2.52779000	1.55543900	0.67849700
C	1.99255000	2.00274900	1.89856600
C	3.51971700	2.31652200	0.04202600
C	2.45114200	3.18703200	2.47530100
H	1.21689000	1.42652800	2.39560300
C	3.97120200	3.50460300	0.62094300
H	3.93827800	1.98751700	-0.90350300
C	3.43967200	3.94027100	1.83632400
H	2.03141900	3.52429200	3.41819100
H	4.73759800	4.08822000	0.12000800
H	3.79105500	4.86563700	2.28271600

Table S116: Optimized geometry for IVb

Free energy G = -1637.421966 Hartree/particle.

C	5.61753500	-0.78054800	0.19447700
C	4.90186500	-0.27933800	-1.08684000
H	5.04657000	-0.98097000	-1.90962200
C	7.09367500	-0.46325100	0.35176300
H	7.47356700	-0.85644100	1.29812200
H	7.64346800	-0.94559500	-0.46235000
H	7.29492700	0.60777000	0.31621000
C	5.29775300	-2.22696500	0.53610100
H	5.85971000	-2.87662100	-0.14150600
H	5.61308400	-2.46893800	1.55626100
H	4.23447400	-2.44696400	0.41889800
O	4.88404400	0.12649600	1.25247900
C	3.72324300	0.93171100	0.43995400
C	5.28361200	1.18688600	-1.40849600
H	6.35917500	1.36501200	-1.45143100
H	4.86789400	1.45333100	-2.38283200
C	4.57796200	1.98662900	-0.26962100
H	3.91315100	2.76174600	-0.66095100
H	5.27038500	2.46578100	0.42758800
H	4.47107600	-0.43707900	1.93358100
C	3.45928100	-0.10478400	-0.61596900
C	2.31892400	-0.65854800	-1.05173000
H	2.46701100	-1.36836800	-1.87134400
Au	0.36504000	-0.34761700	-0.50048400
P	-1.93079900	-0.01839400	0.02880100
C	2.71641100	1.38117400	1.45780400
H	1.95856100	1.97871900	0.94379300
H	2.20502500	0.53888500	1.93098000

H	3.18489200	2.00861900	2.22066500
C	-2.49822900	-0.97577100	1.48957700
C	-1.59831700	-1.18147300	2.54868200
C	-3.79881500	-1.49475100	1.58207200
C	-1.99727900	-1.88547100	3.68501300
H	-0.58548200	-0.79392300	2.48152900
C	-4.19194500	-2.20322800	2.71937900
H	-4.50302600	-1.35136100	0.76895300
C	-3.29410400	-2.39833000	3.77099700
H	-1.29420400	-2.03979100	4.49799200
H	-5.19950800	-2.60313200	2.78096000
H	-3.60204800	-2.95239400	4.65259500
C	-2.32383700	1.73854500	0.39331700
C	-3.23441300	2.11440300	1.39207800
C	-1.68817200	2.73428000	-0.36845100
C	-3.50695200	3.46528900	1.62052600
H	-3.72914300	1.35881000	1.99343300
C	-1.96852500	4.08145900	-0.14076600
H	-0.97385200	2.45493900	-1.13829200
C	-2.87724700	4.44871900	0.85544600
H	-4.21155600	3.74635300	2.39737000
H	-1.47337100	4.84269100	-0.73605100
H	-3.09033400	5.49791400	1.03661800
C	-3.04397100	-0.50589800	-1.34839200
C	-2.71638500	-1.64911100	-2.09700400
C	-4.20469800	0.21498400	-1.66615500
C	-3.54341600	-2.06868100	-3.13875200
H	-1.81380300	-2.20851900	-1.86643200
C	-5.02633600	-0.20575600	-2.71437800
H	-4.46816000	1.10346300	-1.10163100
C	-4.69859700	-1.34640200	-3.44982000
H	-3.28146200	-2.95326700	-3.71131200
H	-5.92148700	0.35990000	-2.95462600
H	-5.33818700	-1.66948900	-4.26563300

Table S117: Optimized geometry for TS_{5b}

Free energy G = -1637.423236 Hartree/particle.

C	5.59709200	-0.81738000	0.20707900
C	4.90731100	-0.22002200	-1.05887900
H	5.02789700	-0.88732300	-1.91415400
C	7.11346000	-0.66033200	0.26391600
H	7.51371500	-1.15788700	1.15143100
H	7.56458900	-1.12291600	-0.61923700
H	7.41433600	0.38772100	0.29912600
C	5.18442400	-2.26604900	0.45509300
H	4.10086500	-2.39600400	0.40126600
H	5.64506500	-2.91117200	-0.29833600
H	5.53542600	-2.60307700	1.43632400
O	5.03318600	0.04734500	1.30031100
C	3.64599700	1.07240500	0.36462300
C	5.35762300	1.23680100	-1.33371800
H	6.43921400	1.37665600	-1.32544600
H	4.99665800	1.53810100	-2.32027100
C	4.63884800	2.05024300	-0.21967400
H	4.06989400	2.89911800	-0.61898500
H	5.30655200	2.44766600	0.54839000
H	4.60696200	-0.51520700	1.96837100
C	3.46480700	-0.00449900	-0.60918900
C	2.32511600	-0.61126400	-1.00892300
H	2.48925100	-1.35218700	-1.79745500

Au	0.36429300	-0.30990900	-0.49107700
P	-1.94154100	-0.01784600	0.01972700
C	2.70491500	1.47934200	1.43966200
H	1.97319500	2.16389700	0.99257100
H	2.15723800	0.63450900	1.85982700
H	3.22576200	2.03022300	2.22667000
C	-2.27513100	-0.00346900	1.82584200
C	-1.60765200	-0.94098200	2.63307900
C	-3.16718900	0.90248700	2.41755500
C	-1.83962500	-0.97689200	4.00764200
H	-0.90769200	-1.64189300	2.18609900
C	-3.39017600	0.86728600	3.79629600
H	-3.68632900	1.63489800	1.80843400
C	-2.72974500	-0.07085600	4.59133600
H	-1.32171600	-1.70664300	4.62258800
H	-4.08109800	1.57390000	4.24599300
H	-2.90478600	-0.09484700	5.66266200
C	-2.99591500	-1.35088600	-0.67243200
C	-2.65519300	-1.89331200	-1.92306200
C	-4.13208500	-1.82836500	-0.00114000
C	-3.44575400	-2.88940500	-2.49626100
H	-1.77104100	-1.53867700	-2.44565100
C	-4.91700900	-2.82948300	-0.57716600
H	-4.40349900	-1.42542500	0.96922700
C	-4.57653200	-3.35941200	-1.82340600
H	-3.17403500	-3.30297300	-3.46269700
H	-5.79312200	-3.19458100	-0.04991500
H	-5.18739300	-4.13978300	-2.26708800
C	-2.61975200	1.55647200	-0.63728900
C	-1.78069300	2.68269900	-0.67822000
C	-3.94383700	1.66887100	-1.08848300
C	-2.26217200	3.90292200	-1.15262900
H	-0.75032600	2.60224100	-0.34331500
C	-4.41949100	2.89112000	-1.56772600
H	-4.60254100	0.80674400	-1.07221400
C	-3.58174700	4.00793800	-1.59939000
H	-1.60537900	4.76707200	-1.18112000
H	-5.44440100	2.96785200	-1.91769400
H	-3.95414500	4.95585700	-1.97592400

Table S118: Optimized geometry for VIb

Free energy G = -1637.437332 Hartree/particle.

C	5.67446300	1.27065600	-0.94997700
C	4.94477400	-0.09298200	-0.82714200
C	3.54019700	1.63180600	0.09820900
C	4.91007700	2.20630500	0.00366800
H	6.73626300	1.22205200	-0.70920800
H	5.58361500	1.64026400	-1.97610900
H	4.89711000	3.26409100	-0.28088700
H	5.33267400	2.15904200	1.01961600
C	2.43610200	2.41578800	0.68853500
H	1.52727000	1.83624700	0.85088600
H	2.77382500	2.86292900	1.63124700
H	2.20874300	3.25999000	0.02273800
C	3.51760300	0.33311000	-0.44709100
C	2.37197700	-0.40870400	-0.70156600
H	2.59567900	-1.32292400	-1.26010900
P	-1.97018800	-0.05197100	0.02335700
Au	0.38476100	-0.17512500	-0.32876200
O	5.65125900	-0.29446200	1.44291100

H	6.05683700	-0.85251900	2.12094100
C	5.58425000	-1.05432400	0.22282300
C	4.73017200	-2.31126800	0.45137000
H	4.59720000	-2.88395300	-0.47217500
H	5.22599800	-2.96629800	1.17624300
H	3.74547500	-2.05949800	0.85549200
C	6.99598400	-1.47407700	-0.21762800
H	7.65915700	-0.61324700	-0.32680700
H	7.43551000	-2.14260300	0.53056000
H	6.96853700	-2.01390800	-1.16940900
H	4.94057800	-0.62680800	-1.78226900
C	-2.42064100	0.05674000	1.79771000
C	-1.67322700	-0.68627100	2.72739400
C	-3.49060200	0.84599500	2.24554100
C	-1.99923700	-0.64605800	4.08280800
H	-0.83809100	-1.29544300	2.39253900
C	-3.80913000	0.88622200	3.60468300
H	-4.07237000	1.43021800	1.54020100
C	-3.06647400	0.14141800	4.52294200
H	-1.41642900	-1.22334900	4.79398000
H	-4.63742500	1.50095200	3.94342500
H	-3.31555600	0.17701200	5.57914800
C	-2.84473900	-1.51903800	-0.64393400
C	-3.95592900	-2.08182400	0.00203800
C	-2.38761500	-2.08043400	-1.84824700
C	-4.60128000	-3.18843900	-0.55414000
H	-4.31655600	-1.66338800	0.93598700
C	-3.03943700	-3.18209400	-2.40164500
H	-1.52237200	-1.65756800	-2.35141100
C	-4.14595300	-3.73809000	-1.75421000
H	-5.45894800	-3.61973800	-0.04707300
H	-2.67920500	-3.60960000	-3.33232900
H	-4.64895600	-4.59997300	-2.18203300
C	-2.72213600	1.40971800	-0.79170200
C	-3.97140500	1.34519500	-1.42670200
C	-2.02337700	2.62866000	-0.76334500
C	-4.51430600	2.48715000	-2.01979100
H	-4.51915200	0.40935900	-1.46197500
C	-2.57329300	3.76708700	-1.35179500
H	-1.05146600	2.68738400	-0.28157600
C	-3.81857200	3.69702300	-1.98213100
H	-5.48069200	2.42845100	-2.51105200
H	-2.02721800	4.70505900	-1.32418500
H	-4.24284100	4.58234100	-2.44590600

Table S119: Optimized geometry for TS_{4b}

Free energy G = -1637.404075 Hartree/particle.

C	5.93182300	0.25210900	0.21599400
C	4.96619800	-0.56600100	-0.61997100
C	3.75897000	1.43288000	0.04105800
C	5.06917300	1.36203800	0.86315600
H	6.45849300	-0.36234000	0.95228800
H	6.69478900	0.66740700	-0.45082200
H	5.57915300	2.32746000	0.87591500
H	4.81448300	1.11335200	1.89506300
C	3.82920900	2.48645400	-1.08963300
H	2.92995100	2.45806900	-1.71330200
H	3.90051800	3.47180600	-0.62112900
H	4.70245800	2.34211400	-1.73231200
C	3.70339700	0.09974800	-0.71687200

C	2.58432500	-0.77391800	-0.93942500
H	2.66637000	-1.37016700	-1.84842700
P	-1.69203200	0.01905400	0.02629200
Au	0.59393300	-0.37316000	-0.45491600
O	2.67932900	1.69991000	0.91111900
H	1.84676800	1.62741600	0.41236000
C	3.68827200	-1.48087400	0.07415400
C	3.93202900	-2.92865400	-0.39387800
H	4.78459900	-3.34581200	0.15001700
H	3.04086500	-3.51699300	-0.15693100
H	4.12452700	-3.00374300	-1.46433300
C	3.46481000	-1.39335800	1.57728800
H	3.09648400	-0.41975800	1.89570500
H	2.71948600	-2.14390900	1.85740600
H	4.38886800	-1.62750300	2.11582700
H	5.36021100	-1.19640500	-1.40910100
C	-2.80895900	-0.76306000	-1.20061900
C	-2.42295500	-1.98220900	-1.78259500
C	-4.03676000	-0.18585100	-1.55976400
C	-3.25829300	-2.61811300	-2.70112400
H	-1.46921500	-2.43162400	-1.51978700
C	-4.86715100	-0.82458600	-2.48283400
H	-4.34402300	0.75987000	-1.12520700
C	-4.48048200	-2.03955200	-3.05261300
H	-2.95131500	-3.55943400	-3.14661600
H	-5.81456300	-0.37051800	-2.75665200
H	-5.12731600	-2.53190300	-3.77241900
C	-2.18746100	-0.63367600	1.66827600
C	-3.43887900	-1.22996900	1.88481300
C	-1.28320700	-0.51944900	2.73822000
C	-3.78025000	-1.70075600	3.15455300
H	-4.14531500	-1.33118500	1.06761500
C	-1.63209000	-0.98549400	4.00559900
H	-0.30760900	-0.06775000	2.58135400
C	-2.88006500	-1.57858200	4.21467100
H	-4.74979100	-2.16321700	3.31228200
H	-0.92750900	-0.89177300	4.82632700
H	-3.14774000	-1.94749200	5.20017600
C	-2.12015700	1.80384400	0.03144800
C	-1.56534300	2.62757400	-0.96336000
C	-2.98548600	2.36180900	0.98367200
C	-1.88005100	3.98534600	-1.00754100
H	-0.88919400	2.20811500	-1.70357600
C	-3.29192800	3.72400300	0.93861800
H	-3.41874300	1.74020800	1.76014600
C	-2.74216000	4.53575000	-0.05493000
H	-1.44779900	4.61337200	-1.78057000
H	-3.96071100	4.14780000	1.68151800
H	-2.98118400	5.59440900	-0.08599500

Table S120: Optimized geometry for Vb

Free energy G = -1637.471641 Hartree/particle.

C	5.33513900	0.90238000	1.40840400
C	3.77518800	1.30719300	-0.45650700
C	4.51356800	2.01102800	0.71289600
H	5.38987600	1.02500600	2.49593100
H	6.37392600	0.87743400	1.04970600
H	5.12317100	2.85483600	0.37772300
H	3.75247000	2.39412400	1.39957600
C	4.50993600	1.47012700	-1.79566200

H	4.02840900	0.89265900	-2.59059300
H	4.51495000	2.52413600	-2.09692200
H	5.55056800	1.14281100	-1.71145800
C	3.73766700	-0.15203100	0.00550000
P	-1.50461600	0.14856100	0.05382400
Au	0.67604000	-0.62473200	-0.28034200
O	2.41557100	1.75520200	-0.59363800
H	2.41476300	2.62585900	-1.01575100
C	4.61486100	-0.35727400	1.00509700
H	4.84465700	-1.32110300	1.44549800
C	2.40009900	-2.34260500	-0.25376000
C	2.52743000	-2.81840700	1.17193400
H	3.41378400	-3.46112800	1.25829900
H	2.63720600	-1.99856800	1.88295800
H	1.66306400	-3.42715000	1.45122900
C	1.93200000	-3.38751400	-1.24066500
H	1.80686600	-2.98313200	-2.24751000
H	2.68530900	-4.18547300	-1.28426000
H	0.99537800	-3.85232700	-0.91975200
C	2.91052800	-1.13400200	-0.71510400
H	2.86998800	-0.98596400	-1.79538500
C	-2.71792900	-0.68095400	-1.03847600
C	-2.52074700	-2.02795000	-1.38355700
C	-3.85311000	-0.00420300	-1.51192400
C	-3.45173500	-2.69011800	-2.18349400
H	-1.63922600	-2.55535200	-1.03070000
C	-4.77904600	-0.67155600	-2.31589300
H	-4.01425900	1.03883600	-1.25991900
C	-4.58061500	-2.01248900	-2.65120500
H	-3.29130400	-3.73098700	-2.44694600
H	-5.65316200	-0.14105200	-2.68054500
H	-5.30103300	-2.52713900	-3.27958600
C	-2.05395100	-0.13665700	1.77732800
C	-3.38454000	-0.46424700	2.07887500
C	-1.11883900	-0.00523600	2.81766200
C	-3.77191800	-0.65588000	3.40662700
H	-4.11571000	-0.57454500	1.28489100
C	-1.51384100	-0.19175100	4.14201200
H	-0.08474100	0.23978500	2.59140100
C	-2.83985500	-0.51968300	4.43725800
H	-4.80206700	-0.91331800	3.63256900
H	-0.78563700	-0.08854200	4.94049500
H	-3.14434400	-0.67265800	5.46809900
C	-1.66838800	1.94484900	-0.26882300
C	-1.10071800	2.47378900	-1.44096600
C	-2.35101700	2.79372600	0.61393400
C	-1.22447000	3.83237400	-1.72666000
H	-0.56244600	1.82665500	-2.12803700
C	-2.46538700	4.15578700	0.32458700
H	-2.79236000	2.39952900	1.52296100
C	-1.90482500	4.67552700	-0.84282000
H	-0.78534600	4.23338700	-2.63499500
H	-2.99376700	4.80712400	1.01387900
H	-1.99440400	5.73474400	-1.06371300

Coordinates and energies for the the cyclization of **Ib** calculated at the B3LYP/6-31G(d,p) (C, H, P, O), SDD(Au) level taking into account solvent effect of CH_2Cl_2 (IEF-PCM) and employing (1,3-diphenyl)imidazol-2-ylidene (NHC) as the ligand. ΔG energies are given in kcal·mol-1

Table S121: Optimized geometry for Ib

Free energy G = -1289.458116 Hartree/particle.

Au	0.66577500	-0.34224200	-0.69647500
C	3.25914900	2.45068000	1.18351700
C	2.17274400	3.24500700	1.00469300
H	4.21690100	2.65018100	1.63564300
H	2.00209900	4.28036100	1.25063900
C	1.66789200	1.20599000	0.17086900
N	1.20749400	2.46903300	0.37867200
N	2.93332400	1.20320300	0.66947400
C	-0.09530400	2.95488600	0.00667500
C	-2.20872100	3.91454500	0.64318600
C	-1.73885700	3.43972200	-1.68396400
C	-2.60560300	3.91515000	-0.69656000
H	-2.87889700	4.28533700	1.41200700
H	-2.04013500	3.44997300	-2.72656500
H	-3.58625300	4.29063600	-0.97118900
C	3.82686900	0.07482700	0.67730000
C	5.92781900	-0.90749700	0.03341100
C	4.35251900	-2.16928800	1.37003400
C	5.57904300	-2.08075700	0.70688300
H	6.88006600	-0.83546300	-0.48211700
H	4.08438700	-3.07511800	1.90441400
H	6.26400000	-2.92269100	0.71907900
C	-0.47398100	2.96156700	-1.33778000
H	0.21646100	2.61001400	-2.09685500
C	-0.94932800	3.43147700	1.00271600
H	-0.63583700	3.41396900	2.04146900
C	5.05087000	0.17814100	0.01349700
H	5.30665400	1.08994500	-0.51671700
C	3.47008700	-1.08756900	1.36444100
H	2.52465400	-1.13633000	1.89368100
C	-0.18682400	-1.83559600	-2.10534700
H	0.11353400	-1.75580100	-3.13168300
C	-0.69643400	-2.17341800	-1.03343900
C	-1.39954600	-2.79760600	0.13079200
C	-2.44714300	-1.83406600	0.72960200
H	-2.85493700	-2.33541000	1.61371400
H	-1.91389300	-0.94497100	1.08509200
C	-2.01666400	-4.12564400	-0.34514000
H	-1.23712100	-4.81142200	-0.69060900
H	-2.54318900	-4.58442400	0.49610300
H	-2.72188900	-3.97284300	-1.16503900
O	-0.45878100	-3.02764700	1.18068000
H	0.15121900	-3.72556900	0.89922200
C	-3.59264500	-1.41599700	-0.21121100
H	-4.19718700	-2.28882700	-0.47140900
H	-3.16467100	-1.04098300	-1.15187600
C	-4.44124000	-0.33130000	0.40148000
H	-3.92367400	0.61947400	0.54079000
C	-5.72289500	-0.39938100	0.79610200
C	-6.40823900	0.80618500	1.39537400
H	-7.29146900	1.08922200	0.80755800
H	-6.76932000	0.58937200	2.40934900
H	-5.74198000	1.67176600	1.44790300
C	-6.59606800	-1.62607000	0.69297500
H	-6.98562900	-1.90199400	1.68127900
H	-7.47152800	-1.42439400	0.06209400
H	-6.07907700	-2.49526400	0.28244300

Table S122: Optimized geometry for TS_{1b}

Free energy G = -1289.443049 Hartree/particle.

C	-3.28129400	0.66881300	0.93818700
C	-2.21041200	1.55018300	1.62040100
H	-2.70668500	2.43349300	2.03582300
H	-1.71529900	1.01655100	2.43465300
H	-1.46222000	1.87178200	0.89370400
O	-3.86603300	1.36325600	-0.17126100
H	-4.32102700	2.14371100	0.17860100
C	-4.36014700	0.24449200	1.95478000
H	-3.87133300	-0.25558100	2.79790800
H	-4.85857800	1.13610100	2.35092600
C	-5.35788700	-0.70599400	1.27447800
H	-6.01199300	-1.15483300	2.03020600
H	-5.99255900	-0.14808900	0.58421100
C	-4.59231500	-1.78594000	0.56450000
H	-4.13253400	-2.53523400	1.20757200
C	-4.56526300	-2.02007100	-0.78345800
C	-5.26298000	-1.16804500	-1.80762400
H	-4.67183400	-1.11483600	-2.72711500
H	-6.22009800	-1.63566000	-2.07412700
H	-5.44963700	-0.15395000	-1.45832200
C	-3.85839700	-3.22876900	-1.32167300
H	-3.07105600	-2.93601900	-2.02824900
H	-3.42500900	-3.85039300	-0.53516900
H	-4.56480400	-3.84343700	-1.89371100
C	-2.62706400	-0.54301800	0.37072400
C	-1.58983900	-1.18571100	-0.01394500
H	-1.57879300	-2.20562400	-0.38540800
Au	0.29867200	-0.28186300	-0.05249400
C	2.20625800	0.47665900	-0.12874700
N	2.59093500	1.78101200	-0.20726300
N	3.37113700	-0.22294900	-0.03239900
C	4.46577400	0.63485100	-0.03046500
H	5.47848400	0.26970500	0.01977900
C	3.97580800	1.89377700	-0.14019000
H	4.47563900	2.84625600	-0.20573600
C	1.70614000	2.90667800	-0.32366900
C	0.78037400	2.95959900	-1.36865700
C	1.80737200	3.94669300	0.60311700
C	-0.06483600	4.06569500	-1.47300700
H	0.73449500	2.15351300	-2.09253900
C	0.96423600	5.05225600	0.48230500
H	2.52829000	3.88400000	1.41192000
C	0.02623000	5.11167200	-0.55132300
H	-0.78478300	4.11303300	-2.28387600
H	1.03788700	5.86212800	1.20106300
H	-0.62878000	5.97260800	-0.64097200
C	3.47735900	-1.65207800	0.07238900
C	4.16012500	-2.20498000	1.15795000
C	2.92266300	-2.46409800	-0.92004100
C	4.28083400	-3.59231200	1.25224300
H	4.57935800	-1.55803900	1.92187900
C	3.04094900	-3.85059900	-0.80958900
H	2.42005600	-2.01387000	-1.76912000
C	3.71896000	-4.41557800	0.27340700
H	4.80835000	-4.02714300	2.09516700
H	2.61385700	-4.48643100	-1.57866000
H	3.81336600	-5.49406400	0.35179000

Table S123: Optimized geometry for IIb

Free energy G = -1289.455487 Hartree/particle.

C	-5.03369100	-1.55793500	1.09568700
C	-3.93366200	-2.22731300	0.26870600
C	-3.16525000	0.05567600	1.00208900
C	-4.69983100	-0.05603100	1.14967100
H	-6.02564600	-1.75518600	0.67858800
H	-5.01183400	-1.99808900	2.09666500
H	-5.04221100	0.39999000	2.08149600
H	-5.17945000	0.49064500	0.33646100
C	-2.49588700	0.15501000	2.38541600
H	-1.40524900	0.16246300	2.29666600
H	-2.82007600	1.08108900	2.86939300
H	-2.77719100	-0.68857300	3.02390900
C	-2.69986100	-1.26004500	0.32858300
C	-1.42045700	-1.53900300	-0.05542700
H	-1.27218000	-2.52817300	-0.48743100
Au	0.24735000	-0.36377400	-0.01398300
O	-2.87422900	1.21580300	0.22294900
H	-1.90927800	1.27530000	0.12923400
C	-3.77529600	-1.93940500	-1.14849700
C	-3.18514100	-3.00254000	-2.02790600
H	-4.04977400	-3.46074300	-2.53121900
H	-2.53976000	-2.59503800	-2.80901700
H	-2.67203400	-3.79337600	-1.48089300
C	-4.44664300	-0.80492500	-1.87793100
H	-4.13061300	0.17435300	-1.51771900
H	-4.22718700	-0.86437400	-2.94527500
H	-5.53261100	-0.88234400	-1.74113400
H	-3.70990100	-3.25894200	0.53464100
C	2.00123400	0.74770700	-0.04728400
N	2.15550800	2.09934600	-0.12406100
N	3.27423800	0.26146700	-0.01339700
C	4.20510300	1.29278600	-0.06405900
H	5.26678000	1.10737700	-0.05641800
C	3.50113500	2.44902200	-0.13923700
H	3.82498300	3.47578900	-0.18869700
C	3.63034300	-1.12999900	0.05587300
C	4.41480300	-1.57773100	1.12079600
C	3.21102400	-2.00534400	-0.94902400
C	4.77639500	-2.92459200	1.18207100
H	4.72704500	-0.88267000	1.89359300
C	3.57053100	-3.35197200	-0.87214600
H	2.62230800	-1.63197900	-1.77987000
C	4.35225400	-3.81234200	0.19033600
H	5.38406800	-3.27841900	2.00877800
H	3.24924000	-4.03672400	-1.65065000
H	4.63425500	-4.85923600	0.24266900
C	1.08685400	3.05889100	-0.18792500
C	0.99626100	3.89856600	-1.29983700
C	0.17744300	3.15845000	0.86772200
C	-0.02692500	4.84648200	-1.35625700
H	1.71227200	3.80393100	-2.10996300
C	-0.84791700	4.10354200	0.79582900
H	0.28401900	2.51489500	1.73423000
C	-0.95061300	4.94712700	-0.31318000
H	-0.10337600	5.50076100	-2.21881100
H	-1.55872300	4.18488600	1.61167600
H	-1.74614700	5.68392100	-0.36206700

Table S124: Optimized geometry for TS_{2b}

Free energy G = -1289.440073 Hartree/particle.

C	-1.74435700	-2.67513500	0.09512900
C	-0.65765800	-3.44698200	-0.66060600
H	-0.73377700	-4.51156000	-0.42034300
H	-0.76656900	-3.32313400	-1.74230300
H	0.33878900	-3.09280100	-0.37870000
O	-1.57989600	-2.83367000	1.50802100
H	-0.66809500	-2.59652100	1.73212300
C	-3.13170700	-3.24147100	-0.24630100
H	-3.22944700	-3.29872700	-1.33634600
H	-3.14767900	-4.26791900	0.13304800
C	-4.31989100	-2.46738500	0.34615600
H	-5.21568100	-3.09389500	0.24179900
H	-4.16173200	-2.32665700	1.41797700
C	-4.59794800	-1.16126400	-0.35370000
H	-4.66148600	-1.22762000	-1.44057300
C	-5.03595800	0.00135200	0.21467000
C	-5.13861500	0.23749000	1.69432200
H	-4.71162100	-0.56568800	2.29449000
H	-4.64755400	1.17928600	1.96630300
H	-6.19437000	0.34728100	1.97366300
C	-5.44120500	1.16224100	-0.65008800
H	-4.82240100	2.04442700	-0.43411500
H	-5.37134600	0.93421700	-1.71616300
H	-6.47343600	1.45656300	-0.42500400
C	-1.67650000	-1.18297000	-0.22202400
C	-2.50888200	-0.23842000	-0.40197900
H	-2.72933500	0.78290200	-0.64277200
Au	0.14512100	-0.06504500	-0.18866800
C	1.94104100	0.91374900	-0.02850400
N	2.13791800	2.25721700	0.07908400
N	3.18083500	0.36855000	0.10524900
C	3.48135300	2.54408800	0.29581800
C	4.13645600	1.35756700	0.31257300
H	3.84180700	3.55553300	0.38828800
H	5.18300500	1.12550100	0.42348000
C	1.11055300	3.25866500	0.00108200
C	0.30126700	3.33608600	-1.13520100
C	0.95744300	4.15790000	1.05853200
C	-0.68643700	4.32036900	-1.20094500
H	0.45515000	2.64539900	-1.95715700
C	-0.02706500	5.14397000	0.97700400
H	1.59280100	4.07841000	1.93483200
C	-0.85127900	5.22398200	-0.14815300
H	-1.31566000	4.38837300	-2.08280500
H	-0.15177800	5.84416700	1.79675500
H	-1.61598100	5.99211500	-0.20679600
C	3.48505900	-1.03524700	0.05798900
C	3.16142600	-1.77656500	-1.08079400
C	4.12952500	-1.62734200	1.14637000
C	3.47587700	-3.13628200	-1.11865100
H	2.68435200	-1.29054500	-1.92483700
C	4.44609100	-2.98567600	1.09352900
H	4.37057500	-1.03429600	2.02283000
C	4.11730500	-3.74109100	-0.03483200
H	3.22974300	-3.71774200	-2.00142500
H	4.94500000	-3.45201200	1.93700900
H	4.36467200	-4.79731200	-0.07137000

Table S125: Optimized geometry for IIIb

Free energy G = -1289.457997 Hartree/particle.

Au	-0.04840700	-0.07831900	-0.07741500
C	2.70340500	-0.93679700	0.64820500
C	2.65250400	0.08406100	-1.68119900
C	4.21055300	-1.34844000	0.46720300
H	2.13652500	-1.45025400	1.41527300
C	3.79567300	-0.89194000	-2.00767700
C	4.83203000	-1.01358000	-0.88709900
H	4.36657400	-2.36834500	0.81758200
H	4.28040200	-0.55720300	-2.93040100
H	3.35132800	-1.87236700	-2.21294200
H	5.40905900	-0.08840000	-0.82012600
H	5.54167800	-1.80718100	-1.13848000
C	1.96490000	-0.31948300	-0.36316800
C	1.66673900	0.13663800	-2.85137500
H	0.87860400	0.87431300	-2.66649100
H	2.19916000	0.41294200	-3.76676200
H	1.18554200	-0.83405700	-3.00520000
O	3.24375000	1.38525500	-1.49952000
H	2.52564200	2.02948100	-1.41137700
C	4.08801600	-0.34879800	1.51780800
C	4.00933000	-0.81740300	2.95301700
H	5.00592700	-0.70097900	3.39622000
H	3.31650200	-0.20520500	3.53712500
H	3.72303000	-1.86781400	3.03827000
C	4.47969400	1.09687200	1.35917800
H	3.91393700	1.72244600	2.05510000
H	5.54006600	1.17920200	1.63260000
H	4.34121800	1.47135600	0.34786400
C	-2.09263500	0.17324900	0.20435400
N	-2.78863900	1.34173300	0.27885900
N	-3.04720700	-0.79755600	0.24541500
C	-4.15752000	1.10318600	0.34618200
H	-4.87469500	1.90323600	0.42991900
C	-4.32005500	-0.24233000	0.32445300
H	-5.20727700	-0.85121400	0.38420400
C	-2.20618000	2.65439800	0.26522100
C	-1.23397100	2.99412600	1.20967100
C	-2.64089000	3.58052200	-0.68570100
C	-0.67887700	4.27485700	1.18487400
H	-0.93096100	2.27015900	1.95815200
C	-2.08620700	4.86121100	-0.69406800
H	-3.39281700	3.29679100	-1.41501600
C	-1.10310400	5.20830200	0.23586100
H	0.07447900	4.54520200	1.91806800
H	-2.41890300	5.58330900	-1.43284500
H	-0.67316100	6.20490400	0.22462800
C	-2.79430700	-2.21043800	0.18089400
C	-1.96267100	-2.81187100	1.12884200
C	-3.40705900	-2.96484400	-0.82208100
C	-1.72990000	-4.18634400	1.05572700
H	-1.51849600	-2.21272300	1.91619400
C	-3.17458300	-4.33991300	-0.87937900
H	-4.04664200	-2.47899100	-1.55202300
C	-2.33395600	-4.95070100	0.05443900
H	-1.08683300	-4.65976700	1.79094800
H	-3.64607800	-4.93014000	-1.65861900
H	-2.15434300	-6.02004900	0.00511800

Table S126: Optimized geometry for TS_{3b}

Free energy G = -1289.44547 Hartree/particle.

C	-4.97121500	0.05242200	0.80334100
C	-4.19689000	1.27325000	0.61018900
H	-4.01658300	1.73384100	1.58472200
C	-6.13808300	-0.31593900	-0.02749900
H	-6.31433200	-1.39295100	-0.04402600
H	-7.00037600	0.14118800	0.49081100
H	-6.10692200	0.08584500	-1.03627600
C	-4.68335200	-0.81902500	1.96728400
H	-5.59150600	-0.86192800	2.58611700
H	-4.51169100	-1.84772200	1.62970400
H	-3.84733500	-0.47899100	2.57562100
O	-3.72931100	-0.58892100	-1.62708600
C	-2.98055800	0.63373400	-1.42685600
C	-4.64428700	2.24930500	-0.48922400
H	-5.73096600	2.31139500	-0.58358000
H	-4.29451600	3.24603800	-0.20911700
C	-3.94835600	1.78241400	-1.79707200
H	-3.39029900	2.60732000	-2.24619500
H	-4.65559200	1.42206100	-2.54925800
H	-3.14057500	-1.32513400	-1.40083600
C	-2.76807300	0.76328900	0.08152600
C	-1.70842800	0.55948300	0.87054400
H	-1.91033700	0.66537500	1.94039200
Au	0.24770100	0.10342300	0.43962300
C	-1.72363000	0.61895700	-2.29217400
H	-1.13789400	1.53063200	-2.15048400
H	-1.08390800	-0.23510300	-2.05077700
H	-2.01772700	0.54907300	-3.34397100
C	2.23126100	-0.36562400	0.04754600
N	3.27180300	0.49711300	-0.13232500
N	2.76800000	-1.59678400	-0.19074500
C	4.11390400	-1.49961900	-0.53144700
C	4.43049300	-0.18293700	-0.49507200
H	4.71712800	-2.36881900	-0.73636700
H	5.36480000	0.32730000	-0.66289800
C	3.19984600	1.92484700	0.00321800
C	3.61648800	2.72964800	-1.05972200
C	2.74089500	2.48984200	1.19589300
C	3.56438900	4.11806400	-0.92687700
H	3.96445500	2.27343900	-1.98101800
C	2.68371200	3.87952900	1.31369300
H	2.44446400	1.84860200	2.01857700
C	3.09474300	4.69426900	0.25586100
H	3.88450600	4.74671500	-1.75173900
H	2.32873200	4.32321000	2.23858300
H	3.05342300	5.77449600	0.35448800
C	2.05393000	-2.84036000	-0.12768700
C	1.36100100	-3.18840500	1.03469900
C	2.08642200	-3.69903200	-1.22911300
C	0.67922000	-4.40546200	1.08331800
H	1.36848100	-2.51965900	1.88817200
C	1.40995500	-4.91827300	-1.16519300
H	2.62406400	-3.40984600	-2.12651800
C	0.70290900	-5.27102500	-0.01313400
H	0.14177400	-4.68112900	1.98526000
H	1.43153200	-5.58720600	-2.01968000
H	0.17690900	-6.21952400	0.03201900

Table S127: Optimized geometry for IVb

Free energy G = -1289.458878 Hartree/particle.

C	4.84880300	-1.34769600	0.22832300
C	4.16421600	-0.83910900	-1.06830600
H	4.15180800	-1.62080200	-1.82962500
C	6.36372100	-1.27208900	0.30502900
H	6.72168800	-1.66923400	1.25829200
H	6.78818700	-1.88131700	-0.49890200
H	6.73331800	-0.25223200	0.19554500
C	4.31372900	-2.68947500	0.70453300
H	3.22520100	-2.74233800	0.63333300
H	4.73811100	-3.47738300	0.07548000
H	4.62406600	-2.89278500	1.73482300
O	4.33003700	-0.25206900	1.22810000
C	3.27258600	0.67530200	0.37729100
C	4.76910500	0.50907300	-1.53189400
H	5.85623500	0.50034900	-1.62765900
H	4.35627100	0.76203900	-2.51111500
C	4.26163400	1.50627600	-0.44495400
H	3.72167200	2.35206900	-0.87969400
H	5.05634900	1.91101400	0.18725500
H	3.84903300	-0.68456200	1.95799700
C	2.79412700	-0.38784000	-0.56504100
C	1.55752500	-0.79249200	-0.89598100
H	1.56874400	-1.58656500	-1.65111700
Au	-0.31561900	-0.20457000	-0.31016600
C	2.40960600	1.36946400	1.38781900
H	1.72806000	2.03453900	0.85072000
H	1.79864100	0.66594200	1.95938100
H	3.01472000	1.97562900	2.06720500
C	-2.28358000	0.29070200	0.14210000
N	-3.33864900	-0.56842200	0.25514600
N	-2.84746200	1.52814300	0.24679700
C	-4.22750400	1.44207500	0.40217900
H	-4.84615800	2.31687000	0.51825400
C	-4.53629900	0.12311400	0.40799200
H	-5.47865100	-0.38437600	0.53324100
C	-3.25500400	-2.00043500	0.19621700
C	-2.39560700	-2.68587700	1.05881300
C	-4.05962000	-2.69109100	-0.71321500
C	-2.33179100	-4.07867300	0.99335800
H	-1.79592600	-2.13436400	1.77427500
C	-3.99521800	-4.08449600	-0.76249900
H	-4.71669900	-2.14336800	-1.38130300
C	-3.12981700	-4.77940000	0.08562600
H	-1.66620500	-4.61497900	1.66247500
H	-4.61686700	-4.62358500	-1.47028500
H	-3.08066300	-5.86291400	0.04245000
C	-2.13708300	2.77502600	0.18732300
C	-2.46792700	3.69956700	-0.80557700
C	-1.15604900	3.06129900	1.13929900
C	-1.79918300	4.92415900	-0.84853200
H	-3.23137400	3.45689500	-1.53775900
C	-0.48860400	4.28611600	1.08307100
H	-0.93298600	2.33829000	1.91616900
C	-0.80835900	5.21725500	0.09150100
H	-2.05097000	5.64486600	-1.61999600
H	0.27110900	4.51655800	1.82340600
H	-0.29074900	6.17072000	0.05499500

Table S128: Optimized geometry for TS_{5b}

Free energy G = -1289.459186 Hartree/particle.

C	4.82805600	-1.36268300	0.26601200
C	4.16626500	-0.82945200	-1.04278400
H	4.14151400	-1.60771600	-1.80769000
C	6.35194900	-1.41076400	0.26826200
H	6.71746300	-1.84684200	1.20181700
H	6.70001600	-2.03574600	-0.55987600
H	6.79209000	-0.41836400	0.16206400
C	4.23126800	-2.69264200	0.71742600
H	3.13875200	-2.67125800	0.70984500
H	4.56329600	-3.48801400	0.04410800
H	4.57789400	-2.94890100	1.72452000
O	4.43433500	-0.29089500	1.25793500
C	3.21215000	0.78528800	0.27389000
C	4.81281100	0.49644400	-1.51551500
H	5.90169700	0.46580400	-1.57320400
H	4.43918600	0.73479500	-2.51441600
C	4.29402100	1.53343400	-0.47739800
H	3.82785300	2.40487000	-0.95228300
H	5.06340600	1.91044200	0.20074400
H	3.94687700	-0.69729400	1.99468800
C	2.79981000	-0.34271200	-0.56833200
C	1.55983400	-0.80338900	-0.84535600
H	1.58049900	-1.64287700	-1.54929100
Au	-0.31609400	-0.20840700	-0.29115700
C	2.39643100	1.46377000	1.31600100
H	1.75005900	2.18895900	0.80784800
H	1.75169800	0.76890500	1.85674100
H	3.03196700	2.01851600	2.01073200
C	-2.28701100	0.29201000	0.13892500
N	-3.34454800	-0.56451600	0.24389600
N	-2.84727700	1.53095700	0.24008600
C	-4.22824800	1.44864700	0.38606000
H	-4.84524100	2.32517600	0.49763400
C	-4.54086000	0.13033900	0.38921900
H	-5.48548800	-0.37448900	0.50783200
C	-3.26388000	-1.99682300	0.18386500
C	-2.41015100	-2.68501900	1.04987900
C	-4.06521700	-2.68427800	-0.73085400
C	-2.34845300	-4.07785200	0.98230900
H	-1.81365700	-2.13599800	1.76995000
C	-4.00305800	-4.07768000	-0.78200500
H	-4.71781100	-2.13427400	-1.40145000
C	-3.14313000	-4.77550900	0.06933300
H	-1.68738700	-4.61647900	1.65400000
H	-4.62209100	-4.61449400	-1.49377800
H	-3.09574600	-5.85902100	0.02459900
C	-2.13224500	2.77577600	0.18487200
C	-2.44265900	3.69419900	-0.82019200
C	-1.16746200	3.06542900	1.15229700
C	-1.76937500	4.91644800	-0.85983800
H	-3.19412300	3.44878800	-1.56376900
C	-0.49544000	4.28806200	1.09939500
H	-0.96077100	2.34768500	1.93864900
C	-0.79451800	5.21306500	0.09563300
H	-2.00537400	5.63258600	-1.64046300
H	0.25113100	4.52207300	1.85192500
H	-0.27353700	6.16477700	0.06196100

Table S129: Optimized geometry for VIb

Free energy G = -1289.477097 Hartree/particle.

C	5.07131200	0.87075900	-0.95329700
C	4.25238200	-0.43145900	-0.75133300
C	2.97920500	1.42719300	0.09570900
C	4.38313300	1.90733000	-0.04671400
H	6.13034900	0.76306100	-0.71905100
H	4.99380700	1.18932500	-1.99757600
H	4.42875400	2.94328300	-0.40074600
H	4.82089200	1.90097900	0.96313300
C	1.93977200	2.30906000	0.66730600
H	1.00252400	1.79548800	0.88062200
H	2.32871600	2.78326100	1.57639400
H	1.73950900	3.12876500	-0.03604100
C	2.86243800	0.11076500	-0.38136700
C	1.66198600	-0.56377300	-0.59134400
H	1.82760000	-1.52109500	-1.09671000
Au	-0.28927000	-0.16538700	-0.24258800
O	4.99408100	-0.56312600	1.51415400
H	5.35876600	-1.11811100	2.21747400
C	4.84311300	-1.37904700	0.33851100
C	3.90634200	-2.55591400	0.65357400
H	3.71226100	-3.16826800	-0.23289500
H	4.37113000	-3.20360500	1.40531500
H	2.95174200	-2.21019000	1.06029400
C	6.21003900	-1.92618900	-0.10412200
H	6.93004500	-1.12414400	-0.28092400
H	6.61933400	-2.57847600	0.67503700
H	6.11945100	-2.51973800	-1.01944800
H	4.19886300	-1.01237300	-1.67732700
C	-2.31802200	0.14077500	0.06953100
N	-2.97718800	1.32739600	0.19008100
N	-3.29120200	-0.80494500	0.19583000
C	-4.53517600	-0.21725000	0.39046000
H	-5.43035100	-0.80253900	0.52327500
C	-4.33726500	1.12464000	0.39011400
H	-5.02863400	1.94445300	0.49763100
C	-3.08305100	-2.22591400	0.13184500
C	-2.20175000	-2.84302100	1.02298600
C	-3.79051400	-2.97034900	-0.81465400
C	-2.01603000	-4.22462300	0.94849300
H	-1.68195400	-2.25054800	1.76796600
C	-3.60384800	-4.35228000	-0.87315700
H	-4.46767600	-2.47206600	-1.50107000
C	-2.71509200	-4.97947500	0.00334500
H	-1.33370000	-4.71016100	1.63898100
H	-4.14878500	-4.93497200	-1.60893600
H	-2.57120000	-6.05417500	-0.04684000
C	-2.37045200	2.62781600	0.10293000
C	-2.48448900	3.50405000	1.18433600
C	-1.70560800	3.00630600	-1.06587300
C	-1.91557500	4.77559600	1.09388800
H	-3.00347600	3.19005700	2.08439700
C	-1.13274700	4.27734700	-1.14159600
H	-1.65214800	2.31971600	-1.90379300
C	-1.23730000	5.16177000	-0.06487500
H	-1.99963200	5.46023700	1.93181500
H	-0.61810700	4.57904700	-2.04843200
H	-0.79645600	6.15151400	-0.13088300

Table S130: Optimized geometry for TS_{4b}

Free energy G = -1289.440554 Hartree/particle.

C	-5.04338700	-1.78981200	0.36578600
C	-3.67538800	-2.42854300	0.24096400
C	-3.30063200	-0.22341600	1.16614800
C	-4.76398500	-0.29787400	0.66419900
H	-5.65132200	-1.94105900	-0.53130600
H	-5.57362400	-2.27853600	1.19055900
H	-5.45762700	0.11490200	1.39973600
H	-4.84712100	0.30639400	-0.24113500
C	-3.20396300	-0.31719600	2.70733500
H	-2.16040300	-0.35434400	3.03535900
H	-3.67078000	0.57913300	3.12478400
H	-3.72453700	-1.19621900	3.09903400
C	-2.66025100	-1.53240300	0.68361600
C	-1.35873100	-1.74608100	0.09894800
H	-0.97355700	-2.75114500	0.27707100
Au	0.19420000	-0.36659900	-0.03151100
O	-2.70651400	0.96800000	0.69763100
H	-1.74283600	0.91554800	0.83437300
C	-2.53815600	-1.98936500	-1.01510900
C	-2.27748800	-3.31067200	-1.76205600
H	-3.15349600	-3.57576900	-2.36102000
H	-1.42787200	-3.15273600	-2.43312700
H	-2.04045200	-4.13878500	-1.09408700
C	-2.91400700	-0.85839100	-1.96096800
H	-2.90805800	0.11603300	-1.47591900
H	-2.18210200	-0.83593300	-2.77465300
H	-3.89740700	-1.03889300	-2.40739400
H	-3.58486900	-3.50661400	0.30810300
C	1.83552200	0.89269500	-0.09679500
N	3.14219900	0.51933300	0.01697800
N	1.87418500	2.25410000	-0.14573200
C	3.18091900	2.72073100	-0.04409000
C	3.97825600	1.62968200	0.05832400
H	3.41756400	3.77142100	-0.08224300
H	5.04990900	1.53777400	0.12710100
C	0.73423300	3.11921500	-0.27078100
C	0.52448800	4.10765300	0.69368900
C	-0.12439600	2.98633500	-1.36469900
C	-0.56461700	4.97014600	0.56119200
H	1.20010600	4.19236800	1.53889600
C	-1.21578700	3.84872000	-1.48161800
H	0.07019600	2.22805200	-2.11529600
C	-1.43642800	4.84025900	-0.52258800
H	-0.73281600	5.73842600	1.30912500
H	-1.88553000	3.75106100	-2.33016500
H	-2.28334800	5.51195100	-0.62141800
C	3.61249500	-0.83531600	0.10249300
C	3.31101100	-1.74128000	-0.91762500
C	4.38768000	-1.21779000	1.19940600
C	3.78033100	-3.05265900	-0.82430800
H	2.72976800	-1.41690800	-1.77392900
C	4.85951100	-2.52910500	1.27729700
H	4.60738500	-0.50108500	1.98426900
C	4.55373100	-3.44765800	0.27014800
H	3.55117900	-3.76019900	-1.61494400
H	5.46023800	-2.83183100	2.12902700
H	4.92124400	-4.46694100	0.33537300

Table S131: Optimized geometry for Vb

Free energy G = -1289.510806 Hartree/particle.

C	4.26397900	-2.23468600	-1.35784900
C	1.83244900	-2.43989400	-1.68335300
C	3.14951100	-2.09462500	-2.41589500
H	5.06953800	-1.50242500	-1.48260100
H	4.74038100	-3.22486300	-1.39469500
H	3.30683500	-2.71593900	-3.30123400
H	3.07903000	-1.05273900	-2.74316500
C	1.41032900	-3.90206100	-1.88676300
H	0.51227800	-4.14746700	-1.30719900
H	1.19160400	-4.08100100	-2.94394800
H	2.20540500	-4.58593800	-1.57538700
C	2.18785100	-2.14751800	-0.21361600
Au	-0.07233000	-0.17969000	0.57913400
O	0.80721200	-1.56705600	-2.19066800
H	-0.05688300	-1.94296100	-1.97235100
C	3.52223400	-2.06199800	-0.05853200
H	4.03855200	-1.94485000	0.88773800
C	1.15925800	-1.54581900	2.08461900
C	2.30199600	-0.70767600	2.60529300
H	3.00755800	-1.35624700	3.14171400
H	2.84875400	-0.19791800	1.81101600
H	1.93773400	0.03211700	3.32345200
C	0.17559200	-2.00670900	3.13844800
H	-0.65281700	-2.57834400	2.71387600
H	0.70673900	-2.65288900	3.85004300
H	-0.22506200	-1.16478400	3.71061600
C	1.13836000	-2.12765100	0.81942000
H	0.33038800	-2.84212400	0.65183300
C	-1.18357600	1.37050700	-0.15446200
N	-0.74910100	2.61558500	-0.48729100
N	-2.49256100	1.34207800	-0.52522000
C	-1.77010800	3.35044200	-1.07661400
H	-1.62787600	4.37004200	-1.39559800
C	-2.86546000	2.55027300	-1.10122900
H	-3.86998100	2.73276500	-1.44633900
C	-3.37496400	0.21601200	-0.37996100
C	-3.60593200	-0.32509500	0.88700100
C	-4.00189800	-0.30485700	-1.51389500
C	-4.46575900	-1.41768900	1.01260700
H	-3.12968300	0.11256700	1.75751400
C	-4.86637400	-1.39186000	-1.37428600
H	-3.80705800	0.12828000	-2.48969800
C	-5.09486300	-1.95173800	-0.11485700
H	-4.65241300	-1.84122300	1.99434000
H	-5.35492000	-1.80267800	-2.25197100
H	-5.76649200	-2.79809700	-0.01135000
C	0.58453100	3.11595500	-0.28858800
C	1.31530700	3.55251200	-1.39530900
C	1.11549300	3.18041200	1.00152900
C	2.60367700	4.05392100	-1.20390900
H	0.88453600	3.48899100	-2.38941100
C	2.40832500	3.67594500	1.17911000
H	0.51906500	2.86090300	1.84927500
C	3.15206400	4.11207300	0.07975900
H	3.17866300	4.39221000	-2.05987100
H	2.82706000	3.73242800	2.17884200
H	4.15493800	4.50143600	0.22387000

10. References

[1] Y. Imana, M. Yuasa, I. Nakamura, S.-I. Murahashi, *J. Org. Chem.* **1994**, *59*, 2282-2284.

[2] J. Tummatorn, S. Ruchiwarat, P. Ploypradith, *Chem. Eur. J.* **2010**, *16*, 1445-1448.

[3] Y. Sonoda, M. Goto, S. Tsuzuki, N. Tamaoki, *J. Phys. Chem. A* **2006**, *110*, 13379-13387.

[4] P. Magnus, T. Rainey, Tetrahedron 2001, 57, 8647-8651

[5] T. Janecki, A. Albrecht, E. Warzycha, K. Studzian, A. Jannecka, U. Krajewska, M. Rózalski, *Chem. Biodiversity* **2005**, *2*, 1256-1265.

[6] M. L. Hammond, R. A. Zambias, M. N. Chang, N. P. Jensen, J. McDonald, K. Thompson, D. A. Boulton, I. E. Kopka, K. M. Hand, E. E. Opas, S. Luell, T. Bach, P. Davies, D. E. MacIntyre, R. J. Bonney, J. L. Humes, *J. Med. Chem.* **1990**, *33*, 908-918.

[7] Frisch, M. J.; Trucks, G. W.; Schlegel, H. B.; Scuseria, G. E.; Robb, M. A.; Cheeseman, J. R.; Scalmani, G.; Barone, V.; Mennucci, B.; Petersson, G. A.; Nakatsuji, H.; Caricato, M.; Li, X.; Hratchian, H. P.; Izmaylov, A. F.; Bloino, J.; Zheng, G.; Sonnenberg, J. L.; Hada, M.; Ehara, M.; Toyota, K.; Fukuda, R.; Hasegawa, J.; Ishida, M.; Nakajima, T.; Honda, Y.; Kitao, O.; Nakai, H.; Vreven, T.; Montgomery (Jr.), J. A.; Peralta, J. E.; Ogliaro, F.; Bearpark, M.; Heyd, J. J.; Brothers, E.; Kudin, K. N.; Staroverov, K. N.; Kobayashi, R.; Normand, J.; Raghavachari, K.; Rendell, A.; Burant, J. C.; Iyengar, S. S.; Tomasi, J.; Cossi, M.; Rega, N.; Millam, N. J.; Klene, M.; Knox, J. E.; Cross, J. B.; Bakken, V.; Adamo, C.; Jaramillo, J.; Gomperts, R.; Stratmann, R. E.; Yazyev, O.; Austin, A. J.; Cammi, R.; Pomelli, C.; Ochterski, J. W.; Martin, R. L.; Morokuma, K.; Zakrzewski, V. G.; Voth, G. A.; Salvador, P.; Dannenberg, J. J.; Dapprich, S.; Daniels, A. D.; Farkas, O.; Foresman, J. B.; Ortiz, J. V.; Cioslowski, J.; Fox, D. J.; Gaussian 09, revision 02; Gaussian, Inc.: Wallingford, CT, 2009.

[8] D. Andrae, U. Haussermann, M. Dolg, H. Stoll and H.Preuss, *Theor. Chim. Acta* **1990,** *77*, 123–141.

[9] (a) E. Cances, B. Mennucci and J. J. Tomasi, Chem. Phys. **1997**, *107*, 3032–3041. (b) 12 M. Cossi, V. Barone, B. Mennucci and J. Tomasi, Chem. Phys. Lett. **1998**, *286*, 253-260. (c) B. Mennucci and J. J. Tomasi, *Chem. Phys.* **1997**, *106*, 5151–5158. (d) S. Miertus and J. Tomasi, *J. Chem. Phys.* **1982**, *65*, 239–245.
